# Analysis of a Gene Regulatory Cascade Mediating Circadian Rhythm in Zebrafish

**DOI:** 10.1371/journal.pcbi.1002940

**Published:** 2013-02-28

**Authors:** Ying Li, Guang Li, Haifang Wang, Jiulin Du, Jun Yan

**Affiliations:** 1 CAS-MPG Partner Institute for Computational Biology, Shanghai Institutes of Biological Sciences, Chinese Academy of Sciences, Shanghai, China; 2 Institute of Neuroscience, Shanghai Institutes of Biological Sciences, Chinese Academy of Sciences, Shanghai, China; NNF Center for Protein Research, Denmark

## Abstract

In the study of circadian rhythms, it has been a puzzle how a limited number of circadian clock genes can control diverse aspects of physiology. Here we investigate circadian gene expression genome-wide using larval zebrafish as a model system. We made use of a spatial gene expression atlas to investigate the expression of circadian genes in various tissues and cell types. Comparison of genome-wide circadian gene expression data between zebrafish and mouse revealed a nearly anti-phase relationship and allowed us to detect novel evolutionarily conserved circadian genes in vertebrates. We identified three groups of zebrafish genes with distinct responses to light entrainment: fast light-induced genes, slow light-induced genes, and dark-induced genes. Our computational analysis of the circadian gene regulatory network revealed several transcription factors (TFs) involved in diverse aspects of circadian physiology through transcriptional cascade. Of these, microphthalmia-associated transcription factor a (*mitfa*), a dark-induced TF, mediates a circadian rhythm of melanin synthesis, which may be involved in zebrafish's adaptation to daily light cycling. Our study describes a systematic method to discover previously unidentified TFs involved in circadian physiology in complex organisms.

## Author Summary

For most animals whose lives are dependent on the sun, circadian clocks govern their daily behaviors and physiology. In different animals, novel functions under the circadian clock's control can evolve as adaptations to their specific environment. A zebrafish demonstrates a remarkably high level of interplay between external light and its internal circadian clock due to its transparent nature. In a genome-wide study, we identified a large number of circadian oscillating genes as well as genes whose expression is highly sensitive to the light or dark in zebrafish. Our computational analysis of gene regulatory networks revealed a number of transcription factors (TFs) that mediate novel circadian functions. We investigated one example in depth, a key TF that relays the control of the circadian clock to the enzymes synthesizing melanin in a dark-induced pathway thus causing the daily change of pigmentation in zebrafish. This dark-induced circadian melanogenesis can lead to an anticipatory change in zebrafish skin color allowing zebrafish to adapt to its environment. This mechanism allows zebrafish to better evade predators and effectively adjust its daily light-sensitivity in the pigment cells. Our study provides an excellent example of how the circadian clock is adapted in a specific organism to control its behavior, thus enabling evolutionary adaptation to the organism's ecological niche.

## Introduction

The circadian rhythm evolved as an adaptation to the Earth's daily light cycle. In complex animals, each tissue or cell type contains a functional clock that is controlled by the central pacemaker. In mammals, the suprachiasmatic nucleus (SCN) in the hypothalamus acts as the central circadian pacemaker that coordinates most aspects of behavior and physiology [1]. In lower vertebrates, circadian pacemakers are also present in the eyes and the pineal gland [2]. It is not surprising that circadian rhythm controls physiological processes universal to all tissues such as metabolism [3] and cell cycle [4]. However, tissue-specific functions are also controlled by the circadian clock. Meta-analysis of all existing mammalian circadian gene expression studies indicates that there are thousands of genes showing circadian expression in a given tissue, although only a small number of circadian genes are expressed in more than one tissue [5].

This raises the question as to how genome-wide tissue-specific circadian gene expression is regulated? A simple answer is that all circadian gene expression is entirely regulated by core circadian genes. However, there are only a few transcription factors (TFs) within the core circadian circuit, including *Arntl*/*Clock* (*Bmal1*/*Clock*) and *Bhlhe40*/*41* (*Dec1*/*2*) (binding the E-BOX), *Rora*/*b*/*c* and *Nr1d1*/*2* (*Rev-erb α*/*β*) (binding RRE), and *Dbp*/*Hlf*/*Tef* and *Nfil3* (binding the D-BOX), each of which either activates or represses target genes in mammals. Recent ChIP-seq experiments have revealed 2,049 genome-wide binding sites for *Bmal1* in mouse liver targeting only 16% of the circadian oscillating genes that are expressed in liver and are represented in the circadian gene database [5], [6]. From this we can conclude that, circadian oscillations in gene expression in different tissues are unlikely to all be under the direct regulation of known core circadian TFs, but rather they are likely to be regulated by other TFs that are themselves regulated by core circadian genes [7], [8]. In particular, tissue-specific circadian functions might be regulated by tissue-specific TFs relaying signals from core circadian genes.

Typically, circadian gene expression studies are conducted in a few selected tissues at a time. In mouse, the most studied circadian model in vertebrates, genome-wide circadian gene expression has been studied in only 14 tissues [5]. An additional difficulty is that many tissues or cell types, with potential importance to circadian rhythm are difficult to access without specialized techniques. For example, in Drosophila, Kula-Eversole et al. have studied circadian gene expression in PDF-expressing ventral lateral neurons: s-LNvs and l-LNvs and observed marked differences with those in fly head [9]. In this study, we attempt to circumvent these difficulties by a genome wide analysis of circadian gene expression in whole larval zebrafish (*Danio rerio*). The zebrafish larva is an established model for the study of animal development [10]. Decades of gene expression studies in zebrafish, most of which used *in situ* hybridization (ISH) provide a rich resource of spatial information on gene expression (http://zfin.org). Therefore, circadian oscillating genes identified in this study can be mapped to specific tissues and cell types using a spatial expression atlas.

We have identified a number of tissue-specific TFs that were not previously known to be involved in circadian rhythm. Through the analysis of gene regulatory networks, we identified a novel circadian TF, microphthalmia-associated transcription factor a (*mitfa*), controlling circadian melanin synthesis in pigment cells. *mitfa* shows strong circadian rhythm in expression and is dark-induced. This observation lead us to hypothesize that melanin synthesis itself is similarly circadian controlled. We confirmed this experimentally and speculate that a dark-adaptation pathway is involved. Circadian- regulated melanin synthesis is likely to be crucial to species, including fish and reptiles, that change skin color to evade predators in response to the daily light-cycle. Our study highlights the possibility that different organisms may employ the circadian rhythm differently to control their specific behavior allowing adaptation to their ecological niche.

## Results

### Larval zebrafish exhibit global circadian gene expression

We monitored the circadian behavior of larval zebrafish under an infrared behavioral monitoring platform (Text S1, Figure S1A). Wild-type (WT) larval zebrafish were raised in 14 h∶10 h light/dark (LD) cycle from birth. Starting at 5 days post-fertilization (dpf), they were monitored under either LD or constant dark (DD) conditions. In both conditions, the fish demonstrated robust circadian changes in their locomotor activities (Figure S1B) implying that circadian activities have been fully established at this stage of zebrafish development. The amplitude of oscillation was significantly lower in DD larvae compared to that in LD larvae indicating strong dependence of circadian activities on light entrainment. We subsequently collected larval zebrafish starting at 5 dpf every 4 hours for a period of 48 hours under both LD and DD conditions. The whole-genome transcriptome profiles of the animals were assayed on Agilent zebrafish microarrays.

**Figure 1:**
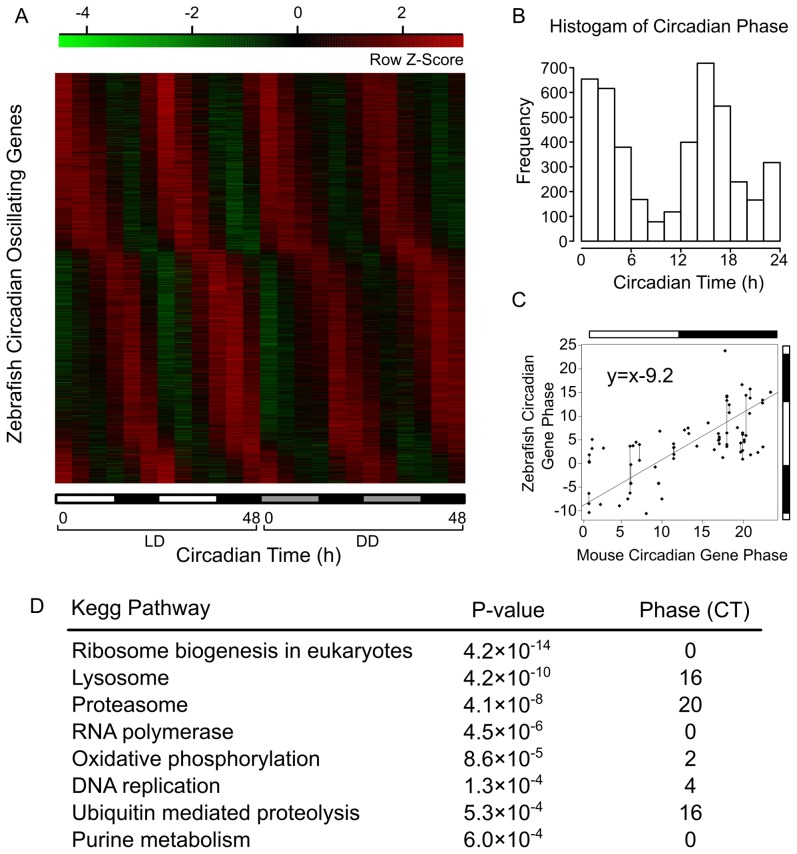
Global circadian gene expression in larval zebrafish. (A) Circadian expression of ZCOGs under both LD and DD conditions. High expression is indicated in red and low expression in green. The bar on the x-axis indicates light (white) and dark (black) in LD, and subjective day (gray) and subjective night (black) in DD. (B) Bimodal distribution of circadian phases of ZCOGs. (C) Circadian phases of homologous circadian genes between zebrafish and mouse. The mouse circadian genes are common circadian genes oscillating in at least six tissues from our previously constructed circadian gene databases [5]. Their circadian phases are averaged across these tissues. The regression line reflects the approximately 9-hour phase shift between zebrafish and mouse circadian genes. Duplicated genes in zebrafish are connected with vertical lines. The vertical bar indicates the zebrafish light/dark cycle (14 h∶10 h) and the horizontal bar indicates the mouse light/dark cycle (12 h∶12 h). (D) KEGG pathways enriched in specific time windows among ZCOGs.

Circadian oscillating genes were identified using Fisher's g test, and their circadian phases were determined by fitting to cosine functions with shifting phases (Materials and Methods). We identified 2,856 circadian oscillating genes in both LD and DD conditions with an overall False Discovery Rate (FDR)≤5% (Table S1). These account for over 17% of expressed genes in larval zebrafish (Figure 1A). We thus refer to these zebrafish circadian oscillating genes in both LD and DD as “zebrafish circadian oscillating genes” (ZCOGs). The mean circadian phases under LD and DD are used to represent the circadian phases of ZCOGs. This dataset displays a prominent bimodal distribution with peaks at CT2 (Circadian Time 2; CT0 is lights-on and CT14 is lights-off) and CT16 corresponding to 2 hours after lights-on and lights-off respectively (Figure 1B).

We annotated the biological functions of ZCOGs using the Gene Ontology (GO) and KEGG databases. ZCOGs are enriched in GO terms related to light and abiotic stimuli and transporter functions (Table S2). As expected, circadian rhythm is the most significantly enriched KEGG pathway among ZCOGs. We then used a sliding window approach to identify the specific time window when the biological functions of ZCOGs are enriched (Figure 1D). Components of the lysosome are enriched around CT16, proteasome components are enriched around CT20, and those involved in ribosome biogenesis are enriched around CT0. We compiled a spatial gene expression atlas from annotations in the ZFIN database, which contains expression information for 96,189 records encompassing 6,429 genes in 1,173 tissues or cell types in larval zebrafish. A total of 1,310 ZCOGs were mapped to the tissues or cell types of expression and we computed the enrichment of circadian oscillating genes in these tissues. We found that ZCOGs are most enriched in retina photoreceptive layer, intestine bulb, and retina.

Zebrafish homologs of almost all known mammalian core circadian genes are represented among ZCOGs (Table 1), indicating that the basic circadian circuit is highly conserved between zebrafish and mammals. To search for novel circadian genes conserved between mammals and zebrafish, we obtained an extended list of mouse circadian genes oscillating in at least six tissues from our previously constructed circadian gene databases [5]. The circadian phases of these genes have very low variability across different mouse tissues. We identified 74 ZCOGs that are homologs of 48 mouse circadian genes (Table S3). Many circadian genes in zebrafish have undergone gene duplications including *arntl1* (*bmal1*), *clock*, and *per1*. Duplicated circadian genes tend to have similar circadian phases. In addition to commonly known core circadian clock genes, heat/cold shock proteins, purine nucleoside phosphorylases, and ubiquitins are also among the conserved circadian oscillating genes between mouse and zebrafish. Interestingly, we observed a nearly anti-phase (9 hour shift) relationship between zebrafish and mouse among conserved circadian genes (Figure 1C). Such phase shift could arise from different entrainment conditions between zebrafish and mouse (14∶10 LD for zebrafish and 12∶12 LD for mouse) and other species differences such as activity patterns.

**Table 1:** Comparison of circadian genes between mouse and zebrafish.

Mouse Gene Symbol	Zebrafish Gene Symbol	Mouse Gene phase	Zebrafish Gene phase
Arntl	arntl1b	22.37	12.79
Arntl	arntl1a	22.37	13.42
Clock	clock	23.35	15.08
Clock	clock3	23.35	15.08
Cry1	cry-dash	17.98	3.58
Cry1	cry1a	17.98	Not present
Cry1	cry2a	17.98	13.33
Cry1	cry4	17.98	14.29
Cry1	cry1b	17.98	6.46
Cry1	cry2b	17.98	14.08
Cry1	cry5	17.98	4.00
Cry2	cry-dash	12.23	3.58
Cry2	cry3	12.23	2.17
Cry2	cry4	12.23	14.29
Cry2	cry5	12.23	4.00
Dbp	dbpb	9.18	23.25
Dbp	dbpa	9.18	Not present
Hlf	hlfb	13.21	14.25
Hlf	hlfa	13.21	Not oscillating
Nfil3	nfil3-2	20.33	Not oscillating
Nfil3	nfil3-6	20.33	5.92
Nfil3	nfil3-4	20.33	Not oscillating
Nfil3	nfil3	20.33	14.42
Nr1d1	nr1d4b	6.19	19.75
Nr1d1	nr1d1	6.19	23.75
Nr1d1	nr1d4a	6.19	19.83
Nr1d2	nr1d4b	9.60	19.75
Nr1d2	nr1d2b	9.60	Not present
Nr1d2	nr1d4a	9.60	19.83
Nr1d2	nr1d2a	9.60	Not oscillating
Per1	per1b	11.44	3.33
Per1	per1a	11.44	1.04
Per2	per2	13.22	7.50
Per3	per3	11.44	4.17
Rora	roraa	11.65	Not oscillating
Rora	rorab	11.65	7.50
Rorb	rorb	9.67	Not oscillating
Rorc	rorca	18.27	10.75
Rorc	rorcb	18.27	12.42
Tef	tefa	11.48	3.42
Tef	tefb	11.48	1.92

### Light entrainment influences circadian gene expression

In larval zebrafish, the amplitude of circadian activity is significantly reduced under DD compared to that under LD conditions. Using the same false discovery rate (FDR) parameters, the number of circadian oscillating genes under DD is also significantly lower than that under LD. To examine the effects of light entrainment on circadian gene expression, we first compared the phases and amplitudes of circadian oscillations of ZCOGs between DD and LD conditions. Among 2,856 ZCOGs, the circadian phases are highly consistent between DD and LD (circular correlation coefficient r = 0.93) with only 169 (6%) ZCOGs showing phase shifts of more than four hours. In contrast, 233 ZCOGs oscillate at reduced amplitudes with only 21 ZCOGs oscillating at increased amplitudes under DD compared to LD. Thus light entrainment tends to enhance the amplitudes of circadian oscillation of gene expression.

**Figure 2:**
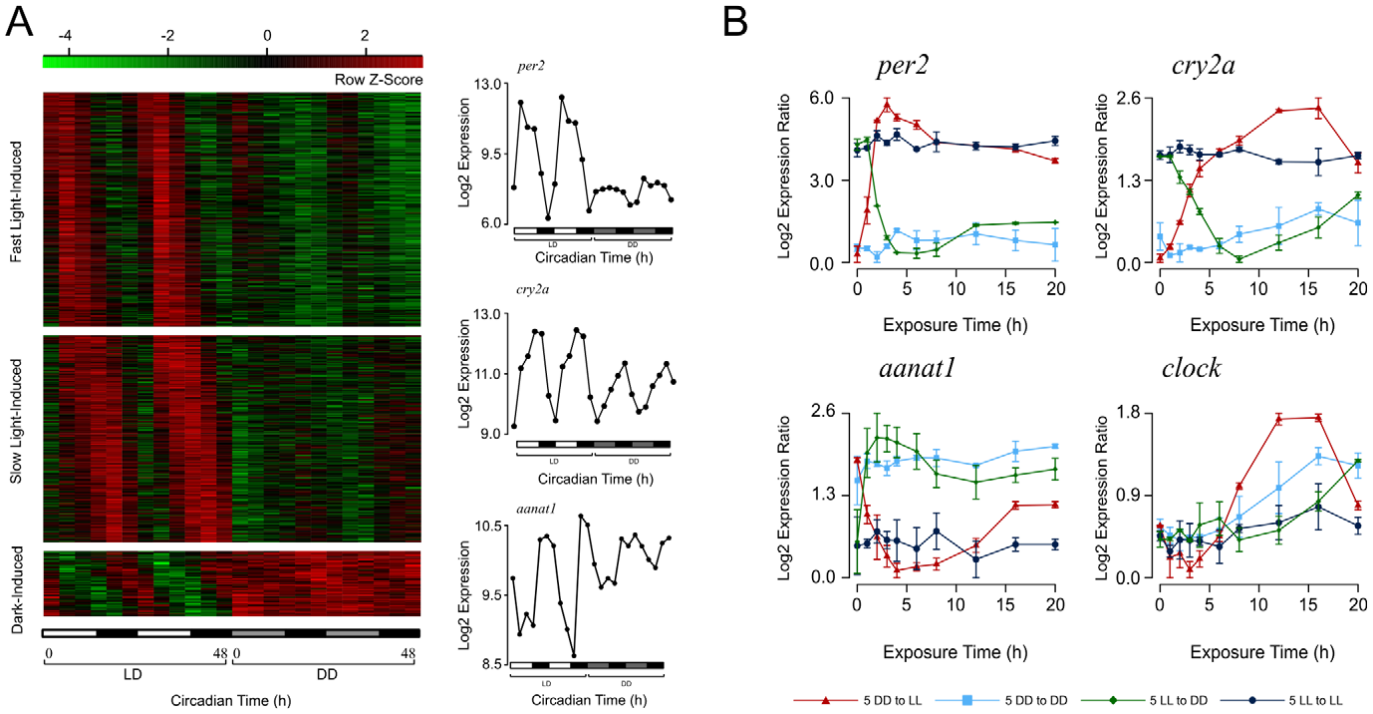
Three types of genes affected by light entrainment. (A) The circadian expression of fast light-induced genes, slow light-induced genes, and dark-induced genes and representatives of each group (*per2*, *cry2a*, and *aanat1*) on the microarrays are shown. (B) The distinct kinetics of *per2*, *cry2a*, *aanat1*, and *clock* genes upon light or dark exposure in larval zebrafish fertilized and raised under either DD or LL conditions respectively for 20 hours starting at 5 dpf (5DD to LL and 5LL to DD). The larval zebrafish remained in the constant conditions (5DD to DD and 5LL to LL) were used as controls. The lowest log2-transformed expression level for each gene was normalized to zero. Two independent time-series experiments were conducted with error bars shown in the figure.

Next, we searched for genes having reduced amplitudes as a consequence of either decreased peak levels or elevated trough levels in DD compared to LD. To include the genes that might have lost rhythmicity under DD, we examined 3,677 LD oscillating genes (FDR≤10%) regardless of their rhythmicity under DD. Among them, we identified 464 genes showing decreased peaks and 113 genes showing elevated troughs in DD compared to LD (Materials and Methods). We defined 366 peak decreased genes with LD circadian phases lying in the light period as “light-induced” because their expression increases in the light period in LD but remains at low level in DD (Figure 2A; Table S4). Similarly, 50 trough-increased genes with LD circadian phases lying in the dark period were defined as “dark-induced genes”. Furthermore, we observed that the LD circadian phases of light-induced genes were predominantly distributed around CT5. The expression of these genes increases rapidly after lights-on and reaches a peak after 5 hours in LD. Thus we defined 197 light-induced genes with LD circadian phases between CT2 and CT8 as “fast light-induced genes”. This group includes known light-sensitive genes (e.g. *per2*, *cry5*, *cry-dash*, and *tefa*). It also contains genes showing significantly enriched expression in epiphysis and the retinal photoreceptive layer (e.g. arrestin 3 (*arr3a*/*b*) and interphotoreceptor retinoid-binding protein (*irbp*)). The remaining light-induced genes show a slow increase in expression during the light period, reaching a peak before lights-off in LD. Accordingly, they were defined as “slow light-induced genes”. This group includes key circadian genes (e.g. *cry2a*/*b*, *arntl1b*, and *nfil3-5*). The expression of dark-induced genes is repressed during the light period but increases in the dark period reaching peak level after lights-off. Among the dark-induced genes are melatonin synthesizing enzyme (*aanat1*), dopamine D4 receptor (*drd4a*) and two genes related to pigmentation (*mitfa* and *slc24a5*). In short, there are complex, dynamic responses to light entrainment during circadian gene expression.

In another set of experiments, we collected larval zebrafish fertilized and raised under either DD or LL (constant light) conditions and exposed them to light or dark, respectively for 20 hours starting at 5 dpf. The expression of one representative gene from each of the previously described groups was measured by quantitative real-time PCR (Text S1): *per2* from the fast light-induced genes, *cry2a* from the slow light-induced genes, *aanat1* from the dark-induced genes and the *clock* gene (Figure 2B, primers used for real-time PCR are in Table S5). The first few hours after light/dark exposure were sampled more frequently. Without light/dark exposure, the circadian clock was desynchronized in animals kept in DD or LL conditions [11], [12]. The expression levels of *per2* and *cry2a* are significantly higher in LL than DD and *aanat1* was expressed at a significantly lower level in LL than DD whereas the expression level of the *clock* gene showed no significant difference between LL and DD. After light exposure, the expression of *per2* increases rapidly and reaches a peak after 3 hours whereas, after dark exposure, it decreases rapidly within 3 hours. In contrast, *cry2a* expression increases slowly after light exposure and decreases slowly after dark exposure only reaching its peak or trough about 12 hours after lights-on or lights-off, respectively. The expression of *aanat1* increases quickly upon dark exposure and drops just as rapidly after light exposure. The expression of *clock* shows a slow increase of expression reaching its peak around 12 hours after light exposure and an even later increase around 20 hours after dark exposure. These results indicate that the endogenous circadian clock has been resynchronized after the exposures and may regulate the induction and repression of slow light-induced genes and dark-induced genes.

### A transcriptional cascade regulates diverse circadian functions

To understand what drives the circadian oscillation of ZCOGs, we developed a comparative genomics pipeline to predict TF binding sites for zebrafish genes. Our pipeline uses orthologous promoter sequences from teleost fish genomes, including zebrafish, fugu, medaka, stickleback, and Tetraodon (Materials and Methods). Our ZCOGs consist of 140 TFs, including novel circadian TFs in addition to the known core circadian TFs (Table S6). We focused on the 54 circadian TFs with known DNA-binding motifs in the TRANSFAC database [13] for promoter analysis.

**Figure 3:**
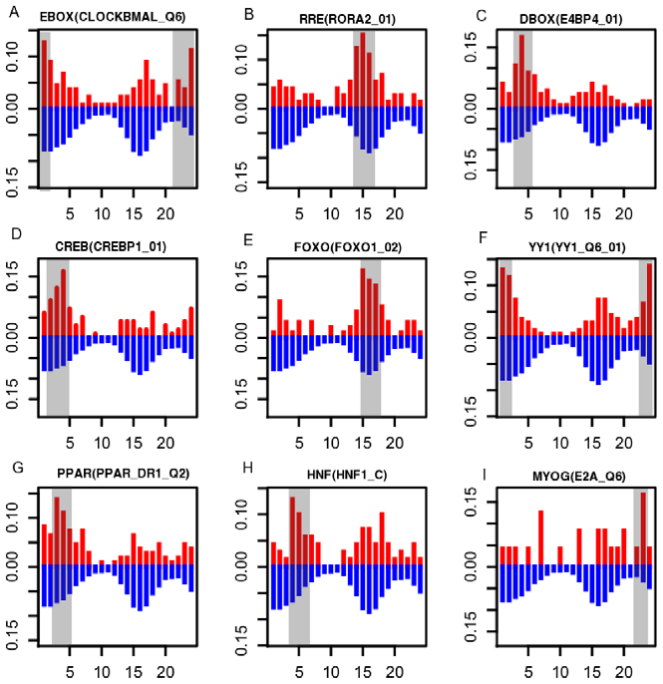
The circadian phase distributions of predicted targets of nine circadian TF motifs. The percentage of predicted targets within a certain phase is plotted against the circadian phase between 0 and 24 hours. The red bars indicate phase distributions of targets of circadian TFs, whereas the blue bars indicate the phase distribution of all ZCOGs as the background. Gray boxes show the enriched time windows of the targets (p<0.001). The matrix IDs corresponding to TFs were indicated in the parentheses (Table S7).

Because the circadian phases of ZCOGs show a bimodal distribution (Figure 1B), we divided ZCOGs into two groups, the first with peak times within CT22-CT10, and the second with peak times within CT10-CT22. In the first group, we identified E-BOX as the most enriched promoter motif (p value = 2.5×10^−10^, odds ratio = 2.3), while RRE is significantly enriched in promoters from the second group of genes (p value = 1.3×10^−5^, odds ratio = 2.0). This is consistent with observations in mouse except that the enriched phases are also shifted by about 10 hours following the same phase-shift of homologous TFs in zebrafish and mouse [5]. We then used a sliding window approach to identify the specific time window when the circadian phases of circadian TF targets are enriched (Table S7). In addition to E-BOX and RRE, we also observed that targets of *ppargc1b*, *yy1a*/*b*, *atf*/*creb*, *hnf1a*, *foxo3b*, and *myog* were enriched at a specific time window (p<0.001, Figure 3). There are varying delays between phases of TFs and enriched phases of their targets. Phases of *tef*, *foxo3b*, *ppargc1b*, and *hnf1a* are close to their targets, while *arntl*/*clock*, *nfil3*, *yy1a*, and *myog* are nearly anti-phase to their targets.

To assist sequence-based TF-target prediction, we generated co-expression groups of zebrafish genes using zebrafish microarray data available in the Gene Expression Omnibus (GEO) database (Materials and Methods) and mapped ZCOGs to these groups. By making use of the fact that co-expression often indicates co-regulation by TFs, we were able to identify the TF motifs enriched in the promoters of the co-expression groups and their associated circadian TFs (Table S8). A group consisting of mostly mitochondrial genes is enriched with ZCOGs and their circadian phases are around CT0. The genes in this group are predicted to be regulated by *yy1a*. Consistent with our GO analysis, proteasome components form a co-expression group having circadian phases around CT18, although no known TF motifs are enriched in the group. Other co-expression groups regulated by circadian TFs include heat shock proteins regulated by *hsf2*, liver-specific genes regulated by *hnf1a*, and genes in visual processing regulated by *crx* (core-rod homeobox).

*Hnf1a* is known to be a liver-specific TF [14] and expression of *crx* is restricted to retinal photoreceptor cells and the epiphysis [15], [16]. Using the spatial gene expression atlas in zebrafish, we searched for tissue-specific TFs among ZCOGs by requiring that the predicted targets of the TFs are predominantly expressed in the same tissue as the TFs themselves. We systematically identified 11 tissue-specific circadian TFs. They include several well-known tissue-specific TFs: *mitfa* in pigment cells [17], *cdx1b* in the intestine [18], *ppargc1b* in the liver, *mef2a* in heart and muscle tissue [19], *pax6a* in nerve tissues [20], *smad1* [21], *smad3a* [22], and *myog* [23] in muscle tissue. A less well-known tissue-specific circadian TF, *maf*, is expressed in the lens vesicle [24]. There is evidence that *maf* plays an important role in lens development and in lens fiber cells in mice [25]. Interestingly, two key circadian TFs appear to be tissue-specific: *nr1d1* and *rorab* are expressed in epiphysis and retina, while their predicted targets show enriched expression in the same tissues. Notably, *nr1d1* plays an important role in photoreceptor development and function [26].

**Figure 4:**
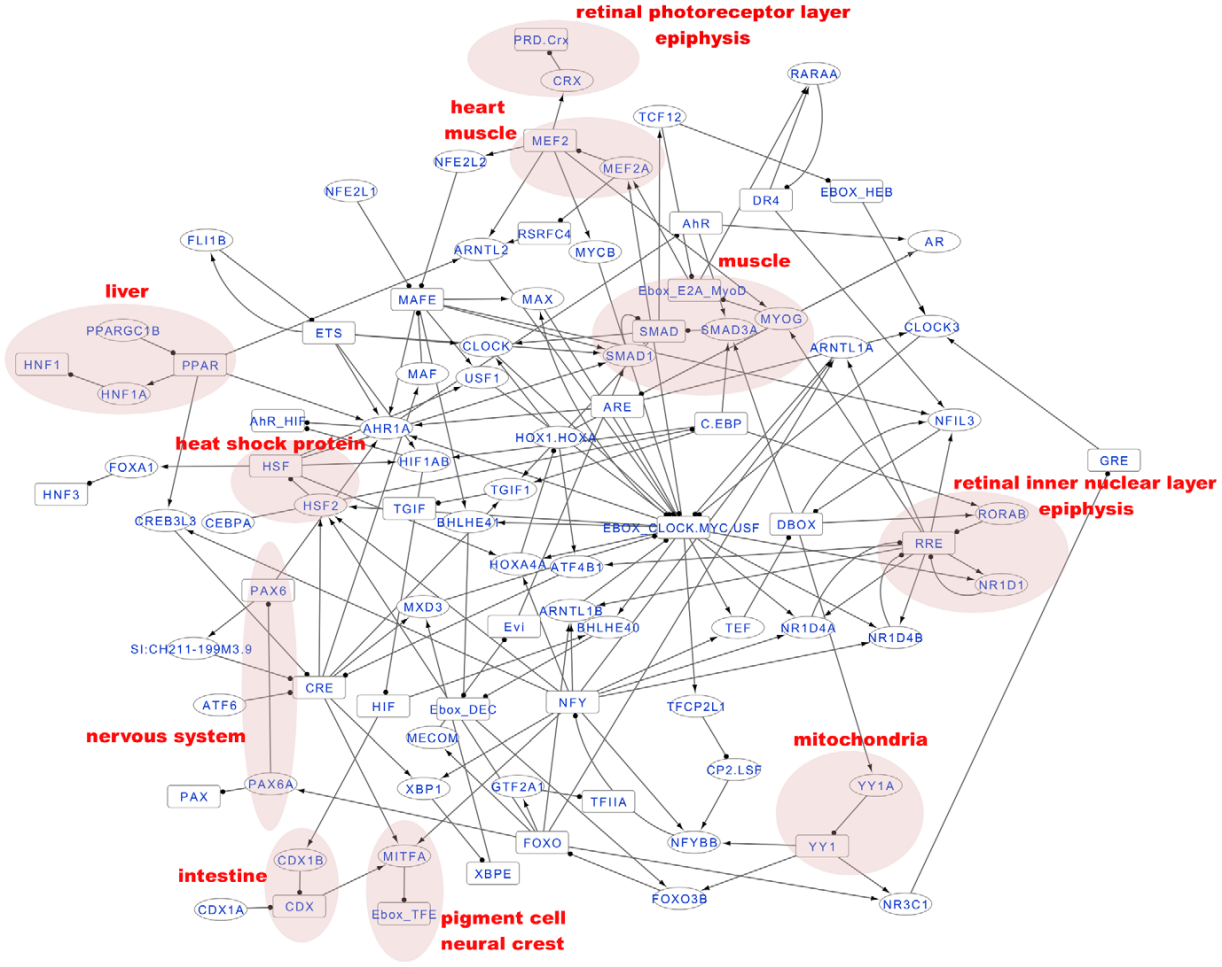
Gene regulatory network of circadian oscillating TFs and their motifs. Predicted regulatory events between all circadian oscillating TFs are connected by TF-motif and motif-target relationships. Circles represent circadian oscillating TFs and boxes represent *cis*-regulatory elements characterized by TF DNA-binding motifs. Arrowed edges represent motif-target relationships and circle-capped edges represent TF-motif relationships. The shaded areas represent the tissues or cell types where specific motifs are enriched.

From this data we constructed a gene regulatory network of circadian TFs to depict how they regulate each other and how circadian oscillating genes in specific tissues are regulated (Figure 4). In this network, circadian-oscillating TFs were grouped by TF-motif and motif-target relationships, where TF-motif relationships were obtained from the TRANSFAC database, while motif-target relationships were predicted through promoter analysis in our comparative genomics pipeline (see Materials and Methods). This approach successfully recapitulated the known core transcriptional feedback loop in circadian clock regulation: E-BOX binding TFs *arntl*/*clock* regulate *nr1d1* and *rorab*, which in turn regulate *arntl* through RRE. Most circadian TFs are directly regulated by the circadian clock via known circadian *cis*-elements. For example, *mef2a*, *hsf2*, *tef*, *bhlhe40*/*41* (*dec1*/*2*), *tfcp2l1*, *foxo3b*, and *ahr1a* via E-BOX, *myog*, *nfil3*, *atf4b1* via RRE and *yy1a* and *smad3a* via D-BOX. The remaining TFs are indirect targets of core circadian TFs via further transcriptional cascades. For example, *maf* and *mitfa* are regulated via cAMP responsive element (CRE), *hnf1a* and *creb3l3* via the *cis*-element of the PPAR family TFs and *pax6a* and *nr3c1* via the *cis*-element of *foxo3b*.

**Figure 5:**
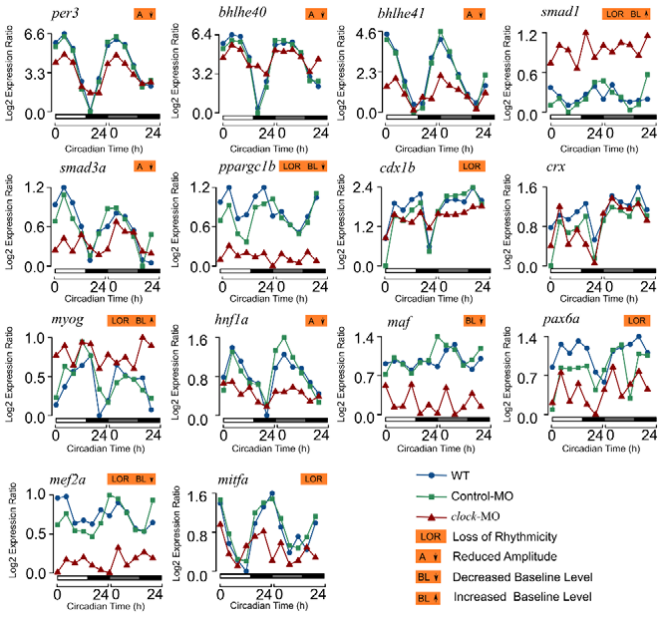
Circadian expression profiles of 14 tissue-specific TFs. The expression levels of *bhlhe40*/*41* and *per3* together with 11 circadian tissue-specific TFs were measured by real-time PCR in 5 dpf *clock* morphants (red) compared to WT (blue) or control (green) morphants. The lowest log2-transformed expression level for each TF was normalized to zero. The reduction in oscillation amplitude, loss of rhythmicity, and increase or decrease in baseline expression levels in *clock* morphants compared to WT or control morphants were annotated for each gene.

Our network analysis shows that *clock* is at the center of the regulatory network and all other circadian TFs are under its control either directly or indirectly. In order to validate this experimentally, we generated *clock* morpholino (MO) knock-down larvae. We measured circadian mRNA levels of *bhlhe40*/*41* and *per3* together with 11 circadian tissue-specific TFs by real-time PCR in 5 dpf *clock* morphants compared to WT or control morphants (Figure 5). In WT and controls, the circadian peak times of these genes show high consistency between real-time PCR and arrays. In *clock* morphants, *bhlhe40/41*, *per3*, *smad3a*, and *hnf1a* continue to oscillate but show significant reductions in oscillation amplitudes. The remaining genes show loss of rhythmicity except for *crx* in *clock* morphants. Furthermore, *smad1* and *myog* show significant up-regulation (p<0.001), whereas *ppargc1b*, *maf*, *mef2a* show significant down-regulation in baseline levels in *clock* morphants compared to the WT and controls (Primers used for real-time PCR are in Table S5).

### Circadian control of melanogenesis is mediated by *mitfa*

In order to show that TF-mediated circadian control can have novel functional consequences, we focused on *mitfa*, one of our identified circadian TFs and the most enriched TF in the pigment cells. It is also a dark-induced gene. The mammalian homolog of *mitfa* is *Mitf*, which is a key TF controlling melanogenesis in mammals [27]. In zebrafish, *mitfa* is specifically expressed in pigment cells and retinal pigment epithelium (RPE) [28]. Notably, *mitfb*, a gene duplicate of *mitfa*, does not show circadian rhythm in our microarray result. To validate the function of *mitfa* in zebrafish, we generated *mitfa* MO knock-down zebrafish larvae. The *mitfa* morphants lost melanin production in melanocytes of the fish trunk and had smaller eyes compared to WT and control morphants (Figure S2). It has been reported that *Mitf* directly regulates *Tyr*, *Tyrp1*, and *Dct*, genes encoding three key enzymes involved in melanin synthesis, in addition to other genes involved in melanogenesis in mouse and human [29], [30]. In our study, *tyrp1b* (the zebrafish homolog of *Tyrp1*), *slc24a5* (another gene known to be involved in melanogenesis) and *dct* all showed circadian peaks around CT20 in LD, reduced oscillation amplitudes and elevated troughs in DD, similar to *mitfa* (Figure S3). We then measured the mRNA levels of these genes in *mitfa* morphants by real-time PCR. All showed reduced expression in *mitf*a morphants compared to WT and control morphants, suggesting that they are also *mitfa* targets in zebrafish.

**Figure 6:**
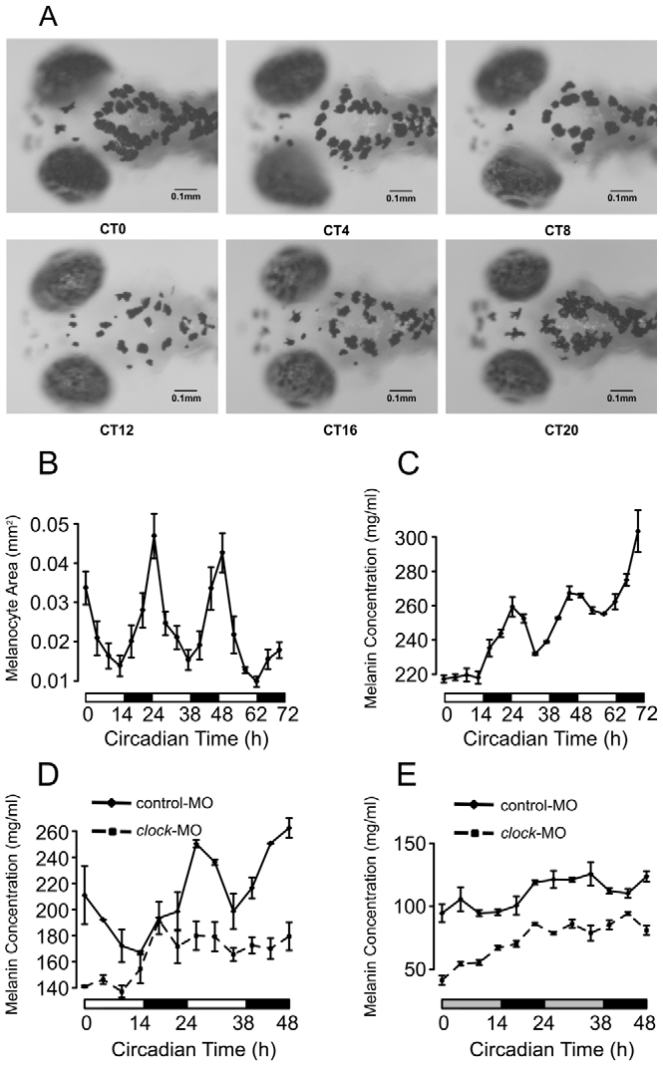
The circadian rhythm of melanogenesis in larval zebrafish. (A) Images of 5 dpf WT larval melanocytes in 4 hour intervals over 24 hours under LD conditions. (B) The area of melanocytes in WT larvae showed robust circadian rhythm in LD starting at 4 dpf (p<0.001, Fisher's g test). (C) Melanin concentrations of WT larvae showed robust circadian rhythm in LD while increasing with time (p<0.002, Fisher's g test after detrend). (D) The rhythm of melanin concentration was abolished in *clock* morphants in LD conditions, while the rhythm in control morphants remained robust. (E) Under DD conditions, the circadian rhythms of melanin concentrations in *clock* morphants and control morphants were not significant.

We reasoned that the circadian oscillation of *mitfa* expression implies that melanin synthesis also oscillates on a daily basis. To experimentally demonstrate the circadian oscillation of melanin synthesis, we first measured the areas of melanocytes in the head regions of larval zebrafish under LD beginning at 4 dpf (Figure 6A, Materials and Methods) and found a significant circadian rhythm in pigmentation with a peak around CT0 (p<0.001, Fisher's g test, Figure 6B). Then we quantified total melanin concentration using a melanin assay in whole larval zebrafish. The melanin concentration showed a strong circadian rhythm with a circadian phase also around CT0 in LD (p<0.002, Fisher's g test after detrend, Figure 6C) starting at 4 dpf, while the absolute amount of melanin increased during development. To determine the effect of *clock* on pigment synthesis, we examined the melanin concentration rhythm in *clock* morphants. The melanin concentration showed a significantly reduced circadian rhythm in LD compared to control morphants (Figure 6D). Melanin oscillations were not significant in DD in either *clock* morphants or controls (Figure 6E). Similarly, the amplitude of *mitfa* expression was significantly reduced by over 30% in *clock* morphants in LD compared to WT and controls, and oscillations are nearly absent in DD. From this we conclude that the circadian control of melanogenesis is mediated by *mitfa* in zebrafish larvae.

The melanogenesis signaling pathway has been well-characterized in mammals. Alpha-melanocyte stimulating hormone (α-MSH) activates melanocortin receptor in pigment cells leading to the up-regulation of *Mitf* through cAMP signaling pathway [31]. Consistent with this, we identified a conserved CRE in the promoter of *mitfa* in zebrafish (Figure S4). In mammals corticotropin releasing hormone (CRH) secreted from the paraventricular nucleus (PVN) of the hypothalamus affects the secretion of α-MSH in the pituitary gland thus forming a hypothalamus-pituitary-melanocyte (HPM) axis. In our study, the mRNA level of *crh* shows circadian oscillation at CT9 in LD and reduced amplitude in DD. To examine if the HPM axis controls the expression of *mitfa* in zebrafish, we treated zebrafish larvae with the Crh receptor 1 antagonist antalarmin. We observed significant reduction of *mitfa* expression upon antalarmin treatment at CT0 (Text S1, Figure S5). Thus, it appears as though the circadian oscillation of *mitfa* may be regulated by the *crh* signaling cascade through the HPM axis.

The *mitfa* gene is involved in the early development of the neural crest from which pigment cells are derived [32]. Multiple neural cell types are also derived from the neural crest cells and *mitfa*'s control of melanogenesis is likely to be only one aspect of its function. To investigate the broader regulatory functions of *mitfa* in zebrafish early development, we collected *mitfa* morphants together with WT and control morphants at 48 hpf (hours post-fertilization) and 50 hpf for microarray experiments (Text S1). We identified 555 down-regulated genes and 691 up-regulated genes in *mitfa* morphants, compared to WT and control larvae (Table S9). In addition to *tyr*, *tyrp1b*, *dct*, and *slc24a5*, we also identified *tcf7l2*, *lef1*, *camk2d2*, and *slc45a2* which are also involved in melanogenesis, as being down-regulated in *mitfa* morphants. Mapping of the down-regulated genes onto the spatial gene expression atlas revealed that the retina is the most common tissue for their expression. This is consistent with the small eye phenotype of *mitfa* morphants. Genes involved in brain development and the *wnt* signaling pathway are enriched in the down-regulated genes. Of the *mitfa*-affected genes represented by ZCOGs, we found 87 ZCOGs down-regulated and 213 ZCOGs up-regulated in the *mitfa* knock-down. Among these, two TFs, *six3a* and *vsx1*, are involved in retina development and both show circadian peaks at CT16. We conclude that, in addition to mediating circadian melanogenesis, *mitfa* is also likely to mediate circadian control of early development in other tissues such as the retina.

## Discussion

Until now, studies of circadian rhythm have been limited to a handful of organisms such as mouse, fruitfly, Neurospora [33], cyanobacteria [34], and Arabidopsis [35], species with large evolutionary distances separating them. Mouse is the only vertebrate species in which the circadian rhythm has been extensively studied. However, other vertebrate model systems have been largely unexplored. In recent years, zebrafish has become a model organism to study the vertebrate circadian rhythm, including at the molecular level [36], [37]. Our results suggest that the molecular mechanism of the zebrafish circadian rhythm has many characteristics in common with the mammalian system. Following the duplication of many core circadian genes in zebrafish, the relative phase relationship of the duplicates has been largely conserved. Our genome-wide comparison of circadian gene expression between zebrafish and mouse identified novel evolutionarily conserved circadian genes in vertebrates that deserve further functional studies in both species.

The transparent nature of larval zebrafish has also made it an excellent system to study light entrainment of the circadian rhythm. Our study shows that the amplitudes of many circadian oscillating genes are significantly reduced in DD compared to LD but their phases are still similar between LD and DD. Comparing gene expression under LD and DD conditions, we discovered that circadian gene expression was induced by light in a progressive manner. The fast light-induced genes showed the most dramatic up-regulation, within eight hours of light-onset. In comparison, expression of the slow light-induced genes rose slowly and reached their peak only near the time of light-off. The transcripts of slow light-induced genes may have longer degradation times than those of fast light-induced genes [6]. Gavriouchkina et al. (2010) identified a set of light-induced genes in zebrafish from a light exposure experiment in early embryos [38]. Among the 19 light-induced genes identified in their experiment, we detected 16 on our microarray. Twelve of these 16 light-induced genes were identified as fast light-induced genes except for *cry2a* and *cry2b* which were identified as slow light-induced genes in our study. The light-exposed samples in Gavriouchkina et al.'s experiment were collected after nine hours of light exposure which is close to the time to reach peak expression after light-onset among our fast light-induced genes. Weger et al. (2011) analyzed light-induced transcriptome change in zebrafish larvae, heart and cell cultures after one and three hours of light exposure [39]. Although very few genes changed their expression after one hour of light exposure, after three hours of light exposure 74 were up-regulated and 24 down-regulated in larvae. Among the up-regulated genes, we identified 32 as being fast light-induced genes and 8 as slow light-induced genes in our study (Table S10). Notably, none of the 24 down-regulated genes in Weger et al's study overlap with the dark-induced genes in our study. The distinct kinetics of light induction has also been observed in Neurospora [7] and Drosophila [40]. In Neurospora, early light response genes peak within minutes and late light response genes peak after 30 minutes or more, response times which are much faster than observed in zebrafish. In our study, *tefa* is a fast light-induced TF binding the D-BOX, whose expression spikes at CT4 under LD. Our promoter analysis also supports the possibility that D-BOX is enriched in the promoters of fast light-induced genes. Among ZCOGs, the targets of D-BOX were enriched around CT5 at which time fast light-induced genes reach their peaks (Figure 2). Thus we conclude that the up-regulation of fast light-induced genes is under the direct control of light via D-BOX. This observation is consistent with the results of Gavriouchkina et al (2010), in which light-induced genes were mostly regulated by *tef*. Vatine et al. (2009) have shown that light entrains circadian rhythm via D-BOX in *per2*'s promoter [41]. In Neurospora, the light responses largely depend on the white collar complex (WCC), which consists of WC-1 and WC-2, via an early light response element (ELRE). D-BOX performs a similar role in fast light-induced genes in zebrafish. Dark-induced genes have been previously observed in both Arabidopsis [42] and cyanobacteria [43]. Fast light-induced genes in zebrafish include core clock genes such as *per2*. Therefore dark-induced genes and slow light-induced genes are likely to be regulated by fast light-induced genes through clock regulatory mechanism. In support of this, the expression of *clock* gene increases during the DD to LL experiment, reaching the peak at CT12 in subjective time. Further studies are needed to separate the direct light-response from the indirect clock regulation. Among the dark-induced genes, *aanat1*, a key enzyme that synthesizes melatonin at night, is known to be suppressed by light in the zebrafish retina [44]. In addition, the mouse homolog of dopamine D4 receptor (*drd4a*) has also been reported to be involved in dark sensing [45]. We also observed that two dark-induced genes, *mitfa* and *slc24a5*, are involved in melanogenesis, which led us to investigate the circadian and photic controls of melanogenesis in more detail.

On considering previous studies of circadian rhythm in mouse [5], [46], we find that our zebrafish network contains homologs of many known mouse circadian TFs. The basic core transcriptional feedback loops are similar in these two networks. However, the zebrafish network includes a number of novel TFs showing circadian rhythm in zebrafish larvae. Among them, only *crx* has been previously associated with circadian functions. It has been proposed that *Crx* and cAMP responsive TFs synergistically activate *Aanat* and *Asmt*, which encode the two enzymes synthesizing melatonin in rat pineal gland [47]. The mRNA level of *Crx* in rat pineal gland showed a circadian peak in the middle of night. In our result, the circadian phase of *crx* (CT17) also occurs in the middle of night and just precedes the circadian phase of *aanat1* (CT20), the zebrafish homolog of *Aanat*. Similar to mouse, zebrafish *crx* is also expressed in retina in addition to epiphysis [15]. *Crx*-deficient mice were affected in circadian entrainment as well as photoreceptor- and pineal-specific gene expression [48]. Among the *Crx*-targeted genes identified in ChIP-seq experiments in the mouse retina [49], we identified 40 of them as having zebrafish homologs exhibiting circadian oscillation, including the retinal arrestin gene and prostaglandin synthases.

The target genes of some circadian TFs tend to peak at a specific time of day. We predicted that *yy1a*, oscillating at CT12, regulates a group of mitochondrial genes oscillating around CT0. Support for this prediction comes from a *Yy1* ChIP-seq assay in human cells that identified *Yy1* targets as being enriched in mitochondrial genes [50]. The coordinated oscillation of genes in the mitochondrial respiratory chain has also been observed with mouse SCN [51]. This was suggested to provide the maximal metabolic output during the active phase of SCN neurons [52]. The time-lag between circadian TF peaks and that of their targets can vary from immediate up to 12 hours. This is likely to reflect differences between TFs with respect to time delays in their translation and the trans-activation of their target *cis*-regulatory elements.

The circadian system in the whole zebrafish larva consists of a collection of peripheral clocks [37], [53]. Although the mRNAs collected from zebrafish larvae are a mixture of transcripts originating from different tissues, we could associate different transcripts with tissues or cell types of expression using the spatial expression atlas in zebrafish. We observed that many circadian TFs show tissue- or cell-type specific expression. We postulated that they are effectors of tissue-specific circadian output from core circadian genes via transcriptional cascades, as implied by the reduction of their amplitude or loss of their rhythmicity in *clock* morphants. This may be the key to explaining why such a wide range of circadian functions can be driven by a handful of core circadian genes. We then focused on *mitfa*, a novel circadian TF governing melanogenesis and found not only that melanogenesis followed a circadian rhythm but that this was mediated by circadian oscillation of *mitfa*.

Interestingly, a similar pathway mediating circadian control of melanogenesis has been elucidated for the circadian control of cortisol synthesis in adrenal gland along the hypothalamus-pituitary-adrenal (HPA) axis in mouse [54]. In the HPA axis, adrenocorticotropic hormone (ACTH) is released by the pituitary gland to stimulate cortisol release in the adrenal gland. In fact, ACTH and α-MSH are generated from the same protein precursor: proopiomelanocortin (POMC) in the pituitary gland. Thus, it is conceivable that the HPM axis governs the circadian synthesis of melanin in zebrafish melanocytes in the same way as the HPA axis governs circadian synthesis of cortisol in the mammalian adrenal gland. The dark-inducing or light-suppressing signals may be first transmitted to the hypothalamus from the retina and then relayed to melanocytes through the HPM axis. However, the circadian system in zebrafish has a very different organization from that of the mouse, as individual peripheral clocks in zebrafish are directly light responsive [53], [55]. Our whole-larva study cannot distinguish the direct impact of light on peripheral clocks from the indirect systemic effects based on light input to specialized photoreceptor tissues such as the pineal complex and deep brain photoreceptors. It is possible that the light signals enter melanocytes and influence the local clock directly. Although melanogenesis is strongly activated by darkness, it is not entirely driven by the light/dark cycle because residual circadian oscillations in *mitfa* and *slc24a5* still persist even under DD conditions. This is perhaps a consequence of the persistent oscillation of cAMP level governed by the local clock. In the zebrafish retina, the concentration of cAMP oscillated in a circadian manner and was regulated by the *clock* gene [56]. Furthermore, the circadian phase of cAMP concentration is in the subjective early morning, close to the peak time of *mitfa*. Another dark-induced gene, *aanat1*, is also regulated by the cAMP signaling pathway [57]. In our study, two adenylate cyclases, *adcy2b* and *adcy8l* (*LOC560410*), showed circadian oscillation at CT16 under DD conditions, indicating that cAMP level is likely to be still oscillating under DD conditions in melanocytes. In combination, local clock and external photic signals may synergistically control melanogenesis mediated by *mitfa*.

Organisms can use circadian rhythms to control their specific physiological behavior in adapting to their own ecological niche. In Neurospora, four genes encoding enzymes in the consecutive steps of the carotenoid biosynthesis pathway, which produces photoprotective pigments, are all early light-induced [7]. In contrast, in zebrafish larvae, melanin synthesis is dark-induced which may lead to skin color adaptation to their environment so that they can better evade predators. This may also help them to adjust their daily light-sensitivity in pigment cells. The melanin biosynthesis pathway regulated by *mitfa* conforms to a network motif common in gene regulatory networks, namely the single-input module (SIM), a master TF controlling a group of target genes [58]. A SIM can generate temporal order of gene expression in metabolic pathways such as arginine biosynthesis in *E.coli* [59]. The wiring of such modules in the circadian pathway may have evolved to generate a daily change of pigmentation in larval zebrafish.

Tissue-specific TFs such as *mitfa* mediate the circadian control of the central circadian clock by receiving inputs from the central clock and relaying signals to multiple targets, thus generating circadian-linked physiological changes such as circadian melanin synthesis. This phenomenon is not unique to zebrafish. In the mouse heart, the circadian cycle controls cardiac arrhythmogenesis through a *Bmal1*/*Clock* targeted TF, kruppel-like factor 15 (*Klf15*) [60]. *Klf15* then regulates the circadian expression of Kv channel-interacting protein 2 (*KChIP2*) thus leading to a daily change of susceptibility to heart arrhythmia. Notably, TF-encoding genes are enriched among *Bmal1* targets in a genome-wide ChIP-seq experiment [6]. Mouse homologs of zebrafish circadian TFs investigated in this study (e.g. *Mef2a*, *Foxo3*, *Ppargc1b*, *Smad1*, and *Maf*) have been identified as *Bmal1* targets in mouse. *Mef2a* in heart, *Smad3* in skeletal muscle, and *Ppargc1b* and *Foxo3* in liver showed strong circadian oscillations in their respective tissues [5]. Their tissue-specific circadian functions merit further investigation. Our results suggest that the transcriptional cascade via TFs from a central clock is a universal mechanism that generates diverse circadian functions in all complex organisms.

## Materials and Methods

### Animals

WT AB strain adult zebrafish (*Danio rerio*) were obtained from the National Zebrafish Resources of China (Shanghai Institutes for Biological Sciences). Fertilized embryos were collected in the morning shortly after lights-on. Zebrafish larvae were maintained in the incubator at 28°C under 14 h∶10 h light/dark cycle from birth. The light was turned on at 9:00 and turned off at 23:00. The luminance during light exposure was determined to be about 1000 lux as measured by a digital luxmeter (Model ZDS-10, SHXL) on the water surface. Zebrafish handling procedures were approved by the Institute of Neuroscience, Shanghai Institutes for Biological Sciences, Chinese Academy of Sciences.

### Sample collection

To examine the circadian gene expression of larval zebrafish, we continuously collected larval samples for microarray analysis starting at 5 dpf in both LD (14 h∶10 h light/dark) and DD (constant dark) conditions, respectively. WT larvae were raised in 14 h∶10 h LD conditions from birth to 4 dpf. At 4 dpf, the larvae were transferred to 24 dishes (35 mm) each containing 40 larvae. Before lights-on at 5 dpf, 12 dishes remained in the LD conditions and the other 12 dishes were placed in the DD conditions. A total of 40 larvae/dish in LD conditions and 40 larvae/dish in DD conditions were sampled simultaneously at 4 h intervals starting at CT0 (CT0 = lights-on at 9:00) of 5 dpf for 12 time points. None of the larvae in the samples was found to have died during this process. The larvae were sucked into freezing tubes, removed of water, frozen immediately in liquid nitrogen, and stored at −80°C. The collection of samples under dark was performed under dim red light.

### Zebrafish microarray

Total RNA of larval sample was extracted using Trizol (Invitrogen) according to the manufacturer's instructions. The quantity and quality of the RNA samples were assessed with a NanoDrop ND-1000 spectrometer (NanoDrop Technologies) and an Agilent 2100 bioanalyzer (Agilent). 12 LD RNA samples and 12 DD RNA samples were used for Agilent whole zebrafish 4x44K microarrays, consisting of 43,603 probes providing a whole-genome transcriptional profile. Purified total RNA of each sample was amplified and labeled with a fluorescent dye Cyanine 3 (Cy3) using a low-RNA input linear amplification kit following the manufacturer's protocol (5184-3523, Agilent). Cy3-labeled cRNA (800 ng each) was hybridized to the zebrafish microarray (G5219F, Agilent) for 17 h at 65°C. The hybridized microarrays were then washed according to the manufacturer's protocol. Microarray results were extracted using Agilent G2565BA Scanner and Feature Extraction software (v10.5.1, Agilent), and subsequently analyzed by Gene-Spring GX software (v11.0.1, Agilent).

### Microarray data analysis

Microarray data were quantile normalized. The probe sets detected in 75% of the samples were kept for downstream analyses. The annotations for Agilent probe sets including gene names, gene symbols, RefSeq accessions, Genbank accessions, UniGene IDs, Entrez Gene IDs, Ensembl IDs, and TIGR IDs were obtained from the Agilent whole zebrafish 4x44K Microarrays annotation website (http://www.home.agilent.com/agilent/). For the probe sets with no Gene IDs assigned, we used information contained in the NCBI (Gene and RefSeq accessions) and ZFIN databases to provide further annotation (Text S1). Gene descriptions are based on genome release Zv9 in the Ensembl database (release 56). The microarray data have been deposited in GEO under accession: GSE37332.

Time-series of microarray expression values were converted from time domain to frequency domain using discrete Fourier transform algorithm. The significance of the observed periodicity compared to random noise was estimated by Fisher's g-test [61] from the GeneCycle package [62] in R. We chose a dominant period of 24 h to identify circadian oscillating genes in our data. The false discovery rate (FDR) was estimated by performing Fisher's g-test on randomly permuted time-series data 1,000 times. The FDR for genes oscillating in both LD and DD (ZCOGs) was estimated from permutations of LD and DD data simultaneously. The selection criteria used were as follows: g-test p values less than 0.5 in LD and g-test p values less than 0.5 in DD with dominant period as 24 h each corresponded to an overall FDR less than 5%. Circadian phases were estimated by fitting the time-series data to a set of cosine curves with 24 h periods but shifting phases [5].

To identify the genes affected by light-entrainment, we searched for the DD amplitude-reduced genes reflecting either decreased peaks or elevated troughs among LD oscillating genes. We fitted the joint LD/DD gene expression values to a set of cosine curves with 24 h periods of shifting phases in LD but constant levels at either +1 (elevated troughs) or −1 (decreased peaks) in DD. A G-test p value less than 0.15 was used to select 3,677 LD oscillating genes (FDR≤10%). A p value less than 0.0001 was used in the fit of joint LD/DD expression values. Among the peak-decreased genes, those with LD phases between CT2 and CT16 were defined as light-induced genes. A delay of 2 hours from light period was used here so that the gene expression increases after light onset. In addition, the light-induced genes with LD phases between CT2 and CT8 were defined as fast light-induced genes and those with LD phases between CT8 and CT16 were defined as slow light-induced genes. Among the trough-elevated genes, those with LD phases between CT16 and CT24 or between CT0 and CT2 were defined as dark-induced genes.

### Light and dark-induced gene expression assay

To examine the expression of light- and dark-induced genes upon light or dark exposure, we raised WT larval zebrafish from birth to 4 dpf at 28°C in DD (constant dark) and LL (constant light) conditions respectively. At CT0 at 5dpf, DD-control larvae remained in DD and DD-LL larvae were transferred to LL conditions. Similarly, LL-control larvae remained in LL and LL-DD larvae were transferred to DD conditions. Twenty five larvae per sample in each group were collected at CT0, CT1, CT2, CT3, CT4, CT6, CT8, CT12, CT16, and CT20 at 5dpf. None of the larvae in the samples was found to have died during this process. The larvae were sucked into freezing tubes, cleared of water, frozen immediately in liquid nitrogen, and stored at −80°C. The collection of samples under dark was performed under dim red light. Real-time PCR was performed to determine the expression of different types of light- and dark-induced genes.

### Promoter analysis of zebrafish genes

The gene annotations and repeat-masked genome sequences for the five teleost species zebrafish, fugu, medaka, stickleback, and Tetraodon were downloaded from ENSEMBL (version 62). Promoter sequences, defined as the region 1 kb upstream to 200 bp downstream of the transcriptional start site (TSS), were extracted from each species using Perl Scripts. For each zebrafish gene, we obtained their orthologous gene information in the other four fish species using ENSEMBL homologs data (version 62). The Pscan program was applied to calculate the enrichment of TF motifs given by TRANSFAC vertebrate TF database for each group of orthologous fish genes [63]. The enriched TF motifs with a p value<0.01 and ranking at least in the top 20 were selected for each orthologous gene group. Fisher's exact test was further applied to calculate the enrichment of TF motifs in a given gene set. For each circadian TF, a sliding window approach described in our previous work [5] was applied to identify the specific time window when their putative targets are enriched.

### Co-expression group analysis

In order to identify co-expressed zebrafish genes, we downloaded all the zebrafish Affymetrix array data with raw data in CEL format deposited in GEO, comprising 57 experiments in total. The raw data were normalized by the rma method. Pearson's correlation coefficient was calculated for each pair of genes. A gene pair was considered to be co-expressed if their Pearson's correlation coefficient is larger than 0.6 and each gene is within the top 20 most correlated genes of the other member of the pair. Using this criterion, 9,926 genes can have at least one other co-expressed gene. The Qcut program [64] was applied to cluster the genes according to their Pearson's correlation coefficients. In total, we obtained 536 clusters of which 435 contained probe sets that can be mapped to the genes on our Agilent zebrafish microarray. For these 435 groups of genes, we calculated the enrichments of the circadian genes, circadian phase, and TF binding sites respectively using Fisher's exact test.

### Circadian gene regulatory network

The circadian gene regulatory network consisted of circadian TFs and their DNA-binding motifs. They were connected by TF-motif and motif-target relationships. Usually, several similar motifs corresponded to one TF. We grouped the motifs using the motif grouping information in TRANSFAC. The smallest p-value of motif-target predictions in the motif group was selected as the p-value of the motif group with the target. The motif-target prediction with p value<0.01 within the top five target predictions for a given motif was used as the criteria to select the motif-target pairs. Fisher's exact tests were used to calculate the enrichment of TF binding sites for the genes expressed in each tissue. The network was visualized using the Cytoscape program [65].

### Morpholino injection

Morpholinos oligonucleotides (MOs) were purchased from Gene Tools. All MOs except standard control were designed to target the start codon region of the genes. The sequence of the *clock* MO was 5′-CAT CCC GGT CTA TGC TGG AGG TCA T-3′ as previously used by Li et al. [56]. The sequence of the *mitfa* (*nacre*) MO was 5′-CAT GTT CAA CTA TGT GTT AGC TTC A -3′ as previously described [66]. The sequence of the standard control MO was 5′-CCT CTT ACC TCA GTT ACA ATT TAT A-3′. MOs were used at the following final doses: *clock* MO: 2.5 ng; *mitfa* MO: 9 ng or 13 ng (microarray analysis). MOs were pressure-injected into 1- to 2-cell stage embryos at a volume of 1 nl using Picospritzer II injectors as previously described [67]. Each MO sample contained 40 individually treated larvae.

### Melanocyte area measurement

WT larvae were raised in a 14 h∶10 h LD cycle from birth to 3 dpf at 28°C. From CT0 of 4 dpf, 10–15 larvae were collected under LD conditions at 4 h intervals for a 72 h period. Larvae were fixed with 4% paraformaldehyde (PFA) for 12 h at 4°C and were then embedded in 1% low melting point agarose. Images were taken using an Olympus microscope SZX16 equipped with a DP71 CCD camera controlled by DP Controller software. The covered area of melanocyte was measured in a 1,360×1,024 pixel frame in the head region from the pineal gland to the optic vesicles excluding the eyes using ImageJ 1.41 software.

### Melanin quantification assay

WT and MO-injected larvae were raised in 14 h∶10 h LD cycle from birth to 3 dpf at 28°C. WT larvae were collected in LD from CT0 of 4 dpf at 4 h intervals for 72 hours. Control MO and *clock* MO-injected larvae were collected under both LD and DD conditions simultaneously from CT0 of 4 dpf at 4 h intervals for 48 hours. Each time point consisted of two or three sample replicates and each sample contained 15 individual larvae. The melanin quantification assay protocol used was based on the published work by Maldonado et al. [68] with minor modifications. Briefly, 15 larvae per sample were placed in 300 µl buffer (20 mM Tris-HCl, 2 mM EGTA, 1 mM PMSF, pH 7.1). They were immediately placed on ice to anesthetize the larvae and then stored at −80°C. The amount of melanin was measured as follows: whole bodies of larvae were homogenized with a disposable pestle (T10, IKA). Then 100 µl DMSO, 500 µl of 2M NaOH and 100 µl dH_2_O were added to each tube. The standard melanin was freshly made from the common cuttlefish (*Sepia officinalis*) (M2649, Sigma) at 1 mg/ml in 1% hydrogen peroxide and diluted to a concentration gradient of 0 µg/ml, 50 µg/ml, 100 µg/ml, 200 µg/ml, 300 µg/ml, and 400 µg/ml. The larval samples and standards were then heated for 2 h at 80°C and centrifuged at 12,000 g for 10 min. The supernatant was collected and the absorbance analyzed at 350 nm using FlexStation 3 (Molecular Devices Inc.). The melanin quantities were obtained by a linear fit to the standard curve using the OriginPro 8 software.

## Supporting Information

Figure S1 Zebrafish larva locomotor activity recording system. (A) An infrared behavioral monitoring platform. The locomotor activities of all larvae in the 96-well plate can be tracked simultaneously. The activity curve of a selected fish in the red rectangle was displayed in real time on the computer screen. The color of the curve reflected the intensity of the locomotion: white, lower than freezing threshold; red, higher than burst threshold; green, between freezing threshold and burst threshold. Freezing threshold and burst threshold parameters for detection were matched to visual observation of the locomotion of single larva. (B) Locomotor activities of larval zebrafish beginning at 5 dpf of development exhibited robust circadian rhythm in LD and the rhythmicity persisted in DD but with reduced amplitude. The average activities of 10 larvae were plotted. The y-axis indicates the mean value of pixels per second. The x-axis indicates light (white) and dark (black) in LD, subjective day (grey) and subjective night (black) in DD.
(TIFF)

Figure S2 *Mitfa* MO knock-down leads to loss of pigmentation in the trunk and smaller eyes. Phenotypes of WT (A–B), control morphants (C–D) and *mitfa* morphants (E–F) at 48 hpf are shown.
(TIF)

Figure S3 The circadian expression of four dark-induced genes: *tyrp1b*, *slc24a5*, *dct* and *mitfa* on the microarrays are shown. These four genes have been known to be involved in melanogenesis.
(TIFF)

Figure S4 Multiple sequence alignment of the promoters of *mitfa* in fish species reveals a conserved CRE.
(TIFF)

Figure S5 Crh receptor1 antagonist antalarmin treatment to 5 dpf larvae. Antalarmin repressed *mitfa* expression level at CT0. *gapdh* was used as a control. The lowest log2-transformed expression level for each gene was normalized to zero. Error bars represent the standard error of mean (SEM) among independent replicates. (***P<0.001, unpaired two-tailed Student's t-test).
(TIFF)

Table S1 Zebrafish circadian oscillating genes.
(PDF)

Table S2 Functional enrichment of ZCOGs (P<0.01).
(PDF)

Table S3 ZCOGs with mouse circadian gene homologs.
(PDF)

Table S4 Genes affected by light entrainment.
(PDF)

Table S5 Primers used for real-time PCR.
(PDF)

Table S6 Transcription factors in zebrafish circadian oscillating genes.
(PDF)

Table S7 Circadian phase of transcription factor and their targets.
(PDF)

Table S8 Enrichment of co-expressed genes in circadian genes, circadian phase, and TF binding sites.
(PDF)

Table S9 Up/Down regulated genes of *mitfa* KD.
(PDF)

Table S10 Comparison of our light-induced genes with Gavriouchkina et al (2010) and Weger et al. (2011) studies.
(PDF)

Text S1 Supplementary Materials and Methods.
(DOC)
