# Supplementary figures and images for: Analysis of a Gene Regulatory Cascade Mediating Circadian Rhythm in Zebrafish
Source: PLoS Comput Biol. 2013 Feb 28;9(2):e1002940. doi: 10.1371/journal.pcbi.1002940 (PMC3585402; doi:10.1371/journal.pcbi.1002940)

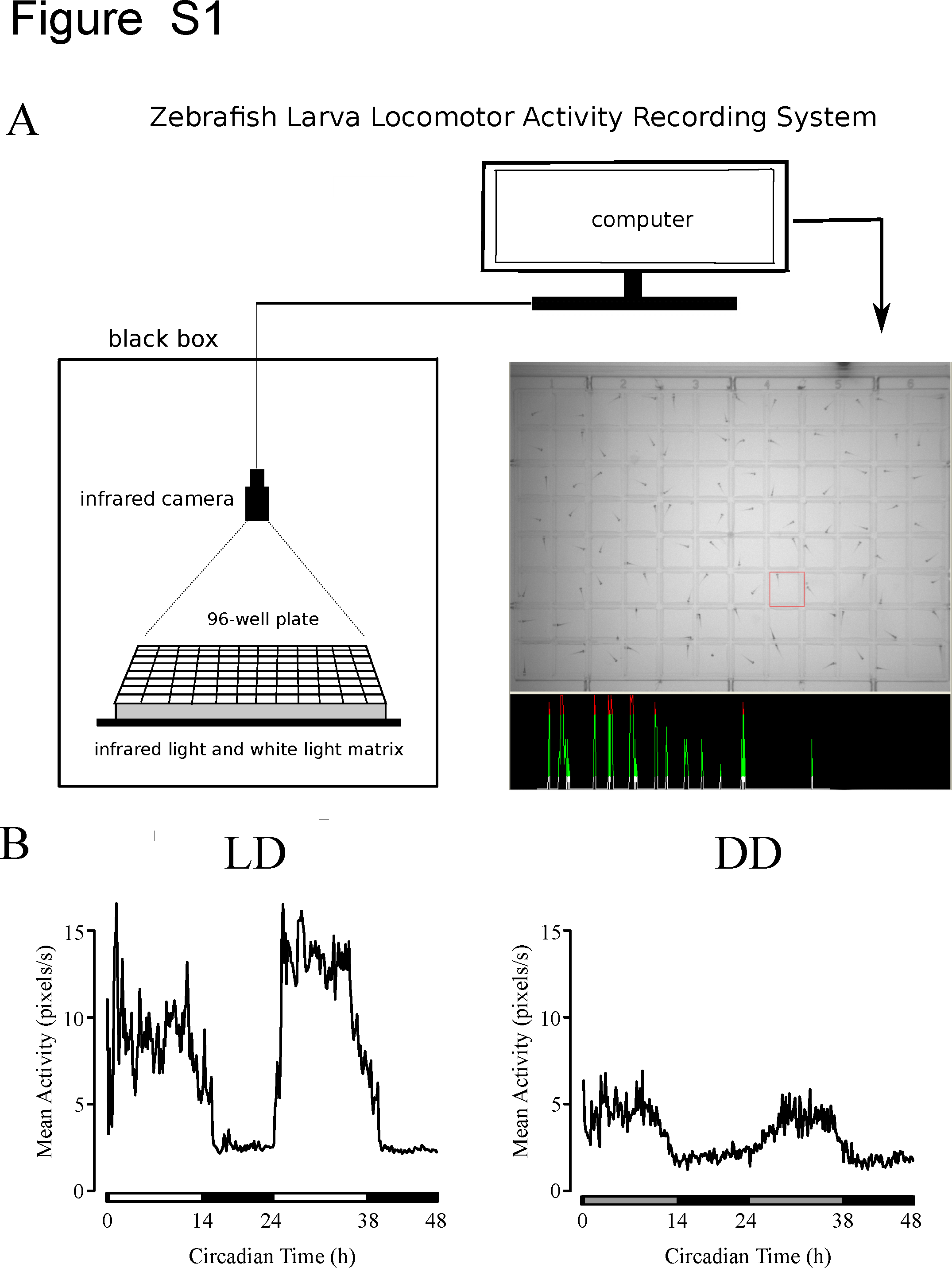

Supplement: Figure S1 — Zebrafish larva locomotor activity recording system. (A) An infrared behavioral monitoring platform. The locomotor activities of all larvae in the 96-well plate can be tracked simultaneously. The activity curve of a selected fish in the red rectangle was displayed in real time on the computer screen. The color of the curve reflected the intensity of the locomotion: white, lower than freezing threshold; red, higher than burst threshold; green, between freezing threshold and burst threshold. Freezing threshold and burst threshold parameters for detection were matched to visual observation of the locomotion of single larva. (B) Locomotor activities of larval zebrafish beginning at 5 dpf of development exhibited robust circadian rhythm in LD and the rhythmicity persisted in DD but with reduced amplitude. The average activities of 10 larvae were plotted. The y-axis indicates the mean value of pixels per second. The x-axis indicates light (white) and dark (black) in LD, subjective day (grey) and subjective night (black) in DD. (TIFF) [file pcbi.1002940.s001.tiff]

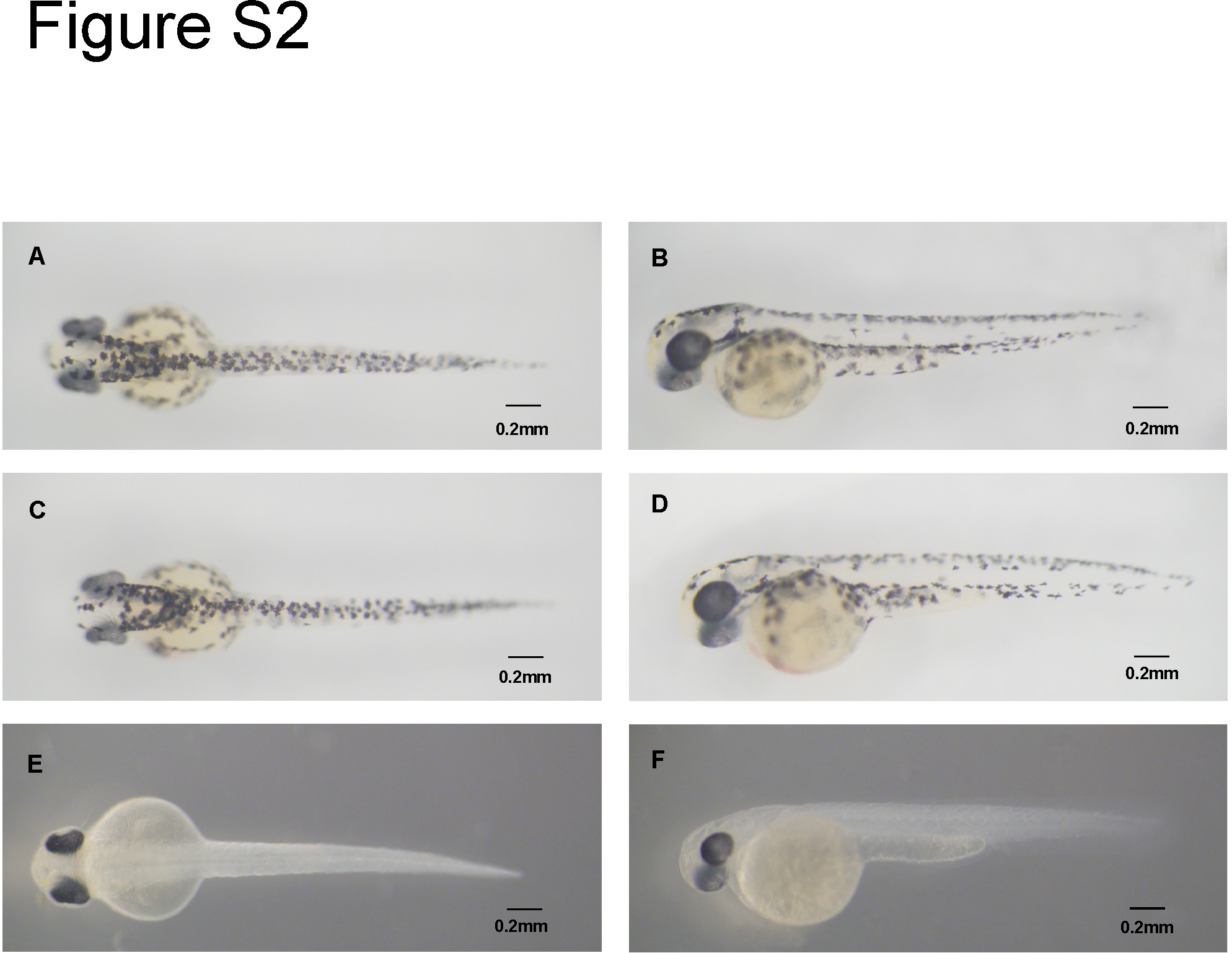

Supplement: Figure S2 — Mitfa MO knock-down leads to loss of pigmentation in the trunk and smaller eyes. Phenotypes of WT (A–B), control morphants (C–D) and mitfa morphants (E–F) at 48 hpf are shown. (TIF) [file pcbi.1002940.s002.tif]

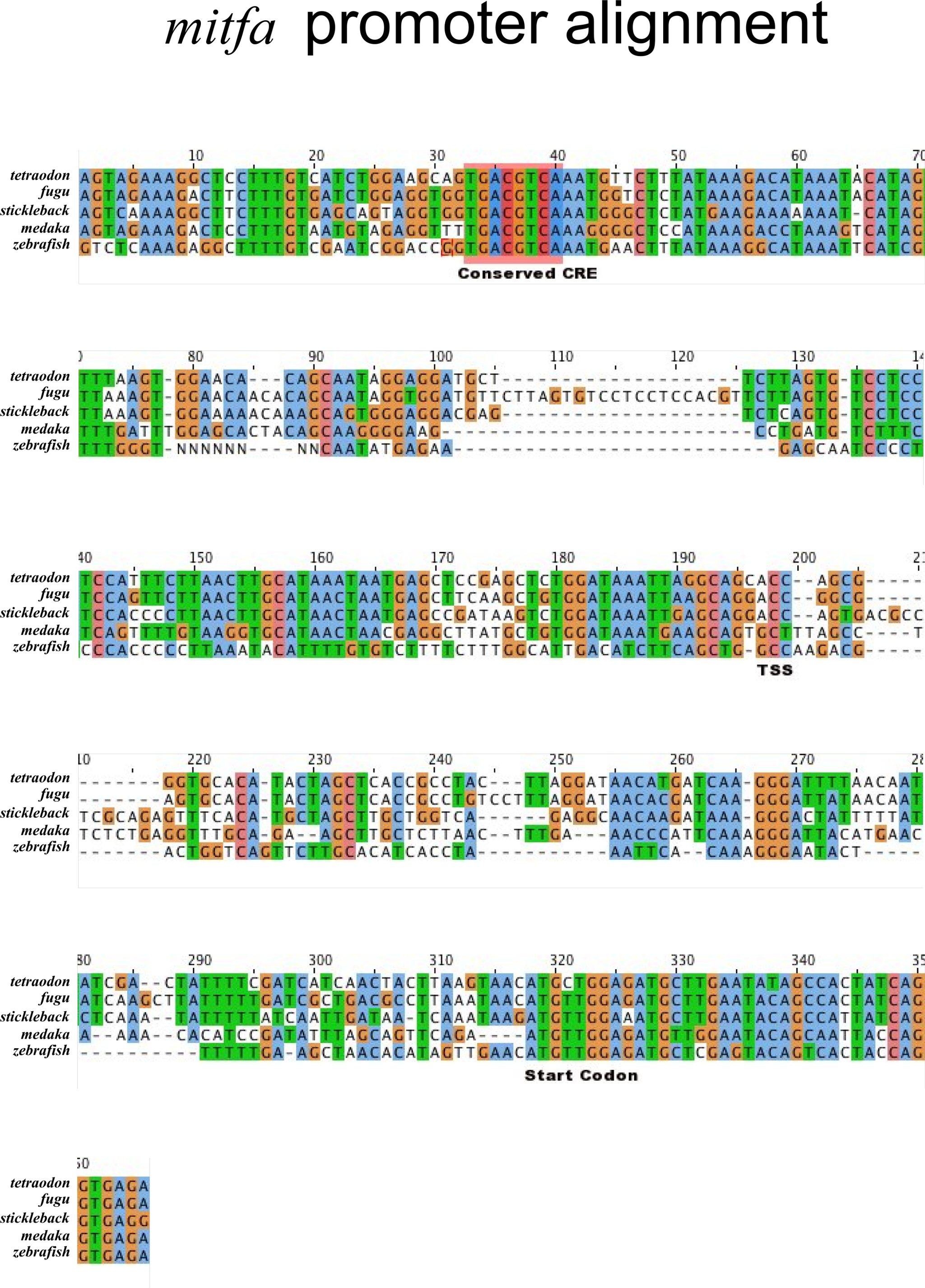

Supplement: Figure S4 — Multiple sequence alignment of the promoters of mitfa in fish species reveals a conserved CRE. (TIFF) [file pcbi.1002940.s004.tiff]
