# Supplementary material for: Analysis of a Gene Regulatory Cascade Mediating Circadian Rhythm in Zebrafish
Source: PLoS Comput Biol. 2013 Feb 28;9(2):e1002940. doi: 10.1371/journal.pcbi.1002940 (PMC3585402; doi:10.1371/journal.pcbi.1002940)
Supplement: Table S1 — Zebrafish circadian oscillating genes. (PDF) [file pcbi.1002940.s006.pdf]

Table S1: Zebrafish circadian oscillating genes

Gene Symbol	Gene ID	Gene Description	Fisher's g test p value (LD)	Fisher's g test p value (DD)	LD Phase	DD Phase	Phase *	FDR **
rbp4	30077	retinol binding protein 4, plasma	8.98E-05	2.06E-04	8.3	8.2	8.3	0.001
neurod	30169	neurogenic differentiation	1.17E-03	1.11E-02	10.5	13.3	11.9	0.001
myog	30200	myogenin	4.40E-02	9.16E-03	10.6	10.7	10.6	0.001
spon2b	30202	spondin 2b, extracellular matrix protein	2.31E-03	3.81E-02	19.3	19.7	19.5	0.001
hsp47	30449	heat shock protein 47	2.10E-03	2.20E-02	5.3	4.8	5.0	0.001
etv5b	30452	ets variant 5b	2.29E-04	1.13E-03	6.5	6.5	6.5	0.001
cmyb	30519	transcription factor cmyb	1.36E-02	2.01E-02	8.3	10.7	9.5	0.001
tef	30674	thyrotroph embryonic factor	2.53E-02	5.73E-03	3.7	3.1	3.4	0.001
irbp	30735	interphotoreceptor retinoid-binding protein	2.76E-04	6.19E-04	5.8	6.2	6.0	0.001
hspe1	58041	heat shock 10 protein 1 (chaperonin 10)	1.10E-03	4.19E-02	2.3	2.8	2.5	0.001
arntl1a	58098	aryl hydrocarbon receptor nuclear translocator-like 1a	1.81E-04	9.24E-04	12.3	14.5	13.4	0.001
arntl2	58099	aryl hydrocarbon receptor nuclear translocator-like 2	2.57E-02	3.04E-02	15.2	17.4	16.3	0.001
per3	58107	period homolog 3 (Drosophila)	2.51E-03	2.07E-03	3.8	4.5	4.2	0.001
apaf1	58131	apoptotic protease activating factor 1	1.17E-03	3.84E-02	12.3	14.8	13.6	0.001
aox3	64265	aldehyde oxidase 3	4.95E-02	4.07E-02	6.5	4.8	5.7	0.001
atp1a2a	64609	ATPase, Na+/K+ transporting, alpha 2a polypeptide	1.00E-02	1.15E-02	0.9	1.9	1.4	0.001
cldn15la	81591	claudin 15-like a	2.93E-02	1.51E-02	5.7	6.2	6.0	0.001
bcl7bb	81595	B-cell CLL/lymphoma 7B, b	3.64E-02	4.98E-02	6.4	6.9	6.6	0.001
bambia	83496	BMP and activin membrane-bound inhibitor (Xenopus lae	6.11E-03	1.15E-02	19.5	21.5	20.5	0.001
cry3	83774	cryptochrome 3	4.15E-03	3.94E-02	1.5	2.8	2.2	0.001
cry4	83775	cryptochrome 4	1.36E-03	5.86E-03	13.3	15.3	14.3	0.001
cry2b	83780	cryptochrome 2b	2.80E-03	1.57E-03	12.9	15.4	14.1	0.001
bcmo1	84039	beta-carotene 15,15'-monooxygenase 1	2.92E-04	1.06E-02	4.7	3.3	4.0	0.001
bcdo2l	84041	beta-carotene 15, 15-dioxygenase 2, like	4.74E-04	1.72E-02	6.8	5.4	6.1	0.001
bcl2l1	114401	bcl2-like 1	3.81E-04	2.15E-02	2.0	3.4	2.7	0.001
angptl3	114421	angiopoietin-like 3	1.64E-02	4.22E-03	4.8	5.2	5.0	0.001
hsf2	140427	heat shock factor 2	6.78E-03	1.32E-02	3.3	4.2	3.8	0.001
prps1a	140620	phosphoribosyl pyrophosphate synthetase 1A	5.25E-03	2.22E-02	0.8	2.2	1.5	0.001
glra3	192124	glycine receptor, alpha 3	4.79E-03	1.10E-02	0.5	3.1	1.8	0.001
glra4b	192125	glycine receptor, alpha 4b	2.16E-03	2.12E-03	13.8	15.0	14.4	0.001
tagln2	259183	transgelin 2	1.43E-03	1.22E-02	5.0	5.5	5.3	0.001

Table S1: Zebrafish circadian oscillating genes

Gene Symbol	Gene ID	Gene Description	Fisher's g test p value (LD)	Fisher's g test p value (DD)	LD Phase	DD Phase	Phase *	FDR **
cyp2ad2	259306	cytochrome P450, family 2, subfamily AD, polypeptide 2	4.49E-03	1.46E-03	4.2	5.3	4.8	0.001
tram1	266757	translocating chain-associating membrane protein 1	2.60E-02	1.66E-02	1.5	2.8	2.1	0.001
sec61al2	266796	Sec61 alpha like 2	5.82E-03	5.29E-03	21.7	0.3	23.0	0.001
hnf1a	266961	HNF1 homeobox a	1.61E-02	1.32E-02	4.2	4.7	4.5	0.001
etf1	266989	eukaryotic translation termination factor 1	2.13E-02	2.65E-02	23.0	1.2	0.1	0.001
pwp2h	266990	PWP2 periodic tryptophan protein homolog (yeast)	4.31E-02	1.44E-02	0.9	2.7	1.8	0.001
cad	266992	carbamoyl-phosphate synthetase 2, aspartate transcarba	6.02E-03	6.59E-04	2.1	4.6	3.3	0.001
bckdk	282677	branched chain alpha-ketoacid dehydrogenase kinase	5.44E-03	3.01E-02	13.8	15.3	14.6	0.001
diexf	286787	digestive organ expansion factor homolog	8.99E-04	3.52E-04	0.8	3.7	2.3	0.001
mc5ra	286834	melanocortin 5a receptor	4.63E-02	4.06E-02	3.7	7.5	5.6	0.001
mki67ip	317644	mki67 (FHA domain) interacting nucleolar phosphoprotei	3.44E-02	1.87E-02	23.0	1.3	0.2	0.001
zgc:63569	321056	zgc:63569	8.75E-03	1.43E-04	12.2	14.5	13.4	0.001
mcmbp	321119	minichromosome maintenance complex binding protein	1.61E-02	7.87E-03	0.8	2.8	1.8	0.001
lmbr1l	321314	limb region 1 like	3.12E-02	1.87E-02	4.3	5.3	4.8	0.001
cd63	321461	Cd63 antigen	3.21E-04	1.09E-03	16.5	17.8	17.2	0.001
zgc:109901	321700	zgc:109901	3.73E-02	1.65E-02	23.7	2.5	1.1	0.001
gnl3	321897	guanine nucleotide binding protein-like 3 (nucleolar)	2.11E-02	4.55E-02	22.2	0.2	23.2	0.001
cth	322055	cystathionase (cystathionine gamma-lyase)	2.46E-02	2.09E-02	5.5	5.0	5.3	0.001
ctps	322089	CTP synthase	1.97E-02	8.45E-03	22.2	23.8	23.0	0.001
wu:fb51f10	322152	wu:fb51f10	1.24E-03	2.41E-03	12.2	14.2	13.2	0.001
adkb	322229	adenosine kinase b	9.21E-04	8.30E-03	2.5	4.0	3.3	0.001
sult3st2	322415	sulfotransferase family 3, cytosolic sulfotransferase 2	1.65E-02	6.16E-03	5.8	5.2	5.5	0.001
cd9a	322455	CD9 antigen (p24) a	4.23E-02	2.66E-03	15.0	15.8	15.4	0.001
dnajc3	322544	DnaJ (Hsp40) homolog, subfamily C, member 3	7.27E-05	5.03E-04	0.0	1.0	0.5	0.001
cx32.3	322617	connexin 32.3	2.60E-02	2.51E-02	23.5	1.0	0.3	0.001
tomm40	322645	translocase of outer mitochondrial membrane 40 homolo	4.26E-02	1.93E-02	0.5	0.8	0.7	0.001
myst2	323181	MYST histone acetyltransferase 2	4.09E-02	4.29E-02	12.0	14.8	13.4	0.001
zgc:163008	323951	zgc:163008	2.46E-02	4.86E-02	15.3	14.7	15.0	0.001
wu:fc15e02	323961	wu:fc15e02	1.35E-02	2.54E-02	2.3	3.0	2.6	0.001
dspa	324023	desmoplakin a	4.12E-02	4.25E-03	4.2	5.2	4.7	0.001
cd82a	324098	CD82 antigen, a	2.61E-03	1.98E-02	14.8	16.2	15.5	0.001

Table S1: Zebrafish circadian oscillating genes

Gene Symbol	Gene ID	Gene Description	Fisher's g test p value (LD)	Fisher's g test p value (DD)	LD Phase	DD Phase	Phase *	FDR **
wu:fc20c04	324148	wu:fc20c04	1.28E-03	2.63E-02	15.5	18.2	16.9	0.001
fam164a	324412	family with sequence similarity 164, member A	1.47E-03	7.94E-03	13.0	14.3	13.7	0.001
bhlhe40	324413	basic helix-loop-helix family, member e40	9.94E-06	7.41E-03	5.2	5.3	5.3	0.001
phb2	324421	prohibitin 2	3.62E-02	3.94E-02	1.8	2.8	2.3	0.001
si:ch73-22c10.1	324543	si:ch73-22c10.1	1.12E-02	1.19E-02	13.2	14.3	13.8	0.001
nucb2b	326050	nucleobindin 2b	4.05E-04	9.37E-03	3.7	4.8	4.3	0.001
cnn3a	326931	calponin 3, acidic a	8.46E-05	3.78E-02	7.2	5.8	6.5	0.001
dnajb1b	327244	DnaJ (Hsp40) homolog, subfamily B, member 1b	1.77E-04	4.47E-02	7.6	10.0	8.8	0.001
sec22bb	327277	SEC22 vesicle trafficking protein homolog B (S. cerevisia	8.88E-03	4.83E-02	14.8	21.2	18.0	0.001
zgc:158623	327345	zgc:158623	3.34E-03	6.11E-03	17.9	18.6	18.2	0.001
zgc:63480	327556	zgc:63480	2.81E-03	2.07E-02	2.8	2.8	2.8	0.001
lpcat4	327566	lysophosphatidylcholine acyltransferase 4	2.34E-04	3.05E-03	4.2	5.5	4.9	0.001
gclm	333974	glutamate-cysteine ligase, modifier subunit	2.30E-02	1.16E-02	18.7	22.7	20.7	0.001
naa15b	334099	N(alpha)-acetyltransferase 15, NatA auxiliary subunit b	1.34E-02	5.17E-03	21.0	21.5	21.3	0.001
alg1	334161	asparagine-linked glycosylation 1 homolog (yeast, beta-1	4.36E-04	2.98E-02	13.4	15.4	14.4	0.001
zgc:77057	334166	zgc:77057	2.41E-03	4.71E-02	16.0	17.0	16.5	0.001
ldhd	334208	lactate dehydrogenase D	4.43E-02	4.57E-02	5.5	5.2	5.4	0.001
hnrnpa0	334222	heterogeneous nuclear ribonucleoprotein A0	4.49E-02	1.10E-02	1.0	2.8	1.9	0.001
cuedc2	334351	CUE domain containing 2	1.79E-02	6.86E-03	11.5	10.7	11.1	0.001
zgc:101724	334483	zgc:101724	1.55E-03	2.41E-02	19.2	20.2	19.7	0.001
prkcd	334571	protein kinase C, delta	4.50E-02	4.40E-02	13.3	14.2	13.8	0.001
mrpl39	334588	mitochondrial ribosomal protein L39	4.92E-02	1.62E-02	23.2	0.7	24.0	0.001
trmt2a	334936	trm2 tRNA methyltransferase 2 homolog A (S. cerevisiae	2.34E-02	4.24E-03	0.2	2.5	1.4	0.001
ebna1bp2	335049	EBNA1 binding protein 2	1.60E-02	1.03E-02	15.8	2.4	21.1	0.001
si:rp71-61h23.4	335278	si:rp71-61h23.4	5.97E-03	9.51E-03	3.2	2.5	2.9	0.001
wu:fl07c04	335281	wu:fl07c04	2.20E-02	1.86E-02	1.5	2.7	2.1	0.001
zgc:64190	335628	zgc:64190	6.86E-03	4.55E-03	16.8	17.0	16.9	0.001
gngt2a	335655	guanine nucleotide binding protein (G protein), gamma tr	3.27E-02	2.34E-03	12.9	13.7	13.3	0.001
gngt1	335656	guanine nucleotide binding protein (G protein), gamma tr	5.66E-03	4.73E-03	12.0	12.0	12.0	0.001
nme2a	335716	non-metastatic cells 2a, protein (NM23B) expressed in	8.50E-03	2.46E-02	2.5	2.3	2.4	0.001
cox7a2l	335751	cytochrome c oxidase subunit VIIa polypeptide 2 like	1.09E-02	4.22E-02	21.8	21.3	21.6	0.001

Table S1: Zebrafish circadian oscillating genes

Gene Symbol	Gene ID	Gene Description	Fisher's g test p value (LD)	Fisher's g test p value (DD)	LD Phase	DD Phase	Phase *	FDR **
gas6	335830	growth arrest-specific 6	3.48E-02	3.93E-02	15.5	18.5	17.0	0.001
wu:fj44c04	336034	wu:fj44c04	5.27E-05	3.54E-02	0.7	3.7	2.2	0.001
wu:fb10g03	336442	wu:fb10g03	1.72E-02	4.74E-02	1.0	2.5	1.8	0.001
ptgds	336492	prostaglandin D2 synthase	1.03E-03	4.46E-02	7.8	6.8	7.3	0.001
ifi30	336503	interferon gamma inducible protein 30	2.60E-02	4.53E-02	15.7	17.2	16.5	0.001
grn2	336575	granulin 2	1.01E-02	7.39E-03	18.5	18.7	18.6	0.001
samsn1a	336695	SAM domain, SH3 domain and nuclear localisation signa	6.51E-03	2.61E-02	9.6	11.8	10.7	0.001
dnajc21	336984	DnaJ (Hsp40) homolog, subfamily C, member 21	5.75E-03	1.65E-02	20.8	22.3	21.5	0.001
setd3	337193	SET domain containing 3	6.80E-04	2.21E-02	2.7	3.8	3.3	0.001
tbcb	337350	tubulin folding cofactor B	1.10E-02	2.80E-02	5.3	6.0	5.7	0.001
rrs1	337399	RRS1 ribosome biogenesis regulator homolog (S. cerevi	1.61E-02	1.70E-02	22.5	0.6	23.5	0.001
nfatc2ip	337574	nuclear factor of activated T-cells, cytoplasmic, calcineur	2.14E-03	3.73E-04	10.3	13.3	11.8	0.001
wu:fj84d10	337575	wu:fj84d10	1.84E-02	1.66E-02	22.7	1.5	0.1	0.001
dact2	337795	dapper homolog 2, antagonist of beta-catenin (xenopus)	1.64E-02	3.81E-02	3.3	5.5	4.4	0.001
zgc:55259	338155	zgc:55259	2.84E-02	3.80E-03	0.5	2.0	1.3	0.001
ppdpfa	338304	pancreatic progenitor cell differentiation and proliferation	8.85E-04	1.89E-03	17.4	18.0	17.7	0.001
selt2	352917	selenoprotein T, 2	1.01E-02	4.01E-02	16.3	18.8	17.5	0.001
clock3	352927	clock homolog 3 (mouse)	1.33E-02	1.59E-02	14.8	15.3	15.1	0.001
arntl1b	352935	aryl hydrocarbon receptor nuclear translocator-like 1b	2.42E-03	1.39E-03	11.9	13.8	12.8	0.001
ier5	359827	immediate early response 5	5.73E-03	3.30E-02	10.5	13.3	11.9	0.001
slc34a2b	359838	solute carrier family 34 (sodium phosphate), member 2b	2.20E-02	2.32E-02	11.7	13.2	12.5	0.001
nfe2l2	360149	nuclear factor (erythroid-derived 2)-like 2	1.37E-02	2.35E-02	6.7	7.5	7.1	0.001
ypel3	368214	yippee-like 3	6.35E-03	9.64E-03	12.3	14.8	13.6	0.001
sult1st3	368270	sulfotransferase family 1, cytosolic sulfotransferase 3	3.52E-03	6.55E-04	5.0	5.4	5.2	0.001
ldlrap1a	368278	low density lipoprotein receptor adaptor protein 1a	6.10E-06	6.42E-04	14.5	16.3	15.4	0.001
abcc4	368620	ATP-binding cassette, sub-family C (CFTR/MRP), memb	4.01E-03	4.01E-02	1.8	2.1	2.0	0.001
ptf1a	368662	pancreas specific transcription factor, 1a	3.96E-02	1.90E-02	18.3	20.8	19.6	0.001
scel	368684	sciellin	1.25E-02	2.20E-02	1.7	5.2	3.5	0.001
si:busm1-163l24.3	368753	si:busm1-163l24.3	4.15E-02	4.23E-02	13.8	16.7	15.3	0.001
guca1c	373099	guanylate cyclase activator 1C	7.77E-03	1.46E-02	3.6	1.5	2.6	0.001
grk7b	373871	G protein-coupled receptor kinase 7b	3.79E-02	3.80E-02	1.3	2.1	1.7	0.001

Table S1: Zebrafish circadian oscillating genes

Gene Symbol	Gene ID	Gene Description	Fisher's g test p value (LD)	Fisher's g test p value (DD)	LD Phase	DD Phase	Phase *	FDR **
dhcr7	378446	7-dehydrocholesterol reductase	1.55E-02	1.64E-03	5.8	6.9	6.3	0.001
fbxl3a	378451	F-box and leucine-rich repeat protein 3A	1.19E-03	1.14E-02	13.0	15.0	14.0	0.001
mtr	378847	5-methyltetrahydrofolate-homocysteine methyltransferas	2.03E-02	2.57E-02	2.2	3.3	2.8	0.001
pdca	386613	phosducin a	2.73E-05	1.83E-02	3.9	5.1	4.5	0.001
tob1a	386629	transducer of ERBB2, 1a	2.58E-03	4.12E-02	2.3	3.0	2.7	0.001
slc13a1	387592	solute carrier family 13 (sodium/sulphate symporters), m	1.39E-03	1.40E-02	4.7	4.0	4.4	0.001
hsd17b4	393105	hydroxysteroid (17-beta) dehydrogenase 4	2.77E-03	3.31E-03	6.5	7.0	6.8	0.001
rasl11b	393109	RAS-like, family 11, member B	1.23E-02	1.57E-02	4.0	3.0	3.5	0.001
slmapb	393146	sarcolemma associated protein b	1.51E-03	3.20E-02	4.2	3.7	4.0	0.001
zgc:56005	393155	zgc:56005	2.01E-03	5.19E-03	5.7	9.2	7.5	0.001
polr3c	393201	polymerase (RNA) III (DNA directed) polypeptide C	1.48E-02	4.71E-03	22.8	1.5	0.2	0.001
vil1l	393207	villin 1 like	3.81E-02	4.09E-02	3.2	4.8	4.0	0.001
rnaseh2b	393241	ribonuclease H2, subunit B	3.76E-02	2.98E-02	4.0	4.8	4.4	0.001
zgc:56407	393257	zgc:56407	1.72E-02	3.14E-02	23.3	0.7	0.0	0.001
psmd6	393261	proteasome (prosome, macropain) 26S subunit, non-ATI	2.33E-02	4.82E-02	18.2	19.7	19.0	0.001
zgc:56066	393282	zgc:56066	1.84E-02	3.74E-02	0.4	2.3	1.3	0.001
slc10a2	393329	solute carrier family 10 (sodium/bile acid cotransporter fa	3.99E-03	4.43E-02	22.3	0.2	23.3	0.001
zgc:65987	393539	zgc:65987	7.60E-03	1.78E-02	16.9	17.9	17.4	0.001
cyp2p9	393592	cytochrome P450, family 2, subfamily P, polypeptide 9	3.70E-03	3.52E-02	4.0	5.5	4.8	0.001
acsl4a	393622	acyl-CoA synthetase long-chain family member 4a	9.55E-06	2.54E-03	2.3	4.0	3.2	0.001
arl3l1	393666	ADP-ribosylation factor-like 3, like 1	5.90E-03	4.31E-02	6.5	20.8	1.7	0.001
guk1	393697	guanylate kinase 1	1.19E-02	5.07E-03	18.5	19.3	18.9	0.001
gabra6a	393704	gamma-aminobutyric acid (GABA) A receptor, alpha 6a	1.23E-03	3.25E-02	1.3	23.5	0.4	0.001
smfn	393717	small fragment nuclease	1.88E-02	4.69E-02	0.2	2.3	1.3	0.001
rpe65a	393724	retinal pigment epithelium-specific protein 65a	1.73E-03	4.56E-03	23.5	1.0	0.3	0.001
kcnip3	393755	Kv channel interacting protein 3, calsenilin	3.26E-04	7.42E-03	2.8	3.3	3.1	0.001
rdh8l	393761	retinol dehydrogenase 8 like	3.53E-03	3.92E-02	21.8	7.5	2.7	0.001
prdx6	393778	peroxiredoxin 6	2.50E-04	4.45E-02	5.5	5.8	5.7	0.001
ppp1r3cb	393807	protein phosphatase 1, regulatory (inhibitor) subunit 3Cb	1.17E-02	3.96E-02	9.3	11.8	10.6	0.001
znrf1	393844	zinc and ring finger 1	3.08E-02	8.03E-03	14.5	14.5	14.5	0.001
zgc:56064	393885	zgc:56064	4.67E-03	1.10E-02	18.0	19.0	18.5	0.001

Table S1: Zebrafish circadian oscillating genes

Gene Symbol	Gene ID	Gene Description	Fisher's g test p value (LD)	Fisher's g test p value (DD)	LD Phase	DD Phase	Phase *	FDR **
abhd2a	393888	abhydrolase domain containing 2a	5.50E-03	1.50E-02	3.7	3.8	3.8	0.001
ttc4	393981	tetratricopeptide repeat domain 4	1.02E-02	4.58E-02	23.3	0.8	0.1	0.001
aacs	393984	acetoacetyl-CoA synthetase	6.87E-03	3.24E-02	1.3	21.8	23.6	0.001
lipc	393997	lipase, hepatic	4.59E-05	5.12E-03	7.0	7.3	7.2	0.001
mxd3	394031	MAX dimerization protein 3	2.14E-04	6.48E-04	11.8	13.5	12.7	0.001
ftcd	394052	formiminotransferase cyclodeaminase	8.67E-04	2.32E-03	10.8	11.2	11.0	0.001
fus	394058	fusion (involved in t(12;16) in malignant liposarcoma)	2.47E-03	1.76E-03	15.0	15.2	15.1	0.001
sh3gl2	394091	SH3-domain GRB2-like 2	4.87E-04	3.82E-02	2.0	2.5	2.3	0.001
aldh4a1	394133	aldehyde dehydrogenase 4 family, member A1	1.11E-02	1.34E-03	5.0	5.0	5.0	0.001
dusp4	394146	dual specificity phosphatase 4	1.01E-03	5.00E-02	6.5	0.5	3.5	0.001
sb:cb1058	394209	sb:cb1058	4.15E-02	2.27E-02	1.7	1.7	1.7	0.001
rrp9	402808	ribosomal RNA processing 9, small subunit (SSU) proces	2.18E-02	4.19E-02	23.5	1.8	0.7	0.001
gcat	402822	glycine C-acetyltransferase	6.29E-05	7.54E-03	5.3	5.5	5.4	0.001
si:dkey-4e7.3	402865	si:dkey-4e7.3	2.03E-03	3.87E-02	2.7	3.7	3.2	0.001
mpp6a	402878	membrane protein, palmitoylated 6a (MAGUK p55 subfa	2.69E-03	1.54E-02	23.7	0.3	0.0	0.001
mmgt1	402891	membrane magnesium transporter 1	1.55E-02	3.44E-02	19.9	21.7	20.8	0.001
rgl1	402933	ral guanine nucleotide dissociation stimulator-like 1	1.95E-03	1.90E-03	12.7	12.8	12.8	0.001
pnp4b	402940	purine nucleoside phosphorylase 4b	5.62E-03	9.59E-03	6.0	6.3	6.1	0.001
zgc:77906	402945	zgc:77906	3.30E-03	1.64E-03	4.5	4.7	4.6	0.001
si:dkey-194e6.1	402976	si:dkey-194e6.1	4.51E-02	3.24E-02	11.4	4.1	7.8	0.001
zgc:77838	402980	zgc:77838	2.58E-02	3.16E-02	16.7	17.5	17.1	0.001
hnrnph1	402984	heterogeneous nuclear ribonucleoprotein H1	5.12E-03	7.33E-03	14.0	14.0	14.0	0.001
atad3b	403004	ATPase family, AAA domain containing 3B	1.80E-02	7.40E-03	23.0	1.0	0.0	0.001
dock8	403040	dedicator of cytokinesis 8	2.40E-04	2.99E-02	12.3	14.7	13.5	0.001
rgn	403070	regucalcin	3.58E-02	6.90E-03	7.0	6.5	6.8	0.001
gnmt	403338	glycine N-methyltransferase	2.79E-02	3.98E-02	9.5	7.8	8.7	0.001
traf4b	404035	tnf receptor-associated factor 4b	2.69E-03	2.92E-02	15.5	16.8	16.2	0.001
cx52.9	404625	connexin 52.9	3.55E-02	4.33E-02	18.2	19.3	18.8	0.001
mao	404730	monoamine oxidase	4.42E-02	3.86E-02	4.0	5.0	4.5	0.001
blnk	405764	B-cell linker	4.59E-02	1.95E-02	17.8	18.8	18.3	0.001
zgc:73371	405817	zgc:73371	1.32E-04	4.45E-02	4.2	3.7	4.0	0.001

Table S1: Zebrafish circadian oscillating genes

Gene Symbol	Gene ID	Gene Description	Fisher's g test p value (LD)	Fisher's g test p value (DD)	LD Phase	DD Phase	Phase *	FDR **
clic5	405833	chloride intracellular channel 5	1.12E-02	1.63E-03	5.5	6.8	6.2	0.001
spast	405851	spastin	1.99E-02	2.94E-02	14.2	15.7	15.0	0.001
per1b	406204	period homolog 1b (Drosophila)	3.91E-04	1.56E-03	2.3	4.5	3.4	0.001
sema6d	406272	sema domain, transmembrane domain (TM), and cytopla	1.45E-02	2.67E-02	13.6	13.4	13.5	0.001
itpk1	406290	inositol 1,3,4-triphosphate 5/6 kinase	2.63E-03	2.98E-02	5.0	4.8	4.9	0.001
chia.2	406338	chitinase, acidic.2	6.59E-03	7.43E-03	7.8	6.5	7.2	0.001
hk2	406339	hexokinase 2	4.35E-02	1.11E-02	21.2	23.0	22.1	0.001
znf259	406382	zinc finger protein 259	3.18E-03	1.31E-02	0.5	1.5	1.0	0.001
sort1a	406511	sortilin 1a	2.02E-02	3.19E-02	14.2	15.4	14.8	0.001
csnk1db	406533	casein kinase 1, delta b	4.68E-04	2.07E-03	2.0	2.7	2.4	0.001
decr2	406623	2,4-dienoyl CoA reductase 2, peroxisomal	1.74E-02	2.93E-02	4.8	4.3	4.6	0.001
lgmn	406625	legumain	1.82E-04	9.84E-03	15.5	17.8	16.7	0.001
grtp1a	406711	growth hormone regulated TBC protein 1a	1.19E-02	2.36E-02	23.7	0.2	24.0	0.001
eif3jb	406823	eukaryotic translation initiation factor 3, subunit Jb	4.77E-03	5.59E-03	22.3	23.8	23.1	0.001
sesn3	406840	sestrin 3	1.19E-03	2.97E-03	11.8	14.0	12.9	0.001
mrps31	407642	mitochondrial ribosomal protein S31	1.75E-02	9.14E-04	0.2	1.2	0.7	0.001
prlra	407651	prolactin receptor a	3.26E-03	2.80E-02	1.0	2.2	1.6	0.001
wu:fd55e03	407663	wu:fd55e03	1.53E-03	1.07E-03	3.8	4.5	4.2	0.001
rpia	407673	ribose 5-phosphate isomerase A (ribose 5-phosphate ep	9.09E-03	2.42E-02	2.9	3.3	3.1	0.001
lrrfip1a	407677	leucine rich repeat (in FLII) interacting protein 1a	1.28E-03	3.92E-02	4.8	3.5	4.1	0.001
akr1b1	415138	aldo-keto reductase family 1, member B1 (aldose reducta	7.83E-04	1.56E-04	5.3	4.9	5.1	0.001
msrb3	415184	methionine sulfoxide reductase B3	5.25E-04	4.26E-04	7.2	6.6	6.9	0.001
cdkrap3	415195	CDK5 regulatory subunit associated protein 3	2.14E-02	1.33E-04	23.5	1.0	0.3	0.001
rpp40l	415238	ribonuclease P 40 subunit like	1.53E-02	8.45E-03	0.7	2.3	1.5	0.001
rpf1	415250	ribosome production factor 1 homolog (S. cerevisiae)	4.53E-02	3.39E-02	23.2	0.3	23.8	0.001
mrrf	431721	mitochondrial ribosome recycling factor	8.98E-04	1.88E-02	1.3	0.4	0.8	0.001
zgc:92432	431722	zgc:92432	1.06E-02	4.02E-03	20.3	21.2	20.8	0.001
tfcp2l1	431761	transcription factor CP2-like 1	8.48E-03	5.56E-03	14.0	15.3	14.7	0.001
nus1	436629	nuclear undecaprenyl pyrophosphate synthase 1 homolo	4.70E-02	3.25E-02	0.7	1.5	1.1	0.001
trim55a	436631	tripartite motif-containing 55a	1.29E-03	2.50E-03	15.2	17.0	16.1	0.001
zgc:92090	436640	zgc:92090	2.40E-02	4.61E-02	11.2	13.6	12.4	0.001

Table S1: Zebrafish circadian oscillating genes

Gene Symbol	Gene ID	Gene Description	Fisher's g test p value (LD)	Fisher's g test p value (DD)	LD Phase	DD Phase	Phase *	FDR **
zgc:92035	436665	zgc:92035	1.17E-02	1.43E-02	3.8	6.0	4.9	0.001
zgc:92712	436668	zgc:92712	8.91E-03	1.13E-02	2.2	2.7	2.5	0.001
arr3a	436678	arrestin 3a, retinal (X-arrestin)	4.16E-02	3.03E-03	4.7	2.8	3.7	0.001
zgc:92658	436699	zgc:92658	1.70E-02	6.62E-03	20.9	22.9	21.9	0.001
cldn15lb	436719	claudin 15-like b	2.01E-02	1.64E-02	15.4	16.5	15.9	0.001
zgc:92739	436849	zgc:92739	4.95E-02	2.41E-03	0.4	2.6	1.5	0.001
gcshb	436852	glycine cleavage system protein H (aminomethyl carrier)	8.32E-03	1.13E-02	22.5	1.0	23.8	0.001
sult1st6	436872	sulfotransferase family 1, cytosolic sulfotransferase 6	1.18E-02	8.83E-03	3.8	4.5	4.2	0.001
rgp1	436881	RGP1 retrograde golgi transport homolog (S. cerevisiae)	1.55E-03	8.09E-03	14.7	16.7	15.7	0.001
nrbf2	436886	nuclear receptor binding factor 2	1.02E-02	1.79E-03	13.7	15.0	14.4	0.001
zgc:92254	436894	zgc:92254	2.10E-03	1.33E-04	7.5	7.0	7.3	0.001
slc17a9b	436908	solute carrier family 17, member 9b	4.92E-03	3.80E-03	22.3	1.3	23.8	0.001
camk2n2	436915	calcium/calmodulin-dependent protein kinase II inhibitor :	7.86E-03	1.28E-02	17.7	18.5	18.1	0.001
slc1a1	436939	solute carrier family 1 (neuronal/epithelial high affinity glu	6.15E-04	6.65E-03	16.2	16.3	16.3	0.001
zgc:91931	436945	zgc:91931	7.63E-04	5.26E-04	13.2	15.2	14.2	0.001
cdc34b	436961	cell division cycle 34 homolog (S. cerevisiae) b	1.15E-03	4.58E-03	12.8	14.7	13.7	0.001
zgc:92630	436969	zgc:92630	1.09E-04	8.88E-03	5.8	4.7	5.3	0.001
pde6d	436981	phosphodiesterase 6D, cGMP-specific, rod, delta	1.00E-02	1.26E-02	1.7	2.2	2.0	0.001
zgc:100980	437012	zgc:100980	5.08E-03	4.38E-02	1.8	3.0	2.4	0.001
rab3aa	445024	RAB3A, member RAS oncogene family, a	7.24E-04	2.17E-03	20.4	20.0	20.2	0.001
hars2	445046	histidyl-tRNA synthetase 2, mitochondrial (putative)	5.24E-03	3.01E-02	1.2	3.2	2.2	0.001
spsb4b	445080	splA/ryanodine receptor domain and SOCS box containir	6.56E-05	6.86E-03	12.0	13.3	12.7	0.001
zgc:92006	445101	zgc:92006	3.18E-02	2.29E-02	21.7	22.8	22.3	0.001
cdk5r2b	445114	cyclin-dependent kinase 5, regulatory subunit 2	2.66E-04	3.88E-03	3.2	1.5	2.4	0.001
zgc:101071	445175	zgc:101071	3.03E-02	3.51E-02	23.2	2.2	0.7	0.001
mnd1	445203	meiotic nuclear divisions 1 homolog (S. cerevisiae)	3.23E-05	2.62E-02	9.8	9.5	9.7	0.001
mphosph6	445224	M-phase phosphoprotein 6	8.77E-03	1.53E-02	22.8	0.7	23.8	0.001
harbi1	445279	harbinger transposase derived 1	2.94E-02	9.96E-03	10.7	11.8	11.3	0.001
tmem144a	445495	transmembrane protein 144a	2.08E-02	5.69E-03	15.2	17.0	16.1	0.001
zgc:92445	447802	zgc:92445	2.68E-02	2.43E-02	3.2	0.8	2.0	0.001
rassf1	447811	Ras association (RalGDS/AF-6) domain family 1	4.64E-02	1.85E-02	13.0	14.8	13.9	0.001

Table S1: Zebrafish circadian oscillating genes

Gene Symbol	Gene ID	Gene Description	Fisher's g test p value (LD)	Fisher's g test p value (DD)	LD Phase	DD Phase	Phase *	FDR **
zgc:92161	447817	zgc:92161	3.33E-04	8.34E-03	8.2	8.8	8.5	0.001
fth1b	447823	ferritin, heavy polypeptide 1b	3.17E-04	1.26E-02	14.8	15.0	14.9	0.001
eif4e3	447850	eukaryotic translation initiation factor 4E family member 3	3.21E-04	2.00E-03	13.0	15.0	14.0	0.001
dirc2	447858	disrupted in renal carcinoma 2	2.20E-02	1.21E-02	14.7	15.7	15.2	0.001
acsl5	447860	acyl-CoA synthetase long-chain family member 5	7.57E-04	1.61E-02	4.2	6.0	5.1	0.001
zgc:92470	447867	zgc:92470	1.95E-02	2.19E-02	4.0	3.7	3.9	0.001
smyd5	447875	SMYD family member 5	3.06E-02	2.01E-02	21.8	23.2	22.5	0.001
itgb1bp3	447879	integrin beta 1 binding protein 3	5.45E-03	1.11E-02	1.0	2.4	1.7	0.001
zgc:101748	447888	zgc:101748	2.93E-02	4.85E-02	23.8	12.0	17.9	0.001
si:ch211-217g15.2	447896	si:ch211-217g15.2	3.48E-02	9.72E-04	23.3	2.5	0.9	0.001
cox17	447914	COX17 cytochrome c oxidase assembly homolog (S. cer	3.02E-03	2.71E-03	5.2	2.8	4.0	0.001
zgc:103748	447917	zgc:103748	5.22E-05	2.64E-02	23.3	0.2	23.8	0.001
zgc:103625	447931	zgc:103625	1.72E-02	1.44E-02	19.3	18.7	19.0	0.001
crata	449545	carnitine O-acetyltransferase a	3.59E-03	1.15E-02	11.3	14.0	12.7	0.001
stk25a	449653	serine/threonine kinase 25a	1.81E-02	1.83E-02	15.7	16.7	16.2	0.001
acn9	449654	ACN9 homolog (S. cerevisiae)	6.74E-03	9.55E-03	2.8	1.8	2.3	0.001
osbpl7	449656	oxysterol binding protein-like 7	6.39E-03	3.91E-02	3.3	1.2	2.2	0.001
mylk4	449707	myosin light chain kinase family, member 4	6.46E-03	3.93E-02	21.8	23.5	22.7	0.001
mgst1	449784	microsomal glutathione S-transferase 1	7.84E-03	3.12E-02	6.8	7.7	7.3	0.001
pgm2l1	449945	phosphoglucomutase 2-like 1	5.36E-04	2.08E-02	12.5	16.2	14.4	0.001
exosc9	450057	exosome component 9	4.64E-02	1.38E-02	0.7	3.0	1.9	0.001
eif1axa	450062	eukaryotic translation initiation factor 1A, X-linked, a	1.17E-02	1.64E-03	0.7	3.0	1.9	0.001
dera	450077	2-deoxyribose-5-phosphate aldolase homolog (C. elegar	7.62E-03	4.06E-03	5.7	5.0	5.4	0.001
cx28.9	492358	connexin 28.9	1.43E-02	3.73E-02	11.4	1.5	6.4	0.001
zgc:103488	492473	zgc:103488	2.37E-02	2.51E-03	14.9	17.1	16.0	0.001
cyp2n13	492484	cytochrome P450, family 2, subfamily N, polypeptide 13	8.74E-05	9.04E-04	5.0	6.2	5.6	0.001
zgc:101897	492500	zgc:101897	2.65E-02	4.37E-02	6.0	6.6	6.3	0.001
dph5	492514	DPH5 homolog (S. cerevisiae)	2.00E-02	3.88E-02	22.9	0.9	23.9	0.001
im:7145112	492652	im:7145112	3.76E-02	2.55E-02	22.8	1.0	23.9	0.001
cyp3c1l2	492759	cytochrome P450, family 3, subfamily c, polypeptide 1 lik	2.43E-02	3.79E-02	1.1	2.4	1.7	0.001
svopl	492766	SVOP-like	1.51E-04	4.08E-02	13.5	15.7	14.6	0.001

Table S1: Zebrafish circadian oscillating genes

Gene Symbol	Gene ID	Gene Description	Fisher's g test p value (LD)	Fisher's g test p value (DD)	LD Phase	DD Phase	Phase *	FDR **
zgc:101722	494087	zgc:101722	1.65E-03	9.23E-03	11.3	13.0	12.1	0.001
nr1d1	494487	nuclear receptor subfamily 1, group d, member 1	5.74E-03	2.21E-02	22.8	0.8	23.8	0.001
zgc:92218	494495	zgc:92218	4.46E-05	4.40E-02	14.0	18.5	16.3	0.001
pcdh1gb2	503573	protocadherin 1 gamma b 2	3.74E-02	3.70E-03	15.5	15.0	15.3	0.001
upp1	503707	uridine phosphorylase 1	1.07E-02	4.31E-02	12.1	1.3	6.7	0.001
si:ch211-221p4.4	503788	si:ch211-221p4.4	9.20E-04	5.68E-03	3.0	3.8	3.4	0.001
im:7150988	504039	im:7150988	2.38E-02	3.18E-02	5.1	4.1	4.6	0.001
im:7155152	504151	im:7155152	1.34E-02	1.78E-03	15.3	15.0	15.2	0.001
zgc:112185	541326	zgc:112185	1.51E-03	3.56E-03	1.5	2.5	2.0	0.001
timm44	541380	translocase of inner mitochondrial membrane 44 homolo	2.25E-02	3.72E-02	22.1	0.8	23.5	0.001
cdc42ep2	541404	CDC42 effector protein (Rho GTPase binding) 2	2.38E-02	1.52E-02	13.2	19.2	16.2	0.001
zgc:113006	541429	zgc:113006	8.12E-04	1.39E-04	6.5	6.8	6.7	0.001
cntn1b	541474	contactin 1b	2.05E-02	1.73E-02	15.7	15.0	15.4	0.001
glb1	548343	galactosidase, beta 1	7.67E-03	1.83E-03	15.3	18.2	16.8	0.001
zgc:110712	550250	zgc:110712	1.02E-02	2.36E-02	4.3	5.0	4.7	0.001
zgc:110283	550268	zgc:110283	4.79E-03	4.13E-02	6.3	7.0	6.7	0.001
syt13	550345	synaptotagmin XIII	3.76E-03	1.14E-03	6.7	4.8	5.8	0.001
zgc:112492	550351	zgc:112492	7.73E-03	2.34E-03	16.7	17.7	17.2	0.001
srd5a2a	550398	steroid-5-alpha-reductase, alpha polypeptide 2a	6.42E-03	4.08E-03	2.6	4.0	3.3	0.001
coq10b	550442	coenzyme Q10 homolog B (S. cerevisiae)	4.34E-02	1.89E-02	3.3	4.1	3.7	0.001
mrps18b	550455	mitochondrial ribosomal protein S18B	7.93E-03	4.70E-03	22.9	0.3	23.6	0.001
hmgb3b	550466	high-mobility group box 3b	1.42E-02	2.38E-02	2.7	4.7	3.7	0.001
zgc:110200	550523	zgc:110200	3.24E-03	6.32E-04	3.5	3.3	3.4	0.001
fxc1	550527	fracture callus 1 homolog (rat)	2.01E-03	3.11E-03	0.0	0.7	0.4	0.001
gnpda1	550565	glucosamine-6-phosphate deaminase 1	3.88E-03	1.94E-02	12.0	12.8	12.4	0.001
itprip	550570	inositol 1,4,5-triphosphate receptor interacting protein	3.90E-03	1.08E-02	1.7	2.3	2.0	0.001
c1qtnf1	550573	C1q and tumor necrosis factor related protein 1	3.72E-03	2.92E-04	6.7	7.0	6.9	0.001
zgc:113106	550574	zgc:113106	3.35E-02	3.67E-02	16.5	19.7	18.1	0.001
rgra	550575	retinal G protein coupled receptor a	2.13E-03	3.39E-03	6.3	4.8	5.6	0.001
stx6	550577	syntaxin 6	4.17E-02	2.23E-02	13.5	15.0	14.3	0.001
si:dkey-171o17.2	550606	si:dkey-171o17.2	1.31E-02	1.09E-02	23.5	1.3	0.4	0.001

Table S1: Zebrafish circadian oscillating genes

Gene Symbol	Gene ID	Gene Description	Fisher's g test p value (LD)	Fisher's g test p value (DD)	LD Phase	DD Phase	Phase *	FDR **
lrrc30	552944	leucine rich repeat containing 30	8.79E-03	3.30E-03	12.7	12.8	12.8	0.001
pm20d1.1	553216	peptidase M20 domain containing 1, tandem duplicate 1	3.92E-03	1.70E-02	3.7	3.2	3.5	0.001
LOC553231	553231	hypothetical protein LOC553231	1.13E-02	4.85E-02	12.0	15.5	13.8	0.001
lrata	553239	lecithin retinol acyltransferase a (phosphatidylcholine--re	1.16E-02	4.91E-02	17.3	17.7	17.5	0.001
ca4b	553246	carbonic anhydrase IV b	5.12E-03	2.43E-02	17.8	18.5	18.2	0.001
si:ch211-196f5.1	553305	si:ch211-196f5.1	3.10E-02	4.08E-02	23.5	1.5	0.5	0.001
sec24d	553407	SEC24 family, member D (S. cerevisiae)	1.91E-03	4.37E-02	0.7	1.8	1.3	0.001
mep1a.1	553530	meprin A, alpha.1	4.90E-04	9.98E-03	1.3	2.0	1.7	0.001
cfdl	553553	complement factor D (adipsin) like	1.55E-02	1.88E-02	7.0	8.7	7.9	0.001
tp53rk	553571	TP53 regulating kinase	3.53E-02	3.68E-02	22.6	0.8	23.7	0.001
tmx3	553578	thioredoxin-related transmembrane protein 3	1.73E-02	8.76E-03	9.3	12.3	10.8	0.001
lactb	553619	lactamase, beta	7.88E-03	3.53E-02	14.5	16.7	15.6	0.001
zgc:112180	553686	zgc:112180	2.92E-02	3.46E-02	0.7	3.2	2.0	0.001
zgc:112255	553695	zgc:112255	6.25E-03	2.01E-02	5.2	3.8	4.5	0.001
zgc:112282	553701	zgc:112282	4.31E-03	8.59E-03	4.0	4.2	4.1	0.001
mrpl27	553709	mitochondrial ribosomal protein L27	1.29E-02	3.42E-02	1.7	3.0	2.4	0.001
zgc:109977	553766	zgc:109977	3.02E-02	3.02E-02	4.8	2.5	3.7	0.001
zgc:110034	553771	zgc:110034	1.15E-02	4.20E-02	0.2	1.3	0.8	0.001
gtpbp3	553807	GTP binding protein 3	3.99E-03	6.13E-03	2.8	2.7	2.8	0.001
pcdh1g18	554005	protocadherin 1 gamma 18	3.31E-02	3.29E-02	14.1	15.0	14.5	0.001
pcdh1g26	554012	protocadherin 1 gamma 26	6.92E-04	8.14E-03	14.5	14.0	14.3	0.001
pcdh2g20	554067	protocadherin 2 gamma 20	2.37E-02	5.72E-05	16.5	16.2	16.4	0.001
rpap2	554138	RNA polymerase II associated protein 2	3.24E-02	1.98E-02	19.0	20.2	19.6	0.001
rorab	554722	RAR-related orphan receptor A, paralog b	1.03E-04	3.06E-03	6.8	8.2	7.5	0.001
cry1b	554836	cryptochrome 1b	6.40E-03	1.50E-02	5.4	7.6	6.5	0.001
stk24a	555297	serine/threonine kinase 24a (STE20 homolog, yeast)	3.94E-02	2.80E-02	2.5	3.3	2.9	0.001
zgc:162780	555377	zgc:162780	2.15E-03	1.32E-03	15.3	15.7	15.5	0.001
siae	555385	sialic acid acetylesterase	1.40E-02	1.13E-02	15.8	17.3	16.5	0.001
si:ch211-242e8.1	555427	si:ch211-242e8.1	1.86E-02	2.36E-02	22.5	0.7	23.6	0.001
otud5a	555459	OTU domain containing 5a	5.55E-03	3.46E-02	15.2	15.5	15.4	0.001
LOC555631	555631	riboflavin transporter 2-like	1.70E-03	2.60E-02	4.2	5.8	5.0	0.001

Table S1: Zebrafish circadian oscillating genes

Gene Symbol	Gene ID	Gene Description	Fisher's g test p value (LD)	Fisher's g test p value (DD)	LD Phase	DD Phase	Phase *	FDR **
pcyt1bb	555725	phosphate cytidylyltransferase 1, choline, beta b	4.11E-03	1.63E-03	3.0	4.0	3.5	0.001
LOC555864	555864	retinol dehydrogenase 10-like	5.83E-06	8.51E-06	3.5	4.2	3.9	0.001
LOC556083	556083	similar to CG11909 CG11909-PA	1.41E-03	6.54E-03	2.7	2.8	2.8	0.001
si:ch211-45m15.1	556137	si:ch211-45m15.1	3.62E-03	9.45E-03	18.7	17.7	18.2	0.001
nt5dc2	556186	5'-nucleotidase domain containing 2	9.58E-04	2.41E-03	9.7	13.0	11.4	0.001
pfkfb4	556190	6-phosphofructo-2-kinase/fructose-2,6-biphosphatase 4	3.40E-05	1.77E-03	2.8	3.5	3.2	0.001
acacb	556236	acetyl-CoA carboxylase beta	1.00E-02	1.17E-02	6.3	6.7	6.5	0.001
sept4a	556486	septin 4a	3.80E-02	1.04E-02	18.8	18.2	18.5	0.001
rdh5	556528	retinol dehydrogenase 5 (11-cis/9-cis)	1.07E-02	2.33E-03	5.2	3.5	4.4	0.001
LOC556575	556575	lecithin retinol acyltransferase-like	4.58E-02	2.05E-03	5.3	0.0	2.7	0.001
LOC556578	556578	AMP deaminase 3-like	6.59E-05	6.56E-04	14.7	16.0	15.4	0.001
trub1	556642	TruB pseudouridine (psi) synthase homolog 1 (E. coli)	1.87E-02	1.88E-02	0.0	1.0	0.5	0.001
si:dkey-165a24.3	556869	si:dkey-165a24.3	9.30E-03	2.83E-02	4.5	6.2	5.4	0.001
zgc:154075	556929	zgc:154075	1.36E-03	4.05E-03	14.7	16.5	15.6	0.001
zgc:123244	556933	zgc:123244	2.61E-04	2.68E-02	1.7	2.5	2.1	0.001
si:busm1-234g15.3	557053	si:busm1-234g15.3	1.39E-04	1.45E-03	12.7	13.0	12.9	0.001
antxr2a	557239	anthrax toxin receptor 2a	1.29E-02	1.80E-02	15.2	16.8	16.0	0.001
zgc:175221	557286	zgc:175221	7.69E-03	2.59E-02	0.0	0.5	0.3	0.001
LOC557394	557394	G-protein coupled receptor 26-like	1.86E-02	1.16E-02	22.2	0.3	23.3	0.001
zgc:198329	557409	zgc:198329	1.78E-03	4.63E-02	15.5	16.3	15.9	0.001
wu:fk57d06	557454	wu:fk57d06	3.96E-04	4.78E-02	9.9	11.6	10.7	0.001
nom1	557585	nucleolar protein with MIF4G domain 1	6.57E-03	6.79E-03	0.4	2.2	1.3	0.001
si:ch211-212d10.2	557710	si:ch211-212d10.2	3.21E-03	2.21E-02	14.8	17.3	16.1	0.001
LOC557884	557884	monocarboxylate transporter 3-like	1.01E-02	3.59E-02	1.8	2.5	2.2	0.001
LOC557975	557975	hypothetical protein LOC557975	7.92E-05	4.68E-03	3.5	2.7	3.1	0.001
si:dkey-12e7.4	558132	si:dkey-12e7.4	3.97E-02	4.40E-03	13.5	16.2	14.9	0.001
ttll11	558156	tubulin tyrosine ligase-like family, member 11	3.95E-02	3.89E-02	16.8	17.3	17.1	0.001
spoplb	558170	speckle-type POZ protein-like b	1.99E-03	3.53E-02	10.0	14.5	12.3	0.001
cyp3c4	558541	cytochrome P450, family 3, subfamily C, polypeptide 4	6.80E-03	2.58E-02	0.4	2.7	1.5	0.001
soul4	558832	heme-binding protein soul4	2.92E-05	2.05E-02	13.8	16.0	14.9	0.001
LOC558876	558876	e3 ubiquitin-protein ligase RNF14-like	1.05E-04	2.45E-03	4.5	4.7	4.6	0.001

Table S1: Zebrafish circadian oscillating genes

Gene Symbol	Gene ID	Gene Description	Fisher's g test p value (LD)	Fisher's g test p value (DD)	LD Phase	DD Phase	Phase *	FDR **
dap3	559007	death associated protein 3	3.66E-02	1.67E-02	0.8	1.7	1.3	0.001
LOC559232	559232	regulator of G-protein signaling 9-binding protein-like	1.62E-03	2.19E-03	0.7	1.0	0.9	0.001
rorca	559245	RAR-related orphan receptor C a	7.92E-04	1.71E-03	9.5	12.0	10.8	0.001
cspg5b	559358	chondroitin sulfate proteoglycan 5b	1.07E-02	1.15E-02	15.5	16.3	15.9	0.001
si:ch211-103n10.4	559384	si:ch211-103n10.4	8.79E-03	3.73E-03	16.0	16.7	16.4	0.001
acaca	559403	acetyl-Coenzyme A carboxylase alpha	3.16E-03	2.60E-02	2.2	3.5	2.9	0.001
slc23a1	559435	solute carrier family 23 (nucleobase transporters), memb	2.05E-03	1.26E-02	21.9	23.0	22.4	0.001
lman1	559775	lectin, mannose-binding, 1	3.66E-02	7.52E-03	2.0	3.0	2.5	0.001
si:ch211-243g18.2	559906	si:ch211-243g18.2	4.02E-02	3.02E-02	16.2	17.8	17.0	0.001
LOC559936	559936	PI-PLC X domain-containing protein 3-like	8.30E-03	1.22E-04	11.2	12.8	12.0	0.001
LOC560023	560023	trypsin I-P1-like	1.41E-02	1.13E-02	14.3	18.5	16.4	0.001
LOC560057	560057	prostaglandin E receptor 4, subtype EP4-like	7.08E-03	4.79E-02	2.2	2.3	2.3	0.001
tprg1	560596	tumor protein p63 regulated 1	3.06E-03	4.54E-02	15.5	16.2	15.9	0.001
si:dkey-206f10.1	560719	si:dkey-206f10.1	2.42E-05	1.59E-02	0.5	0.5	0.5	0.001
zgc:153343	560896	zgc:153343	9.00E-03	2.21E-02	17.0	20.2	18.6	0.001
plch2	560919	phospholipase C, eta 2	7.53E-04	1.41E-02	15.3	15.8	15.6	0.001
dazap1	561459	DAZ associated protein 1	3.99E-03	4.10E-02	18.7	18.3	18.5	0.001
slc4a1b	561787	solute carrier family 4, anion exchanger, member 1b	1.58E-02	1.45E-02	22.3	22.7	22.5	0.001
gyg2	561803	glycogenin 2	7.33E-04	4.11E-03	15.8	17.3	16.6	0.001
opn3	561815	opsin 3 (encephalopsin, panopsin)	1.95E-03	1.35E-02	10.3	13.2	11.8	0.001
si:dkey-218h11.4	561964	si:dkey-218h11.4	1.93E-03	4.71E-02	16.5	21.5	19.0	0.001
wu:fc07b10	562019	wu:fc07b10	3.35E-03	2.93E-02	3.4	3.1	3.3	0.001
zcchc24	562028	zinc finger, CCHC domain containing 24	2.92E-02	1.17E-03	11.7	15.3	13.5	0.001
LOC562098	562098	hypothetical protein LOC562098	7.59E-03	1.99E-02	12.0	14.5	13.3	0.001
LOC562104	562104	glutaredoxin domain-containing cysteine-rich protein 1-lik	4.69E-03	2.18E-02	19.8	22.2	21.0	0.001
zgc:198195	562304	zgc:198195	1.52E-02	3.47E-03	6.5	6.6	6.5	0.001
btr02	562439	bloodthirsty-related gene family, member 2	4.50E-02	2.21E-02	3.3	3.3	3.3	0.001
si:dkey-72l14.3	562480	si:dkey-72l14.3	2.97E-02	2.56E-02	16.0	16.0	16.0	0.001
zgc:152863	562658	zgc:152863	5.34E-03	6.41E-03	12.8	15.8	14.3	0.001
LOC562752	562752	coiled-coil domain-containing protein 14-like	2.47E-02	1.33E-02	8.3	8.8	8.6	0.001
opn4x1	563080	opsin 4x1	3.25E-02	4.53E-02	10.8	12.5	11.7	0.001

Table S1: Zebrafish circadian oscillating genes

Gene Symbol	Gene ID	Gene Description	Fisher's g test p value (LD)	Fisher's g test p value (DD)	LD Phase	DD Phase	Phase *	FDR **
aqp8a.2	563130	aquaporin 8a, tandem duplicate 2	4.01E-03	1.53E-02	1.2	1.8	1.5	0.001
si:ch211-215l11.5	563180	si:ch211-215l11.5	1.66E-03	4.60E-02	4.3	3.4	3.8	0.001
grsf1	563249	G-rich RNA sequence binding factor 1	1.60E-03	2.76E-02	22.0	0.2	23.1	0.001
ubtd1	563264	ubiquitin domain containing 1	3.03E-02	1.34E-02	1.0	0.0	0.5	0.001
zgc:172079	563398	zgc:172079	1.90E-02	1.70E-02	0.5	1.3	0.9	0.001
insl3	563417	insulin-like 3 (Leydig cell)	2.55E-02	4.25E-02	0.8	0.0	0.4	0.001
LOC563472	563472	novel protein similar to vertebrate potassium channel, su	7.77E-03	1.36E-02	23.8	22.7	23.3	0.001
LOC563546	563546	ubiquitin carboxyl-terminal hydrolase 21-like	2.23E-06	2.79E-02	1.8	0.9	1.3	0.001
si:dkey-108d22.2	563551	si:dkey-108d22.2	1.59E-02	3.31E-02	14.8	17.2	16.0	0.001
LOC563744	563744	inward rectifier potassium channel 2-like	4.08E-02	2.90E-02	0.8	4.0	2.4	0.001
bhlhe41	563771	basic helix-loop-helix family, member e41	1.19E-02	4.50E-02	23.8	1.3	0.5	0.001
LOC563777	563777	similar to Proteasome-associated protein ECM29 homolo	9.09E-03	1.99E-02	17.8	19.3	18.6	0.001
si:ch211-284a13.1	564009	si:ch211-284a13.1	5.57E-04	5.86E-04	1.3	3.2	2.2	0.001
lnx2b	564464	ligand of numb-protein X 2b	4.56E-02	2.28E-03	23.5	1.8	0.7	0.001
dcaf6	564498	ddb1 and cul4 associated factor 6	1.65E-02	4.53E-03	13.7	16.8	15.3	0.001
si:ch211-132b12.7	564531	si:ch211-132b12.7	3.88E-02	2.96E-02	1.7	1.8	1.8	0.001
ildr2	564738	immunoglobulin-like domain containing receptor 2	3.68E-02	2.61E-02	11.2	15.3	13.3	0.001
slc2a11l	564849	solute carrier family 2 (facilitated glucose transporter), m	1.81E-03	5.18E-03	4.9	5.4	5.2	0.001
zgc:171765	564875	zgc:171765	1.99E-04	1.33E-02	1.5	1.7	1.6	0.001
hspa14	565232	heat shock protein 14	2.17E-02	7.01E-03	23.7	1.3	0.5	0.001
gltpd2	565337	glycolipid transfer protein domain containing 2	2.35E-02	4.86E-02	1.0	2.7	1.9	0.001
slc12a3	565439	solute carrier family 12 (sodium/chloride transporters), m	7.36E-03	3.63E-03	4.5	4.5	4.5	0.001
grma	565450	glutamate receptor, metabotropic a	6.54E-03	7.21E-03	17.0	18.8	17.9	0.001
mep1a.2	565535	meprin A, alpha.2	1.09E-03	1.26E-02	23.5	1.2	0.4	0.001
mylipb	565911	myosin regulatory light chain interacting protein b	1.65E-03	1.22E-02	3.3	4.2	3.8	0.001
ift46	565922	intraflagellar transport protein 46	4.46E-02	3.86E-03	13.6	15.8	14.7	0.001
si:ch211-124e16.1	565980	si:ch211-124e16.1	3.67E-03	4.06E-02	0.0	0.8	0.4	0.001
acss3	566019	acyl-CoA synthetase short-chain family member 3	1.47E-02	1.10E-04	7.5	7.3	7.4	0.001
grk7a	566120	G-protein-coupled receptor kinase 7a	1.39E-02	1.40E-02	2.3	0.7	1.5	0.001
LOC566188	566188	phosphatidylinositol 4,5-bisphosphate 5-phosphatase A-l	2.25E-03	2.88E-05	3.2	2.3	2.8	0.001
si:dkey-181m9.10	566384	si:dkey-181m9.10	1.29E-02	1.46E-02	14.0	15.8	14.9	0.001

Table S1: Zebrafish circadian oscillating genes

Gene Symbol	Gene ID	Gene Description	Fisher's g test p value (LD)	Fisher's g test p value (DD)	LD Phase	DD Phase	Phase *	FDR **
slc16a10	566499	solute carrier family 16, member 10 (aromatic amino acic	3.31E-03	4.23E-02	21.8	23.7	22.8	0.001
wu:fk66f10	566607	wu:fk66f10	1.31E-02	1.32E-02	5.0	5.8	5.4	0.001
si:ch73-289a15.2	566775	si:ch73-289a15.2	1.90E-02	2.61E-02	13.8	14.8	14.3	0.001
mmp14b	566945	matrix metalloproteinase 14b (membrane-inserted)	6.94E-03	1.29E-02	14.5	16.5	15.5	0.001
rrbp1	567029	ribosome binding protein 1 homolog (dog)	1.83E-03	1.94E-02	4.5	4.7	4.6	0.001
zgc:152698	567084	zgc:152698	3.77E-02	1.45E-02	19.2	20.3	19.8	0.001
si:dkey-189l3.1	567200	si:dkey-189l3.1	2.67E-02	3.71E-02	22.3	23.0	22.7	0.001
LOC567242	567242	similar to cyclic nucleotide gated channel alpha 3	1.18E-03	2.39E-02	23.2	21.7	22.5	0.001
LOC567594	567594	uncharacterized protein C7orf50 homolog	2.91E-02	1.13E-02	0.2	2.7	1.5	0.001
slc12a7b	567667	solute carrier family 12, member 7b	1.36E-02	9.52E-03	4.0	3.0	3.5	0.001
mrpl37	567819	mitochondrial ribosomal protein L37	1.25E-02	4.56E-02	0.8	2.2	1.5	0.001
zgc:123246	567956	zgc:123246	3.49E-03	3.08E-02	13.0	15.3	14.2	0.001
si:dkey-222b8.4	568013	si:dkey-222b8.4	1.89E-02	4.24E-02	22.7	22.8	22.7	0.001
cacna2d4a	568225	calcium channel, voltage-dependent, alpha 2/delta subur	1.70E-02	3.84E-02	16.0	15.5	15.8	0.001
zgc:162232	568256	zgc:162232	9.16E-03	4.77E-02	10.5	14.7	12.6	0.001
ankrd33ba	568270	ankyrin repeat domain 33ba	3.29E-03	4.96E-05	12.8	14.0	13.4	0.001
si:dkey-22l11.5	568328	si:dkey-22l11.5	1.67E-02	3.92E-02	23.0	23.5	23.3	0.001
LOC568355	568355	c-type lectin domain family 11 member A-like	7.99E-04	4.82E-02	4.3	4.5	4.4	0.001
crb2b	568520	crumbs homolog 2b	4.61E-03	1.71E-02	16.8	16.2	16.5	0.001
rbm45	568524	RNA binding motif protein 45	1.87E-02	4.93E-02	17.7	18.5	18.1	0.001
si:dkey-3n7.3	568579	si:dkey-3n7.3	4.62E-03	3.99E-03	14.3	16.0	15.2	0.001
zgc:161979	568707	zgc:161979	2.16E-03	6.58E-03	2.3	4.3	3.3	0.001
si:dkey-177p2.6	568712	si:dkey-177p2.6	2.92E-02	1.10E-02	0.5	0.0	0.3	0.001
si:ch211-154o6.7	568750	si:ch211-154o6.7	7.62E-04	6.56E-03	6.5	4.7	5.6	0.001
nyx	568821	nyctalopin	6.42E-03	4.47E-03	0.3	1.7	1.0	0.001
LOC568833	568833	claudin-3-like	9.99E-04	6.84E-03	3.2	3.7	3.5	0.001
gabrr1	568984	gamma-aminobutyric acid (GABA) receptor, rho 1	1.87E-06	4.10E-03	3.2	3.5	3.4	0.001
LOC569014	569014	gamma-glutamyltranspeptidase 1-like	8.05E-04	4.82E-03	0.2	1.3	0.8	0.001
LOC569085	569085	thiamine transporter 1-like	4.95E-03	4.98E-02	1.7	23.7	0.7	0.001
zgc:174379	569379	zgc:174379	2.82E-02	3.36E-03	22.8	2.7	0.8	0.001
lars	569778	leucyl-tRNA synthetase	3.76E-02	1.00E-03	1.1	3.6	2.4	0.001

Table S1: Zebrafish circadian oscillating genes

Gene Symbol	Gene ID	Gene Description	Fisher's g test p value (LD)	Fisher's g test p value (DD)	LD Phase	DD Phase	Phase *	FDR **
tspan3b	569864	tetraspanin 3b	1.62E-02	1.04E-02	13.8	15.3	14.6	0.001
zgc:153896	569930	zgc:153896	2.77E-02	1.09E-02	0.3	2.2	1.3	0.001
LOC569958	569958	protein S100-A5-like	3.45E-05	3.23E-03	6.8	7.0	6.9	0.001
si:dkey-7l6.7	569986	si:dkey-7l6.7	7.66E-03	2.28E-02	14.5	14.3	14.4	0.001
si:dkey-183n20.15	570411	si:dkey-183n20.15	2.62E-04	1.76E-02	15.5	15.8	15.7	0.001
aox1	570457	aldehyde oxidase 1	3.02E-02	4.81E-02	12.8	16.8	14.8	0.001
LOC570552	570552	novel asparaginase protein	1.58E-02	1.87E-03	4.3	4.8	4.6	0.001
LOC570693	570693	BTB/POZ domain-containing protein KCTD20-like	1.55E-03	4.24E-03	14.2	15.3	14.8	0.001
zgc:194845	570876	zgc:194845	3.24E-02	4.83E-03	14.8	15.8	15.3	0.001
arid3b	571249	AT rich interactive domain 3B (Bright like)	8.88E-04	3.14E-02	14.4	15.4	14.9	0.001
LOC571722	571722	lipid phosphate phosphatase-related protein type 4-like	2.23E-03	3.95E-03	13.7	14.7	14.2	0.001
si:ch211-14c7.2	571950	si:ch211-14c7.2	1.25E-02	1.30E-02	13.5	11.6	12.6	0.001
ido1	571967	indoleamine 2,3-dioxygenase 1	1.32E-02	1.07E-02	4.1	4.5	4.3	0.001
mrpl9	572170	mitochondrial ribosomal protein L9	1.89E-03	2.50E-02	0.0	2.2	1.1	0.001
tekt3	572368	tektin 3	2.95E-02	2.80E-02	14.7	16.7	15.7	0.001
cry2a	573209	cryptochrome 2a	1.31E-03	6.05E-04	12.3	14.3	13.3	0.001
xpnpep3	573360	X-prolyl aminopeptidase (aminopeptidase P) 3, putative	3.28E-02	4.48E-02	1.3	1.7	1.5	0.001
zgc:114108	573996	zgc:114108	7.79E-03	8.46E-03	3.6	4.4	4.0	0.001
zgc:113984	573997	zgc:113984	4.38E-03	1.94E-02	16.6	17.6	17.1	0.001
zgc:110409	574009	zgc:110409	4.93E-03	2.91E-02	21.7	23.5	22.6	0.001
vgll2b	606659	vestigial like 2b	3.57E-02	2.85E-02	17.8	20.0	18.9	0.001
aqp9a	606660	aquaporin 9a	4.09E-04	1.74E-02	22.8	0.5	23.7	0.001
dkc1	613144	dyskeratosis congenita 1, dyskerin	9.08E-03	2.76E-02	22.8	1.5	0.2	0.001
zgc:113338	619262	zgc:113338	3.06E-02	4.88E-02	13.7	14.3	14.0	0.001
sagb	619268	S-antigen; retina and pineal gland (arrestin) b	1.45E-04	9.48E-03	6.0	7.2	6.6	0.001
zgc:123297	641563	zgc:123297	1.26E-02	1.20E-02	17.2	17.7	17.5	0.001
figf	678512	c-fos induced growth factor	5.82E-03	1.83E-02	2.5	4.5	3.5	0.001
mmp30	678531	matrix metallopeptidase 30	2.85E-03	4.41E-02	7.3	8.3	7.8	0.001
xkr9	678537	XK, Kell blood group complex subunit-related family, mei	2.87E-03	1.75E-03	3.0	3.5	3.3	0.001
ndrg4	692305	N-myc downstream regulated gene 4	5.69E-03	1.45E-03	14.8	15.9	15.3	0.001
fam102ba	724011	family with sequence similarity 102, member B, a	2.84E-03	4.44E-02	3.0	3.5	3.3	0.001

Table S1: Zebrafish circadian oscillating genes

Gene Symbol	Gene ID	Gene Description	Fisher's g test p value (LD)	Fisher's g test p value (DD)	LD Phase	DD Phase	Phase *	FDR **
zgc:153408	751633	zgc:153408	1.49E-02	2.99E-02	2.3	2.0	2.2	0.001
zgc:153417	751659	zgc:153417	1.69E-04	4.80E-03	2.5	2.5	2.5	0.001
zgc:153443	751686	zgc:153443	8.88E-06	6.39E-03	0.3	22.0	23.2	0.001
zgc:153154	751701	zgc:153154	9.61E-03	4.89E-02	7.7	5.6	6.6	0.001
ms4a17a.16	751754	membrane-spanning 4-domains, subfamily A, member 1	3.57E-02	1.84E-03	18.2	18.0	18.1	0.001
med19a	767651	mediator complex subunit 19a	3.36E-02	3.27E-02	14.5	17.8	16.2	0.001
zgc:153394	767653	zgc:153394	1.05E-02	1.29E-02	3.4	1.3	2.3	0.001
zgc:153018	767701	zgc:153018	2.41E-02	7.38E-03	8.5	8.2	8.4	0.001
ugt2a5	767705	UDP glucuronosyltransferase 2 family, polypeptide A5	1.10E-03	4.37E-02	15.0	16.8	15.9	0.001
si:ch211-195b13.1	768159	si:ch211-195b13.1	1.16E-04	1.28E-02	5.7	3.3	4.5	0.001
cyp2x9	768288	cytochrome P450, family 2, subfamily X, polypeptide 9	2.20E-02	2.90E-02	3.8	4.5	4.2	0.001
zgc:153629	768289	zgc:153629	3.47E-03	2.05E-02	5.8	6.2	6.0	0.001
zgc:153914	777612	zgc:153914	1.54E-03	7.32E-03	3.7	3.3	3.5	0.001
zgc:153521	777620	zgc:153521	2.50E-02	3.53E-02	13.8	15.5	14.7	0.001
zgc:154093	777623	zgc:154093	1.99E-05	2.96E-02	3.8	3.7	3.8	0.001
zgc:153892	777708	zgc:153892	7.32E-03	4.40E-03	6.0	5.5	5.8	0.001
papss2a	777719	3'-phosphoadenosine 5'-phosphosulfate synthase 2a	9.91E-04	2.72E-02	2.8	4.3	3.6	0.001
ubc	777766	ubiquitin C	4.35E-03	4.69E-02	15.0	16.5	15.8	0.001
zgc:158644	791165	zgc:158644	6.45E-04	3.84E-02	14.0	15.0	14.5	0.001
zgc:110847	791612	zgc:110847	7.17E-03	1.04E-02	3.0	3.2	3.1	0.001
sult1st2	791732	sulfotransferase family 1, cytosolic sulfotransferase 2	4.84E-05	8.98E-04	5.0	6.2	5.6	0.001
pdxka	791775	pyridoxal (pyridoxine, vitamin B6) kinase a	1.81E-03	2.95E-02	6.2	6.5	6.4	0.001
zgc:114201	791783	zgc:114201	8.00E-03	2.76E-03	4.3	6.0	5.1	0.001
ndrg1	792085	N-myc downstream regulated gene 1	5.42E-03	6.02E-03	15.3	15.2	15.2	0.001
zgc:110259	792229	zgc:110259	2.35E-05	2.70E-02	14.2	16.2	15.2	0.001
fa2h	792257	fatty acid 2-hydroxylase	4.09E-02	3.24E-02	21.7	23.8	22.8	0.001
c21orf59l	792532	c21orf59 homolog (H. sapiens), like	2.77E-03	9.39E-03	3.0	3.0	3.0	0.001
zgc:162331	793007	zgc:162331	8.13E-04	2.19E-02	2.3	2.8	2.6	0.001
sb:cb1035	793418	sb:cb1035	1.88E-04	4.07E-04	16.3	16.8	16.6	0.001
LOC795535	795535	hypothetical protein LOC795535	4.48E-03	1.54E-02	6.7	0.2	3.5	0.001
treh	795901	trehalase (brush-border membrane glycoprotein)	1.72E-02	1.38E-02	7.2	7.5	7.4	0.001

Table S1: Zebrafish circadian oscillating genes

Gene Symbol	Gene ID	Gene Description	Fisher's g test p value (LD)	Fisher's g test p value (DD)	LD Phase	DD Phase	Phase *	FDR **
si:dkey-167i21.2	796588	si:dkey-167i21.2	3.58E-04	4.86E-03	21.2	20.7	21.0	0.001
arr3b	796867	arrestin 3b, retinal (X-arrestin)	3.42E-02	2.55E-02	3.3	1.5	2.4	0.001
LOC798349	798349	similar to UDP-Gal:betaGlcNAc beta 1,3-galactosyltransf	6.62E-03	3.05E-02	2.7	4.0	3.4	0.001
abcb5	798527	ATP-binding cassette, sub-family B (MDR/TAP), membe	4.47E-05	2.52E-02	4.8	6.2	5.5	0.001
si:ch211-240l19.7	799219	si:ch211-240l19.7	4.03E-02	9.07E-03	13.5	15.3	14.4	0.001
LOC799768	799768	similar to arrestin domain containing 3	3.25E-02	3.58E-02	5.3	2.0	3.7	0.001
zgc:162180	799770	zgc:162180	6.61E-04	6.11E-03	4.2	5.0	4.6	0.001
cyp2ad6	799957	cytochrome P450, family 2, subfamily AD, polypeptide 6	3.57E-03	1.14E-04	5.2	6.0	5.6	0.001
zgc:162946	100000104	zgc:162946	2.31E-02	1.44E-02	13.8	15.3	14.6	0.001
LOC100000596	100000596	hypothetical protein LOC100000596	2.97E-03	2.89E-02	8.0	5.0	6.5	0.001
ngb	100000711	neuroglobin	5.46E-03	3.14E-02	4.0	8.3	6.2	0.001
snx33	100001421	sorting nexin 33	6.87E-04	7.81E-03	12.3	15.5	13.9	0.001
im:7136021	100002065	im:7136021	4.84E-04	2.97E-02	21.3	22.5	21.9	0.001
LOC100002893	100002893	zinc transporter ZIP8-like	1.72E-02	1.31E-02	16.0	17.0	16.5	0.001
zgc:165597	100002948	zgc:165597	6.82E-03	1.67E-02	0.8	0.7	0.8	0.001
zgc:123180	100003313	zgc:123180	3.86E-02	4.49E-03	0.5	1.8	1.2	0.001
nhp2	100003787	NHP2 ribonucleoprotein homolog (yeast)	9.78E-03	1.82E-02	0.0	2.2	1.1	0.001
zgc:194629	100004339	zgc:194629	9.52E-03	4.99E-02	2.8	3.0	2.9	0.001
LOC100005130	100005130	calcium homeostasis modulator protein 3-like	4.08E-04	4.38E-03	3.7	3.8	3.8	0.001
mettl2a	100006618	methyltransferase like 2A	1.55E-02	3.57E-02	22.3	0.0	23.2	0.001
tdo2a	100008541	tryptophan 2,3-dioxygenase a	6.71E-03	6.15E-03	2.2	3.5	2.9	0.001
slc43a1b	100009631	solute carrier family 43, member 1b	3.76E-03	2.56E-03	23.2	0.5	23.8	0.001
sgpl1	100037312	sphingosine-1-phosphate lyase 1	2.04E-04	2.87E-03	4.7	5.6	5.1	0.001
zgc:162213	100037318	zgc:162213	2.91E-03	3.55E-02	12.8	15.0	13.9	0.001
mlycd	100037349	malonyl-CoA decarboxylase	1.01E-02	4.90E-02	7.7	8.5	8.1	0.001
zgc:165502	100073336	zgc:165502	2.15E-03	2.09E-03	2.5	3.2	2.9	0.001
zgc:165423	100101646	zgc:165423	1.87E-04	2.78E-03	3.7	4.8	4.3	0.001
zgc:171603	100141349	zgc:171603	3.55E-03	1.35E-02	3.7	4.8	4.3	0.001
mrpl22	100142645	mitochondrial ribosomal protein L22	3.80E-03	2.23E-02	23.3	0.3	23.8	0.001
LOC100147862	100147862	hydrolethalus syndrome protein 1 homolog	1.27E-02	3.90E-02	13.0	14.5	13.8	0.001
zgc:55418	100149890	zgc:55418	2.07E-03	1.74E-02	3.1	4.1	3.6	0.001

Table S1: Zebrafish circadian oscillating genes

Gene Symbol	Gene ID	Gene Description	Fisher's g test p value (LD)	Fisher's g test p value (DD)	LD Phase	DD Phase	Phase *	FDR **
si:ch211-191a24.4	100150331	si:ch211-191a24.4	2.55E-02	6.31E-03	9.7	9.2	9.5	0.001
si:ch211-160b11.4	100150558	si:ch211-160b11.4	1.07E-03	5.89E-03	17.7	18.5	18.1	0.001
map1aa	100151220	microtubule-associated protein 1Aa	2.26E-02	9.57E-03	14.5	13.3	13.9	0.001
LOC100151479	100151479	urea transporter 2-like	1.59E-02	2.06E-02	1.2	1.8	1.5	0.001
slc1a7a	100170783	solute carrier family 1 (glutamate transporter), member 7	7.54E-03	1.03E-02	14.3	15.7	15.0	0.001
si:dkey-177p2.5	100270730	si:dkey-177p2.5	7.40E-03	1.69E-02	0.5	0.6	0.6	0.001
im:7165255	100318736	im:7165255	1.19E-04	5.42E-03	14.2	15.5	14.9	0.001
LOC100329330	100329330	ral guanine nucleotide dissociation stimulator-like 1-like	1.18E-02	1.74E-04	13.0	14.2	13.6	0.001
LOC100332005	100332005	hypothetical protein LOC100332005	2.54E-03	3.15E-03	4.3	3.7	4.0	0.001
LOC100332503	100332503	UPF0640 protein C3orf78 homolog	3.79E-03	8.52E-03	1.5	1.7	1.6	0.001
LOC100333680	100333680	hypothetical protein LOC100333680	5.96E-03	2.91E-02	0.7	3.3	2.0	0.001
LOC100333777	100333777	hydroxysteroid 11-beta-dehydrogenase 1-like protein-like	9.48E-03	2.44E-02	18.3	19.5	18.9	0.001
LOC100334711	100334711	cyclic nucleotide-gated cation channel alpha-3-like	1.28E-02	2.27E-02	1.8	23.2	0.5	0.001
cat	30068	catalase	4.73E-02	8.26E-02	11.7	13.3	12.5	0.003
nccrp1	30078	nonspecific cytotoxic cell receptor protein 1	3.09E-03	9.88E-02	4.2	3.8	4.0	0.003
clock	30140	clock	9.01E-02	2.31E-03	14.0	16.2	15.1	0.003
hoxb1a	30337	homeo box B1a	7.97E-02	9.39E-02	15.2	17.8	16.5	0.003
dlx3b	30585	distal-less homeobox gene 3b	5.69E-02	2.40E-02	17.8	21.0	19.4	0.003
raraa	30680	retinoic acid receptor, alpha a	7.26E-02	6.53E-02	11.7	12.7	12.2	0.003
hccs	58016	holocytochrome c synthase	2.04E-04	7.22E-02	2.0	0.3	1.2	0.003
smad3a	58092	MAD homolog 3a (Drosophila)	9.36E-04	7.24E-02	4.7	4.3	4.5	0.003
htatip2	64605	HIV-1 Tat interactive protein 2	3.04E-02	8.38E-02	8.5	8.5	8.5	0.003
rnd3a	80963	Rho family GTPase 3a	1.28E-02	6.68E-02	16.2	17.7	17.0	0.003
meis3	81885	myeloid ecotropic viral integration site 3	9.86E-02	3.22E-02	13.2	13.5	13.4	0.003
glrba	83412	gylcine receptor, beta a	1.40E-02	6.08E-02	3.0	3.2	3.1	0.003
psma6b	83917	proteasome (prosome, macropain) subunit, alpha type, 6	6.84E-03	6.90E-02	19.3	20.3	19.8	0.003
gc3	140426	guanylyl cyclase 3	8.31E-03	5.26E-02	0.5	23.7	0.1	0.003
xbp1	140614	X-box binding protein 1	2.25E-02	6.00E-02	2.2	3.7	3.0	0.003
ckbb	140744	creatine kinase, brain b	7.33E-03	9.61E-02	3.0	3.4	3.2	0.003
rp42-pen	192303	rp42 homolog (pending)	3.04E-02	5.58E-02	12.7	15.3	14.0	0.003
onecutl	246225	one cut domain, family member, like	5.27E-02	7.58E-02	18.7	17.3	18.0	0.003

Table S1: Zebrafish circadian oscillating genes

Gene Symbol	Gene ID	Gene Description	Fisher's g test p value (LD)	Fisher's g test p value (DD)	LD Phase	DD Phase	Phase *	FDR **
p2rx4a	259258	purinergic receptor P2X, ligand-gated ion channel, 4a	1.31E-03	9.66E-02	18.3	19.5	18.9	0.003
id2a	266599	inhibitor of DNA binding 2, dominant negative helix-loop-	6.43E-02	4.70E-02	16.8	17.9	17.3	0.003
aldh3d1	282559	aldehyde dehydrogenase 3 family, member D1	8.91E-02	3.40E-02	0.8	3.8	2.3	0.003
timm9	282678	translocase of inner mitochondrial membrane 9 homolog	4.84E-03	6.99E-02	0.2	0.5	0.4	0.003
impdh2	317745	IMP (inosine monophosphate) dehydrogenase 2	7.34E-02	7.77E-02	22.2	0.2	23.2	0.003
nhp2l1	321114	NHP2 non-histone chromosome protein 2-like 1 (S. cerev	1.68E-02	5.17E-02	0.3	2.7	1.5	0.003
si:dkey-91i17.1	321130	si:dkey-91i17.1	7.65E-02	9.72E-02	23.2	1.5	0.4	0.003
ssb	321504	Sjogren syndrome antigen B (autoantigen La)	3.50E-02	9.78E-02	23.1	1.7	0.4	0.003
psmc6	321585	proteasome (prosome, macropain) 26S subunit, ATPase	5.79E-02	5.04E-03	18.2	20.0	19.1	0.003
wdr36	321745	WD repeat domain 36	1.70E-02	6.11E-02	0.8	2.0	1.4	0.003
tgif1	321756	TGFB-induced factor homeobox 1	6.17E-02	4.75E-02	12.3	15.0	13.7	0.003
setd6	322348	SET domain containing 6	8.27E-02	4.92E-02	1.5	4.0	2.8	0.003
wu:fb62f08	322487	wu:fb62f08	1.93E-02	5.31E-02	22.5	0.7	23.6	0.003
dnaja2l	324164	DnaJ (Hsp40) homolog, subfamily A, member 2, like	5.54E-02	1.22E-02	16.8	17.5	17.2	0.003
si:ch73-73d17.3	324212	si:ch73-73d17.3	3.20E-04	9.03E-02	5.3	5.3	5.3	0.003
mgea5	324487	meningioma expressed antigen 5 (hyaluronidase)	7.71E-03	6.85E-02	14.2	15.8	15.0	0.003
pex19	324778	peroxisomal biogenesis factor 19	2.20E-02	5.59E-02	19.2	22.5	20.9	0.003
zgc:55512	325470	zgc:55512	3.86E-02	9.05E-02	12.7	16.5	14.6	0.003
cnp	325896	2',3'-cyclic nucleotide 3' phosphodiesterase	6.58E-02	1.91E-03	1.0	0.3	0.7	0.003
gabarapa	326974	GABA(A) receptor-associated protein a	7.84E-02	5.95E-02	12.2	16.3	14.3	0.003
larp1	327175	La ribonucleoprotein domain family, member 1	1.87E-03	6.97E-02	23.2	23.7	23.5	0.003
sec23b	327268	Sec23 homolog B (S. cerevisiae)	7.01E-02	3.77E-02	1.7	2.3	2.0	0.003
slc9a3r1	327272	solute carrier family 9 (sodium/hydrogen exchanger), me	3.48E-02	6.26E-02	22.7	1.5	0.1	0.003
f5	327320	coagulation factor V	7.27E-02	6.14E-02	0.4	0.7	0.6	0.003
hsd17b12a	327417	hydroxysteroid (17-beta) dehydrogenase 12a	1.70E-02	5.29E-02	23.5	0.3	23.9	0.003
micall2b	327592	mical-like 2b	2.40E-04	9.71E-02	15.5	18.2	16.9	0.003
hsd11b2	334098	hydroxysteroid 11-beta dehydrogenase 2	5.69E-02	3.40E-04	13.7	16.3	15.0	0.003
mat1a	334195	methionine adenosyltransferase I, alpha	7.24E-02	1.31E-02	2.5	3.5	3.0	0.003
osbpl2	334206	oxysterol binding protein-like 2	5.59E-02	3.61E-02	2.2	3.2	2.7	0.003
agk	334270	acylglycerol kinase	3.98E-03	7.84E-02	1.2	2.2	1.7	0.003
pls1	334273	plastin 1 (I isoform)	1.40E-02	8.98E-02	20.3	22.9	21.6	0.003

Table S1: Zebrafish circadian oscillating genes

Gene Symbol	Gene ID	Gene Description	Fisher's g test p value (LD)	Fisher's g test p value (DD)	LD Phase	DD Phase	Phase *	FDR **
si:dkey-52h23.1	335737	si:dkey-52h23.1	6.96E-02	9.20E-04	12.0	14.3	13.1	0.003
utp11l	336149	UTP11-like, U3 small nucleolar ribonucleoprotein (yeast)	8.40E-02	5.07E-02	22.8	0.5	23.7	0.003
wu:fj53d07	336170	wu:fj53d07	5.55E-02	9.72E-04	17.5	17.5	17.5	0.003
wu:fb12g12	336532	wu:fb12g12	3.84E-03	7.03E-02	19.8	21.0	20.4	0.003
zgc:77151	337153	zgc:77151	6.10E-02	1.90E-02	13.0	13.3	13.2	0.003
sepx1a	352916	selenoprotein X, 1a	1.24E-03	6.81E-02	8.0	6.5	7.3	0.003
txnrd1	352924	thioredoxin reductase 1	5.89E-02	1.90E-02	4.7	5.2	5.0	0.003
anxa4	353362	annexin A4	1.30E-02	7.62E-02	4.3	4.2	4.2	0.003
gulp1	368404	GULP, engulfment adaptor PTB domain containing 1	2.23E-02	6.70E-02	13.8	15.2	14.5	0.003
snx9	368424	sorting nexin 9	1.28E-02	6.12E-02	11.5	14.2	12.9	0.003
wdr75	368478	WD repeat domain 75	5.57E-02	9.75E-02	21.8	11.4	16.6	0.003
si:busm1-57f23.1	368621	si:busm1-57f23.1	2.61E-02	5.81E-02	12.3	9.3	10.8	0.003
ctsc	368704	cathepsin C	3.52E-03	8.75E-02	15.3	17.8	16.6	0.003
hspa9	373085	heat shock protein 9	2.59E-03	5.58E-02	2.3	2.3	2.3	0.003
cdh17	378452	cadherin 17, LI cadherin (liver-intestine)	6.23E-02	8.38E-02	0.8	3.8	2.3	0.003
slmo2	378855	slowmo homolog 2 (Drosophila)	4.16E-04	6.88E-02	2.8	2.8	2.8	0.003
hopx	386589	HOP homeobox	1.72E-02	7.07E-02	5.2	6.0	5.6	0.003
fam107b	386641	family with sequence similarity 107, member B	9.65E-02	8.62E-02	4.6	3.3	4.0	0.003
mycb	393141	myelocytomatosis oncogene b	2.42E-02	8.41E-02	2.8	5.2	4.0	0.003
zgc:56072	393159	zgc:56072	7.08E-02	8.83E-02	10.5	18.8	14.7	0.003
fxr2	393180	fragile X mental retardation, autosomal homolog 2	1.07E-02	8.84E-02	2.7	3.8	3.3	0.003
zgc:55343	393222	zgc:55343	5.82E-02	2.57E-02	1.3	2.4	1.8	0.003
imp3	393237	IMP3, U3 small nucleolar ribonucleoprotein, homolog (ye	5.13E-03	6.00E-02	0.5	2.7	1.6	0.003
zgc:56565	393268	zgc:56565	1.02E-02	6.46E-02	4.4	3.6	4.0	0.003
slc22a7b	393320	solute carrier family 22 (organic anion transporter), meml	2.14E-03	5.32E-02	4.2	5.7	5.0	0.003
zgc:63675	393369	zgc:63675	8.69E-02	3.30E-02	13.5	14.5	14.0	0.003
pdcd2	393370	programmed cell death 2	5.90E-02	2.15E-02	22.8	23.3	23.1	0.003
prmt7	393475	protein arginine N-methyltransferase 7	4.11E-02	7.21E-02	23.3	1.2	0.3	0.003
cyp2k18	393484	cytochrome P450, family 2, subfamily K, polypeptide 18	3.79E-02	6.85E-02	11.5	12.8	12.2	0.003
zgc:66337	393492	zgc:66337	8.55E-03	5.57E-02	4.2	3.5	3.9	0.003
mfsd4a	393503	major facilitator superfamily domain containing 4a	9.22E-02	6.85E-02	15.8	16.8	16.3	0.003

Table S1: Zebrafish circadian oscillating genes

Gene Symbol	Gene ID	Gene Description	Fisher's g test p value (LD)	Fisher's g test p value (DD)	LD Phase	DD Phase	Phase *	FDR **
psmd2	393518	proteasome (prosome, macropain) 26S subunit, non-ATI	6.61E-02	3.27E-02	17.8	19.0	18.4	0.003
tbl1xr1	393581	transducin (beta)-like 1 X-linked receptor 1	8.22E-02	8.23E-02	21.0	20.8	20.9	0.003
fam84a	393590	family with sequence similarity 84, member A	5.47E-02	5.21E-02	13.8	13.7	13.8	0.003
phactr4b	393643	phosphatase and actin regulator 4b	1.58E-03	6.56E-02	3.2	7.7	5.5	0.003
ndrg1l	393665	N-myc downstream regulated gene 1, like	7.20E-02	6.59E-02	14.0	14.7	14.3	0.003
gnb5b	393719	guanine nucleotide binding protein (G protein), beta 5b	1.29E-02	8.84E-02	1.3	1.7	1.5	0.003
cox4i2	393776	cytochrome c oxidase subunit IV isoform 2	6.55E-02	6.13E-02	15.0	15.5	15.3	0.003
kcnip3l	393792	Kv channel interacting protein 3, calsenilin, like	3.63E-03	5.18E-02	2.7	3.6	3.1	0.003
c8g	393837	complement component 8, gamma polypeptide	1.48E-02	5.26E-02	16.2	16.7	16.5	0.003
arhgap12	393848	Rho GTPase activating protein 12	3.81E-02	7.79E-02	2.5	4.5	3.5	0.003
myoz2a	393873	myozenin 2a	6.59E-02	5.53E-03	5.8	6.3	6.1	0.003
pdcd6	393925	programmed cell death 6	1.73E-04	6.90E-02	5.2	5.2	5.2	0.003
nola1	393950	nucleolar protein family A, member 1 (H/ACA small nucle	7.26E-02	9.95E-02	23.3	2.7	1.0	0.003
surf6	394040	surfeit 6	5.15E-02	5.17E-02	23.8	2.3	1.1	0.003
qtrtd1	402798	queuine tRNA-ribosyltransferase domain containing 1	8.54E-02	7.30E-02	23.5	1.9	0.7	0.003
zgc:77391	402930	zgc:77391	7.94E-03	5.64E-02	13.8	16.2	15.0	0.003
pnp6	402953	purine nucleoside phosphorylase 6	3.41E-03	6.86E-02	2.3	3.2	2.8	0.003
ap1m2	403021	adaptor-related protein complex 1, mu 2 subunit	5.60E-02	3.90E-03	22.3	23.8	23.1	0.003
cx30.3	404725	connexin 30.3	8.38E-02	5.32E-02	2.2	4.7	3.4	0.003
cdx1a	405762	caudal type homeo box transcription factor 1 a	5.01E-02	9.06E-02	16.3	15.3	15.8	0.003
nfe2l1	405781	nuclear factor, erythroid derived 2,-like 1	1.22E-02	8.37E-02	9.1	10.0	9.5	0.003
zgc:77001	405847	zgc:77001	6.75E-02	3.63E-02	14.8	16.0	15.4	0.003
tomm20a	406309	translocase of outer mitochondrial membrane 20 homolo	1.30E-02	8.31E-02	23.7	1.6	0.7	0.003
zfand5a	406312	zinc finger, AN1-type domain 5a	7.05E-02	8.07E-02	19.1	21.5	20.3	0.003
chia.1	406323	chitinase, acidic.1	1.61E-02	6.08E-02	5.8	4.3	5.1	0.003
thap11	406390	THAP domain containing 11	1.66E-02	5.28E-02	14.3	16.0	15.2	0.003
prrx1a	406431	paired related homeobox 1a	4.09E-03	5.25E-02	15.7	19.2	17.5	0.003
slc25a33	406436	solute carrier family 25, member 33	6.02E-02	2.77E-02	3.7	2.5	3.1	0.003
psma8	406445	proteasome (prosome, macropain) subunit, alpha type, 8	1.49E-02	9.26E-02	17.7	20.2	19.0	0.003
slc20a1a	406458	solute carrier family 20, member 1a	7.18E-04	5.74E-02	21.5	0.8	23.2	0.003
zgc:77836	406477	zgc:77836	2.11E-02	7.55E-02	21.7	23.5	22.6	0.003

Table S1: Zebrafish circadian oscillating genes

Gene Symbol	Gene ID	Gene Description	Fisher's g test p value (LD)	Fisher's g test p value (DD)	LD Phase	DD Phase	Phase *	FDR **
zgc:85829	406561	zgc:85829	7.41E-02	1.94E-02	0.2	1.5	0.9	0.003
rsrc2	406566	arginine/serine-rich coiled-coil 2	5.00E-02	1.19E-02	16.8	18.3	17.6	0.003
atp5j	406599	ATP synthase, H+ transporting, mitochondrial F0 comple	9.34E-02	8.10E-04	1.2	4.2	2.7	0.003
dcun1d5	406622	DCN1, defective in cullin neddylation 1, domain containir	7.39E-03	8.91E-02	17.0	19.2	18.1	0.003
cox4nb	406679	cox4 neighbor	6.57E-02	1.41E-02	21.3	0.0	22.7	0.003
fpgs	406746	folylpolyglutamate synthase	2.88E-03	6.71E-02	1.9	2.3	2.1	0.003
ddt	415237	D-dopachrome tautomerase	5.02E-02	4.75E-02	6.9	6.3	6.6	0.003
impdh1a	431724	inosine 5'-phosphate dehydrogenase 1a	1.11E-02	8.92E-02	1.2	2.3	1.8	0.003
zgc:77060	431765	zgc:77060	2.34E-02	7.80E-02	5.8	6.0	5.9	0.003
rbm34	436655	RNA binding motif protein 34	9.06E-02	5.98E-03	22.5	1.5	0.0	0.003
epdr1	436689	ependymin related protein 1	5.82E-02	2.52E-02	3.0	4.8	3.9	0.003
gtpbp10	436727	GTP-binding protein 10 (putative)	1.11E-02	6.74E-02	0.2	2.7	1.5	0.003
ldhbb	436747	lactate dehydrogenase Bb	5.05E-02	1.80E-02	16.2	17.0	16.6	0.003
fam203a	436795	family with sequence similarity 203, member A	3.89E-02	5.77E-02	23.0	1.2	0.1	0.003
nop16	436807	NOP16 nucleolar protein homolog (yeast)	5.42E-02	2.76E-03	23.7	2.0	0.9	0.003
nup37	436892	nucleoporin 37	2.27E-02	9.59E-02	5.0	6.0	5.5	0.003
blvrb	436959	biliverdin reductase B (flavin reductase (NADPH))	1.55E-03	5.86E-02	6.7	6.7	6.7	0.003
ophn1	436982	oligophrenin 1	3.84E-02	9.09E-02	13.3	11.3	12.3	0.003
imp4	436992	IMP4, U3 small nucleolar ribonucleoprotein, homolog (ye	7.14E-02	3.54E-02	0.0	1.8	0.9	0.003
dimt1l	445162	DIM1 dimethyladenosine transferase 1-like (S. cerevisiae	8.62E-02	4.34E-02	23.8	2.0	0.9	0.003
dnajb1a	445177	DnaJ (Hsp40) homolog, subfamily B, member 1a	1.88E-02	5.26E-02	3.7	3.7	3.7	0.003
ncaldb	445319	neurocalcin delta b	1.27E-02	5.36E-02	0.3	1.3	0.8	0.003
polr1d	445412	polymerase (RNA) I polypeptide D	4.75E-02	7.94E-02	0.3	1.3	0.8	0.003
nfil3	445509	nuclear factor, interleukin 3 regulated	6.69E-02	2.29E-02	12.7	16.2	14.5	0.003
cthl	445818	cystathionase (cystathionine gamma-lyase), like	3.29E-02	8.87E-02	6.0	6.0	6.0	0.003
zgc:92303	447842	zgc:92303	5.45E-02	9.62E-02	12.2	17.0	14.6	0.003
cratb	447855	carnitine O-acetyltransferase b	8.86E-02	5.63E-02	15.5	19.2	17.4	0.003
ap1s3b	447897	adaptor-related protein complex 1, sigma 3 subunit, b	5.36E-02	7.47E-02	23.0	1.7	0.4	0.003
adam9	447940	a disintegrin and metalloproteinase domain 9	6.71E-02	7.75E-02	13.3	15.8	14.6	0.003
acox1	449662	acyl-Coenzyme A oxidase 1, palmitoyl	3.21E-03	8.78E-02	6.5	5.7	6.1	0.003
mcfd2	449676	multiple coagulation factor deficiency 2	6.87E-02	9.26E-02	22.3	0.5	23.4	0.003

Table S1: Zebrafish circadian oscillating genes

Gene Symbol	Gene ID	Gene Description	Fisher's g test p value (LD)	Fisher's g test p value (DD)	LD Phase	DD Phase	Phase *	FDR **
zgc:103681	449773	zgc:103681	6.04E-02	2.01E-03	3.8	4.8	4.3	0.003
samsn1b	450018	SAM domain, SH3 domain and nuclear localisation signa	9.16E-04	7.60E-02	1.5	0.5	1.0	0.003
ankrd9	492328	ankyrin repeat domain 9	9.47E-03	8.11E-02	4.2	2.9	3.5	0.003
pgam5	492357	phosphoglycerate mutase family member 5	5.14E-02	2.67E-02	16.3	16.8	16.6	0.003
stam2	492497	signal transducing adaptor molecule (SH3 domain and IT	1.14E-02	5.24E-02	14.3	15.8	15.1	0.003
zgc:103414	492522	zgc:103414	6.13E-02	2.95E-03	21.8	23.5	22.7	0.003
bin2a	492780	bridging integrator 2a	9.00E-02	2.28E-02	22.3	23.2	22.8	0.003
c3orf58a	492795	C3orf58 chromosome 3 open reading frame 58, a	3.36E-02	5.65E-02	13.8	17.0	15.4	0.003
rhoua	492802	ras homolog gene family, member Ua	2.69E-03	7.43E-02	5.7	5.3	5.5	0.003
pygl	493916	phosphorylase, glycogen; liver (Hers disease, glycogen s	6.75E-02	4.56E-02	18.8	18.7	18.8	0.003
lonp2	494030	lon peptidase 2, peroxisomal	8.77E-03	7.86E-02	11.5	14.2	12.9	0.003
pus1	494060	pseudouridine synthase 1	5.88E-02	1.02E-02	22.3	1.8	0.1	0.003
guca1d	494573	guanylate cyclase activator 1d	6.69E-02	2.76E-02	2.5	2.7	2.6	0.003
nfybb	503744	nuclear transcription factor Y, beta b	4.27E-03	7.75E-02	15.7	18.2	17.0	0.003
zgc:110308	541347	zgc:110308	5.35E-02	4.48E-02	13.9	16.5	15.2	0.003
zgc:103571	541401	zgc:103571	3.92E-02	5.23E-02	23.7	23.3	23.5	0.003
slc47a1	541497	solute carrier family 47, member 1	1.70E-02	5.96E-02	2.8	3.3	3.1	0.003
zgc:113364	548339	zgc:113364	7.64E-02	4.85E-03	13.8	15.7	14.8	0.003
rhbdl3	550128	rhomboid, veinlet-like 3 (Drosophila)	6.08E-02	7.24E-02	13.3	14.5	13.9	0.003
zgc:110224	550335	zgc:110224	4.05E-02	8.38E-02	6.2	6.8	6.5	0.003
pdss1	550349	prenyl (decaprenyl) diphosphate synthase, subunit 1	7.07E-03	8.77E-02	23.7	0.5	0.1	0.003
rnmtl1a	550390	RNA methyltransferase like 1a	1.57E-02	5.56E-02	22.8	0.8	23.8	0.003
idh3g	550408	isocitrate dehydrogenase 3 (NAD+) gamma	9.94E-02	8.56E-02	15.3	18.2	16.8	0.003
zgc:110182	550535	zgc:110182	5.44E-02	8.01E-02	13.2	15.0	14.1	0.003
zgc:112992	550608	zgc:112992	1.85E-02	9.26E-02	1.3	2.5	1.9	0.003
crybb3	553184	crystallin, beta B3	1.41E-02	9.91E-02	10.8	15.0	12.9	0.003
tom1	553244	target of myb1 (chicken)	4.64E-04	8.75E-02	4.9	6.1	5.5	0.003
ptcd3	553325	pentatricopeptide repeat domain 3	5.23E-02	9.55E-02	22.2	23.3	22.8	0.003
lrit2	553557	leucine-rich repeat, immunoglobulin-like and transmembı	1.70E-02	5.55E-02	9.4	12.7	11.1	0.003
zgc:109982	553564	zgc:109982	4.91E-03	6.08E-02	21.7	0.8	23.3	0.003
pin4	553574	protein (peptidylprolyl cis/trans isomerase) NIMA-interact	7.51E-02	8.26E-02	1.7	2.2	2.0	0.003

Table S1: Zebrafish circadian oscillating genes

Gene Symbol	Gene ID	Gene Description	Fisher's g test p value (LD)	Fisher's g test p value (DD)	LD Phase	DD Phase	Phase *	FDR **
zgc:110031	553580	zgc:110031	1.67E-02	5.40E-02	23.8	1.8	0.8	0.003
ube2q1	553603	ubiquitin-conjugating enzyme E2Q (putative) 1	5.13E-02	9.54E-02	15.7	18.0	16.9	0.003
hrasb	553656	-Ha-ras Harvey rat sarcoma viral oncogene homolog b	2.34E-02	6.21E-02	15.0	14.0	14.5	0.003
galnt7	553668	UDP-N-acetyl-alpha-D-galactosamine: polypeptide N-ace	8.84E-02	6.95E-02	6.3	7.7	7.0	0.003
mgat3	553676	mannosyl (beta-1,4-)-glycoprotein beta-1,4-N-acetylgluco	9.66E-02	6.02E-02	2.7	23.7	1.2	0.003
zgc:112320	553708	zgc:112320	2.30E-02	9.09E-02	21.3	0.4	22.9	0.003
zgc:110652	553790	zgc:110652	2.70E-02	5.43E-02	2.2	4.0	3.1	0.003
zgc:113305	553815	zgc:113305	3.04E-02	6.09E-02	0.0	0.3	0.2	0.003
gnpnat1	554143	glucosamine-phosphate N-acetyltransferase 1	2.52E-02	7.62E-02	0.3	1.2	0.8	0.003
psmd1	554967	proteasome (prosome, macropain) 26S subunit, non-ATP	8.99E-02	2.39E-02	18.4	19.3	18.8	0.003
acsl1	555308	acyl-CoA synthetase long-chain family member 1	8.83E-02	8.11E-02	3.7	4.3	4.0	0.003
zyg11	555349	zyg-11 homolog (C. elegans)	5.21E-03	9.22E-02	12.8	14.8	13.8	0.003
zgc:154142	555481	zgc:154142	1.34E-02	5.89E-02	3.2	6.6	4.9	0.003
slc2a1a	555778	solute carrier family 2 (facilitated glucose transporter), me	5.32E-03	9.33E-02	3.2	2.8	3.0	0.003
ppargc1b	555906	peroxisome proliferative activated receptor, gamma, coa	5.54E-02	7.64E-03	21.8	10.5	16.2	0.003
arpc5lb	556104	actin related protein 2/3 complex, subunit 5-like, b	4.35E-05	6.79E-02	20.2	21.7	21.0	0.003
LOC556362	556362	alkylated DNA repair protein alkB homolog 8-like	4.78E-02	9.46E-02	23.5	1.5	0.5	0.003
mxra8b	556385	matrix-remodelling associated 8b	4.33E-02	6.05E-02	13.5	16.1	14.8	0.003
si:dkeyp-22b2.3	556399	si:dkeyp-22b2.3	2.83E-02	5.16E-02	10.7	16.5	13.6	0.003
LOC556700	556700	similar to sphingomyelin phosphodiesterase 1, acid lysos	2.51E-02	5.11E-02	14.2	16.3	15.3	0.003
lrpprc	557195	leucine-rich PPR-motif containing	1.76E-02	7.27E-02	23.8	12.0	17.9	0.003
pdzd3a	557269	PDZ domain containing 3a	1.48E-02	8.52E-02	4.8	4.3	4.6	0.003
chchd1	557606	coiled-coil-helix-coiled-coil-helix domain containing 1	3.04E-02	7.66E-02	0.3	0.7	0.5	0.003
zgc:154015	557682	zgc:154015	1.38E-02	6.15E-02	15.7	16.7	16.2	0.003
hlfb	557886	hepatic leukemia factor b	6.15E-02	3.62E-02	13.3	15.2	14.3	0.003
si:ch211-88n13.3	558093	si:ch211-88n13.3	3.61E-03	6.89E-02	0.2	23.0	23.6	0.003
cspg5a	558206	chondroitin sulfate proteoglycan 5a	2.37E-02	6.81E-02	16.6	16.2	16.4	0.003
si:ch211-15d5.5	558368	si:ch211-15d5.5	8.14E-03	8.66E-02	3.7	3.5	3.6	0.003
si:dkey-30c15.13	558731	si:dkey-30c15.13	6.15E-02	5.05E-02	22.5	0.8	23.7	0.003
si:ch211-89f7.1	558964	si:ch211-89f7.1	2.47E-02	5.99E-02	1.8	4.7	3.3	0.003
LOC559575	559575	nuclear receptor coactivator 7-like	6.39E-02	1.14E-03	1.8	3.7	2.8	0.003

Table S1: Zebrafish circadian oscillating genes

Gene Symbol	Gene ID	Gene Description	Fisher's g test p value (LD)	Fisher's g test p value (DD)	LD Phase	DD Phase	Phase *	FDR **
si:ch211-106h4.4	559790	si:ch211-106h4.4	1.97E-02	5.81E-02	2.7	2.8	2.8	0.003
LOC559868	559868	rho guanine nucleotide exchange factor 9-like	2.74E-04	8.89E-02	2.0	2.2	2.1	0.003
bmp1b	559877	bone morphogenetic protein 1b	5.19E-02	1.04E-02	16.0	16.8	16.4	0.003
si:dkey-14a7.1	559995	si:dkey-14a7.1	7.77E-03	5.63E-02	15.5	16.7	16.1	0.003
LOC560410	560410	adenylate cyclase type 8-like	8.23E-02	1.49E-02	17.3	17.5	17.4	0.003
btbd10a	560466	BTB (POZ) domain containing 10a	8.20E-03	6.14E-02	14.8	15.7	15.3	0.003
hspa13	560481	heat shock protein 13	5.90E-02	5.64E-03	22.0	22.8	22.4	0.003
pycrl	560682	pyrroline-5-carboxylate reductase-like	6.52E-02	6.34E-02	0.2	3.2	1.7	0.003
atp2b1b	561190	ATPase, Ca++ transporting, plasma membrane 1b	2.22E-03	9.92E-02	19.8	20.7	20.3	0.003
si:ch211-254d18.3	561194	si:ch211-254d18.3	1.19E-02	9.71E-02	23.8	22.3	23.1	0.003
tep1	561284	telomerase-associated protein 1	5.63E-02	9.30E-02	2.8	3.2	3.0	0.003
atoh8	561606	atonal homolog 8	5.81E-02	1.09E-02	17.8	20.5	19.2	0.003
lpgat1	561832	lysophosphatidylglycerol acyltransferase 1	4.42E-02	8.75E-02	19.7	17.6	18.6	0.003
vcam1	561971	vascular cell adhesion molecule 1	2.56E-03	8.10E-02	6.0	7.5	6.8	0.003
LOC562156	562156	neurotrypsin-like	2.82E-02	9.00E-02	23.0	0.0	23.5	0.003
rorcb	562258	RAR-related orphan receptor C b	5.84E-02	2.28E-04	11.5	13.3	12.4	0.003
slc39a4	562762	solute carrier family 39 (zinc transporter), member 4	7.11E-02	4.53E-02	2.0	2.8	2.4	0.003
slco1c1	562772	solute carrier organic anion transporter family, member 1	7.23E-02	2.22E-02	14.2	17.8	16.0	0.003
LOC563252	563252	protein CBFA2T3-like	1.38E-02	7.37E-02	17.7	17.7	17.7	0.003
gprc5c	563405	G protein-coupled receptor, family C, group 5, member C	3.14E-02	8.78E-02	2.7	1.8	2.3	0.003
ugt5c3	563413	UDP glucuronosyltransferase 5 family, polypeptide C3	8.87E-02	2.08E-02	20.5	1.3	22.9	0.003
LOC563672	563672	BTB/POZ domain-containing protein 17-like	3.28E-02	7.36E-02	2.5	2.8	2.7	0.003
per1a	563926	period homolog 1a (Drosophila)	4.59E-02	7.35E-02	23.8	2.3	1.0	0.003
gstt1a	563972	glutathione S-transferase theta 1a	1.79E-02	9.19E-02	12.3	13.7	13.0	0.003
wdr85	564032	WD repeat domain 85	4.00E-02	7.65E-02	16.2	19.5	17.9	0.003
trmt5	564078	TRM5 tRNA methyltransferase 5 homolog (S. cerevisiae	5.32E-02	5.39E-02	23.4	0.9	0.1	0.003
sb:cb474	564134	sb:cb474	1.48E-03	5.39E-02	5.5	5.0	5.3	0.003
ip6k1	564478	inositol hexakisphosphate kinase 1	7.51E-03	6.48E-02	2.0	2.2	2.1	0.003
si:dkeyp-73d8.3	564534	si:dkeyp-73d8.3	7.93E-05	6.06E-02	21.7	21.3	21.5	0.003
slc5a9	564815	solute carrier family 5 (sodium/glucose cotransporter), m	5.97E-02	5.79E-03	2.5	5.3	3.9	0.003
spag1	564953	sperm associated antigen 1	6.86E-02	1.54E-02	0.5	0.8	0.6	0.003

Table S1: Zebrafish circadian oscillating genes

Gene Symbol	Gene ID	Gene Description	Fisher's g test p value (LD)	Fisher's g test p value (DD)	LD Phase	DD Phase	Phase *	FDR **
LOC566570	566570	solute carrier family 22 member 6-like	5.94E-02	4.33E-03	1.5	23.8	0.7	0.003
LOC566587	566587	ERBB receptor feedback inhibitor 1-like	9.70E-02	1.48E-02	1.0	22.3	23.7	0.003
LOC566599	566599	hypothetical LOC566599	1.71E-02	5.52E-02	20.7	20.7	20.7	0.003
LOC566659	566659	hypothetical LOC566659	2.45E-02	9.50E-02	6.0	7.7	6.9	0.003
znf395b	566703	zinc finger protein 395b	1.40E-03	7.18E-02	0.8	3.0	1.9	0.003
zgc:163083	566848	zgc:163083	4.15E-04	7.23E-02	3.5	3.8	3.7	0.003
epas1a	566886	endothelial PAS domain protein 1a	3.81E-02	7.99E-02	3.3	3.9	3.6	0.003
ptpmt1	567019	protein tyrosine phosphatase, mitochondrial 1	3.81E-02	8.10E-02	1.0	1.8	1.4	0.003
LOC567075	567075	hypothetical protein LOC567075	3.08E-03	6.68E-02	23.2	21.8	22.5	0.003
lrmp	567234	lymphoid-restricted membrane protein	1.68E-02	6.88E-02	22.8	8.1	3.5	0.003
agpat9l	567414	1-acylglycerol-3-phosphate O-acyltransferase 9, like	8.97E-04	5.48E-02	4.7	4.8	4.8	0.003
si:ch211-69i14.7	567417	si:ch211-69i14.7	8.13E-02	5.51E-02	12.0	13.5	12.8	0.003
cbx3b	567497	chromobox homolog 3b	9.19E-02	6.36E-02	8.0	5.0	6.5	0.003
asb2a	567986	ankyrin repeat and SOCS box-containing 2a	1.04E-02	5.73E-02	15.4	16.7	16.0	0.003
zgc:109984	568055	zgc:109984	6.48E-02	1.22E-02	13.5	13.7	13.6	0.003
wu:fc76b04	568156	wu:fc76b04	3.38E-02	7.30E-02	23.6	2.5	1.1	0.003
LOC568313	568313	hypothetical protein LOC568313	3.53E-02	6.41E-02	22.0	1.0	23.5	0.003
nars	568332	asparaginyl-tRNA synthetase	3.60E-02	9.35E-02	0.5	22.7	23.6	0.003
LOC568678	568678	similar to PAP-associated domain-containing protein 5 (T	5.40E-03	5.49E-02	2.3	2.7	2.5	0.003
LOC569046	569046	methyltransferase-like protein 7A-like	5.16E-02	1.79E-03	1.5	3.0	2.3	0.003
si:dkeyp-69b9.5	569109	si:dkeyp-69b9.5	4.99E-02	6.47E-02	22.0	0.5	23.3	0.003
ecsit	569243	ECSIT homolog (Drosophila)	1.39E-02	7.35E-02	0.1	1.1	0.6	0.003
LOC569518	569518	ankyrin repeat domain-containing protein 33B-like	1.92E-03	8.60E-02	3.5	1.0	2.3	0.003
zgc:123347	569617	zgc:123347	5.21E-02	7.45E-03	1.8	2.8	2.3	0.003
LOC569661	569661	hypothetical protein LOC569661	7.91E-02	5.87E-03	22.2	23.3	22.8	0.003
abcb9	570148	ATP-binding cassette, sub-family B (MDR/TAP), membe	2.73E-02	9.05E-02	17.6	19.7	18.6	0.003
zgc:123271	570159	zgc:123271	5.91E-02	3.11E-02	22.3	23.7	23.0	0.003
lrrtm1	570385	leucine rich repeat transmembrane neuronal 1	4.94E-02	9.81E-02	16.5	16.7	16.6	0.003
si:dkeyp-84f11.5	570476	si:dkeyp-84f11.5	3.83E-03	5.65E-02	14.3	17.0	15.6	0.003
letm1	570745	leucine zipper-EF-hand containing transmembrane prote	7.10E-02	8.17E-02	22.1	22.7	22.4	0.003
si:dkey-258f14.3	571152	si:dkey-258f14.3	5.26E-02	8.23E-03	1.8	2.5	2.2	0.003

Table S1: Zebrafish circadian oscillating genes

Gene Symbol	Gene ID	Gene Description	Fisher's g test p value (LD)	Fisher's g test p value (DD)	LD Phase	DD Phase	Phase *	FDR **
zgc:162913	571830	zgc:162913	3.73E-02	8.22E-02	14.2	17.3	15.8	0.003
zgc:165520	571872	zgc:165520	6.46E-02	2.70E-02	18.8	19.8	19.3	0.003
LOC571944	571944	tripartite motif-containing protein 58-like	2.32E-03	9.56E-02	12.5	13.8	13.2	0.003
nr1d4b	571948	nuclear receptor subfamily 1, group D, member 4b	4.67E-02	7.41E-02	19.0	20.5	19.8	0.003
cyp2ae1	572114	cytochrome P450, family 2, subfamily AE, polypeptide 1	5.88E-02	4.69E-03	10.3	14.8	12.6	0.003
mns1	573382	meiosis-specific nuclear structural 1	4.62E-03	6.93E-02	13.2	16.7	15.0	0.003
si:ch211-103f16.2	613021	si:ch211-103f16.2	4.75E-02	5.35E-02	0.2	1.8	1.0	0.003
zgc:112334	619199	zgc:112334	6.53E-02	2.56E-02	12.8	15.0	13.9	0.003
zgc:109965	619266	zgc:109965	3.73E-02	9.30E-02	21.0	18.2	19.6	0.003
zgc:123284	641422	zgc:123284	8.85E-02	1.97E-02	4.2	6.0	5.1	0.003
ripply2	654447	ripply2	1.75E-02	9.07E-02	4.7	5.0	4.9	0.003
zgc:110045	664755	zgc:110045	5.18E-02	5.94E-02	12.3	13.5	12.9	0.003
zgc:136360	678588	zgc:136360	7.04E-02	3.89E-02	23.1	1.6	0.4	0.003
unc119b	678653	unc-119 homolog b (C. elegans)	3.21E-02	7.90E-02	3.3	11.9	7.6	0.003
zgc:136353	692303	zgc:136353	1.85E-02	7.62E-02	0.0	2.2	1.1	0.003
zgc:136808	692332	zgc:136808	4.04E-02	5.93E-02	4.7	4.2	4.5	0.003
st8sia7.1	751830	ST8 alpha-N-acetyl-neuraminide alpha-2,8-sialyltransfera	7.43E-02	3.82E-02	21.8	20.8	21.3	0.003
deptor	768177	DEP domain containing MTOR-interacting protein	3.55E-03	8.16E-02	3.7	4.2	3.9	0.003
zgc:153420	768187	zgc:153420	2.20E-02	9.67E-02	0.2	1.7	1.0	0.003
zgc:153423	768297	zgc:153423	1.58E-02	9.79E-02	15.4	16.0	15.7	0.003
zgc:152898	777614	zgc:152898	5.82E-02	8.86E-02	11.8	15.3	13.6	0.003
zgc:154054	777616	zgc:154054	3.43E-02	9.21E-02	11.9	1.6	6.7	0.003
zgc:152965	777746	zgc:152965	1.11E-02	6.37E-02	22.8	23.8	23.3	0.003
zgc:158659	791141	zgc:158659	5.90E-04	5.32E-02	3.5	3.8	3.7	0.003
zgc:158868	791147	zgc:158868	2.81E-03	8.88E-02	5.6	5.5	5.5	0.003
ccbl1	791592	cysteine conjugate-beta lyase; cytoplasmic (glutamine tra	8.77E-02	2.04E-02	3.8	4.6	4.2	0.003
eef2a.1	792182	eukaryotic translation elongation factor 2a, tandem duplic	5.54E-02	4.15E-02	0.7	1.7	1.2	0.003
saga	792319	S-antigen; retina and pineal gland (arrestin) a	1.71E-03	9.83E-02	22.7	0.8	23.7	0.003
LOC792903	792903	recoverin-like	1.52E-02	5.29E-02	21.3	2.5	23.9	0.003
LOC793794	793794	Krueppel-like factor 10-like	1.93E-02	7.89E-02	6.0	7.5	6.8	0.003
LOC794083	794083	pyruvate dehydrogenase [lipoamide] kinase isozyme 2-lil	1.09E-02	9.93E-02	8.8	9.7	9.3	0.003

Table S1: Zebrafish circadian oscillating genes

Gene Symbol	Gene ID	Gene Description	Fisher's g test p value (LD)	Fisher's g test p value (DD)	LD Phase	DD Phase	Phase *	FDR **
zgc:162150	794783	zgc:162150	5.88E-02	1.52E-02	5.7	7.3	6.5	0.003
sema4e	796377	semaphorin 4e	1.82E-03	5.25E-02	13.2	16.7	15.0	0.003
LOC796577	796577	novel immunoglobulin I-set domain containing protein	7.25E-02	1.96E-02	23.2	0.5	23.9	0.003
wu:fk33d07	797198	wu:fk33d07	8.38E-02	9.79E-02	22.5	1.3	23.9	0.003
LOC797371	797371	tripartite motif-containing protein 39-like	2.08E-02	5.29E-02	5.0	5.2	5.1	0.003
tex2	798401	testis expressed 2	2.31E-02	8.91E-02	13.7	13.3	13.5	0.003
rhbdf1	798402	rhomboid 5 homolog 1 (Drosophila)	6.27E-02	1.80E-02	18.3	20.3	19.3	0.003
LOC799177	799177	heat shock factor binding protein 1-like	1.59E-02	7.98E-02	6.3	3.8	5.1	0.003
dio1	799937	deiodinase, iodothyronine, type I	1.07E-02	9.86E-02	5.3	3.0	4.2	0.003
fgfr4	100000160	fibroblast growth factor receptor 4	4.32E-02	7.79E-02	14.7	16.0	15.3	0.003
LOC100000736	100000736	membrane-spanning 4-domains subfamily A member 4A	4.45E-02	5.48E-02	16.3	17.3	16.8	0.003
LOC100000962	100000962	hypothetical protein LOC100000962	2.40E-02	7.32E-02	3.7	3.3	3.5	0.003
LOC100001136	100001136	occludin-like	7.48E-02	9.58E-02	14.7	14.5	14.6	0.003
stard10	100001416	START domain containing 10	4.40E-02	9.72E-02	2.0	1.3	1.7	0.003
LOC100001772	100001772	hypothetical protein LOC100001772	8.49E-02	8.81E-02	3.2	4.3	3.8	0.003
LOC100001838	100001838	solute carrier family 22 member 7-like	5.76E-02	1.98E-02	1.5	4.0	2.8	0.003
si:ch211-283h6.6	100001978	si:ch211-283h6.6	1.35E-02	8.86E-02	15.2	16.3	15.8	0.003
LOC100002059	100002059	A disintegrin and metalloproteinase with thrombospondin	6.29E-02	2.75E-02	5.2	5.7	5.5	0.003
tmem70	100002535	transmembrane protein 70	1.16E-02	6.72E-02	21.5	23.8	22.7	0.003
si:ch211-157p22.10	100003849	si:ch211-157p22.10	6.32E-02	5.02E-02	10.7	15.5	13.1	0.003
LOC100003999	100003999	2-epi-5-epi-valiolone synthase-like	4.75E-02	9.93E-02	2.2	1.2	1.7	0.003
LOC100004247	100004247	solute carrier family 38, member 8	8.52E-02	1.43E-02	1.2	1.8	1.5	0.003
hal	100004331	histidine ammonia-lyase	5.82E-02	5.70E-02	16.2	17.5	16.9	0.003
si:dkey-39n1.3	100004437	si:dkey-39n1.3	2.14E-04	7.55E-02	16.2	16.7	16.5	0.003
zgc:194242	100005854	zgc:194242	6.74E-02	6.74E-02	5.2	5.0	5.1	0.003
idh1	100006589	isocitrate dehydrogenase 1 (NADP+), soluble	1.12E-02	6.25E-02	7.7	8.0	7.9	0.003
LOC100006660	100006660	similar to phosphatidic acid phosphatase type 2d	3.14E-02	8.32E-02	16.3	16.3	16.3	0.003
adrb2b	100037315	adrenergic receptor, beta 2b	6.81E-02	1.49E-02	1.0	2.3	1.7	0.003
zgc:162608	100037332	zgc:162608	7.50E-02	1.87E-03	5.2	2.8	4.0	0.003
zgc:163044	100037354	zgc:163044	8.54E-02	5.52E-02	12.7	16.2	14.5	0.003
zgc:162495	100038771	zgc:162495	1.08E-02	9.72E-02	5.8	6.0	5.9	0.003

Table S1: Zebrafish circadian oscillating genes

Gene Symbol	Gene ID	Gene Description	Fisher's g test p value (LD)	Fisher's g test p value (DD)	LD Phase	DD Phase	Phase *	FDR **
zgc:163121	100038790	zgc:163121	4.90E-04	5.29E-02	5.7	7.2	6.5	0.003
ralgps1	100101642	Ral GEF with PH domain and SH3 binding motif 1	6.50E-02	5.00E-02	15.0	15.1	15.0	0.003
pnpla8	100126812	patatin-like phospholipase domain containing 8	5.19E-02	2.75E-02	13.5	14.4	13.9	0.003
hbl4	100137128	hexose-binding lectin 4	1.21E-02	9.71E-02	10.8	9.1	9.9	0.003
si:ch211-119o8.7	100141334	si:ch211-119o8.7	5.38E-02	5.75E-02	15.0	15.2	15.1	0.003
mgam	100148922	maltase-glucoamylase (alpha-glucosidase)	5.21E-02	3.28E-03	3.7	7.0	5.4	0.003
LOC100150195	100150195	gap junction beta-1 protein-like	1.00E-02	6.06E-02	3.7	3.7	3.7	0.003
zgc:194737	100170786	zgc:194737	3.81E-03	7.84E-02	2.5	1.7	2.1	0.003
snx19a	100170806	sorting nexin 19a	3.85E-02	9.75E-02	16.8	17.0	16.9	0.003
si:dkeyp-51f11.8	100319971	si:dkeyp-51f11.8	8.46E-04	5.69E-02	16.7	18.3	17.5	0.003
LOC100332164	100332164	e3 ubiquitin-protein ligase TRIM9-like	6.67E-02	8.47E-03	16.2	15.3	15.8	0.003
LOC100333000	100333000	hypothetical protein LOC100333000	6.21E-02	1.53E-02	3.0	3.7	3.4	0.003
LOC100333650	100333650	nucleolar pre-ribosomal-associated protein 1-like	2.09E-03	9.05E-02	1.3	1.3	1.3	0.003
men1	30130	multiple endocrine neoplasia type 1	8.19E-02	1.46E-01	6.3	8.5	7.4	0.007
mpp7	30166	membrane protein, palmitoylated 7 (MAGUK p55 subfam	1.59E-02	1.14E-01	4.3	3.3	3.8	0.007
cx43	30236	connexin 43	1.20E-01	7.93E-02	0.5	3.2	1.9	0.007
her2	30300	hairy-related 2	1.05E-01	4.79E-02	4.8	5.0	4.9	0.007
hoxa11b	30382	homeo box A11b	3.39E-02	1.33E-01	19.5	20.3	19.9	0.007
foxa1	30541	forkhead box A1	6.22E-02	1.01E-01	13.8	15.5	14.7	0.007
vsx1	30598	visual system homeobox 1 homolog, chx10-like	1.15E-01	1.42E-01	15.8	15.8	15.8	0.007
psme3	30649	proteasome activator subunit 3	7.97E-03	1.06E-01	21.1	23.2	22.2	0.007
pdgfra	30745	platelet derived growth factor receptor alpha	4.17E-02	1.24E-01	12.5	14.2	13.4	0.007
fgd	58075	faciogenital dysplasia	6.07E-03	1.02E-01	17.0	17.7	17.4	0.007
pard6gb	80959	par-6 partitioning defective 6 homolog gamma B (C. eleg	6.09E-02	1.03E-01	3.2	4.7	4.0	0.007
cry5	83776	cryptochrome 5	1.14E-01	7.48E-02	5.0	3.0	4.0	0.007
klf2b	117509	Kruppel-like factor 2b	1.22E-01	4.07E-04	13.5	13.5	13.5	0.007
gnat1	140428	guanine nucleotide binding protein (G protein), alpha trar	7.83E-02	1.00E-01	15.0	14.8	14.9	0.007
guca1a	140430	guanylate cyclase activator 1A	5.81E-02	1.25E-01	19.7	22.3	21.0	0.007
cebpa	140815	CCAAT/enhancer binding protein (C/EBP), alpha	4.36E-02	1.45E-01	21.2	11.7	16.5	0.007
ccng2	266794	cyclin G2	3.12E-03	1.11E-01	12.2	14.3	13.3	0.007
dnmt4	317744	DNA (cytosine-5-)-methyltransferase 4	4.41E-02	1.19E-01	1.7	2.8	2.3	0.007

Table S1: Zebrafish circadian oscillating genes

Gene Symbol	Gene ID	Gene Description	Fisher's g test p value (LD)	Fisher's g test p value (DD)	LD Phase	DD Phase	Phase *	FDR **
sb:cb307	321198	sb:cb307	1.04E-01	6.96E-02	14.0	13.0	13.5	0.007
pcm1	321709	pericentriolar material 1	4.79E-03	1.02E-01	15.2	15.8	15.5	0.007
keap1a	321837	kelch-like ECH-associated protein 1a	1.64E-02	1.05E-01	5.5	5.3	5.4	0.007
frmd6	321937	FERM domain containing 6	5.16E-03	1.17E-01	14.0	16.0	15.0	0.007
wu:fb38h10	321946	wu:fb38h10	1.37E-01	3.96E-02	21.2	18.2	19.7	0.007
ddx5	322206	DEAD (Asp-Glu-Ala-Asp) box polypeptide 5	3.80E-03	1.02E-01	19.3	21.0	20.2	0.007
rpl7l1	322251	ribosomal protein L7-like 1	1.47E-01	3.20E-02	12.0	1.6	6.8	0.007
psmd11b	322265	proteasome (prosome, macropain) 216S subunit, non-AT	4.83E-02	1.19E-01	16.0	17.0	16.5	0.007
yy1a	322397	YY1 transcription factor a	9.30E-03	1.34E-01	11.7	13.7	12.7	0.007
map1lc3b	322425	microtubule-associated protein 1 light chain 3 beta	7.20E-02	1.39E-01	14.1	15.8	14.9	0.007
uap1	322513	UDP-N-acteylglucosamine pyrophosphorylase 1	1.22E-01	1.85E-02	12.0	12.4	12.2	0.007
dnajc11	322953	DnaJ (Hsp40) homolog, subfamily C, member 11	1.04E-01	1.36E-01	23.7	1.5	0.6	0.007
pgam1a	323107	phosphoglycerate mutase 1a	9.93E-03	1.45E-01	2.3	3.8	3.1	0.007
mat2a	323329	methionine adenosyltransferase II, alpha	1.56E-02	1.45E-01	0.5	0.8	0.7	0.007
hnf1bb	324283	HNF1 homeobox Bb	4.31E-03	1.07E-01	14.3	15.3	14.8	0.007
slc39a6	324303	solute carrier family 39 (zinc transporter), member 6	2.23E-02	1.45E-01	18.8	19.2	19.0	0.007
cul3a	324655	cullin 3a	5.29E-02	1.34E-01	16.8	17.8	17.3	0.007
sppl2	325540	signal peptide peptidase-like 2	2.21E-02	1.43E-01	15.8	11.2	13.5	0.007
zgc:73340	326652	zgc:73340	1.28E-02	1.20E-01	2.2	3.0	2.6	0.007
zff9	326846	zinc finger protein 9	9.24E-02	1.49E-01	1.3	4.3	2.8	0.007
rrp15	327053	ribosomal RNA processing 15 homolog (S. cerevisiae)	1.31E-01	3.17E-02	23.8	1.3	0.6	0.007
uck2b	327057	uridine-cytidine kinase 2b	1.25E-01	1.24E-01	11.8	12.2	12.0	0.007
inka1b	327328	induced in neural crest by AP2, 1b	1.33E-01	6.12E-02	1.1	3.4	2.2	0.007
ampd3	333997	adenosine monophosphate deaminase 3	5.97E-02	1.28E-01	12.8	14.5	13.6	0.007
spsb1	334190	splA/ryanodine receptor domain and SOCS box containi	8.60E-03	1.05E-01	13.0	14.1	13.6	0.007
aqp7	334529	aquaporin 7	1.41E-01	1.02E-01	17.2	19.0	18.1	0.007
synj2bp	334599	synaptojanin 2 binding protein	1.28E-01	1.04E-01	3.5	4.2	3.9	0.007
ccnc	335429	cyclin C	1.01E-01	1.00E-01	11.3	15.5	13.4	0.007
wu:fj15c08	335467	wu:fj15c08	1.35E-01	5.31E-02	12.7	14.7	13.7	0.007
ppap2c	335485	phosphatidic acid phosphatase type 2C	6.71E-02	1.50E-01	15.3	16.8	16.1	0.007
dfna5	335722	deafness, autosomal dominant 5	9.29E-02	1.31E-01	12.2	14.3	13.2	0.007

Table S1: Zebrafish circadian oscillating genes

Gene Symbol	Gene ID	Gene Description	Fisher's g test p value (LD)	Fisher's g test p value (DD)	LD Phase	DD Phase	Phase *	FDR **
chmp1b	336426	chromatin modifying protein 1B	8.59E-02	1.08E-01	15.5	17.4	16.4	0.007
icn	336655	ictacalcin	8.22E-02	1.19E-01	6.3	7.7	7.0	0.007
lamp1	337357	lysosomal-associated membrane protein 1	1.39E-01	1.36E-01	14.3	15.0	14.7	0.007
gli1	352930	GLI-Kruppel family member 1	1.29E-01	9.96E-02	15.0	13.8	14.4	0.007
slc16a1	368274	solute carrier family 16 (monocarboxylic acid transporters	6.27E-02	1.48E-01	2.0	2.8	2.4	0.007
si:ch211-217g15.3	368914	si:ch211-217g15.3	1.01E-01	3.28E-02	0.3	3.0	1.7	0.007
tjp3	373089	tight junction protein 3	9.98E-02	1.09E-01	12.9	14.6	13.7	0.007
cbr1	373866	carbonyl reductase 1	4.31E-04	1.09E-01	5.8	3.7	4.8	0.007
pdcb	386614	phosducin b	9.93E-05	1.27E-01	3.2	2.4	2.8	0.007
fli1b	386723	friend leukemia integration 1b	1.02E-01	3.87E-02	13.8	14.5	14.2	0.007
stam	386967	signal transducing adaptor molecule (SH3 domain and IT	1.16E-01	1.39E-01	14.7	16.9	15.8	0.007
cyp4t8	387527	cytochrome P450, family 4, subfamily T, polypeptide 8	6.59E-03	1.48E-01	7.0	6.2	6.6	0.007
ivns1abpa	387591	influenza virus NS1A binding protein a	1.07E-01	4.06E-02	11.9	13.3	12.6	0.007
lipg	393096	lipase, endothelial	1.10E-01	5.16E-02	3.8	2.5	3.2	0.007
rad18	393106	RAD18 homolog (S. cerevisiae)	1.01E-01	3.31E-02	6.0	7.5	6.8	0.007
cyp2p6	393108	cytochrome P450, family 2, subfamily P, polypeptide 6	1.12E-02	1.45E-01	5.4	6.5	6.0	0.007
zgc:55634	393138	zgc:55634	1.50E-01	9.66E-02	13.3	16.0	14.7	0.007
zgc:55815	393145	zgc:55815	8.48E-02	1.41E-01	14.5	16.8	15.6	0.007
usp44	393227	ubiquitin specific peptidase 44	1.32E-01	1.05E-01	13.3	14.7	14.0	0.007
hsd17b3	393335	hydroxysteroid (17-beta) dehydrogenase 3	7.01E-02	1.32E-01	3.8	4.5	4.2	0.007
adam10b	393392	a disintegrin and metalloprotease domain 10b	1.15E-01	4.50E-02	16.3	18.0	17.2	0.007
zgc:63523	393573	zgc:63523	1.48E-01	1.32E-02	13.5	15.8	14.7	0.007
slc25a1	393579	solute carrier family 25 (mitochondrial carrier; citrate tran	2.03E-02	1.30E-01	12.5	15.2	13.8	0.007
cdkal1	393600	CDK5 regulatory subunit associated protein 1-like 1	3.43E-02	1.35E-01	22.6	11.5	17.1	0.007
ccdc43	393631	coiled-coil domain containing 43	1.18E-01	2.04E-02	23.7	1.8	0.8	0.007
zgc:66030	393649	zgc:66030	9.56E-02	1.05E-01	17.2	19.0	18.1	0.007
rwdd3	393729	RWD domain containing 3	3.87E-02	1.41E-01	13.5	16.2	14.9	0.007
ccdc58	393780	coiled-coil domain containing 58	5.34E-02	1.09E-01	0.2	1.2	0.7	0.007
gnao1b	393801	guanine nucleotide binding protein (G protein), alpha acti	1.28E-01	8.48E-02	1.7	2.0	1.9	0.007
zgc:73359	393810	zgc:73359	1.16E-03	1.06E-01	1.8	3.2	2.5	0.007
tppp3	393825	tubulin polymerization-promoting protein family member :	1.30E-01	5.30E-02	8.2	6.8	7.5	0.007

Table S1: Zebrafish circadian oscillating genes

Gene Symbol	Gene ID	Gene Description	Fisher's g test p value (LD)	Fisher's g test p value (DD)	LD Phase	DD Phase	Phase *	FDR **
naprt1	393864	nicotinate phosphoribosyltransferase domain containing	1.06E-01	4.90E-02	21.2	22.7	22.0	0.007
uvrag	394044	UV radiation resistance associated gene	1.17E-01	5.41E-02	17.5	18.7	18.1	0.007
minpp1b	394075	multiple inositol polyphosphate histidine phosphatase, 1b	1.42E-02	1.34E-01	17.3	20.0	18.7	0.007
bokb	394160	BCL2-related ovarian killer b	6.57E-04	1.09E-01	4.7	2.3	3.5	0.007
qars	394188	glutaminyl-tRNA synthetase	6.22E-02	1.09E-01	22.3	8.1	3.2	0.007
kri1l	402800	KRI1 homolog like	1.44E-01	3.90E-02	22.8	1.8	0.3	0.007
LOC402804	402804	hypothetical LOC402804	1.01E-01	4.87E-02	15.3	16.8	16.1	0.007
fancg	402936	Fanconi anemia, complementation group G	3.43E-02	1.46E-01	8.2	7.7	8.0	0.007
cry-dash	402986	cryptochrome DASH	1.30E-01	3.13E-02	4.3	2.8	3.6	0.007
rlbp1b	402990	retinaldehyde binding protein 1b	1.39E-01	8.34E-02	4.4	3.4	3.9	0.007
zgc:111878	403024	zgc:111878	9.52E-02	1.37E-01	23.3	0.8	0.1	0.007
tbpl1	403035	TBP-like 1	1.22E-01	7.78E-02	14.3	17.7	16.0	0.007
fbxo25	403037	F-box protein 25	5.06E-02	1.07E-01	19.6	22.8	21.2	0.007
zgc:77041	404632	zgc:77041	1.30E-01	1.29E-01	22.0	0.0	23.0	0.007
arih1l	405859	ariadne homolog, ubiquitin-conjugating enzyme E2 bindi	1.01E-01	1.35E-01	14.5	17.5	16.0	0.007
mrpl38	405881	mitochondrial ribosomal protein L38	6.01E-02	1.21E-01	12.1	0.2	6.1	0.007
fbl	406275	fibrillarin	2.44E-02	1.34E-01	22.6	2.3	0.4	0.007
s100a10b	406276	S100 calcium binding protein A10b	2.77E-02	1.27E-01	6.8	6.5	6.6	0.007
zgc:73347	406293	zgc:73347	3.79E-02	1.38E-01	19.3	22.8	21.1	0.007
ubxn4	406314	UBX domain protein 4	1.47E-01	1.05E-01	15.7	19.0	17.4	0.007
abcf1	406467	ATP-binding cassette, sub-family F (GCN20), member 1	1.12E-01	1.28E-02	22.3	0.5	23.4	0.007
pck2	406473	phosphoenolpyruvate carboxykinase 2 (mitochondrial)	1.51E-02	1.11E-01	1.8	3.5	2.7	0.007
nudt1	406727	nudix (nucleoside diphosphate linked moiety X)-type mot	1.39E-01	3.40E-02	4.5	6.2	5.4	0.007
pank1a	406789	pantothenate kinase 1a	3.28E-05	1.10E-01	23.8	22.8	23.3	0.007
tmem50a	406850	transmembrane protein 50A	7.04E-03	1.34E-01	16.0	19.5	17.8	0.007
mobkl1ab	407654	MOB1, Mps One Binder kinase activator-like 1A (yeast),	7.53E-05	1.06E-01	4.6	3.7	4.1	0.007
ddx52	407696	DEAD (Asp-Glu-Ala-Asp) box polypeptide 52	1.43E-01	2.09E-02	23.3	3.2	1.3	0.007
LOC407698	407698	anoctamin 1, calcium activated chloride channel	1.29E-01	2.92E-02	14.7	15.3	15.0	0.007
psmd4a	415202	proteasome (prosome, macropain) 26S subunit, non-ATF	1.00E-01	6.96E-02	18.8	21.5	20.1	0.007
eaf1	415205	ELL associated factor 1	1.45E-01	2.20E-02	14.2	16.3	15.3	0.007
dram2	415225	DNA-damage regulated autophagy modulator 2	4.70E-02	1.23E-01	14.0	16.8	15.4	0.007

Table S1: Zebrafish circadian oscillating genes

Gene Symbol	Gene ID	Gene Description	Fisher's g test p value (LD)	Fisher's g test p value (DD)	LD Phase	DD Phase	Phase *	FDR **
lrrc6	432388	leucine rich repeat containing 6	9.76E-02	1.20E-01	16.2	18.7	17.5	0.007
entpd8	436652	ectonucleoside triphosphate diphosphohydrolase 8	2.04E-02	1.02E-01	4.2	4.5	4.3	0.007
mpv17l2	436840	MPV17 mitochondrial membrane protein-like 2	5.61E-02	1.15E-01	22.7	0.7	23.7	0.007
tnfsf10l2	436866	tumor necrosis factor (ligand) superfamily, member 10 lik	4.87E-02	1.38E-01	12.6	13.5	13.1	0.007
zgc:91940	436942	zgc:91940	1.06E-01	5.18E-03	2.0	1.2	1.6	0.007
gpr137bb	436964	G protein-coupled receptor 137bb	7.23E-02	1.40E-01	12.7	16.5	14.6	0.007
tfip11	436994	tuftelin interacting protein 11	1.18E-01	1.33E-01	15.7	18.5	17.1	0.007
rs1	445044	retinoschisis (X-linked, juvenile) 1	6.40E-02	1.40E-01	2.2	0.7	1.5	0.007
grwd1	445115	glutamate-rich WD repeat containing 1	2.64E-02	1.08E-01	22.7	0.9	23.8	0.007
polr2eb	445170	polymerase (RNA) II (DNA directed) polypeptide E, b	8.45E-02	1.19E-01	1.2	2.2	1.7	0.007
riok3	445220	RIO kinase 3 (yeast)	7.61E-02	1.47E-01	15.8	17.7	16.8	0.007
mrps23	445221	mitochondrial ribosomal protein S23	2.45E-02	1.24E-01	23.0	0.8	23.9	0.007
wdr55	445394	WD repeat domain 55	1.32E-01	2.59E-02	1.0	3.8	2.4	0.007
epb41l3a	445478	erythrocyte membrane protein band 4.1-like 3a	1.39E-01	5.91E-02	19.5	17.5	18.5	0.007
sdhdb	445500	succinate dehydrogenase complex, subunit D, integral m	4.56E-02	1.24E-01	1.3	1.3	1.3	0.007
rimkla	447815	ribosomal modification protein rimK-like family member A	7.02E-02	1.40E-01	12.4	13.6	13.0	0.007
slc35b1	447844	solute carrier family 35, member B1	7.43E-02	1.47E-01	20.7	23.7	22.2	0.007
zgc:92194	447849	zgc:92194	1.23E-01	4.08E-02	13.8	16.3	15.1	0.007
rrh	447916	retinal pigment epithelium-derived rhodopsin homolog	1.28E-01	9.95E-02	12.7	16.7	14.7	0.007
aqp8a.1	447923	aquaporin 8a, tandem duplicate 1	1.55E-04	1.22E-01	2.2	2.2	2.2	0.007
zgc:92915	449650	zgc:92915	1.02E-01	5.73E-02	12.5	13.2	12.9	0.007
zgc:103636	449779	zgc:103636	1.51E-03	1.17E-01	1.1	3.4	2.2	0.007
polr3glb	449786	polymerase (RNA) III (DNA directed) polypeptide G like b	1.08E-01	8.94E-03	9.3	10.0	9.7	0.007
zgc:103545	449812	zgc:103545	1.48E-01	1.24E-01	23.0	2.0	0.5	0.007
zgc:101827	492513	zgc:101827	1.47E-01	1.17E-01	13.3	16.0	14.7	0.007
xkrx	492765	XK, Kell blood group complex subunit-related, X-linked	1.06E-01	7.10E-02	13.6	16.5	15.0	0.007
efna1b	494151	ephrin A1b	8.02E-02	1.21E-01	8.7	8.7	8.7	0.007
guca1g	494572	guanylate cyclase activator 1g	1.24E-01	7.02E-02	0.7	22.7	23.7	0.007
ext1c	497281	exostoses (multiple) 1c	9.83E-02	1.37E-01	17.1	17.8	17.4	0.007
mrpl48	503593	mitochondrial ribosomal protein L48	1.01E-01	3.65E-02	11.9	0.9	6.4	0.007
ptrh1	503752	peptidyl-tRNA hydrolase 1 homolog (S. cerevisiae)	1.03E-01	1.19E-01	20.8	23.2	22.0	0.007

Table S1: Zebrafish circadian oscillating genes

Gene Symbol	Gene ID	Gene Description	Fisher's g test p value (LD)	Fisher's g test p value (DD)	LD Phase	DD Phase	Phase *	FDR **
zgc:110789	503757	zgc:110789	3.68E-02	1.03E-01	17.3	20.5	18.9	0.007
zgc:112496	541336	zgc:112496	1.08E-01	8.30E-02	7.0	7.5	7.3	0.007
zdhhc2	541365	zinc finger, DHHC-type containing 2	2.65E-03	1.36E-01	11.3	15.3	13.3	0.007
tufm	541378	Tu translation elongation factor, mitochondrial	1.20E-01	1.43E-02	1.3	2.3	1.8	0.007
zgc:101788	541408	zgc:101788	2.23E-04	1.42E-01	4.3	4.0	4.2	0.007
pop5	541421	processing of precursor 5, ribonuclease P/MRP subunit (	1.26E-01	1.42E-01	0.6	1.9	1.2	0.007
znrd1	541428	zinc ribbon domain containing 1	9.79E-02	1.48E-01	0.3	2.2	1.3	0.007
zgc:110801	541536	zgc:110801	3.04E-02	1.27E-01	1.0	12.8	6.9	0.007
exosc2	550234	exosome component 2	1.11E-01	4.43E-02	21.1	22.4	21.7	0.007
cox16	550257	COX16 cytochrome c oxidase assembly homolog (S. cer	1.31E-01	1.48E-01	0.2	1.5	0.9	0.007
zgc:110388	550350	zgc:110388	1.05E-01	9.59E-03	23.8	1.8	0.8	0.007
ppie	550373	peptidylprolyl isomerase E (cyclophilin E)	1.13E-01	1.21E-01	23.5	1.7	0.6	0.007
mrpl12	550391	mitochondrial ribosomal protein L12	1.05E-02	1.21E-01	15.7	8.4	12.1	0.007
zgc:110325	550504	zgc:110325	1.03E-01	2.91E-02	0.9	2.6	1.7	0.007
crlf3	550515	cytokine receptor-like factor 3	3.54E-04	1.01E-01	11.3	15.3	13.3	0.007
sema3c	553130	semaphorin 3c	8.87E-02	1.13E-01	13.3	17.8	15.6	0.007
lmo7a	553341	LIM domain only 7a	4.86E-02	1.38E-01	2.5	2.7	2.6	0.007
aqp11	553476	aquaporin 11	6.96E-02	1.36E-01	2.0	3.8	2.9	0.007
si:dkey-251i10.3	553498	si:dkey-251i10.3	6.56E-02	1.16E-01	21.7	12.0	16.9	0.007
zgc:109995	553568	zgc:109995	1.48E-01	2.80E-02	22.8	1.8	0.3	0.007
acbd5a	553674	acyl-Coenzyme A binding domain containing 5a	1.39E-02	1.47E-01	11.8	14.2	13.0	0.007
zgc:112294	553704	zgc:112294	1.51E-02	1.04E-01	16.7	15.7	16.2	0.007
pcdh1g3	553987	protocadherin 1 gamma 3	1.32E-01	3.56E-02	14.7	16.8	15.8	0.007
noc4l	554096	nucleolar complex associated 4 homolog (S. cerevisiae)	5.55E-02	1.47E-01	0.7	2.2	1.5	0.007
ctssb.1	554157	cathepsin S, b.1	6.19E-03	1.03E-01	15.7	18.5	17.1	0.007
myo1e	555285	myosin IE	1.04E-01	1.34E-04	11.7	12.0	11.9	0.007
LOC555552	555552	rho-related GTP-binding protein RhoU-like	1.04E-01	4.61E-02	15.7	16.5	16.1	0.007
LOC555692	555692	G patch domain containing 4-like	7.81E-02	1.13E-01	22.0	23.3	22.7	0.007
lpcat1	555969	lysophosphatidylcholine acyltransferase 1	2.83E-02	1.14E-01	4.3	5.0	4.7	0.007
zgc:194768	556744	zgc:194768	1.39E-01	1.58E-03	7.1	7.2	7.2	0.007
dcun1d4	556765	DCN1, defective in cullin neddylation 1, domain containir	8.71E-02	1.04E-01	15.8	17.5	16.7	0.007

Table S1: Zebrafish circadian oscillating genes

Gene Symbol	Gene ID	Gene Description	Fisher's g test p value (LD)	Fisher's g test p value (DD)	LD Phase	DD Phase	Phase *	FDR **
dnajc10	557858	DnaJ (Hsp40) homolog, subfamily C, member 10	1.18E-01	9.19E-02	16.8	17.0	16.9	0.007
thsd7a	557991	thrombospondin, type I, domain containing 7A	3.93E-02	1.22E-01	18.0	15.7	16.9	0.007
st7l	558013	suppression of tumorigenicity 7 like	3.47E-02	1.36E-01	15.8	18.0	16.9	0.007
si:dkey-256k13.1	558466	si:dkey-256k13.1	9.72E-03	1.12E-01	4.0	3.5	3.8	0.007
LOC558513	558513	similar to Transcription cofactor vestigial-like protein 1 (V	1.48E-01	1.09E-01	0.8	1.7	1.3	0.007
si:ch211-67e16.9	558702	si:ch211-67e16.9	6.47E-02	1.43E-01	20.8	22.5	21.7	0.007
cftr	559080	cystic fibrosis transmembrane conductance regulator, AT	1.15E-01	1.08E-01	20.3	20.3	20.3	0.007
zgc:158309	559124	zgc:158309	4.08E-02	1.07E-01	16.0	16.7	16.4	0.007
LOC559127	559127	similar to AWKS9372	1.15E-01	4.90E-02	0.8	1.5	1.2	0.007
si:ch211-203k16.3	559151	si:ch211-203k16.3	6.61E-02	1.46E-01	21.2	21.7	21.5	0.007
im:7152348	559250	im:7152348	4.46E-03	1.29E-01	3.7	4.0	3.9	0.007
LOC559268	559268	novel protein similar to vertebrate solute carrier family 4,	1.50E-01	1.87E-02	19.3	22.0	20.7	0.007
kbtbd12	559380	kelch repeat and BTB (POZ) domain containing 12	2.44E-03	1.05E-01	14.3	16.2	15.3	0.007
mxd4	559531	MAX dimerization protein 4	4.61E-03	1.24E-01	12.8	15.5	14.2	0.007
aggf1	559905	angiogenic factor with G patch and FHA domains 1	1.41E-01	1.19E-01	20.5	20.2	20.4	0.007
slc47a2	560024	solute carrier family 47, member 2	1.87E-03	1.02E-01	3.7	3.1	3.4	0.007
dcaf5	560257	ddb1 and cul4 associated factor 5	3.55E-03	1.20E-01	14.8	17.0	15.9	0.007
LOC560530	560530	somatostatin receptor type 2-like	1.16E-01	2.36E-02	0.2	2.0	1.1	0.007
LOC561494	561494	similar to interleukin 17 receptor	4.92E-02	1.47E-01	13.0	15.8	14.4	0.007
ddx59	562123	DEAD (Asp-Glu-Ala-Asp) box polypeptide 59	1.03E-01	5.71E-02	13.8	16.2	15.0	0.007
mrps5	562703	mitochondrial ribosomal protein S5	1.38E-01	2.13E-02	23.2	1.7	0.5	0.007
mxra8a	562784	matrix-remodelling associated 8a	1.93E-03	1.21E-01	13.5	15.9	14.7	0.007
mavs	562867	mitochondrial antiviral signaling protein	5.25E-02	1.06E-01	21.7	1.6	23.6	0.007
wu:fb09b10	563544	wu:fb09b10	1.67E-03	1.20E-01	21.3	18.8	20.1	0.007
mcat	564111	malonyl CoA:ACP acyltransferase (mitochondrial)	9.63E-02	1.35E-01	20.2	23.2	21.7	0.007
wu:fc06d01	564921	wu:fc06d01	1.07E-01	1.27E-01	12.5	15.2	13.9	0.007
lrrc4.1	565021	leucine rich repeat containing 4.1	1.24E-01	9.95E-02	20.4	19.0	19.7	0.007
hsp90a.2	565155	heat shock protein 90-alpha 2	1.97E-02	1.14E-01	4.3	4.3	4.3	0.007
LOC565371	565371	novel protein similar to Down syndrome cell adhesion mo	1.22E-01	1.86E-02	15.0	16.3	15.7	0.007
si:dkey-30j22.5	565465	si:dkey-30j22.5	1.34E-01	3.64E-02	6.5	6.0	6.3	0.007
LOC565707	565707	hypothetical LOC565707	2.07E-02	1.09E-01	15.3	17.7	16.5	0.007

Table S1: Zebrafish circadian oscillating genes

Gene Symbol	Gene ID	Gene Description	Fisher's g test p value (LD)	Fisher's g test p value (DD)	LD Phase	DD Phase	Phase *	FDR **
zgc:123280	565964	zgc:123280	2.93E-02	1.26E-01	2.4	2.2	2.3	0.007
tecra	566085	trans-2,3-enoyl-CoA reductase a	2.27E-02	1.29E-01	5.8	7.2	6.5	0.007
med12	566632	mediator of RNA polymerase II transcription, subunit 12 I	1.05E-01	5.92E-02	13.8	17.2	15.5	0.007
LOC566817	566817	glycine N-acyltransferase protein Keg1-like	1.05E-01	7.16E-02	2.0	2.3	2.2	0.007
LOC566845	566845	hypothetical protein LOC566845	1.01E-01	3.15E-03	4.3	3.3	3.8	0.007
LOC566922	566922	gamma-aminobutyric acid receptor subunit beta-3-like	5.18E-02	1.21E-01	1.0	2.5	1.8	0.007
acsl3a	567157	acyl-CoA synthetase long-chain family member 3a	1.24E-02	1.21E-01	3.0	2.7	2.9	0.007
LOC567547	567547	5,10-methylenetetrahydrofolate reductase (NADPH)-like	1.27E-01	1.79E-02	2.9	3.8	3.4	0.007
LOC568142	568142	TAK1-like protein-like	1.34E-01	4.36E-02	16.8	18.2	17.5	0.007
slc44a5a	568158	solute carrier family 44, member 5a	3.16E-02	1.01E-01	17.0	16.3	16.7	0.007
LOC568645	568645	probable polypeptide N-acetylgalactosaminyltransferase	2.39E-02	1.10E-01	20.3	22.3	21.3	0.007
cyp2ad3	569245	cytochrome P450, family 2, subfamily AD, polypeptide 3	2.37E-03	1.31E-01	5.4	6.3	5.9	0.007
si:ch211-107m4.1	569278	si:ch211-107m4.1	1.27E-01	3.56E-02	13.3	11.3	12.3	0.007
aadacl4	569798	arylacetamide deacetylase-like 4	8.44E-03	1.24E-01	5.2	4.8	5.0	0.007
mrps18c	569841	mitochondrial ribosomal protein S18C	1.44E-01	9.97E-02	1.8	1.5	1.7	0.007
alkbh5	570158	alkB, alkylation repair homolog 5 (E. coli)	9.36E-02	1.42E-01	19.9	20.3	20.1	0.007
LOC570267	570267	hypothetical protein LOC570267	1.30E-01	1.18E-01	11.9	2.1	7.0	0.007
abcg2b	570453	ATP-binding cassette, sub-family G (WHITE), member 2I	1.02E-01	4.51E-02	0.2	0.8	0.5	0.007
uckl1	570547	uridine-cytidine kinase 1-like 1	1.21E-05	1.20E-01	3.0	1.7	2.4	0.007
ankha	571148	ankylosis, progressive homolog a	1.49E-01	1.47E-01	3.5	3.0	3.3	0.007
si:dkey-24l11.1	571287	si:dkey-24l11.1	5.59E-03	1.49E-01	0.2	1.7	0.9	0.007
kdm2aa	571851	lysine (K)-specific demethylase 2Aa	1.27E-02	1.46E-01	0.7	2.0	1.4	0.007
zgc:112300	572226	zgc:112300	3.62E-02	1.25E-01	11.8	15.0	13.4	0.007
bbox1	572649	butyrobetaine (gamma), 2-oxoglutarate dioxygenase 1 (g	1.00E-01	1.40E-01	5.2	5.3	5.2	0.007
inppl1b	572691	inositol polyphosphate phosphatase-like	4.33E-03	1.23E-01	14.3	16.5	15.4	0.007
psmb3	573095	proteasome (prosome, macropain) subunit, beta type, 3	1.18E-01	1.17E-01	19.8	21.5	20.7	0.007
zgc:122979	641576	zgc:122979	6.83E-04	1.17E-01	1.2	1.7	1.5	0.007
syn2b	641578	synapsin IIb	1.91E-02	1.30E-01	0.8	20.3	22.6	0.007
tchp	678595	trichoplein, keratin filament binding	2.41E-02	1.39E-01	12.3	13.5	12.9	0.007
crfb12	678605	cytokine receptor family member B12	1.20E-01	6.76E-02	6.7	3.7	5.2	0.007
zgc:136871	678634	zgc:136871	1.11E-01	2.73E-03	1.2	3.3	2.3	0.007

Table S1: Zebrafish circadian oscillating genes

Gene Symbol	Gene ID	Gene Description	Fisher's g test p value (LD)	Fisher's g test p value (DD)	LD Phase	DD Phase	Phase *	FDR **
mgst2	723997	microsomal glutathione S-transferase 2	1.43E-01	1.38E-01	17.7	18.2	18.0	0.007
stra6	724007	stimulated by retinoic acid gene 6 homolog (mouse)	7.03E-03	1.20E-01	4.2	3.5	3.9	0.007
zgc:136844	724012	zgc:136844	3.71E-03	1.38E-01	3.8	4.7	4.3	0.007
si:ch211-117n7.7	751622	si:ch211-117n7.7	1.05E-01	5.95E-02	23.2	1.0	0.1	0.007
exosc5	751632	exosome component 5	4.41E-02	1.03E-01	1.3	3.6	2.4	0.007
zgc:152998	751649	zgc:152998	1.28E-01	1.44E-01	0.7	23.8	0.3	0.007
rgs9	767636	regulator of G-protein signaling 9	2.04E-03	1.41E-01	0.6	1.6	1.1	0.007
zgc:153046	767661	zgc:153046	1.02E-01	1.42E-01	5.5	23.2	2.4	0.007
pmt	767699	phosphoethanolamine methyltransferase	6.77E-02	1.49E-01	18.8	19.8	19.3	0.007
zgc:153310	767730	zgc:153310	8.62E-04	1.34E-01	16.2	17.8	17.0	0.007
ptk6b	767734	PTK6 protein tyrosine kinase 6b	9.91E-02	1.11E-01	10.9	11.9	11.4	0.007
zgc:153943	768199	zgc:153943	6.58E-03	1.47E-01	14.8	12.8	13.8	0.007
ppm1h	768291	protein phosphatase, Mg2+/Mn2+ dependent, 1H	1.10E-01	6.14E-02	15.7	15.3	15.5	0.007
agpat2	777624	1-acylglycerol-3-phosphate O-acyltransferase 2 (lysopho	1.11E-01	1.42E-01	4.3	7.5	5.9	0.007
zgc:136531	777714	zgc:136531	1.60E-02	1.40E-01	17.3	16.5	16.9	0.007
oaz2b	790945	ornithine decarboxylase antizyme 2b	9.28E-02	1.21E-01	3.5	3.8	3.7	0.007
zgc:158258	791186	zgc:158258	3.23E-03	1.02E-01	3.6	2.2	2.9	0.007
blzf1	791432	basic leucine zipper nuclear factor 1	2.59E-02	1.50E-01	14.8	16.8	15.8	0.007
enpp6	791915	ectonucleotide pyrophosphatase/phosphodiesterase 6	3.19E-02	1.27E-01	16.2	16.0	16.1	0.007
hspb6	792610	heat shock protein, alpha-crystallin-related, b6	2.47E-02	1.11E-01	0.8	1.5	1.2	0.007
LOC793037	793037	similar to C1q-like adipose specific protein	1.18E-01	8.18E-02	21.8	0.7	23.3	0.007
LOC793262	793262	transmembrane protein 132D-like	1.32E-01	2.01E-02	14.8	16.0	15.4	0.007
si:ch1073-126c3.3	793687	si:ch1073-126c3.3	1.49E-01	2.16E-02	16.7	19.0	17.9	0.007
LOC796407	796407	RIP140-A	1.39E-01	5.43E-02	13.0	14.3	13.6	0.007
si:dkey-242k1.6	797143	si:dkey-242k1.6	7.50E-02	1.34E-01	14.0	14.5	14.3	0.007
abcb11a	797269	ATP-binding cassette, sub-family B (MDR/TAP), membe	5.26E-03	1.15E-01	20.3	23.2	21.8	0.007
LOC797982	797982	disabled homolog 2-like	2.27E-03	1.32E-01	14.5	16.2	15.4	0.007
cdx4	798281	caudal type homeo box transcription factor 4	1.17E-02	1.11E-01	19.8	21.5	20.7	0.007
spata17	799739	spermatogenesis-associated 17	1.25E-01	1.38E-01	16.8	20.0	18.4	0.007
wu:fb66f03	100000085	wu:fb66f03	5.97E-02	1.01E-01	16.2	17.3	16.8	0.007
ltn1	100000122	listerin E3 ubiquitin protein ligase 1	1.45E-01	4.51E-02	21.7	22.7	22.2	0.007

Table S1: Zebrafish circadian oscillating genes

Gene Symbol	Gene ID	Gene Description	Fisher's g test p value (LD)	Fisher's g test p value (DD)	LD Phase	DD Phase	Phase *	FDR **
parp6b	100002364	poly (ADP-ribose) polymerase family, member 6b	1.18E-01	2.45E-02	12.3	14.7	13.5	0.007
tcirg1	100003139	T-cell, immune regulator 1, ATPase, H+ transporting, lys	1.27E-02	1.06E-01	14.2	15.2	14.7	0.007
dis3	100003553	DIS3 mitotic control homolog (S. cerevisiae)	1.11E-01	3.41E-03	0.2	3.0	1.6	0.007
wu:fb51a10	100004396	wu:fb51a10	9.19E-02	1.02E-01	22.8	21.0	21.9	0.007
sh3gl1a	100005753	SH3-domain GRB2-like 1a	9.27E-03	1.18E-01	8.3	9.2	8.8	0.007
zgc:173619	100006880	zgc:173619	8.82E-02	1.34E-01	7.8	7.2	7.5	0.007
paqr5b	100007661	progestin and adipoQ receptor family member Vb	1.48E-01	1.46E-02	2.5	4.2	3.4	0.007
LOC100007990	100007990	solute carrier family 7 (cationic amino acid transporter, y-	1.70E-02	1.01E-01	14.7	16.8	15.8	0.007
hbl3	100008009	hexose-binding lectin 3	1.11E-01	7.40E-02	3.0	3.5	3.3	0.007
zgc:158677	100009626	zgc:158677	7.49E-03	1.31E-01	0.5	0.3	0.4	0.007
zgc:158846	100009654	zgc:158846	1.30E-02	1.20E-01	14.7	15.3	15.0	0.007
zgc:162228	100037363	zgc:162228	5.94E-03	1.12E-01	12.8	13.0	12.9	0.007
f2rl1.2	100073342	coagulation factor II (thrombin) receptor-like 1.2	1.20E-01	3.73E-02	10.8	16.5	13.7	0.007
ifnphi3	100126803	interferon phi 3	1.05E-01	1.46E-01	10.2	9.7	10.0	0.007
gb:cn833218	100190888	expressed sequence CN833218	4.30E-02	1.02E-01	2.3	2.3	2.3	0.007
csf3	100190920	CSF3 protein	9.49E-03	1.10E-01	22.3	23.2	22.8	0.007
wdr89	100272000	WD repeat domain 89	1.03E-01	1.23E-01	23.2	1.3	0.3	0.007
LOC100329277	100329277	hypothetical protein LOC100329277	5.69E-03	1.35E-01	0.2	1.3	0.8	0.007
LOC100330109	100330109	growth arrest-specific protein 1-like	1.11E-01	5.32E-02	14.5	16.3	15.4	0.007
LOC100330227	100330227	SH2 domain containing 4A-like	1.05E-01	1.11E-01	12.7	17.3	15.0	0.007
LOC100330448	100330448	twinkle protein, mitochondrial-like	3.51E-03	1.02E-01	0.5	2.5	1.5	0.007
LOC100331554	100331554	sugar phosphate exchanger 3-like	3.96E-02	1.35E-01	18.5	20.3	19.4	0.007
exog	100331954	nuclease EXOG, mitochondrial-like	5.46E-02	1.07E-01	1.8	3.2	2.5	0.007
LOC100332380	100332380	LAG1 longevity assurance homolog 2-like	1.08E-02	1.04E-01	14.8	16.2	15.5	0.007
LOC100333973	100333973	CUGBP Elav-like family member 3-like	3.15E-02	1.36E-01	17.0	16.8	16.9	0.007
skia	30101	nuclear oncoprotein skia	1.67E-01	1.32E-01	18.5	15.5	17.0	0.012
foxd5	30524	forkhead box D5	1.91E-01	2.77E-02	12.3	16.5	14.4	0.012
pax6a	30567	paired box gene 6a	1.32E-01	1.57E-01	15.3	15.7	15.5	0.012
npy4r	30717	neuropeptide Y receptor Y4	1.48E-01	1.63E-01	16.2	13.7	15.0	0.012
gbp	30719	glycogen synthase kinase binding protein	5.09E-02	1.88E-01	1.7	2.5	2.1	0.012
gch2	64263	GTP cyclohydrolase 2	1.71E-01	4.60E-02	2.8	5.0	3.9	0.012

Table S1: Zebrafish circadian oscillating genes

Gene Symbol	Gene ID	Gene Description	Fisher's g test p value (LD)	Fisher's g test p value (DD)	LD Phase	DD Phase	Phase *	FDR **
atp1a1	64612	ATPase, Na+/K+ transporting, alpha 1 polypeptide	7.19E-03	1.82E-01	1.7	2.2	2.0	0.012
ctsd	65225	cathepsin D	1.59E-01	1.34E-01	15.5	17.0	16.3	0.012
cldnc	81582	claudin c	9.89E-02	1.66E-01	12.8	15.3	14.1	0.012
clica	84040	chloride intracellular channel a	3.37E-02	1.62E-01	3.7	5.8	4.8	0.012
vars	114427	valyl-tRNA synthetase	1.63E-02	1.73E-01	23.3	0.3	23.8	0.012
zp2.2	114438	zona pellucida glycoprotein 2.2	1.88E-01	4.38E-02	2.3	2.2	2.3	0.012
ncam1b	114442	neural cell adhesion molecule 1b	2.12E-03	1.52E-01	2.5	1.8	2.2	0.012
atp1b2b	114457	ATPase, Na+/K+ transporting, beta 2b polypeptide	1.52E-02	1.80E-01	1.0	1.1	1.1	0.012
rab2a	140617	RAB2A, member RAS oncogene family	2.00E-01	1.03E-01	19.3	0.3	21.8	0.012
cyp1a	140634	cytochrome P450, family 1, subfamily A	9.93E-02	1.94E-01	22.7	0.8	23.8	0.012
psap	140811	prosaposin	3.32E-02	1.60E-01	16.4	18.1	17.2	0.012
ptprf	140819	protein tyrosine phosphatase, receptor type, F	1.83E-01	1.27E-01	17.1	17.1	17.1	0.012
mcm3	192323	MCM3 minichromosome maintenance deficient 3 (S. cer	1.60E-01	1.95E-01	2.7	4.2	3.5	0.012
gpia	246094	glucose phosphate isomerase a	1.88E-01	2.10E-02	5.5	5.5	5.5	0.012
ptgs1	246226	prostaglandin-endoperoxide synthase 1	1.73E-01	1.17E-01	8.7	8.2	8.5	0.012
quo	321031	quattro	1.52E-03	2.00E-01	14.0	15.7	14.9	0.012
bmi1	321505	B lymphoma Mo-MLV insertion region 1	2.21E-03	1.55E-01	16.4	17.3	16.8	0.012
exoc1	322338	exocyst complex component 1	9.53E-02	1.54E-01	13.5	14.0	13.8	0.012
hsd17b12b	322626	hydroxysteroid (17-beta) dehydrogenase 12b	8.91E-02	1.99E-01	1.7	2.0	1.9	0.012
dnajc5ga	322863	DnaJ (Hsp40) homolog, subfamily C, member 5 gamma	1.12E-01	1.71E-01	11.6	11.2	11.4	0.012
zbtb16a	323269	zinc finger and BTB domain containing 16a	1.92E-01	1.86E-01	16.2	14.3	15.3	0.012
dnaja1l	323922	DnaJ (Hsp40) homolog, subfamily A, member 1, like	1.94E-01	5.15E-02	4.8	5.0	4.9	0.012
tnika	325042	TRAF2 and NCK interacting kinase a	1.66E-02	1.75E-01	14.2	13.8	14.0	0.012
sap30l	327079	sap30-like	1.91E-02	1.97E-01	21.2	21.5	21.4	0.012
fam46c	327154	family with sequence similarity 46, member C	4.83E-02	1.55E-01	11.6	1.5	6.6	0.012
pcmtd1	327176	protein-L-isoaspartate (D-aspartate) O-methyltransferase	1.83E-01	8.77E-02	13.5	15.3	14.4	0.012
zgc:66160	327299	zgc:66160	4.83E-02	1.81E-01	15.0	16.8	15.9	0.012
usp42	327528	ubiquitin specific peptidase 42	1.24E-02	1.81E-01	18.6	19.2	18.9	0.012
zfp36l2	333941	zinc finger protein 36, C3H type-like 2	9.28E-03	1.90E-01	6.7	9.2	8.0	0.012
xpo6	333980	exportin 6	1.83E-01	1.89E-01	19.3	19.3	19.3	0.012
c20orf24	334006	c20orf24 homolog (H. sapiens)	1.91E-01	2.69E-02	12.4	2.5	7.5	0.012

Table S1: Zebrafish circadian oscillating genes

Gene Symbol	Gene ID	Gene Description	Fisher's g test p value (LD)	Fisher's g test p value (DD)	LD Phase	DD Phase	Phase *	FDR **
plk3	334202	polo-like kinase 3 (Drosophila)	4.48E-02	1.63E-01	6.3	3.0	4.7	0.012
clca1	334289	chloride channel accessory 1	5.47E-02	1.98E-01	17.1	17.3	17.2	0.012
wu:fa04e07	334823	wu:fa04e07	1.57E-01	1.29E-01	23.3	1.3	0.3	0.012
serpinb1l4	335229	serpin peptidase inhibitor, clade B (ovalbumin), member	3.03E-02	1.91E-01	0.5	2.3	1.4	0.012
rbp4l	335651	retinol binding protein 4, like	1.88E-01	7.27E-02	4.5	23.8	2.2	0.012
itm2cb	335863	integral membrane protein 2Cb	7.96E-03	1.61E-01	0.2	2.0	1.1	0.012
ppdpfb	336303	pancreatic progenitor cell differentiation and proliferation	1.41E-01	1.78E-01	13.2	15.0	14.1	0.012
glulb	336473	glutamate-ammonia ligase (glutamine synthase) b	1.94E-01	1.12E-01	6.8	5.8	6.3	0.012
psmc1a	336786	proteasome (prosome, macropain) 26S subunit, ATPase	8.16E-02	1.81E-01	18.7	20.3	19.5	0.012
zgc:56419	336797	zgc:56419	6.56E-02	1.85E-01	0.4	2.0	1.2	0.012
si:dkey-11e23.5	336888	si:dkey-11e23.5	1.32E-02	1.68E-01	0.8	1.0	0.9	0.012
stat6	337413	signal transducer and activator of transcription 6, interleu	1.50E-01	1.20E-01	12.5	12.3	12.4	0.012
mcm4	337598	MCM4 minichromosome maintenance deficient 4, mitotir	9.59E-02	1.91E-01	2.5	4.2	3.4	0.012
impdh1b	338306	inosine 5'-phosphate dehydrogenase 1b	9.77E-02	1.96E-01	14.6	15.7	15.2	0.012
gmnn	368320	geminin, DNA replication inhibitor	8.89E-02	1.78E-01	4.0	5.3	4.7	0.012
pkp3	368397	plakophilin 3	9.37E-02	1.59E-01	3.5	5.5	4.5	0.012
pr2y4l	368435	pyrimidinergic receptor P2Y, G-protein coupled, 4-like	1.73E-01	9.68E-02	14.0	16.2	15.1	0.012
pa2g4a	368737	proliferation-associated 2G4, a	1.61E-01	1.34E-02	0.2	3.3	1.8	0.012
hig1	373084	hypoxia induced gene 1	1.91E-03	1.86E-01	2.7	1.8	2.3	0.012
p4ha2	373132	procollagen-proline, 2-oxoglutarate 4-dioxygenase (prolir	8.24E-02	1.92E-01	2.0	1.1	1.6	0.012
mmp14a	373882	matrix metalloproteinase 14a (membrane-inserted)	1.63E-01	1.65E-01	16.8	18.5	17.7	0.012
tkt	378713	transketolase	8.95E-02	1.54E-01	1.8	3.7	2.8	0.012
rab32a	378969	RAB32a, member RAS oncogene family	7.53E-02	1.93E-01	13.1	15.9	14.5	0.012
ldlr	387529	low density lipoprotein receptor	1.82E-01	1.02E-01	17.0	18.0	17.5	0.012
dcaf13	393097	ddb1 and cul4 associated factor 13	1.58E-01	3.37E-02	23.4	1.9	0.6	0.012
ccbl2	393315	cysteine conjugate-beta lyase 2	5.31E-02	1.62E-01	5.5	5.5	5.5	0.012
coro6	393367	coronin 6	1.63E-01	3.12E-02	20.8	23.2	22.0	0.012
zgc:63466	393404	zgc:63466	1.96E-01	1.96E-01	13.7	16.7	15.2	0.012
agxt2l1	393421	alanine-glyoxylate aminotransferase 2-like 1	1.54E-01	5.28E-03	4.2	4.5	4.4	0.012
ptpn11b	393426	protein tyrosine phosphatase, non-receptor type 11, b	4.54E-02	1.68E-01	3.5	2.9	3.2	0.012
ube3c	393445	ubiquitin protein ligase E3C	5.34E-02	1.68E-01	13.0	17.0	15.0	0.012

Table S1: Zebrafish circadian oscillating genes

Gene Symbol	Gene ID	Gene Description	Fisher's g test p value (LD)	Fisher's g test p value (DD)	LD Phase	DD Phase	Phase *	FDR **
slc35d1b	393447	solute carrier family 35 (UDP-glucuronic acid/UDP-N-ace	1.80E-01	1.18E-01	0.4	3.1	1.7	0.012
zgc:66455	393502	zgc:66455	1.56E-01	1.40E-01	22.8	22.3	22.6	0.012
zgc:65873	393514	zgc:65873	4.91E-02	1.83E-01	16.5	17.2	16.9	0.012
polr1c	393538	polymerase (RNA) I polypeptide C	1.74E-01	8.23E-02	23.7	1.7	0.7	0.012
zgc:63840	393599	zgc:63840	1.47E-01	1.95E-01	17.2	17.7	17.5	0.012
thyn1	393630	thymocyte nuclear protein 1	1.66E-01	1.91E-01	0.5	2.8	1.6	0.012
tbc1d7	393750	TBC1 domain family, member 7	1.09E-01	1.93E-01	15.2	17.2	16.2	0.012
rprmb	393779	reprimo, TP53 dependent G2 arrest mediator candidate l	1.78E-01	1.74E-01	20.3	0.2	22.3	0.012
pde6c	393845	phosphodiesterase 6C, cGMP-specific, cone, alpha prim	5.83E-02	1.84E-01	0.4	2.2	1.3	0.012
zgc:77739	393870	zgc:77739	1.54E-01	1.09E-02	5.0	6.0	5.5	0.012
kcmf1	393927	potassium channel modulatory factor 1	1.80E-01	1.77E-01	16.5	20.2	18.4	0.012
cdc34a	393933	cell division cycle 34 homolog (S. cerevisiae) a	7.69E-02	1.60E-01	15.4	18.1	16.7	0.012
psmc2	393941	proteasome (prosome, macropain) 26S subunit, ATPase	1.94E-01	5.80E-03	17.0	19.2	18.1	0.012
ankrd10a	393955	ankyrin repeat domain 10a	1.98E-01	1.80E-01	12.5	11.5	12.0	0.012
zgc:56548	393989	zgc:56548	1.68E-01	1.71E-01	16.8	17.2	17.0	0.012
shmt1	394021	serine hydroxymethyltransferase 1 (soluble)	1.78E-02	1.69E-01	4.8	5.7	5.2	0.012
ephx1	394043	epoxide hydrolase 1, microsomal (xenobiotic)	2.53E-02	1.69E-01	6.8	7.0	6.9	0.012
snapc3	394090	small nuclear RNA activating complex, polypeptide 3	1.67E-01	2.83E-02	23.7	1.7	0.7	0.012
chic2	394095	cysteine-rich hydrophobic domain 2	1.18E-02	1.53E-01	14.8	16.2	15.5	0.012
dctn2	394141	dynactin 2 (p50)	1.13E-01	1.86E-01	16.3	17.6	16.9	0.012
grina	394183	glutamate receptor, ionotropic, N-methyl D-aspartate-ass	3.16E-03	1.63E-01	14.7	15.5	15.1	0.012
mmadhc	402886	methylmalonic aciduria (cobalamin deficiency) cblD type,	6.52E-02	1.73E-01	6.0	4.2	5.1	0.012
bcor	402921	BCL6 co-repressor	1.85E-01	7.37E-02	18.2	19.0	18.6	0.012
txn2	402938	thioredoxin 2	7.71E-02	1.67E-01	3.2	3.0	3.1	0.012
ppapdc3	402958	phosphatidic acid phosphatase type 2 domain containing	9.37E-03	1.63E-01	14.8	16.3	15.6	0.012
ppa2	402961	pyrophosphatase (inorganic) 2	1.16E-01	1.55E-01	5.5	0.3	2.9	0.012
zgc:77197	404607	zgc:77197	3.22E-02	1.98E-01	17.7	18.4	18.0	0.012
fam49bb	404610	family with sequence similarity 49, member Bb	1.82E-01	9.26E-02	4.7	4.0	4.4	0.012
hoga1	405806	4-hydroxy-2-oxoglutarate aldolase 1	6.91E-02	1.53E-01	23.0	23.5	23.3	0.012
msrb2	405857	methionine sulfoxide reductase B2	1.11E-01	1.90E-01	15.7	14.0	14.9	0.012
nrp2a	405901	neuropilin 2a	5.19E-04	1.57E-01	18.7	17.3	18.0	0.012

Table S1: Zebrafish circadian oscillating genes

Gene Symbol	Gene ID	Gene Description	Fisher's g test p value (LD)	Fisher's g test p value (DD)	LD Phase	DD Phase	Phase *	FDR **
hlxb9la	406205	homeo box HB9 like a	1.92E-01	2.99E-02	1.2	3.0	2.1	0.012
grb2	406308	growth factor receptor-bound protein 2	1.34E-01	1.74E-01	3.0	22.8	0.9	0.012
wdr24	406336	WD repeat domain 24	1.51E-01	1.28E-01	11.0	14.0	12.5	0.012
nipa2	406399	non imprinted in Prader-Willi/Angelman syndrome 2 (hun	2.64E-02	1.77E-01	1.2	2.2	1.7	0.012
marcksl1	406407	MARCKS-like 1	1.68E-01	9.41E-02	14.7	13.7	14.2	0.012
cyb5a	406409	cytochrome b5 type A (microsomal)	1.94E-01	4.87E-02	2.7	4.2	3.4	0.012
ptges3a	406449	prostaglandin E synthase 3a (cytosolic)	1.96E-01	1.87E-01	3.0	4.5	3.8	0.012
gdpd3	406482	glycerophosphodiester phosphodiesterase domain conta	2.64E-02	1.92E-01	5.3	6.2	5.8	0.012
rnft1	406509	ring finger protein, transmembrane 1	1.04E-01	1.60E-01	12.7	14.0	13.4	0.012
cbwd	406536	COBW domain containing	1.68E-01	5.75E-02	0.5	3.5	2.0	0.012
ppt1	406648	palmitoyl-protein thioesterase 1 (ceroid-lipofuscinosis, ne	1.38E-01	2.00E-01	14.0	18.3	16.2	0.012
got2a	406688	glutamic-oxaloacetic transaminase 2a, mitochondrial (as	1.64E-01	1.37E-01	6.8	7.3	7.0	0.012
pgd	406762	phosphogluconate hydrogenase	3.66E-02	1.85E-01	3.3	3.3	3.3	0.012
cul1b	406816	cullin 1b	1.10E-01	1.56E-01	14.3	16.7	15.5	0.012
ift81	432390	intraflagellar transport 81 homolog	4.48E-02	1.54E-01	13.8	11.9	12.9	0.012
trmt61a	436593	tRNA methyltransferase 61 homolog A (S. cerevisiae)	1.44E-01	1.91E-01	11.5	12.8	12.1	0.012
bpnt1	436627	bisphosphate nucleotidase 1	1.67E-01	7.53E-02	3.0	5.2	4.1	0.012
zgc:92655	436701	zgc:92655	7.12E-04	1.89E-01	0.8	1.9	1.3	0.012
fam189a1	436705	family with sequence similarity 189, member A1	1.79E-01	1.70E-01	17.3	18.8	18.1	0.012
timm13	436738	translocase of inner mitochondrial membrane 13 homolo	3.98E-02	1.75E-01	0.6	2.6	1.6	0.012
hccsa	436771	holocytochrome c synthetase a	1.04E-01	1.55E-01	4.5	1.0	2.8	0.012
mrpl46	436791	mitochondrial ribosomal protein L46	4.94E-02	1.89E-01	0.0	1.8	0.9	0.012
zgc:92912	436805	zgc:92912	1.96E-01	1.63E-01	16.3	13.3	14.8	0.012
mrpl11	436829	mitochondrial ribosomal protein L11	4.45E-03	1.93E-01	1.3	2.2	1.8	0.012
pdlim5	436927	PDZ and LIM domain 5	1.68E-01	1.60E-01	10.5	7.7	9.1	0.012
ttc33	436995	tetratricopeptide repeat domain 33	1.02E-01	1.97E-01	13.0	15.7	14.4	0.012
zdhhc3	436998	zinc finger, DHHC-type containing 3	1.85E-01	9.23E-03	5.0	3.8	4.4	0.012
b4galt7	445022	xylosylprotein beta 1,4-galactosyltransferase, polypeptid	1.63E-01	8.58E-02	0.2	22.3	23.3	0.012
olfm1b	445092	olfactomedin 1b	7.51E-02	1.71E-01	16.8	15.7	16.3	0.012
zgc:101116	445165	zgc:101116	2.93E-03	1.82E-01	18.3	19.5	18.9	0.012
jagn1b	445190	jagunal homolog 1b (Drosophila)	1.85E-01	3.46E-03	23.7	2.2	1.0	0.012

Table S1: Zebrafish circadian oscillating genes

Gene Symbol	Gene ID	Gene Description	Fisher's g test p value (LD)	Fisher's g test p value (DD)	LD Phase	DD Phase	Phase *	FDR **
ifrd2	445227	interferon-related developmental regulator 2	1.99E-01	1.60E-01	21.3	11.9	16.6	0.012
picalml	445286	phosphatidylinositol binding clathrin assembly protein, lik	1.77E-01	6.78E-02	18.5	17.7	18.1	0.012
tcf25	445817	transcription factor 25 (basic helix-loop-helix)	1.99E-01	1.81E-01	22.5	1.7	0.1	0.012
zgc:92316	447840	zgc:92316	1.32E-02	1.64E-01	4.7	3.2	4.0	0.012
zgc:101783	447883	zgc:101783	3.67E-02	1.96E-01	12.8	16.7	14.8	0.012
zgc:101565	447903	zgc:101565	1.38E-02	1.57E-01	0.3	1.3	0.8	0.012
gpsm2l	449665	G-protein signalling modulator 2, like	9.05E-02	1.93E-01	15.3	17.3	16.3	0.012
hspb7	450019	heat shock protein family, alpha-crystallin-related, b7	1.57E-01	1.22E-01	4.3	2.5	3.4	0.012
tspan5	450026	tetraspanin 5	1.70E-01	7.96E-02	15.2	16.5	15.9	0.012
phyhd1	492804	phytanoyl-CoA dioxygenase domain containing 1	3.24E-02	1.86E-01	5.8	7.0	6.4	0.012
slc43a2b	494042	solute carrier family 43, member 2b	5.88E-02	1.51E-01	2.7	3.8	3.3	0.012
tectb	494051	tectorin beta	1.15E-01	1.83E-01	3.2	3.9	3.5	0.012
ephx2	494099	epoxide hydrolase 2, cytoplasmic	7.73E-03	1.95E-01	6.2	4.2	5.2	0.012
si:dkey-13a21.4	494576	si:dkey-13a21.4	1.75E-01	1.49E-01	14.2	16.2	15.2	0.012
im:7137379	497345	im:7137379	1.51E-01	1.66E-01	4.5	7.5	6.0	0.012
ndufaf1	503714	NADH dehydrogenase (ubiquinone) 1 alpha subcomplex	1.65E-01	1.73E-01	0.8	1.5	1.2	0.012
wars2	503745	tryptophanyl tRNA synthetase 2, mitochondrial	7.08E-04	1.95E-01	1.0	3.5	2.3	0.012
zgc:112964	503764	zgc:112964	1.79E-03	1.82E-01	13.2	14.8	14.0	0.012
ak5l	548336	adenylate kinase 5, like	1.83E-01	1.69E-01	13.7	15.2	14.5	0.012
ubb	550134	ubiquitin B	1.51E-01	4.91E-02	12.7	15.0	13.9	0.012
kctd12b	550324	potassium channel tetramerisation domain containing 12	6.45E-02	1.91E-01	10.2	12.0	11.1	0.012
ap1s3a	550415	adaptor-related protein complex 1, sigma 3 subunit, a	4.48E-02	1.79E-01	0.2	3.4	1.8	0.012
arl4ca	550444	ADP-ribosylation factor-like 4Ca	5.89E-02	1.83E-01	14.7	14.4	14.5	0.012
spata6	550512	spermatogenesis associated 6	1.62E-01	1.82E-01	10.7	14.7	12.7	0.012
stmn1b	550548	stathmin 1b	7.74E-02	1.57E-01	6.3	6.2	6.3	0.012
pdlim1	550568	PDZ and LIM domain 1 (elfin)	1.52E-04	1.94E-01	7.5	5.8	6.7	0.012
im:6901974	552974	im:6901974	1.65E-01	1.94E-02	20.5	20.5	20.5	0.012
lsr	553218	lipolysis stimulated lipoprotein receptor	1.99E-01	5.96E-02	14.5	14.7	14.6	0.012
zgc:158564	553264	zgc:158564	1.88E-02	1.72E-01	18.7	18.2	18.4	0.012
im:7150454	553298	im:7150454	5.92E-02	1.87E-01	0.0	2.3	1.2	0.012
ctns	553594	cystinosis, nephropathic	5.72E-02	1.96E-01	13.3	15.8	14.6	0.012

Table S1: Zebrafish circadian oscillating genes

Gene Symbol	Gene ID	Gene Description	Fisher's g test p value (LD)	Fisher's g test p value (DD)	LD Phase	DD Phase	Phase *	FDR **
parp12b	553633	poly (ADP-ribose) polymerase family, member 12b	5.60E-02	1.56E-01	14.3	16.5	15.4	0.012
sptlc2	553646	serine palmitoyltransferase, long chain base subunit 2	2.88E-03	1.66E-01	22.2	2.7	0.5	0.012
gins2	554146	GINS complex subunit 2	1.96E-01	9.29E-02	3.5	4.8	4.2	0.012
si:ch211-11m18.2	555268	si:ch211-11m18.2	1.34E-03	1.79E-01	0.0	0.5	0.3	0.012
tspan9	555341	tetraspanin 9	1.86E-02	1.54E-01	16.3	17.5	16.9	0.012
LOC555689	555689	protein FAM115-like	1.05E-03	1.83E-01	4.2	5.3	4.8	0.012
slc5a8l	556156	solute carrier family 5 (iodide transporter), member 8-like	6.90E-02	1.78E-01	20.7	22.2	21.5	0.012
nudt17	556651	nudix (nucleoside diphosphate linked moiety X)-type mot	7.42E-02	1.98E-01	13.9	16.3	15.1	0.012
im:7142942	557319	im:7142942	1.57E-01	1.46E-01	14.8	18.0	16.4	0.012
im:7137300	557360	im:7137300	1.52E-01	1.96E-02	14.4	15.4	14.9	0.012
bbs7	557491	Bardet-Biedl syndrome 7	1.36E-01	1.62E-01	14.8	16.8	15.8	0.012
LOC557634	557634	proline-rich protein 5-like	1.51E-02	1.73E-01	3.7	22.3	1.0	0.012
LOC557782	557782	similar to N-acetylgalactosaminyltransferase	4.39E-02	1.65E-01	16.0	18.5	17.3	0.012
LOC557789	557789	39S ribosomal protein L47, mitochondrial-like	8.30E-02	1.74E-01	23.5	1.3	0.4	0.012
slain2	557946	SLAIN motif family, member 2	1.97E-01	1.28E-01	15.8	19.5	17.7	0.012
surf1	557970	surfeit 1	1.70E-03	1.92E-01	5.2	1.5	3.4	0.012
si:dkeyp-104h9.6	558407	si:dkeyp-104h9.6	2.43E-02	1.97E-01	5.5	5.2	5.4	0.012
anpepa	558452	alanyl (membrane) aminopeptidase a	2.16E-03	1.74E-01	3.0	2.7	2.9	0.012
pipox	558601	pipecolic acid oxidase	1.60E-01	1.44E-01	10.8	9.0	9.9	0.012
wu:fa99c08	558911	wu:fa99c08	1.93E-01	6.54E-02	0.7	2.2	1.5	0.012
ptgis	559148	prostaglandin I2 (prostacyclin) synthase	6.27E-02	1.85E-01	7.0	9.7	8.4	0.012
rgs11	559197	regulator of G-protein signaling 11	7.48E-03	1.86E-01	18.7	19.3	19.0	0.012
zgc:153225	559769	zgc:153225	1.31E-01	1.51E-01	19.7	18.5	19.1	0.012
zgc:162184	559822	zgc:162184	4.95E-03	1.77E-01	3.8	4.1	3.9	0.012
si:dkey-33c12.4	560112	si:dkey-33c12.4	9.07E-02	1.99E-01	2.7	3.6	3.2	0.012
nolc1	561131	nucleolar and coiled-body phosphoprotein 1	3.29E-02	1.54E-01	22.7	7.3	3.0	0.012
gfm1	561840	G elongation factor, mitochondrial 1	1.81E-01	1.80E-01	22.3	10.2	4.2	0.012
neurla	561943	neuralized homolog a (Drosophila)	8.70E-02	1.79E-01	1.3	23.4	0.3	0.012
irg1l	562007	immunoresponsive gene 1, like	1.65E-01	1.00E-01	20.7	21.4	21.0	0.012
cyp4v7	562008	cytochrome P450, family 4, subfamily V, polypeptide 7	1.84E-01	1.55E-01	12.8	15.7	14.3	0.012
enpp1	562399	ectonucleotide pyrophosphatase/phosphodiesterase 1	3.05E-02	1.84E-01	14.2	13.5	13.9	0.012

Table S1: Zebrafish circadian oscillating genes

Gene Symbol	Gene ID	Gene Description	Fisher's g test p value (LD)	Fisher's g test p value (DD)	LD Phase	DD Phase	Phase *	FDR **
tbc1d10a	562628	TBC1 domain family, member 10A	2.81E-02	1.70E-01	2.7	2.7	2.7	0.012
atp11a	562925	ATPase, class VI, type 11A	1.77E-01	9.09E-02	23.7	21.7	22.7	0.012
nr1d4a	563150	nuclear receptor subfamily 1, group D, member 4a	1.76E-01	7.40E-02	19.3	20.3	19.8	0.012
trmt6	563475	tRNA methyltransferase 6 homolog (S. cerevisiae)	1.02E-03	1.51E-01	12.7	12.7	12.7	0.012
si:dkey-31f5.1	563896	si:dkey-31f5.1	1.96E-01	1.74E-01	2.7	3.0	2.9	0.012
si:ch211-210c8.6	564023	si:ch211-210c8.6	3.99E-03	1.73E-01	5.8	5.9	5.8	0.012
si:ch211-133l5.7	564121	si:ch211-133l5.7	4.31E-02	1.67E-01	1.8	2.7	2.3	0.012
ankrd1b	564159	ankyrin repeat domain 1b (cardiac muscle)	8.97E-03	1.85E-01	0.7	2.3	1.5	0.012
st13	564225	suppression of tumorigenicity 13 (colon carcinoma) (Hsp	9.48E-02	1.59E-01	5.7	9.5	7.6	0.012
usp10	564632	ubiquitin specific peptidase 10	5.59E-02	1.50E-01	23.8	3.2	1.5	0.012
mmaa	564760	methylmalonic aciduria (cobalamin deficiency) cblA type	1.59E-01	1.78E-01	19.3	21.5	20.4	0.012
tnfrsf21	564874	tumor necrosis factor receptor superfamily, member 21	4.01E-02	1.97E-01	18.2	17.7	18.0	0.012
LOC565002	565002	interferon-inducible GTPase 5-like	1.82E-01	1.00E-01	5.8	7.0	6.4	0.012
slc2a9l1	565510	solute carrier family 2 (facilitated glucose transporter), m	6.90E-02	1.68E-01	1.9	3.3	2.6	0.012
si:ch211-203b8.3	565995	si:ch211-203b8.3	1.92E-01	1.80E-01	3.0	2.7	2.9	0.012
tmem55a	566642	transmembrane protein 55A	1.71E-01	1.09E-01	14.0	14.5	14.3	0.012
cdc42bpb	567039	CDC42 binding protein kinase beta (DMPK-like)	1.93E-01	7.86E-02	18.0	19.3	18.7	0.012
LOC567074	567074	kynurenine--oxoglutarate transaminase 3-like	5.88E-03	1.63E-01	4.5	4.8	4.7	0.012
smpd2	567548	sphingomyelin phosphodiesterase 2, neutral	1.68E-01	4.05E-02	4.0	2.5	3.3	0.012
si:ch211-251p5.5	567691	si:ch211-251p5.5	1.08E-01	1.77E-01	12.5	15.2	13.9	0.012
zgc:123339	567972	zgc:123339	4.30E-02	1.55E-01	6.2	6.5	6.4	0.012
si:dkey-222f2.1	568321	si:dkey-222f2.1	7.11E-02	2.00E-01	15.5	17.3	16.4	0.012
si:dkey-222f2.8	568423	si:dkey-222f2.8	4.17E-02	1.96E-01	2.8	2.2	2.5	0.012
kazald2	568748	Kazal-type serine peptidase inhibitor domain 2	1.87E-01	1.24E-01	4.5	3.8	4.2	0.012
srpr	569063	signal recognition particle receptor (docking protein)	1.68E-01	3.47E-02	23.2	0.2	23.7	0.012
si:dkey-119o24.1	569072	si:dkey-119o24.1	2.56E-02	1.72E-01	5.3	4.8	5.1	0.012
irak1	569226	interleukin-1 receptor-associated kinase 1	1.62E-01	2.36E-02	11.0	8.0	9.5	0.012
chd7	569471	chromodomain helicase DNA binding protein 7	5.71E-02	1.73E-01	21.4	19.0	20.2	0.012
si:dkey-60a16.3	569589	si:dkey-60a16.3	1.63E-01	1.02E-01	21.8	17.5	19.7	0.012
LOC570074	570074	WD40 repeat-containing protein SMU1-like	6.71E-02	1.56E-01	22.2	0.0	23.1	0.012
LOC570420	570420	protein TSPEAR-like	1.74E-01	4.56E-02	19.8	21.5	20.7	0.012

Table S1: Zebrafish circadian oscillating genes

Gene Symbol	Gene ID	Gene Description	Fisher's g test p value (LD)	Fisher's g test p value (DD)	LD Phase	DD Phase	Phase *	FDR **
si:ch211-197g15.7	571377	si:ch211-197g15.7	1.74E-01	6.21E-02	23.5	20.3	21.9	0.012
wu:fb18f06	572235	wu:fb18f06	3.85E-02	1.67E-01	7.0	7.0	7.0	0.012
si:dkey-83f6.3	572492	si:dkey-83f6.3	4.24E-02	1.97E-01	5.1	4.1	4.6	0.012
dynll2b	572690	dynein, light chain, LC8-type 2b	7.47E-02	1.57E-01	2.3	3.0	2.7	0.012
zgc:100843	573010	zgc:100843	1.90E-01	1.54E-01	10.0	8.2	9.1	0.012
zgc:153411	751688	zgc:153411	5.80E-02	1.93E-01	16.2	18.2	17.2	0.012
fgf10b	751792	fibroblast growth factor 10b	1.32E-03	1.62E-01	12.2	14.8	13.5	0.012
zgc:153206	767788	zgc:153206	1.22E-01	1.68E-01	6.2	3.2	4.7	0.012
zgc:153952	777621	zgc:153952	5.36E-02	1.87E-01	5.3	5.3	5.3	0.012
phyhipla	790943	phytanoyl-CoA 2-hydroxylase interacting protein-like a	1.93E-04	1.53E-01	3.5	4.7	4.1	0.012
zgc:158270	791213	zgc:158270	1.82E-01	1.16E-01	15.8	16.5	16.2	0.012
wnt11	791595	wingless-type MMTV integration site family, member 11	1.04E-01	1.57E-01	11.5	9.6	10.6	0.012
slc39a3	792986	solute carrier family 39 (zinc transporter), member 3	5.02E-02	1.60E-01	13.0	17.3	15.2	0.012
LOC793321	793321	hypothetical protein LOC793321	3.56E-03	1.75E-01	4.0	4.7	4.4	0.012
pdcd11	794079	programmed cell death 11	7.35E-02	1.97E-01	0.6	1.8	1.2	0.012
LOC795086	795086	active breakpoint cluster region-related protein-like	1.79E-01	1.00E-01	19.8	20.3	20.1	0.012
llgl2	795670	lethal giant larvae homolog 2 (Drosophila)	1.94E-01	5.78E-02	14.2	14.0	14.1	0.012
LOC796453	796453	hypothetical LOC796453	4.28E-02	1.94E-01	13.0	14.8	13.9	0.012
LOC796750	796750	C1orf156-like	1.32E-01	1.69E-01	22.9	2.1	0.5	0.012
sidt2	796833	SID1 transmembrane family, member 2	1.72E-01	1.63E-01	16.7	18.2	17.5	0.012
LOC797244	797244	kelch repeat and BTB domain-containing protein 13-like	1.52E-01	1.52E-01	21.5	1.7	23.6	0.012
LOC797409	797409	voltage-dependent calcium channel gamma-5 subunit-lik	1.68E-01	3.57E-02	11.8	11.3	11.6	0.012
zgc:171429	797867	zgc:171429	1.12E-01	1.83E-01	15.2	17.2	16.2	0.012
gemin5	799538	gem (nuclear organelle) associated protein 5	5.52E-04	1.92E-01	21.7	21.8	21.8	0.012
si:dkey-61p9.11	100000781	si:dkey-61p9.11	2.34E-02	1.57E-01	19.7	20.5	20.1	0.012
LOC100000884	100000884	XK-related protein 5-like	6.18E-02	1.59E-01	0.0	2.0	1.0	0.012
LOC100001665	100001665	hypothetical protein LOC100001665	1.93E-01	1.07E-01	0.3	23.3	23.8	0.012
LOC100001880	100001880	up-regulator of cell proliferation-like	1.52E-01	1.33E-01	22.8	0.8	23.8	0.012
zgc:110020	100002293	zgc:110020	1.03E-01	1.58E-01	22.2	23.8	23.0	0.012
p4ha1b	100003675	prolyl 4-hydroxylase, alpha polypeptide I b	8.39E-02	1.74E-01	0.2	2.3	1.3	0.012
LOC100004175	100004175	cyclin-dependent kinase inhibitor 1C-like	8.25E-02	1.57E-01	3.3	3.7	3.5	0.012

Table S1: Zebrafish circadian oscillating genes

Gene Symbol	Gene ID	Gene Description	Fisher's g test p value (LD)	Fisher's g test p value (DD)	LD Phase	DD Phase	Phase *	FDR **
LOC100005267	100005267	RING finger protein 122-like	1.85E-01	1.59E-01	14.8	14.8	14.8	0.012
si:dkey-204a24.6	100005886	si:dkey-204a24.6	1.76E-01	1.77E-01	3.8	3.7	3.8	0.012
gls2	100006303	glutaminase 2 (liver, mitochondrial)	1.54E-01	1.63E-01	7.3	4.8	6.1	0.012
csnk1e	100006858	casein kinase 1, epsilon	1.88E-01	6.99E-02	13.0	15.2	14.1	0.012
zgc:175280	100007793	zgc:175280	1.78E-01	1.03E-01	1.8	3.5	2.7	0.012
kcnc2	100037377	potassium voltage-gated channel, Shaw-related subfamil	7.73E-02	1.81E-01	17.2	18.2	17.7	0.012
prkg2	100125912	protein kinase, cGMP-dependent, type II	1.89E-02	1.55E-01	5.5	3.3	4.4	0.012
btr22	100144555	bloodthirsty-related gene family, member 22	2.45E-03	1.71E-01	13.5	14.7	14.1	0.012
LOC100148772	100148772	uncharacterized protein DKFZp761E198 homolog	9.26E-02	1.92E-01	12.5	14.8	13.7	0.012
LOC100150452	100150452	sodium- and chloride-dependent creatine transporter 1-lil	1.52E-01	1.80E-01	17.0	18.3	17.7	0.012
LOC100151195	100151195	hereditary hemochromatosis protein homolog	1.99E-01	1.26E-01	2.8	3.5	3.1	0.012
LOC100330593	100330593	hypothetical protein LOC100330593	1.96E-02	1.57E-01	2.0	1.5	1.8	0.012
galnt13	100330695	UDP-N-acetyl-alpha-D-galactosamine:polypeptide N-ace	1.27E-01	1.89E-01	19.8	17.8	18.8	0.012
LOC100333914	100333914	c-C motif chemokine 25-like	1.16E-01	1.54E-01	18.5	17.7	18.1	0.012
LOC100334727	100334727	hypothetical protein LOC100334727	1.77E-01	1.58E-01	13.2	17.5	15.4	0.012
qk	30471	quaking	1.37E-01	2.50E-01	23.2	20.2	21.7	0.016
six3a	30635	sine oculis homeobox homolog 3a	2.02E-01	1.11E-01	16.8	18.0	17.4	0.016
gfap	30646	glial fibrillary acidic protein	2.38E-01	1.75E-01	10.5	11.0	10.8	0.016
fabp2	30708	fatty acid binding protein 2, intestinal	1.38E-01	2.45E-01	3.1	6.0	4.6	0.016
tradd	58130	tnfrsf1a-associated via death domain	5.65E-03	2.30E-01	12.2	15.3	13.8	0.016
gart	58141	phosphoribosylglycinamide formyltransferase	2.07E-01	8.92E-02	1.4	3.0	2.2	0.016
robo2	60309	roundabout homolog 2	2.00E-01	2.15E-01	22.2	17.2	19.7	0.016
sec22ba	114464	SEC22 vesicle trafficking protein homolog B (S. cerevisia	6.98E-02	2.25E-01	23.2	0.0	23.6	0.016
tnfsf10l	114466	tumor necrosis factor (ligand) superfamily, member 10 lik	9.30E-02	2.04E-01	15.1	16.9	16.0	0.016
maf	114467	v-maf musculoaponeurotic fibrosarcoma (avian) oncoger	2.18E-01	2.42E-01	11.9	3.1	7.5	0.016
prkci	117507	protein kinase C, iota	7.28E-02	2.15E-01	17.0	22.5	19.8	0.016
jag1a	140421	jagged 1a	6.55E-02	2.42E-01	16.3	17.3	16.8	0.016
psma6a	171585	proteasome (prosome, macropain) subunit, alpha type, 6	4.53E-02	2.29E-01	18.0	19.7	18.9	0.016
atp1a1a.5	245703	ATPase, Na+/K+ transporting, alpha 1a.5 polypeptide	1.15E-01	2.21E-01	17.8	0.0	20.9	0.016
bbs2	259187	Bardet-Biedl syndrome 2	2.25E-01	7.85E-02	21.0	20.2	20.6	0.016
trpm7	280653	transient receptor potential cation channel, subfamily M,	1.58E-01	2.35E-01	19.3	20.3	19.8	0.016

Table S1: Zebrafish circadian oscillating genes

Gene Symbol	Gene ID	Gene Description	Fisher's g test p value (LD)	Fisher's g test p value (DD)	LD Phase	DD Phase	Phase *	FDR **
mcm5	286747	MCM5 minichromosome maintenance deficient 5 (S. cer	2.08E-01	1.96E-01	2.7	4.3	3.5	0.016
apobl	321166	apolipoprotein B, like	2.38E-01	1.30E-01	6.2	8.3	7.3	0.016
phb	321346	prohibitin	1.00E-01	2.18E-01	0.8	2.0	1.4	0.016
ephx1l	322331	epoxide hydrolase 1, like	2.08E-01	5.81E-03	5.9	5.8	5.8	0.016
wu:fb70a12	322680	wu:fb70a12	2.10E-01	8.29E-02	14.2	16.0	15.1	0.016
lasp1	323216	LIM and SH3 protein 1	3.38E-02	2.04E-01	15.1	16.3	15.7	0.016
si:dkeyp-95d10.1	323834	si:dkeyp-95d10.1	1.56E-02	2.41E-01	5.8	6.3	6.1	0.016
wu:fc16h08	324016	wu:fc16h08	2.06E-01	1.64E-01	5.5	6.2	5.9	0.016
zgc:77415	324587	zgc:77415	2.20E-01	1.41E-01	17.3	17.2	17.3	0.016
plaa	324855	phospholipase A2-activating protein	2.32E-01	7.58E-02	16.7	18.3	17.5	0.016
nsun2	325292	NOL1/NOP2/Sun domain family, member 2	1.11E-02	2.18E-01	0.7	3.2	2.0	0.016
si:ch211-150c22.2	325430	si:ch211-150c22.2	2.49E-01	2.21E-01	13.1	16.6	14.9	0.016
wu:fd51h11	325964	wu:fd51h11	2.25E-01	3.82E-02	6.5	4.8	5.7	0.016
ofd1	327216	oral-facial-digital syndrome 1	3.28E-02	2.21E-01	12.5	16.7	14.6	0.016
srp72	327335	signal recognition particle 72	2.11E-01	5.49E-02	22.3	1.0	23.7	0.016
mtmr6	327346	myotubularin related protein 6	5.29E-03	2.30E-01	12.5	12.2	12.4	0.016
kpna4	327373	karyopherin alpha 4 (importin alpha 3)	1.79E-02	2.01E-01	19.8	19.3	19.6	0.016
zc3h15	334307	zinc finger CCCH-type containing 15	1.19E-01	2.16E-01	22.6	1.3	23.9	0.016
wu:fi42h12	334358	wu:fi42h12	1.39E-03	2.10E-01	14.0	19.7	16.9	0.016
nol6	334362	nucleolar protein family 6 (RNA-associated)	2.37E-01	2.00E-01	22.9	1.2	0.0	0.016
zgc:101121	336461	zgc:101121	6.02E-02	2.08E-01	13.5	16.7	15.1	0.016
zgc:63831	336746	zgc:63831	1.27E-02	2.50E-01	13.5	16.3	14.9	0.016
plcd1b	337489	phospholipase C, delta 1b	1.73E-02	2.32E-01	10.8	13.5	12.2	0.016
tph1a	352943	tryptophan hydroxylase 1 (tryptophan 5-monooxygenase	5.46E-04	2.16E-01	16.2	17.3	16.8	0.016
dcp1a	360153	decapping enzyme	2.02E-01	1.61E-01	15.5	13.7	14.6	0.016
penka	368228	proenkephalin a	2.15E-01	1.38E-01	3.6	4.8	4.2	0.016
prickle2	368250	prickle-like 2 (Drosophila)	2.22E-01	6.27E-02	15.5	15.4	15.5	0.016
mycbp2	368439	MYC binding protein 2	5.39E-03	2.11E-01	21.2	19.2	20.2	0.016
pdzk1ip1l	368722	PDZK1 interacting protein 1, like	8.10E-02	2.08E-01	17.5	18.9	18.2	0.016
si:busm1-241h12.4	368857	si:busm1-241h12.4	2.27E-01	3.18E-03	2.8	23.2	1.0	0.016
pip5k2	373124	phosphatidylinositol-4-phosphate 5-kinase, type II	1.29E-03	2.10E-01	14.5	14.7	14.6	0.016

Table S1: Zebrafish circadian oscillating genes

Gene Symbol	Gene ID	Gene Description	Fisher's g test p value (LD)	Fisher's g test p value (DD)	LD Phase	DD Phase	Phase *	FDR **
aktip	378716	akt interacting protein	4.21E-02	2.49E-01	15.7	18.2	17.0	0.016
apc	386762	adenomatosis polyposis coli	2.14E-01	1.31E-02	16.5	12.7	14.6	0.016
ca2	387526	carbonic anhydrase II	1.43E-02	2.48E-01	0.9	2.6	1.7	0.016
sepsecs	393123	Sep (O-phosphoserine) tRNA:Sec (selenocysteine) tRNA	2.08E-01	1.24E-01	17.2	17.7	17.5	0.016
umps	393143	uridine monophosphate synthetase	1.93E-01	2.35E-01	0.2	2.7	1.5	0.016
tigarb	393160	tp53-induced glycolysis and apoptosis regulator b	1.37E-03	2.24E-01	2.0	2.5	2.3	0.016
tpst1l	393171	tyrosylprotein sulfotransferase 1, like	2.37E-01	1.79E-01	18.0	17.8	17.9	0.016
hif1ab	393202	hypoxia-inducible factor 1, alpha subunit (basic helix-loop	5.29E-02	2.48E-01	0.2	22.7	23.5	0.016
zgc:56525	393249	zgc:56525	6.49E-02	2.22E-01	12.8	15.5	14.2	0.016
mrpl45	393277	mitochondrial ribosomal protein L45	3.58E-02	2.13E-01	23.0	21.7	22.4	0.016
tnfaip8l3	393322	tumor necrosis factor, alpha-induced protein 8-like 3	1.93E-02	2.27E-01	3.8	3.7	3.8	0.016
zgc:64141	393353	zgc:64141	1.27E-01	2.14E-01	1.8	3.2	2.5	0.016
gstt1b	393556	glutathione S-transferase theta 1b	5.53E-02	2.48E-01	11.0	11.3	11.2	0.016
zgc:63614	393580	zgc:63614	4.58E-02	2.17E-01	6.5	5.1	5.8	0.016
timm50	393638	translocase of inner mitochondrial membrane 50 homolo	2.31E-01	2.24E-01	22.4	22.3	22.3	0.016
aanat1	393677	arylalkylamine N-acetyltransferase 1	4.09E-03	2.47E-01	19.3	20.0	19.7	0.016
slc22a4	393822	solute carrier family 22 (organic cation/ergothioneine trar	1.21E-01	2.19E-01	2.3	3.3	2.8	0.016
nek4	393987	NIMA (never in mitosis gene a)-related kinase 4	2.28E-02	2.08E-01	11.7	14.0	12.9	0.016
msi2b	394084	musashi homolog 2b (Drosophila)	6.19E-02	2.41E-01	1.7	6.8	4.3	0.016
zgc:55307	394104	zgc:55307	3.54E-02	2.29E-01	20.5	22.3	21.4	0.016
rem1	394149	rad and gem related GTP binding protein 1	9.89E-04	2.30E-01	15.0	16.3	15.7	0.016
bcs1l	394157	BCS1-like (yeast)	2.04E-01	2.28E-01	22.7	0.7	23.7	0.016
tra2b	394172	transformer 2 beta homolog (Drosophila)	1.04E-01	2.39E-01	3.3	2.7	3.0	0.016
st3gal5	394230	ST3 beta-galactoside alpha-2,3-sialyltransferase 5	2.86E-03	2.03E-01	16.3	17.3	16.8	0.016
mapk3	399480	mitogen-activated protein kinase 3	7.55E-02	2.44E-01	23.2	19.2	21.2	0.016
twistnb	402856	TWIST neighbor	4.19E-02	2.01E-01	23.3	1.7	0.5	0.016
gtpbp1l	402863	GTP binding protein 1, like	1.28E-01	2.36E-01	17.0	20.8	18.9	0.016
zgc:77713	402894	zgc:77713	1.04E-01	2.34E-01	13.9	16.4	15.1	0.016
slc30a6	402949	solute carrier family 30 (zinc transporter), member 6	1.01E-01	2.09E-01	15.2	16.5	15.9	0.016
ezrl	403009	ezrin like	3.31E-04	2.13E-01	3.0	4.1	3.5	0.016
slc31a1	403028	solute carrier family 31 (copper transporters), member 1	8.96E-02	2.05E-01	0.4	1.3	0.8	0.016

Table S1: Zebrafish circadian oscillating genes

Gene Symbol	Gene ID	Gene Description	Fisher's g test p value (LD)	Fisher's g test p value (DD)	LD Phase	DD Phase	Phase *	FDR **
tab1	403084	TGF-beta activated kinase 1/MAP3K7 binding protein 1	2.29E-01	6.75E-02	16.2	17.7	17.0	0.016
n6amt2	404619	N-6 adenine-specific DNA methyltransferase 2 (putative)	2.29E-01	1.75E-01	1.0	2.0	1.5	0.016
zgc:56699	405758	zgc:56699	2.15E-01	1.46E-01	11.9	1.7	6.8	0.016
gatad2a	405852	GATA zinc finger domain containing 2A	5.99E-02	2.39E-01	19.7	18.3	19.0	0.016
zgc:85858	405879	zgc:85858	2.32E-01	3.90E-02	22.3	4.2	1.3	0.016
sucla2	406299	succinate-CoA ligase, ADP-forming, beta subunit	1.54E-01	2.06E-01	18.0	20.5	19.3	0.016
dok1b	406361	docking protein 1b	5.00E-02	2.06E-01	14.0	16.7	15.4	0.016
zgc:76871	406376	zgc:76871	2.22E-01	1.44E-01	18.3	18.0	18.2	0.016
yy1b	406394	YY1 transcription factor b	7.39E-02	2.37E-01	16.2	17.3	16.7	0.016
mre11a	406637	meiotic recombination 11 homolog A (S. cerevisiae)	2.08E-01	4.03E-03	2.9	4.1	3.5	0.016
zgc:56304	406683	zgc:56304	1.09E-02	2.01E-01	0.3	2.7	1.5	0.016
tmem184a	406841	transmembrane protein 184a	5.77E-02	2.34E-01	22.7	1.2	24.0	0.016
ptpn4b	407634	protein tyrosine phosphatase, non-receptor type 4, b	1.35E-01	2.26E-01	2.0	3.7	2.8	0.016
zgc:91852	431744	zgc:91852	1.64E-01	2.05E-01	4.0	1.8	2.9	0.016
pdss2	436624	prenyl (decaprenyl) diphosphate synthase, subunit 2	1.49E-01	2.19E-01	23.0	0.8	23.9	0.016
zgc:92670	436691	zgc:92670	5.19E-03	2.44E-01	22.3	22.2	22.3	0.016
adipor1a	436740	adiponectin receptor 1a	5.08E-02	2.07E-01	17.1	18.4	17.7	0.016
psmf1	436820	proteasome (prosome, macropain) inhibitor subunit 1	1.79E-01	2.26E-01	15.3	16.3	15.8	0.016
suclg1	436850	succinate-CoA ligase, GDP-forming, alpha subunit	1.36E-01	2.07E-01	1.7	1.5	1.6	0.016
zgc:92201	436913	zgc:92201	1.13E-01	2.40E-01	16.0	19.4	17.7	0.016
rnf144ab	437000	ring finger protein 144ab	1.62E-01	2.36E-01	11.5	11.5	11.5	0.016
zgc:91963	445108	zgc:91963	4.34E-02	2.43E-01	15.9	19.3	17.6	0.016
sdhc	445129	succinate dehydrogenase complex, subunit C, integral m	2.38E-01	1.11E-01	5.8	1.8	3.8	0.016
pibf1	445181	progesterone immunomodulatory binding factor 1	4.58E-02	2.20E-01	11.7	13.2	12.5	0.016
uevld	445225	UEV and lactate/malate dehyrogenase domains	2.09E-01	1.22E-01	15.0	20.7	17.9	0.016
timm8a	445243	translocase of inner mitochondrial membrane 8 homolog	4.94E-03	2.03E-01	23.2	1.0	0.1	0.016
cep57l1	445312	centrosomal protein 57-like 1	5.39E-02	2.04E-01	9.5	10.8	10.2	0.016
gtf3aa	445389	general transcription factor IIIAa	5.86E-02	2.08E-01	23.7	1.3	0.5	0.016
psmb1	445413	proteasome (prosome, macropain) subunit, beta type, 1	2.21E-01	1.14E-01	21.7	21.2	21.5	0.016
aldh8a1	447801	aldehyde dehydrogenase 8 family, member A1	2.29E-01	1.44E-01	12.3	13.0	12.7	0.016
zgc:103424	447877	zgc:103424	2.09E-01	1.98E-01	13.8	15.0	14.4	0.016

Table S1: Zebrafish circadian oscillating genes

Gene Symbol	Gene ID	Gene Description	Fisher's g test p value (LD)	Fisher's g test p value (DD)	LD Phase	DD Phase	Phase *	FDR **
osbpl3a	447908	oxysterol binding protein-like 3a	3.03E-02	2.46E-01	3.5	4.6	4.1	0.016
mrpl17	447945	mitochondrial ribosomal protein L17	7.53E-02	2.22E-01	0.5	1.7	1.1	0.016
atg4b	448859	ATG4 autophagy related 4 homolog B (S. cerevisiae)	2.21E-01	1.17E-01	12.8	15.4	14.1	0.016
mvk	492477	mevalonate kinase	9.75E-02	2.31E-01	1.9	2.4	2.1	0.016
plekha8	492502	pleckstrin homology domain containing, family A (phosph	7.63E-02	2.25E-01	1.2	1.2	1.2	0.016
ttc36	492516	tetratricopeptide repeat domain 36	1.58E-02	2.13E-01	7.7	7.8	7.8	0.016
fam173b	492519	family with sequence similarity 173, member B	1.29E-01	2.06E-01	12.1	0.8	6.4	0.016
zgc:101777	492762	zgc:101777	1.81E-02	2.44E-01	13.8	16.2	15.0	0.016
rbm41	492764	RNA binding motif protein 41	4.42E-03	2.01E-01	13.7	12.5	13.1	0.016
nsfl1c	492805	NSFL1 (p97) cofactor (p47)	6.86E-02	2.30E-01	18.2	20.2	19.2	0.016
ift80	494082	intraflagellar transport 80 homolog (Chlamydomonas)	4.28E-02	2.31E-01	13.3	15.7	14.5	0.016
xirp1	497637	xin actin-binding repeat containing 1	2.32E-01	5.21E-02	14.2	16.2	15.2	0.016
zgc:113418	503527	zgc:113418	1.57E-01	2.24E-01	11.8	9.7	10.8	0.016
prnprs3	503702	prion protein, related sequence 3	1.31E-01	2.35E-01	15.1	14.8	15.0	0.016
rfc2	503748	replication factor C (activator 1) 2	2.34E-01	1.71E-01	4.7	5.8	5.3	0.016
zgc:110340	541345	zgc:110340	2.37E-01	1.61E-01	16.7	18.2	17.5	0.016
dbt	541388	dihydrolipoamide branched chain transacylase E2	1.36E-01	2.02E-01	4.0	4.5	4.3	0.016
zgc:112175	550414	zgc:112175	7.82E-04	2.21E-01	1.0	0.5	0.8	0.016
zgc:112433	550469	zgc:112433	2.19E-01	2.48E-01	2.3	2.7	2.5	0.016
zgc:112432	550477	zgc:112432	4.73E-03	2.00E-01	5.9	5.5	5.7	0.016
psmb4	550499	proteasome (prosome, macropain) subunit, beta type, 4	2.03E-01	8.43E-02	18.1	20.0	19.1	0.016
ppa1	550531	pyrophosphatase (inorganic) 1	1.61E-01	2.31E-01	2.3	3.8	3.1	0.016
zgc:110179	550538	zgc:110179	7.01E-05	2.00E-01	13.5	15.5	14.5	0.016
necab1	550546	N-terminal EF-hand calcium binding protein 1	7.01E-04	2.11E-01	3.0	3.8	3.4	0.016
ppp1r14aa	550561	protein phosphatase 1, regulatory (inhibitor) subunit 14A	2.92E-03	2.39E-01	4.8	4.0	4.4	0.016
zgc:110605	550597	zgc:110605	1.28E-01	2.12E-01	13.4	13.8	13.6	0.016
slc6a19a	553256	solute carrier family 6 (neurotransmitter transporter), mer	7.06E-02	2.49E-01	22.6	0.6	23.6	0.016
gnptab	553365	N-acetylglucosamine-1-phosphate transferase, alpha and	2.13E-03	2.36E-01	14.5	15.8	15.2	0.016
si:dkey-76k16.7	553448	si:dkey-76k16.7	2.37E-01	1.73E-01	1.2	2.6	1.9	0.016
zgc:110046	553583	zgc:110046	6.33E-02	2.16E-01	0.3	2.0	1.2	0.016
nle1	553606	notchless homolog 1 (Drosophila)	2.43E-01	4.46E-02	22.2	22.5	22.4	0.016

Table S1: Zebrafish circadian oscillating genes

Gene Symbol	Gene ID	Gene Description	Fisher's g test p value (LD)	Fisher's g test p value (DD)	LD Phase	DD Phase	Phase *	FDR **
cyp1c1	553637	cytochrome P450, family 1, subfamily C, polypeptide 1	1.70E-01	2.07E-01	1.5	1.3	1.4	0.016
ndufs5	553660	NADH dehydrogenase (ubiquinone) Fe-S protein 5	1.48E-01	2.42E-01	1.5	2.7	2.1	0.016
clpp	553713	ClpP caseinolytic peptidase, ATP-dependent, proteolytic	2.03E-01	2.09E-01	18.3	19.9	19.1	0.016
lrrc4c	553785	leucine rich repeat containing 4C	2.27E-01	9.41E-02	16.3	17.0	16.7	0.016
pcdh1gb9	553940	protocadherin 1 gamma b 9	5.20E-02	2.22E-01	13.3	14.8	14.1	0.016
inaa	555251	internexin neuronal intermediate filament protein, alpha a	1.97E-01	2.41E-01	3.5	3.5	3.5	0.016
zgc:171630	555328	zgc:171630	2.09E-01	1.77E-01	4.8	4.3	4.6	0.016
cyp2aa12	555510	cytochrome P450, family 2, subfamily AA, polypeptide 12	8.78E-02	2.24E-01	6.0	6.9	6.4	0.016
si:ch73-212e20.3	555558	si:ch73-212e20.3	2.25E-04	2.43E-01	19.8	22.7	21.3	0.016
cd164	555639	CD164 molecule, sialomucin	2.17E-01	6.67E-02	13.8	15.7	14.8	0.016
mdn1	555704	midasin homolog (yeast)	8.33E-02	2.19E-01	0.6	2.6	1.6	0.016
zgc:109953	555951	zgc:109953	1.79E-01	2.32E-01	12.7	8.7	10.7	0.016
tcap	556258	titin-cap (telethonin)	1.47E-02	2.46E-01	3.0	3.0	3.0	0.016
fxn	556389	frataxin	3.22E-02	2.36E-01	2.0	1.7	1.9	0.016
prom2	556596	prominin 2	2.91E-02	2.07E-01	13.0	15.8	14.4	0.016
stxbp5a	556789	syntaxin binding protein 5a (tomosyn)	3.39E-03	2.00E-01	3.7	4.5	4.1	0.016
cnrip1	556881	cannabinoid receptor interacting protein 1	2.88E-02	2.26E-01	18.2	18.0	18.1	0.016
frem1b	557221	Fras-related extracellular matrix protein 1b	3.36E-02	2.25E-01	17.8	19.1	18.4	0.016
hcn2	557285	hyperpolarization activated cyclic nucleotide-gated potas	4.34E-05	2.05E-01	23.7	23.8	23.8	0.016
si:dkey-158b13.2	557667	si:dkey-158b13.2	2.49E-01	2.22E-01	17.8	15.7	16.8	0.016
im:7159098	557849	im:7159098	1.17E-01	2.41E-01	23.7	2.3	1.0	0.016
si:ch211-202h22.6	558776	si:ch211-202h22.6	2.48E-01	6.84E-02	23.8	1.3	0.6	0.016
slc6a11	558960	solute carrier family 6 (neurotransmitter transporter, GAE	8.62E-02	2.27E-01	3.3	4.0	3.6	0.016
lct	559107	lactase	2.92E-02	2.14E-01	0.9	2.4	1.6	0.016
whsc2	559677	Wolf-Hirschhorn syndrome candidate 2	2.41E-01	7.68E-02	14.7	15.7	15.2	0.016
adipor2	560140	adiponectin receptor 2	1.05E-01	2.27E-01	14.7	16.8	15.8	0.016
zgc:123333	560778	zgc:123333	1.04E-01	2.49E-01	14.6	15.3	14.9	0.016
slc1a2a	560802	solute carrier family 1 (glial high affinity glutamate transp	4.56E-05	2.36E-01	0.8	1.2	1.0	0.016
LOC561083	561083	gamma-aminobutyric acid receptor subunit rho-3-like	1.22E-01	2.09E-01	14.3	15.7	15.0	0.016
pcdh17	561336	protocadherin 17	1.88E-01	2.16E-01	18.5	17.8	18.2	0.016
mepce	561384	methylphosphate capping enzyme	2.23E-01	2.16E-02	11.5	14.3	12.9	0.016

Table S1: Zebrafish circadian oscillating genes

Gene Symbol	Gene ID	Gene Description	Fisher's g test p value (LD)	Fisher's g test p value (DD)	LD Phase	DD Phase	Phase *	FDR **
ntn4	561440	netrin 4	6.83E-02	2.15E-01	15.7	18.0	16.9	0.016
LOC562292	562292	acyl-CoA synthetase family member 3, mitochondrial-like	2.43E-01	8.27E-02	2.0	2.7	2.4	0.016
ppp2r5a	562460	protein phosphatase 2, regulatory subunit B', alpha isofo	2.11E-01	1.15E-01	4.2	0.3	2.3	0.016
si:dkey-8l13.4	562490	si:dkey-8l13.4	1.70E-01	2.04E-01	13.8	16.3	15.1	0.016
si:ch211-146l10.2	563215	si:ch211-146l10.2	1.89E-01	2.30E-01	15.4	17.9	16.6	0.016
si:ch211-239e6.4	563220	si:ch211-239e6.4	1.43E-01	2.37E-01	0.5	1.5	1.0	0.016
phf20a	563332	PHD finger protein 20, a	2.01E-01	6.41E-02	12.8	13.5	13.2	0.016
podxl	563365	podocalyxin-like	1.42E-01	2.03E-01	2.7	3.3	3.0	0.016
si:dkey-223d9.4	564291	si:dkey-223d9.4	2.12E-03	2.18E-01	3.1	2.1	2.6	0.016
psma3	564370	proteasome (prosome, macropain) subunit, alpha type, 3	1.79E-02	2.23E-01	19.8	20.5	20.2	0.016
si:rp71-56i13.6	564457	si:rp71-56i13.6	1.43E-01	2.01E-01	14.2	16.7	15.5	0.016
zgc:171480	564733	zgc:171480	3.49E-02	2.13E-01	0.3	1.7	1.0	0.016
glsb	564746	glutaminase b	8.48E-02	2.40E-01	12.3	11.6	11.9	0.016
LOC564868	564868	hypothetical LOC564868	2.20E-01	1.53E-01	15.5	17.2	16.4	0.016
LOC564886	564886	poly [ADP-ribose] polymerase 10-like	3.99E-02	2.02E-01	15.2	17.2	16.2	0.016
si:ch211-137i24.10	565274	si:ch211-137i24.10	7.33E-02	2.43E-01	16.5	17.0	16.8	0.016
LOC565335	565335	novel protein similar to vertebrate solute carrier family 25	2.11E-01	1.45E-01	1.3	4.3	2.8	0.016
prdm11	565473	PR domain containing 11	1.05E-02	2.36E-01	17.5	17.0	17.3	0.016
sat1a	565700	spermidine/spermine N1-acetyltransferase 1a	1.32E-01	2.09E-01	9.3	10.6	9.9	0.016
LOC565776	565776	sodium-dependent noradrenaline transporter-like	1.97E-01	2.22E-01	14.3	10.0	12.2	0.016
zgc:112329	565852	zgc:112329	2.03E-01	8.90E-02	14.2	16.2	15.2	0.016
btbd2	565970	BTB (POZ) domain containing 2	1.26E-01	2.27E-01	15.9	17.6	16.7	0.016
terf1	566954	telomeric repeat binding factor (NIMA-interacting) 1	2.40E-02	2.07E-01	5.9	5.6	5.7	0.016
rif1	567001	RAP1 interacting factor homolog (yeast)	2.09E-01	1.17E-01	2.7	4.3	3.5	0.016
taf1a	567105	TATA box binding protein (TBP)-associated factor, RNA	1.42E-01	2.06E-01	23.5	2.7	1.1	0.016
slc7a9	567117	solute carrier family 7 (cationic amino acid transporter, y-	1.73E-01	2.27E-01	20.5	23.7	22.1	0.016
si:dkey-151c10.1	567639	si:dkey-151c10.1	1.43E-01	2.04E-01	4.2	4.8	4.5	0.016
zgc:171813	568203	zgc:171813	2.12E-01	3.11E-03	16.5	20.5	18.5	0.016
cngb1a	568211	cyclic nucleotide gated channel beta 1a	2.01E-01	3.06E-02	14.5	15.0	14.8	0.016
tmem135	568244	transmembrane protein 135	2.14E-01	1.90E-01	0.7	2.5	1.6	0.016
bcr	568320	breakpoint cluster region	2.45E-01	9.18E-02	18.0	18.3	18.2	0.016

Table S1: Zebrafish circadian oscillating genes

Gene Symbol	Gene ID	Gene Description	Fisher's g test p value (LD)	Fisher's g test p value (DD)	LD Phase	DD Phase	Phase *	FDR **
sla2	568515	Src-like-adaptor 2	1.78E-01	2.48E-01	15.7	18.2	17.0	0.016
zgc:171755	568879	zgc:171755	4.25E-02	2.10E-01	0.8	1.8	1.3	0.016
plcd3a	569040	phospholipase C, delta 3a	2.32E-01	6.38E-02	13.8	16.0	14.9	0.016
si:dkey-46k9.12	569187	si:dkey-46k9.12	2.02E-01	9.29E-02	14.2	16.6	15.4	0.016
cln5	569407	ceroid-lipofuscinosis, neuronal 5	1.41E-02	2.17E-01	9.7	7.5	8.6	0.016
tfb2m	569468	transcription factor B2, mitochondrial	1.16E-01	2.49E-01	18.5	20.2	19.4	0.016
LOC569779	569779	Heavy chain, Unconventional Myosin family member (hu	1.12E-01	2.38E-01	14.3	15.5	14.9	0.016
aqp9b	570191	aquaporin 9b	4.22E-02	2.19E-01	14.0	12.0	13.0	0.016
zgc:114180	570333	zgc:114180	2.38E-01	6.70E-03	18.8	16.2	17.5	0.016
si:dkey-183n20.16	570437	si:dkey-183n20.16	2.47E-01	6.63E-04	3.7	5.0	4.4	0.016
zgc:153073	570830	zgc:153073	6.63E-02	2.35E-01	13.5	16.0	14.8	0.016
gnav1	571305	guanine nucleotide binding protein (G protein) alpha v1	2.07E-01	5.35E-02	13.8	16.3	15.1	0.016
zgc:73075	572207	zgc:73075	2.20E-01	2.01E-02	5.6	3.5	4.5	0.016
zgc:152911	572245	zgc:152911	9.68E-03	2.42E-01	2.8	2.8	2.8	0.016
cx52.7	572278	connexin 52.7	3.02E-02	2.17E-01	19.8	21.7	20.8	0.016
cbx4	573110	chromobox homolog 4 (Pc class homolog, Drosophila)	2.42E-01	2.50E-01	17.7	16.2	17.0	0.016
LOC573743	573743	similar to zinc finger and BTB domain containing 41	2.44E-01	7.94E-02	14.5	13.7	14.1	0.016
dusp12	573998	dual specificity phosphatase 12	2.25E-01	1.95E-01	0.3	2.7	1.5	0.016
zgc:109809	664743	zgc:109809	8.77E-02	2.43E-01	15.2	15.2	15.2	0.016
cldn15b	678556	claudin 15b	2.05E-01	5.77E-02	20.0	20.8	20.4	0.016
alg5	751635	asparagine-linked glycosylation 5 homolog (yeast, dolich	1.50E-01	2.01E-01	23.0	0.7	23.9	0.016
fam169aa	751650	family with sequence similarity 169, member Aa	1.13E-01	2.39E-01	5.3	4.5	4.9	0.016
eml1	767643	echinoderm microtubule associated protein like 1	3.75E-03	2.24E-01	22.7	23.5	23.1	0.016
zgc:153372	767695	zgc:153372	4.38E-03	2.04E-01	16.5	18.0	17.3	0.016
znrf2	767761	zinc and ring finger 2	1.10E-01	2.37E-01	2.0	4.5	3.3	0.016
zgc:153041	768161	zgc:153041	2.39E-01	1.99E-01	3.8	5.3	4.6	0.016
rilpl1	780843	Rab interacting lysosomal protein-like 1	2.48E-01	6.52E-02	16.9	18.3	17.6	0.016
loxl2b	791144	lysyl oxidase-like 2b	2.27E-01	6.51E-02	2.5	3.0	2.7	0.016
slco2b1	792084	solute carrier organic anion transporter family, member 2	4.71E-02	2.33E-01	13.8	15.1	14.4	0.016
si:dkey-126g1.9	792999	si:dkey-126g1.9	2.00E-01	1.38E-01	10.8	10.0	10.4	0.016
LOC793259	793259	hypothetical protein LOC793259	2.15E-01	1.28E-01	9.7	9.7	9.7	0.016

Table S1: Zebrafish circadian oscillating genes

Gene Symbol	Gene ID	Gene Description	Fisher's g test p value (LD)	Fisher's g test p value (DD)	LD Phase	DD Phase	Phase *	FDR **
lbx1b	793810	ladybird homeobox 1b	7.73E-02	2.02E-01	13.2	15.4	14.3	0.016
LOC794117	794117	similar to Ring finger protein 14	1.38E-02	2.40E-01	5.0	5.3	5.1	0.016
slc22a7a	794188	solute carrier family 22 (organic anion transporter), meml	2.23E-01	2.48E-01	4.7	4.7	4.7	0.016
si:ch211-202f5.3	795321	si:ch211-202f5.3	2.18E-01	1.34E-01	0.3	2.2	1.3	0.016
slc16a9a	795588	solute carrier family 16 (monocarboxylic acid transporters	2.04E-01	5.42E-02	5.5	5.3	5.4	0.016
smc6	797304	structural maintenance of chromosomes 6	3.54E-02	2.46E-01	12.5	16.2	14.4	0.016
si:dkey-224e22.3	797513	si:dkey-224e22.3	2.38E-01	6.71E-02	1.5	2.7	2.1	0.016
tpp1	798347	tripeptidyl peptidase I	6.17E-02	2.36E-01	14.8	17.7	16.3	0.016
si:ch211-217k17.9	798921	si:ch211-217k17.9	2.23E-02	2.16E-01	1.7	4.2	3.0	0.016
anxa2b	799806	annexin A2b	2.26E-01	1.67E-03	3.0	4.1	3.5	0.016
LOC100000576	100000576	ATP-binding cassette, sub-family F, member 2-like	1.64E-02	2.31E-01	3.2	2.3	2.8	0.016
zgc:162060	100001158	zgc:162060	2.16E-01	4.22E-02	5.5	6.5	6.0	0.016
ifitm1	100003209	interferon induced transmembrane protein 1 (9-27)	2.16E-01	7.77E-02	5.2	4.3	4.8	0.016
LOC100004809	100004809	protein FAM78B-like	2.41E-01	8.26E-02	20.3	19.0	19.7	0.016
pcdh10	100006777	protocadherin 10	4.90E-02	2.10E-01	16.8	16.3	16.6	0.016
pcp4l1	100008021	Purkinje cell protein 4 like 1	2.54E-03	2.21E-01	15.3	13.3	14.3	0.016
klhl32	100009653	kelch-like 32 (Drosophila)	2.04E-01	1.94E-01	13.2	14.0	13.6	0.016
patl1	100009659	protein associated with topoisomerase II homolog 1 (yea	1.60E-02	2.26E-01	14.7	16.7	15.7	0.016
atg9a	100009663	ATG9 autophagy related 9 homolog A (S. cerevisiae)	1.85E-01	2.41E-01	11.2	16.2	13.7	0.016
rhcga	100038797	Rhesus blood group, C glycoprotein a	1.08E-01	2.08E-01	2.3	3.4	2.8	0.016
zgc:162641	100093708	zgc:162641	2.57E-02	2.03E-01	15.0	16.2	15.6	0.016
tm2d1	100093710	TM2 domain containing 1	9.12E-03	2.38E-01	16.7	18.0	17.4	0.016
zgc:165582	100124609	zgc:165582	2.49E-01	1.96E-01	18.2	19.3	18.8	0.016
ebi3	100134974	Epstein-Barr virus induced gene 3	2.04E-01	6.03E-02	1.8	3.5	2.6	0.016
fbxw8	100136859	F-box and WD repeat domain containing 8	5.06E-02	2.28E-01	15.5	17.2	16.4	0.016
zgc:173713	100137115	zgc:173713	2.24E-02	2.16E-01	9.7	13.3	11.5	0.016
lysmd4	100149102	LysM, putative peptidoglycan-binding, domain containing	2.39E-02	2.37E-01	6.5	5.5	6.0	0.016
si:dkey-69o16.5	100149419	si:dkey-69o16.5	2.11E-01	1.68E-01	6.7	9.3	8.0	0.016
si:dkey-211e20.10	100329583	si:dkey-211e20.10	3.72E-02	2.21E-01	20.5	22.7	21.6	0.016
LOC100329779	100329779	rho-associated protein kinase 1-like	1.56E-01	2.09E-01	18.8	18.0	18.4	0.016
arfgef1	100329953	ADP-ribosylation factor guanine nucleotide-exchange fac	9.96E-02	2.47E-01	17.9	17.3	17.6	0.016

Table S1: Zebrafish circadian oscillating genes

Gene Symbol	Gene ID	Gene Description	Fisher’s g test p value (LD)	Fisher’s g test p value (DD)	LD Phase	DD Phase	Phase *	FDR **
LOC100330595	100330595	hypothetical protein LOC100330595	2.29E-01	2.43E-01	3.3	3.5	3.4	0.016
LOC100332307	100332307	crossover junction endonuclease EME1-like	9.53E-02	2.36E-01	13.7	16.2	15.0	0.016
mpp5b	100422734	membrane protein, palmitoylated 5b (MAGUK p55 subfa	2.05E-01	2.43E-01	19.2	16.0	17.6	0.016
mitfa	30080	microphthalmia-associated transcription factor a	1.43E-03	2.78E-01	20.3	22.9	21.6	0.022
cdh2	30291	cadherin 2, neuronal	2.91E-01	9.32E-02	16.8	16.0	16.4	0.022
foxo3b	30296	forkhead box O3b	1.42E-01	2.65E-01	11.2	13.7	12.5	0.022
tcf3	30310	transcription factor 3	4.52E-02	2.95E-01	13.0	12.8	12.9	0.022
hsp90ab1	30573	heat shock protein 90, alpha (cytosolic), class B member	8.32E-02	2.61E-01	15.8	17.5	16.7	0.022
ppm1aa	30704	protein phosphatase, Mg2+/Mn2+ dependent, 1Aa	2.38E-01	2.66E-01	18.8	17.7	18.3	0.022
robo1	30769	roundabout homolog 1	2.53E-01	9.15E-02	20.0	22.0	21.0	0.022
copz2	57969	zeta2-cop	1.71E-02	2.50E-01	6.8	6.2	6.5	0.022
pim1	58054	pim-1 oncogene	1.67E-01	2.83E-01	10.5	14.0	12.3	0.022
slc34a2a	58148	solute carrier family 34 (sodium phosphate), member 2a	6.08E-02	2.60E-01	21.0	22.5	21.8	0.022
fech	58215	ferrochelatase	4.99E-02	2.79E-01	6.8	1.5	4.2	0.022
gstp1	79381	glutathione S-transferase pi	2.76E-01	2.78E-01	7.3	8.2	7.8	0.022
crx	81881	cone-rod homeobox	2.60E-01	4.35E-02	17.3	15.9	16.6	0.022
jag2	140422	jagged 2	7.09E-02	2.95E-01	17.5	17.3	17.4	0.022
per2	140633	period homolog 2 (Drosophila)	2.24E-02	2.83E-01	6.3	8.7	7.5	0.022
ptk2.1	142986	protein tyrosine kinase 2a	2.98E-01	7.55E-02	12.0	16.0	14.0	0.022
mcm2	192338	MCM2 minichromosome maintenance deficient 2, mitotir	1.66E-01	2.65E-01	2.3	3.7	3.0	0.022
fmr1	259191	fragile X mental retardation 1	1.23E-01	2.60E-01	19.2	18.6	18.9	0.022
drd3	282554	dopamine receptor D3	4.50E-02	2.74E-01	12.2	14.0	13.1	0.022
sly1	317732	suppressor of ypt1	2.94E-01	9.53E-03	21.5	19.5	20.5	0.022
hmgb1a	321622	high-mobility group box 1a	1.67E-01	2.83E-01	17.3	18.2	17.8	0.022
wwp1	321695	WW domain containing E3 ubiquitin protein ligase 1	2.78E-01	2.19E-01	17.5	19.0	18.3	0.022
apoa4	322543	apolipoprotein A-IV	2.68E-01	5.65E-02	7.2	8.0	7.6	0.022
mid1ip1	322977	MID1 interacting protein 1	2.01E-03	2.75E-01	3.7	3.7	3.7	0.022
ell2	322997	elongation factor, RNA polymerase II, 2	1.41E-02	2.60E-01	2.8	3.3	3.0	0.022
wu:fb93d04	323251	wu:fb93d04	3.11E-02	2.59E-01	10.0	11.5	10.8	0.022
tia1l	323447	cytotoxic granule-associated RNA binding protein 1, like	2.32E-01	2.65E-01	23.4	1.9	0.6	0.022
metap2	323452	methionyl aminopeptidase 2	1.73E-01	2.63E-01	3.5	22.7	1.1	0.022

Table S1: Zebrafish circadian oscillating genes

Gene Symbol	Gene ID	Gene Description	Fisher's g test p value (LD)	Fisher's g test p value (DD)	LD Phase	DD Phase	Phase *	FDR **
dbr1	323746	debranching enzyme homolog 1 (S. cerevisiae)	1.40E-01	2.87E-01	2.5	4.2	3.4	0.022
c20orf14	323855	c20orf14 homolog (H. sapiens)	9.20E-02	2.78E-01	17.5	18.2	17.9	0.022
foxk2	324141	forkhead box K2	3.42E-03	2.84E-01	18.8	18.7	18.8	0.022
tax1bp1b	324152	Tax1 (human T-cell leukemia virus type I) binding proteir	6.94E-02	2.89E-01	14.0	16.0	15.0	0.022
wu:fc31e09	324521	wu:fc31e09	2.59E-01	2.52E-01	22.2	23.3	22.8	0.022
taldo1	324556	transaldolase 1	2.69E-01	1.73E-01	3.5	3.0	3.3	0.022
ogfod1	324573	2-oxoglutarate and iron-dependent oxygenase domain cc	1.68E-01	2.67E-01	23.5	3.3	1.4	0.022
tsr1	325037	TSR1, 20S rRNA accumulation, homolog (yeast)	1.23E-01	2.52E-01	23.3	1.5	0.4	0.022
wu:fc55e03	325104	wu:fc55e03	2.96E-01	1.32E-01	13.0	16.2	14.6	0.022
rcan3	325356	regulator of calcineurin family member 3	2.91E-01	5.73E-02	12.2	14.8	13.5	0.022
polr1b	325603	polymerase (RNA) I polypeptide B	8.86E-02	2.66E-01	21.3	23.6	22.4	0.022
tsku	325958	tsukushi small leucine rich proteoglycan homolog (Xenop	5.62E-03	2.94E-01	5.0	4.5	4.8	0.022
zgc:56622	326033	zgc:56622	3.50E-02	2.80E-01	20.2	21.6	20.9	0.022
mg:cb01g09	326964	mg:cb01g09	4.05E-04	2.67E-01	3.2	3.2	3.2	0.022
micall2a	327198	mical-like 2a	2.53E-02	2.97E-01	14.0	15.5	14.8	0.022
cndp2	327288	CNDP dipeptidase 2 (metallopeptidase M20 family)	1.77E-01	2.80E-01	3.3	4.7	4.0	0.022
zgc:123170	334107	zgc:123170	2.67E-01	5.62E-03	21.0	0.8	22.9	0.022
slc43a1a	334395	solute carrier family 43, member 1a	2.65E-01	1.53E-01	22.7	13.0	17.8	0.022
rdh14b	334665	retinol dehydrogenase 14b (all-trans/9-cis/11-cis)	2.66E-01	1.64E-01	14.2	16.8	15.5	0.022
wu:fa04a09	334800	wu:fa04a09	1.42E-01	2.58E-01	15.2	17.3	16.3	0.022
rogdi	335642	rogdi homolog (Drosophila)	3.78E-02	2.74E-01	14.7	19.0	16.9	0.022
zgc:56683	336470	zgc:56683	1.42E-01	2.88E-01	23.3	1.3	0.3	0.022
drg1	336823	developmentally regulated GTP binding protein 1	3.43E-02	2.52E-01	23.3	2.2	0.8	0.022
ranbp1	337046	RAN binding protein 1	2.52E-01	1.71E-01	0.9	4.9	2.9	0.022
gpx4a	352928	glutathione peroxidase 4a	5.60E-02	2.67E-01	11.6	14.8	13.2	0.022
gtpbp1	378721	GTP binding protein 1	5.71E-02	2.68E-01	14.0	16.3	15.2	0.022
arf5	378724	ADP-ribosylation factor 5	6.77E-02	2.85E-01	0.3	0.3	0.3	0.022
zgc:110586	378854	zgc:110586	1.93E-01	2.86E-01	14.3	15.0	14.6	0.022
ncf1	378966	neutrophil cytosolic factor 1	3.18E-02	2.80E-01	15.5	15.8	15.7	0.022
wu:fb57e05	386954	wu:fb57e05	2.88E-01	1.36E-01	18.4	21.3	19.8	0.022
scarb1	387260	scavenger receptor class B, member 1	8.34E-02	2.78E-01	14.7	16.3	15.5	0.022

Table S1: Zebrafish circadian oscillating genes

Gene Symbol	Gene ID	Gene Description	Fisher's g test p value (LD)	Fisher's g test p value (DD)	LD Phase	DD Phase	Phase *	FDR **
cpne3	393136	copine III	7.50E-02	2.95E-01	6.5	5.3	5.9	0.022
usf1	393266	upstream transcription factor 1	5.48E-02	2.89E-01	12.5	13.8	13.2	0.022
hsd11b3a	393293	hydroxysteroid (11-beta) dehydrogenase 3a	2.16E-02	2.99E-01	3.4	2.6	3.0	0.022
wipi1	393362	WD repeat domain, phosphoinositide interacting 1	2.64E-01	9.39E-02	13.0	15.7	14.4	0.022
dnajc5gb	393371	DnaJ (Hsp40) homolog, subfamily C, member 5 gamma	2.72E-01	7.05E-02	2.0	21.3	23.7	0.022
tmem206	393429	transmembrane protein 206	2.87E-01	2.47E-01	15.2	19.2	17.2	0.022
pfdn6	393485	prefoldin subunit 6	2.54E-01	1.40E-01	1.2	2.8	2.0	0.022
slc39a10	393644	solute carrier family 39 (zinc transporter), member 10	1.40E-01	2.62E-01	1.0	3.7	2.4	0.022
exosc4	393712	exosome component 4	1.18E-01	2.75E-01	0.8	2.0	1.4	0.022
zgc:73238	393731	zgc:73238	3.35E-02	2.53E-01	1.6	1.9	1.7	0.022
zgc:73336	393767	zgc:73336	1.32E-01	2.57E-01	11.9	14.2	13.1	0.022
myoz2b	393828	myozenin 2b	2.63E-01	2.95E-02	2.8	3.5	3.2	0.022
ptdss1	393931	phosphatidylserine synthase 1	1.26E-01	2.82E-01	18.6	20.8	19.7	0.022
zgc:55733	393968	zgc:55733	3.56E-03	2.78E-01	15.4	18.3	16.9	0.022
rnf11	393996	ring finger protein 11	2.22E-01	2.60E-01	12.8	15.5	14.2	0.022
srp9	394071	signal recognition particle 9	3.94E-02	2.86E-01	2.7	3.3	3.0	0.022
asns	394138	asparagine synthetase	3.04E-02	2.84E-01	0.5	1.7	1.1	0.022
dnaja3b	394242	DnaJ (Hsp40) homolog, subfamily A, member 3B	1.23E-02	2.60E-01	2.7	4.2	3.5	0.022
pafah1b1a	394246	platelet-activating factor acetylhydrolase, isoform Ib, alph	2.51E-01	1.69E-02	16.0	16.7	16.4	0.022
myo3a	402814	myosin IIIA	1.49E-02	2.94E-01	3.8	2.2	3.0	0.022
zgc:77234	402970	zgc:77234	2.31E-01	2.74E-01	13.2	16.2	14.7	0.022
myd88	403145	myeloid differentiation primary response gene (88)	2.35E-01	2.51E-01	14.2	18.4	16.3	0.022
zgc:55936	404050	zgc:55936	1.07E-01	2.54E-01	12.7	16.2	14.5	0.022
itgb1b.2	405864	integrin, beta 1b.2	2.71E-01	7.39E-02	7.8	11.0	9.4	0.022
xpnpep1	406253	X-prolyl aminopeptidase (aminopeptidase P) 1, soluble	2.78E-02	2.58E-01	6.1	7.5	6.8	0.022
hprt1	406259	hypoxanthine phosphoribosyltransferase 1	1.02E-01	2.70E-01	0.3	2.8	1.5	0.022
uhrf1	406350	ubiquitin-like, containing PHD and RING finger domains,	2.78E-01	2.28E-01	3.7	4.8	4.3	0.022
tmbim4	406412	transmembrane BAX inhibitor motif containing 4	2.39E-01	2.91E-01	20.3	19.8	20.1	0.022
atf4b1	406514	activating transcription factor 4b1 (tax-responsive enhan	2.78E-01	2.03E-02	14.5	17.0	15.8	0.022
ttyh2l	406540	tweety homolog 2, like	5.28E-03	2.66E-01	14.2	15.5	14.9	0.022
ctsba	406645	cathepsin B, a	2.93E-02	2.87E-01	15.5	18.0	16.8	0.022

Table S1: Zebrafish circadian oscillating genes

Gene Symbol	Gene ID	Gene Description	Fisher's g test p value (LD)	Fisher's g test p value (DD)	LD Phase	DD Phase	Phase *	FDR **
ddc	406651	dopa decarboxylase	5.36E-03	2.97E-01	1.1	2.9	2.0	0.022
slc25a22	406717	solute carrier family 25 (mitochondrial carrier: glutamate)	1.13E-03	2.86E-01	12.7	16.3	14.5	0.022
maf1	406750	MAF1 homolog (S. cerevisiae)	8.46E-02	2.88E-01	12.8	12.0	12.4	0.022
adipor1b	406821	adiponectin receptor 1b	4.41E-02	2.89E-01	14.2	16.7	15.5	0.022
msh2	406845	mutS homolog 2 (E. coli)	2.68E-01	2.03E-01	4.4	5.3	4.8	0.022
si:ch211-102c2.6	407646	si:ch211-102c2.6	2.94E-01	2.80E-02	17.3	17.2	17.3	0.022
znf598	407728	zinc finger protein 598	1.28E-01	2.59E-01	8.1	1.9	5.0	0.022
tcf12	407985	transcription factor 12	2.46E-01	2.87E-01	13.5	14.0	13.8	0.022
zgc:92710	436669	zgc:92710	2.93E-01	2.94E-01	16.7	15.5	16.1	0.022
pdha1b	436672	pyruvate dehydrogenase (lipoamide) alpha 1b	6.25E-03	2.86E-01	0.3	23.5	23.9	0.022
mrpl24	436674	mitochondrial ribosomal protein L24	1.44E-01	2.99E-01	0.8	2.8	1.8	0.022
tspan18b	436712	tetraspanin 18b	8.67E-02	2.54E-01	14.3	13.3	13.8	0.022
zgc:92827	436781	zgc:92827	3.66E-03	2.60E-01	17.3	19.7	18.5	0.022
rnd3b	436864	Rho family GTPase 3b	1.20E-03	2.91E-01	16.5	19.5	18.0	0.022
kctd9	437011	potassium channel tetramerisation domain containing 9	5.23E-02	2.53E-01	15.0	15.2	15.1	0.022
bag3	445139	BCL2-associated athanogene 3	2.86E-01	1.75E-01	1.0	23.0	0.0	0.022
zgc:103678	447924	zgc:103678	2.66E-01	1.17E-01	23.8	22.2	23.0	0.022
pip5k1b	449831	phosphatidylinositol-4-phosphate 5-kinase, type I, beta	1.03E-03	2.92E-01	4.7	3.0	3.9	0.022
zgc:101814	450027	zgc:101814	7.02E-02	2.90E-01	23.5	1.2	0.4	0.022
zgc:101768	450034	zgc:101768	4.46E-02	2.78E-01	23.0	0.8	23.9	0.022
angptl7	450053	angiopoietin-like 7	9.38E-02	2.67E-01	2.8	2.3	2.6	0.022
hsd17b10	450078	hydroxysteroid (17-beta) dehydrogenase 10	1.77E-03	2.65E-01	4.0	4.8	4.4	0.022
zgc:92172	492353	zgc:92172	2.55E-01	1.16E-01	10.0	7.8	8.9	0.022
cab39l	492361	calcium binding protein 39-like	2.99E-01	2.32E-01	16.7	10.7	13.7	0.022
dennd2d	492474	DENN/MADD domain containing 2D	3.00E-01	2.67E-01	16.5	17.8	17.2	0.022
slc25a44a	492478	solute carrier family 25, member 44 a	1.79E-02	2.81E-01	23.7	23.8	23.8	0.022
wdfy2	492636	WD repeat and FYVE domain containing 2	2.64E-02	2.85E-01	13.9	17.0	15.4	0.022
im:7151270	492704	im:7151270	4.73E-03	2.95E-01	16.0	16.5	16.3	0.022
zgc:101553	492778	zgc:101553	2.51E-01	1.19E-01	15.0	16.5	15.8	0.022
zgc:101653	492800	zgc:101653	1.50E-01	2.90E-01	14.3	16.2	15.3	0.022
farsb	493608	phenylalanyl-tRNA synthetase, beta subunit	2.69E-01	2.25E-01	18.4	18.9	18.6	0.022

Table S1: Zebrafish circadian oscillating genes

Gene Symbol	Gene ID	Gene Description	Fisher's g test p value (LD)	Fisher's g test p value (DD)	LD Phase	DD Phase	Phase *	FDR **
drd4b	503565	dopamine receptor D4b	2.03E-03	2.82E-01	7.5	9.7	8.6	0.022
mybpc2b	541384	myosin binding protein C, fast type b	5.09E-02	2.92E-01	14.7	17.5	16.1	0.022
xpc	541386	xeroderma pigmentosum, complementation group C	8.62E-02	2.96E-01	5.0	5.2	5.1	0.022
zgc:113071	541520	zgc:113071	4.36E-03	2.87E-01	15.3	17.5	16.4	0.022
zgc:110113	550236	zgc:110113	2.81E-01	2.89E-01	3.2	4.5	3.9	0.022
zgc:110239	550326	zgc:110239	1.10E-02	2.87E-01	15.7	15.3	15.5	0.022
phyh	550521	phytanoyl-CoA 2-hydroxylase	1.43E-01	2.68E-01	8.3	7.7	8.0	0.022
stk11	550537	serine/threonine kinase 11	5.34E-02	2.59E-01	18.7	17.8	18.3	0.022
zgc:110176	550539	zgc:110176	1.91E-03	2.92E-01	21.7	0.5	23.1	0.022
ift140	553213	intraflagellar transport 140 homolog (Chlamydomonas)	2.51E-01	1.23E-01	10.7	17.3	14.0	0.022
eif4ba	553214	eukaryotic translation initiation factor 4Ba	1.59E-01	2.86E-01	1.2	19.7	22.5	0.022
si:dkey-58f10.1	553284	si:dkey-58f10.1	9.34E-02	2.63E-01	0.8	1.7	1.3	0.022
si:ch211-173p18.3	553317	si:ch211-173p18.3	2.56E-01	6.09E-02	13.5	14.2	13.9	0.022
col4a3	553354	collagen, type IV, alpha 3	7.36E-02	2.76E-01	14.2	17.5	15.9	0.022
zgc:152774	553526	zgc:152774	2.70E-01	5.26E-02	14.8	15.7	15.3	0.022
alg3	553725	asparagine-linked glycosylation 3 homolog (S. cerevisiae	1.86E-02	2.59E-01	21.5	0.3	22.9	0.022
map3k7	553788	mitogen activated protein kinase kinase kinase 7	1.48E-01	2.72E-01	19.7	18.0	18.9	0.022
myh11a	554168	myosin, heavy polypeptide 11, smooth muscle a	2.02E-01	2.91E-01	4.0	5.0	4.5	0.022
orc1	554509	origin recognition complex, subunit 1	1.87E-01	2.60E-01	3.5	5.0	4.3	0.022
ubn2	555525	ubinuclein 2	2.24E-01	2.54E-01	14.7	16.7	15.7	0.022
bmpr2b	555655	bone morphogenetic protein receptor, type II b (serine/th	2.51E-01	8.91E-02	15.5	12.0	13.8	0.022
wu:fi41d10	556702	wu:fi41d10	2.93E-02	2.67E-01	21.5	23.2	22.4	0.022
ankrd13a	556837	ankyrin repeat domain 13A	1.24E-01	2.56E-01	13.6	15.3	14.4	0.022
als2a	557014	amyotrophic lateral sclerosis 2a (juvenile)	2.52E-01	1.64E-01	10.5	14.5	12.5	0.022
slc26a2	557046	solute carrier family 26 (sulfate transporter), member 2	5.27E-04	2.93E-01	1.7	3.2	2.5	0.022
LOC557176	557176	similar to phosphoinositide-3-kinase, regulatory subunit,	2.79E-01	2.38E-02	4.2	6.2	5.2	0.022
LOC557848	557848	hypothetical protein LOC557848	4.18E-02	2.72E-01	19.0	16.3	17.7	0.022
xpot	558020	exportin, tRNA (nuclear export receptor for tRNAs)	1.03E-01	2.81E-01	22.3	23.3	22.8	0.022
si:ch211-150o23.2	558029	si:ch211-150o23.2	1.18E-01	2.58E-01	12.3	18.0	15.2	0.022
lad1	558327	ladinin	1.26E-03	2.98E-01	8.3	9.2	8.8	0.022
peli1b	558420	pellino homolog 1b (Drosophila)	5.40E-02	2.65E-01	13.4	15.0	14.2	0.022

Table S1: Zebrafish circadian oscillating genes

Gene Symbol	Gene ID	Gene Description	Fisher's g test p value (LD)	Fisher's g test p value (DD)	LD Phase	DD Phase	Phase *	FDR **
lipin2	558422	lipin 2	2.09E-01	2.73E-01	12.8	15.5	14.2	0.022
kcna4	559036	potassium voltage-gated channel, shaker-related subfam	1.30E-01	2.97E-01	1.7	0.5	1.1	0.022
grpel1	559037	GrpE-like 1, mitochondrial	5.74E-03	2.64E-01	1.3	2.2	1.8	0.022
zgc:153279	559467	zgc:153279	2.66E-01	2.95E-02	14.0	16.2	15.1	0.022
LOC559804	559804	similar to RING finger protein 150	2.59E-01	3.70E-02	15.5	19.3	17.4	0.022
neu1	559850	neuraminidase 1	6.08E-03	2.70E-01	14.5	18.8	16.7	0.022
LOC559938	559938	inositol hexakisphosphate and diphosphoinositol-pentaki	2.89E-01	2.29E-02	0.8	4.8	2.8	0.022
arl15b	560030	ADP-ribosylation factor-like 15b	2.97E-01	2.66E-01	11.8	16.7	14.3	0.022
serpinf2	560308	serine (or cysteine) peptidase inhibitor, clade F, member	2.57E-01	2.76E-01	2.8	3.5	3.2	0.022
tfpi2	560339	tissue factor pathway inhibitor 2	1.90E-01	2.72E-01	13.2	15.8	14.5	0.022
lin54	560688	lin-54 homolog	2.10E-02	2.93E-01	14.7	15.5	15.1	0.022
si:ch211-127d4.3	560957	si:ch211-127d4.3	7.61E-03	2.71E-01	16.0	17.8	16.9	0.022
ptrfb	561082	polymerase I and transcript release factor b	2.58E-01	4.74E-02	2.2	5.2	3.7	0.022
si:dkey-222f8.3	561722	si:dkey-222f8.3	1.59E-02	2.62E-01	8.0	11.2	9.6	0.022
epha7	562195	eph receptor A7	2.95E-01	2.07E-01	17.9	17.2	17.6	0.022
si:ch211-241e15.2	562991	si:ch211-241e15.2	6.12E-02	2.54E-01	1.0	0.5	0.8	0.022
kif5ba	563459	kinesin family member 5B, a	2.09E-01	2.93E-01	19.3	18.5	18.9	0.022
pbxip1a	563463	pre-B-cell leukemia homeobox interacting protein 1a	2.40E-01	2.82E-01	1.3	1.0	1.2	0.022
slc17a8	563467	solute carrier family 17 (sodium-dependent inorganic pho	2.12E-01	2.86E-01	15.9	2.7	21.3	0.022
prosc	563548	proline synthetase co-transcribed homolog (bacterial)	2.40E-01	2.68E-01	3.5	7.0	5.3	0.022
si:ch211-145c1.1	563686	si:ch211-145c1.1	2.74E-01	2.44E-01	0.5	1.5	1.0	0.022
LOC564004	564004	ubiquitin carboxyl-terminal hydrolase 45-like	1.37E-01	2.74E-01	22.5	23.2	22.9	0.022
ngfrl	564338	nerve growth factor receptor (TNFR superfamily, membe	2.93E-01	1.20E-01	5.3	2.8	4.1	0.022
efcab4b	564347	EF-hand calcium binding domain 4B	1.70E-03	2.68E-01	15.0	14.3	14.7	0.022
LOC564649	564649	similar to 3-oxoacyl-[acyl-carrier-protein] synthase, mitoc	2.52E-02	2.59E-01	12.3	14.5	13.4	0.022
LOC565088	565088	RNA pseudouridylate synthase domain-containing protei	9.38E-02	2.92E-01	22.0	23.7	22.9	0.022
zgc:174642	565794	zgc:174642	2.81E-01	2.82E-01	12.0	9.5	10.8	0.022
arid2	566352	AT rich interactive domain 2 (ARID, RFX-like)	1.12E-01	2.56E-01	21.3	18.7	20.0	0.022
sb:cb1095	566522	sb:cb1095	2.76E-01	2.54E-01	21.8	2.3	0.1	0.022
git2b	567190	G protein-coupled receptor kinase interactor 2b	2.46E-01	2.88E-01	14.8	13.2	14.0	0.022
LOC567310	567310	plectin-like	1.38E-01	2.61E-01	4.5	2.0	3.3	0.022

Table S1: Zebrafish circadian oscillating genes

Gene Symbol	Gene ID	Gene Description	Fisher's g test p value (LD)	Fisher's g test p value (DD)	LD Phase	DD Phase	Phase *	FDR **
farp2	567490	FERM, RhoGEF and pleckstrin domain protein 2	2.12E-01	2.96E-01	15.9	15.8	15.9	0.022
crsp6	567560	cofactor required for Sp1 transcriptional activation, subur	6.08E-02	2.86E-01	16.3	16.5	16.4	0.022
dcaf11	567578	ddb1 and cul4 associated factor 11	2.95E-02	2.54E-01	13.3	14.8	14.0	0.022
mycl1b	567762	v-myc myelocytomatosis viral oncogene homolog 1, lung	6.93E-03	2.57E-01	5.5	6.2	5.9	0.022
mical1	568573	microtubule associated monoxygenase, calponin and LIM	2.38E-01	2.80E-01	12.0	15.0	13.5	0.022
LOC568625	568625	complement C1q-like protein 4-like	1.07E-01	2.93E-01	18.2	20.2	19.2	0.022
slc4a4a	568631	solute carrier family 4, member 4a	4.47E-05	2.76E-01	4.0	5.0	4.5	0.022
pacs1	569034	phosphofurin acidic cluster sorting protein 1	1.56E-01	2.61E-01	14.8	16.3	15.6	0.022
zgc:161969	569044	zgc:161969	2.71E-01	4.35E-02	10.0	8.7	9.4	0.022
zgc:153790	569483	zgc:153790	2.90E-01	2.83E-01	20.7	2.7	23.7	0.022
si:ch211-233e16.1	569692	si:ch211-233e16.1	6.20E-02	2.70E-01	1.7	5.8	3.8	0.022
wdr76	570063	WD repeat domain 76	3.25E-02	2.93E-01	5.7	6.8	6.3	0.022
si:ch211-207d6.2	570069	si:ch211-207d6.2	1.17E-01	2.82E-01	4.8	4.2	4.5	0.022
stab1l	570626	stabilin 1-like	1.92E-03	2.82E-01	15.5	18.8	17.2	0.022
LOC571066	571066	sialin-like	3.94E-02	2.58E-01	9.7	20.0	14.9	0.022
si:ch211-251b21.1	571720	si:ch211-251b21.1	1.15E-02	2.63E-01	11.5	11.2	11.4	0.022
LOC571837	571837	similar to 130 kDa myosin-binding subunit of smooth mus	1.92E-02	2.86E-01	15.8	16.5	16.2	0.022
si:rp71-36a1.1	571927	si:rp71-36a1.1	2.96E-01	2.62E-01	10.0	8.5	9.3	0.022
tmem115	619264	transmembrane protein 115	2.80E-01	1.55E-01	14.5	16.5	15.5	0.022
baiap2a	678518	BAI1-associated protein 2a	2.58E-01	1.91E-01	12.3	10.2	11.3	0.022
asb16	678621	ankyrin repeat and SOCS box-containing 16	2.90E-02	2.67E-01	11.0	17.0	14.0	0.022
cyp2k17	735313	cytochrome P450, family 2, subfamily K, polypeptide17	6.36E-02	2.70E-01	12.2	11.2	11.7	0.022
zgc:153260	751643	zgc:153260	2.78E-01	1.15E-01	1.3	3.3	2.3	0.022
fam102ab	767674	family with sequence similarity 102, member Ab	2.74E-01	1.73E-01	2.0	3.6	2.8	0.022
btr16	767728	bloodthirsty-related gene family, member 16	1.49E-01	2.89E-01	13.8	16.9	15.3	0.022
ugt5a2	767756	UDP glucuronosyltransferase 5 family, polypeptide A2	7.03E-03	2.93E-01	4.3	2.8	3.6	0.022
rab3gap1	777744	RAB3 GTPase activating protein subunit 1	1.62E-01	2.99E-01	9.0	10.1	9.6	0.022
zgc:158626	791189	zgc:158626	2.70E-01	1.50E-01	11.7	15.0	13.4	0.022
ogg1	791194	8-oxoguanine DNA glycosylase	2.12E-01	2.61E-01	15.0	17.2	16.1	0.022
zgc:158254	791209	zgc:158254	7.44E-02	2.53E-01	20.5	18.8	19.7	0.022
pnp5a	791647	purine nucleoside phosphorylase 5a	2.51E-01	9.22E-02	7.6	5.4	6.5	0.022

Table S1: Zebrafish circadian oscillating genes

Gene Symbol	Gene ID	Gene Description	Fisher's g test p value (LD)	Fisher's g test p value (DD)	LD Phase	DD Phase	Phase *	FDR **
rnaset2	791890	ribonuclease T2	2.59E-01	1.42E-01	13.3	17.2	15.3	0.022
lztr1	792832	leucine-zipper-like transcription regulator 1	8.35E-02	2.61E-01	14.4	14.5	14.4	0.022
fars2	793097	phenylalanyl-tRNA synthetase 2, mitochondrial	2.92E-01	1.77E-01	3.3	6.7	5.0	0.022
snx25	793657	sorting nexin 25	3.42E-02	2.83E-01	16.5	17.8	17.2	0.022
zgc:162301	797343	zgc:162301	2.51E-01	8.69E-02	23.2	0.3	23.8	0.022
btr29	797384	bloodthirsty-related gene family, member 29	1.76E-02	2.63E-01	14.5	18.2	16.4	0.022
nt5c1ba	798022	5'-nucleotidase, cytosolic IB a	1.40E-02	2.92E-01	5.5	5.6	5.5	0.022
taok3	798408	TAO kinase 3	2.91E-01	2.29E-01	15.8	17.7	16.8	0.022
si:ch211-217k17.10	798704	si:ch211-217k17.10	2.68E-01	2.07E-01	22.8	1.5	0.2	0.022
tspan13a	798836	tetraspanin 13a	2.54E-01	2.28E-01	13.0	14.2	13.6	0.022
ftr54	799511	finTRIM family, member 54	1.06E-01	2.65E-01	16.7	17.7	17.2	0.022
zgc:175131	799648	zgc:175131	2.60E-01	2.49E-01	15.9	17.4	16.7	0.022
zgc:92606	100000242	zgc:92606	2.44E-01	2.89E-01	12.7	14.2	13.5	0.022
mafl	100000492	v-maf musculoaponeurotic fibrosarcoma (avian) oncoger	3.74E-02	2.66E-01	19.8	19.7	19.8	0.022
glula	100000775	glutamate-ammonia ligase (glutamine synthase) a	6.55E-02	2.64E-01	5.2	4.7	5.0	0.022
LOC100002370	100002370	similar to zinc finger CCCH-type containing 12B	6.97E-02	2.78E-01	21.0	19.0	20.0	0.022
zgc:110779	100002945	zgc:110779	6.24E-02	2.80E-01	14.2	16.7	15.5	0.022
LOC100003592	100003592	probable G-protein coupled receptor 125-like	2.65E-01	2.90E-01	15.7	16.0	15.9	0.022
LOC100004079	100004079	PR domain zinc finger protein 8-like	1.81E-02	2.61E-01	11.5	15.3	13.4	0.022
ddb2	100004133	damage-specific DNA binding protein 2	6.66E-02	2.91E-01	5.2	3.7	4.5	0.022
spryd3	100005036	SPRY domain containing 3	1.16E-01	2.66E-01	16.2	17.8	17.0	0.022
si:ch211-239j15.4	100006377	si:ch211-239j15.4	2.73E-01	2.97E-01	15.5	17.1	16.3	0.022
pcsk7	100009656	proprotein convertase subtilisin/kexin type 7	2.90E-01	1.62E-01	16.1	17.3	16.7	0.022
adat2	100034610	adenosine deaminase, tRNA-specific 2, TAD2 homolog (	5.36E-02	2.90E-01	23.0	1.2	0.1	0.022
zgc:162623	100038768	zgc:162623	1.67E-01	2.77E-01	19.7	17.8	18.8	0.022
mrpl36	100093703	mitochondrial ribosomal protein L36	1.08E-01	2.61E-01	0.7	2.2	1.5	0.022
zgc:175274	100136853	zgc:175274	2.66E-01	1.16E-01	0.0	1.3	0.7	0.022
zgc:174356	100137120	zgc:174356	1.46E-01	2.71E-01	11.0	12.0	11.5	0.022
LOC100148748	100148748	CDK5 and ABL1 enzyme substrate 2-like	2.63E-01	9.74E-02	4.3	2.5	3.4	0.022
LOC100149633	100149633	hypothetical protein LOC100149633	3.06E-02	2.51E-01	16.7	17.5	17.1	0.022
cyp1b1	100150054	cytochrome P450, family 1, subfamily B, polypeptide 1	2.92E-03	2.69E-01	2.2	2.7	2.5	0.022

Table S1: Zebrafish circadian oscillating genes

Gene Symbol	Gene ID	Gene Description	Fisher's g test p value (LD)	Fisher's g test p value (DD)	LD Phase	DD Phase	Phase *	FDR **
cacna2d4	100150428	calcium channel, voltage-dependent, alpha 2/delta subur	4.69E-03	2.52E-01	14.7	16.2	15.4	0.022
LOC100150889	100150889	tapasin-related protein-like	2.89E-01	7.49E-02	16.5	18.3	17.4	0.022
si:dkey-192g7.3	100321723	si:dkey-192g7.3	2.65E-01	1.58E-01	12.5	17.0	14.8	0.022
LOC100329329	100329329	hypothetical protein LOC100329329	8.88E-02	2.75E-01	2.3	4.2	3.3	0.022
LOC100330353	100330353	plakophilin-3-like	3.09E-02	2.53E-01	12.2	14.7	13.5	0.022
LOC100333040	100333040	ADP-ribosylation factor 1-like	1.63E-01	2.61E-01	6.2	6.2	6.2	0.022
man2a2	100333987	mannosidase, alpha, class 2A, member 2	2.77E-01	1.36E-02	18.8	17.3	18.1	0.022
LOC100334439	100334439	CMP-N-acetylneuraminate-beta-galactosamide-alpha-2,3	1.13E-01	2.58E-01	3.4	4.4	3.9	0.022
svil	30112	supervillin	1.48E-02	3.11E-01	12.2	16.5	14.4	0.028
skib	30113	nuclear oncoprotein skib	2.36E-02	3.25E-01	14.8	12.2	13.5	0.028
alcama	30194	activated leukocyte cell adhesion molecule a	3.41E-01	7.42E-02	21.8	18.8	20.3	0.028
hoxa2b	30325	homeo box A2b	3.46E-01	1.12E-01	17.0	18.0	17.5	0.028
fzd5	30364	frizzled homolog 5	3.43E-01	8.76E-02	15.7	17.5	16.6	0.028
psmb6	30388	proteasome (prosome, macropain) subunit, beta type, 6	3.49E-02	3.41E-01	17.3	19.5	18.4	0.028
hoxa10b	30391	homeo box A10b	8.41E-02	3.26E-01	15.3	17.2	16.3	0.028
opn1lw1	30413	opsin 1 (cone pigments), long-wave-sensitive, 1	3.41E-01	2.01E-02	14.3	15.0	14.7	0.028
pcdh8	30456	protocadherin 8	1.33E-02	3.50E-01	15.3	13.2	14.3	0.028
max	30467	myc-associated factor X	3.27E-01	2.48E-01	11.8	22.0	16.9	0.028
hsp90a.1	30591	heat shock protein 90-alpha 1	3.13E-01	1.85E-01	2.1	2.8	2.4	0.028
psme1	30648	proteasome activator subunit 1	2.61E-01	3.15E-01	19.8	21.3	20.6	0.028
pdx1	30721	pancreatic and duodenal homeobox 1	3.49E-01	2.54E-01	14.7	15.5	15.1	0.028
igf2bp3	30967	insulin-like growth factor 2 mRNA binding protein 3	1.49E-01	3.23E-01	23.5	1.2	0.3	0.028
dedd1	58125	death effector domain-containing 1	1.94E-01	3.22E-01	3.7	4.3	4.0	0.028
dmd	83773	dystrophin	3.02E-01	1.70E-01	16.7	17.9	17.3	0.028
irx7	140746	iroquois homeobox protein 7	1.50E-01	3.32E-01	19.2	18.8	19.0	0.028
fshr	195820	follicle stimulating hormone receptor	1.56E-01	3.26E-01	15.3	11.5	13.4	0.028
ppan	317739	peter pan homolog (Drosophila)	3.10E-01	2.97E-01	21.8	0.8	23.3	0.028
wu:fb33b12	321731	wu:fb33b12	2.01E-01	3.00E-01	20.3	18.7	19.5	0.028
prmt1	321974	protein arginine methyltransferase 1	1.39E-01	3.02E-01	0.2	2.6	1.4	0.028
wu:fb49f03	322091	wu:fb49f03	1.25E-01	3.15E-01	15.0	15.3	15.2	0.028
csnk1da	322106	casein kinase 1, delta a	5.31E-03	3.33E-01	2.8	5.0	3.9	0.028

Table S1: Zebrafish circadian oscillating genes

Gene Symbol	Gene ID	Gene Description	Fisher's g test p value (LD)	Fisher's g test p value (DD)	LD Phase	DD Phase	Phase *	FDR **
vdac2	322126	voltage-dependent anion channel 2	3.29E-01	1.75E-01	16.8	18.2	17.5	0.028
agt	322485	angiotensinogen	3.07E-01	1.31E-01	16.3	18.3	17.3	0.028
dus1l	322715	dihydrouridine synthase 1-like (S. cerevisiae)	5.56E-02	3.19E-01	23.5	2.3	0.9	0.028
gas8	323260	growth arrest-specific 8	1.00E-01	3.25E-01	13.2	14.3	13.8	0.028
gtf2a1	323974	general transcription factor IIA, 1	1.42E-03	3.42E-01	3.3	4.5	3.9	0.028
orc6	324256	origin recognition complex, subunit 6	2.00E-01	3.43E-01	3.3	4.7	4.0	0.028
gcnt4a	324510	glucosaminyl (N-acetyl) transferase 4, core 2, a	3.36E-01	3.96E-02	2.5	2.7	2.6	0.028
actr8	326866	ARP8 actin-related protein 8 homolog (yeast)	2.57E-01	3.39E-01	15.8	17.7	16.8	0.028
rnf2	327035	ring finger protein 2	3.48E-01	1.92E-01	14.5	15.7	15.1	0.028
polr1a	327078	polymerase (RNA) I polypeptide A	3.40E-01	3.32E-01	22.2	23.7	23.0	0.028
slc26a11	327138	solute carrier family 26, member 11	2.54E-01	3.32E-01	11.5	11.0	11.3	0.028
shcbp1	333933	SHC SH2-domain binding protein 1	3.39E-01	2.55E-01	10.9	11.4	11.1	0.028
ivns1abpb	334334	influenza virus NS1A binding protein b	8.01E-02	3.19E-01	7.8	8.3	8.1	0.028
calcoco1	334971	calcium binding and coiled-coil domain 1	2.79E-01	3.02E-01	14.6	15.7	15.2	0.028
g3bp1	335512	GTPase activating protein (SH3 domain) binding protein	6.99E-02	3.01E-01	21.6	22.7	22.1	0.028
rcbtb1	336007	regulator of chromosome condensation (RCC1) and BTE	2.34E-01	3.11E-01	13.5	16.7	15.1	0.028
nmnat2	336257	nicotinamide nucleotide adenylyltransferase 2	1.26E-01	3.31E-01	16.0	17.7	16.9	0.028
atp5c1	336957	ATP synthase, H+ transporting, mitochondrial F1 comple	3.01E-01	1.23E-01	1.2	3.8	2.5	0.028
mxc	360145	myxovirus (influenza virus) resistance C	2.58E-01	3.03E-01	17.2	19.4	18.3	0.028
gpm6ba	368225	glycoprotein M6Ba	3.22E-01	1.85E-01	15.8	14.5	15.2	0.028
ubtf	368509	upstream binding transcription factor, RNA polymerase I	3.06E-01	2.23E-01	1.5	21.1	23.3	0.028
slc39a13	368686	solute carrier family 39 (zinc transporter), member 13	3.03E-01	1.73E-01	0.3	1.2	0.8	0.028
heatr5a	368728	HEAT repeat containing 5a	3.41E-01	2.85E-01	14.0	15.5	14.8	0.028
fkbp11	368823	FK506 binding protein 11	3.04E-01	1.42E-01	5.4	5.8	5.6	0.028
tnr	369191	tenascin R (restrictin, janusin)	3.15E-01	6.88E-02	21.8	11.5	16.7	0.028
aldocb	369193	aldolase C, fructose-bisphosphate, b	8.52E-04	3.32E-01	3.2	1.3	2.3	0.028
fyna	373872	FYN oncogene related to SRC, FGR, YES a	3.31E-01	2.46E-01	14.7	15.5	15.1	0.028
tmco1	378980	transmembrane and coiled-coil domains 1	9.09E-02	3.42E-01	23.3	0.5	23.9	0.028
timm17a	386636	translocase of inner mitochondrial membrane 17 homolo	2.62E-02	3.02E-01	23.5	1.2	0.3	0.028
manba	393128	mannosidase, beta A, lysosomal	1.85E-02	3.23E-01	15.5	19.5	17.5	0.028
zgc:56292	393193	zgc:56292	3.16E-01	2.58E-01	20.8	23.8	22.3	0.028

Table S1: Zebrafish circadian oscillating genes

Gene Symbol	Gene ID	Gene Description	Fisher's g test p value (LD)	Fisher's g test p value (DD)	LD Phase	DD Phase	Phase *	FDR **
rnaseh2a	393195	ribonuclease H2, subunit A	3.14E-01	1.59E-01	4.5	5.8	5.2	0.028
slc43a2a	393221	solute carrier family 43, member 2a	3.06E-01	9.68E-03	16.0	18.5	17.3	0.028
zgc:56429	393247	zgc:56429	3.24E-01	8.52E-02	4.8	6.2	5.5	0.028
pwp1	393262	PWP1 homolog (S. cerevisiae)	1.75E-01	3.13E-01	1.3	2.2	1.8	0.028
ccdc25	393359	coiled-coil domain containing 25	3.35E-01	1.98E-02	2.7	3.2	3.0	0.028
prpf31	393476	PRP31 pre-mRNA processing factor 31 homolog (yeast)	3.30E-01	2.23E-01	17.3	16.2	16.8	0.028
vps28	393562	vacuolar protein sorting 28 (yeast)	3.03E-01	2.33E-01	13.5	20.2	16.9	0.028
nat10	393617	N-acetyltransferase 10	3.46E-01	9.86E-02	23.5	1.8	0.7	0.028
ssx2ip	393808	synovial sarcoma, X breakpoint 2 interacting protein	8.82E-02	3.44E-01	14.3	16.5	15.4	0.028
ercc2	393900	excision repair cross-complementing rodent repair deficie	1.93E-01	3.18E-01	16.2	18.5	17.4	0.028
gpr19	393969	G protein-coupled receptor 19	3.45E-01	1.76E-01	16.6	16.8	16.7	0.028
pdk2	393971	pyruvate dehydrogenase kinase, isoenzyme 2	3.27E-01	2.00E-01	8.0	8.5	8.3	0.028
cox15	394181	COX15 homolog, cytochrome c oxidase assembly protei	2.73E-01	3.11E-01	4.5	1.7	3.1	0.028
zgc:55813	399488	zgc:55813	3.32E-01	2.58E-01	16.8	19.2	18.0	0.028
cyp17a1	399692	cytochrome P450, family 17, subfamily A, polypeptide 1	2.11E-01	3.41E-01	11.0	13.5	12.3	0.028
nptnb	403006	neuroplastin b	4.57E-02	3.09E-01	23.3	3.2	1.3	0.028
yipf4	405827	Yip1 domain family, member 4	1.00E-01	3.22E-01	16.0	16.5	16.3	0.028
nlk1	405892	nemo like kinase, type 1	3.02E-01	2.09E-02	14.8	15.7	15.3	0.028
slc13a2	406537	solute carrier family 13 (sodium-dependent dicarboxylate	5.19E-02	3.01E-01	5.3	5.8	5.6	0.028
mcrs1	406557	microspherule protein 1	3.38E-01	5.78E-02	12.8	15.2	14.0	0.028
dnajc7	406579	DnaJ (Hsp40) homolog, subfamily C, member 7	3.29E-01	5.65E-03	16.2	16.0	16.1	0.028
dnaja2	406814	DnaJ (Hsp40) homolog, subfamily A, member 2	6.12E-02	3.09E-01	17.7	19.2	18.5	0.028
si:dkey-37o8.1	407641	si:dkey-37o8.1	3.16E-01	2.62E-01	15.0	16.7	15.9	0.028
ift52	414931	intraflagellar transport protein 52	4.75E-02	3.05E-01	15.7	17.6	16.7	0.028
zgc:91897	432373	zgc:91897	2.70E-02	3.18E-01	3.9	5.8	4.8	0.028
zgc:111821	432385	zgc:111821	3.82E-03	3.18E-01	7.4	6.6	7.0	0.028
ift172	432389	intraflagellar transport 172	2.81E-02	3.01E-01	14.3	16.3	15.3	0.028
zgc:92045	436663	zgc:92045	6.17E-02	3.16E-01	16.5	18.5	17.5	0.028
ccdc85cb	436901	coiled-coil domain containing 85C, b	3.45E-03	3.07E-01	13.8	15.0	14.4	0.028
timm10	436951	translocase of inner mitochondrial membrane 10 homolo	9.01E-02	3.14E-01	23.2	1.2	0.2	0.028
zgc:92610	436978	zgc:92610	5.06E-03	3.18E-01	14.5	17.5	16.0	0.028

Table S1: Zebrafish circadian oscillating genes

Gene Symbol	Gene ID	Gene Description	Fisher's g test p value (LD)	Fisher's g test p value (DD)	LD Phase	DD Phase	Phase *	FDR **
zgc:91985	445105	zgc:91985	2.07E-02	3.01E-01	8.2	10.0	9.1	0.028
mrpl19	445150	mitochondrial ribosomal protein L19	3.19E-01	3.35E-02	22.5	1.4	24.0	0.028
tm9sf1	445156	transmembrane 9 superfamily member 1	3.28E-01	2.76E-01	0.8	0.7	0.8	0.028
slc16a9b	445158	solute carrier family 16 (monocarboxylic acid transporters	3.17E-01	2.08E-02	16.1	19.2	17.7	0.028
mfsd2ab	445176	major facilitator superfamily domain containing 2ab	6.95E-02	3.20E-01	17.3	18.9	18.1	0.028
zgc:101053	445184	zgc:101053	3.04E-01	6.55E-02	7.0	6.2	6.6	0.028
med20	445191	mediator complex subunit 20	3.14E-01	2.85E-01	3.0	4.8	3.9	0.028
mettl16	445217	methyltransferase like 16	1.99E-01	3.19E-01	0.5	2.5	1.5	0.028
ddx56	445399	DEAD (Asp-Glu-Ala-Asp) box polypeptide 56	3.07E-01	4.37E-02	21.8	0.0	22.9	0.028
gle1	445408	GLE1 RNA export mediator homolog (yeast)	3.45E-01	6.33E-02	16.8	19.3	18.1	0.028
cdh5	445471	cadherin 5	3.06E-01	1.13E-01	22.8	21.5	22.2	0.028
pnp5b	447889	purine nucleoside phosphorylase 5b	1.89E-01	3.21E-01	2.2	23.7	1.0	0.028
tmem56b	449652	transmembrane protein 56b	6.84E-02	3.16E-01	3.0	3.5	3.2	0.028
glrx	449769	glutaredoxin (thioltransferase)	3.25E-01	2.46E-01	2.7	4.8	3.7	0.028
dtwd1	449778	DTW domain containing 1	1.74E-01	3.05E-01	19.7	20.2	20.0	0.028
dhrs3b	450050	dehydrogenase/reductase (SDR family) member 3b	3.06E-01	2.34E-02	1.0	2.7	1.9	0.028
acot8	450052	acyl-CoA thioesterase 8	2.91E-01	3.15E-01	22.8	23.5	23.2	0.028
zgc:103652	492492	zgc:103652	3.42E-01	2.38E-01	0.8	2.3	1.6	0.028
def8	492523	differentially expressed in FDCP 8 homolog (mouse)	1.04E-03	3.08E-01	15.6	18.1	16.8	0.028
si:rp71-39b20.7	492647	si:rp71-39b20.7	2.00E-01	3.03E-01	6.3	7.0	6.7	0.028
dhrs13	492783	dehydrogenase/reductase (SDR family) member 13	1.08E-01	3.08E-01	9.8	9.8	9.8	0.028
zgc:101716	492784	zgc:101716	3.44E-01	4.86E-02	19.7	21.2	20.5	0.028
atg5	494180	ATG5 autophagy related 5 homolog (S. cerevisiae)	3.27E-01	3.30E-01	14.8	17.5	16.2	0.028
il15l	494451	interleukin 15, like	2.61E-01	3.22E-01	1.5	1.8	1.7	0.028
si:ch211-150a22.1	541317	si:ch211-150a22.1	3.07E-01	3.35E-01	15.8	18.2	17.0	0.028
lamp2	541406	lysosomal membrane glycoprotein 2	1.91E-02	3.27E-01	15.9	18.4	17.1	0.028
zgc:110537	541544	zgc:110537	3.28E-01	2.13E-01	0.2	21.3	22.8	0.028
zgc:110307	550253	zgc:110307	2.45E-01	3.08E-01	13.3	18.0	15.7	0.028
fcf1	550264	FCF1 small subunit (SSU) processome component homo	1.57E-01	3.39E-01	0.7	1.8	1.2	0.028
npepl1	550383	aminopeptidase-like 1	3.99E-05	3.13E-01	5.8	4.3	5.1	0.028
zgc:112095	550448	zgc:112095	3.17E-01	1.06E-01	13.8	12.8	13.3	0.028

Table S1: Zebrafish circadian oscillating genes

Gene Symbol	Gene ID	Gene Description	Fisher's g test p value (LD)	Fisher's g test p value (DD)	LD Phase	DD Phase	Phase *	FDR **
shisa4	550567	shisa homolog 4 (Xenopus laevis)	1.97E-01	3.35E-01	17.8	16.0	16.9	0.028
zgc:110609	550594	zgc:110609	4.82E-02	3.43E-01	16.3	18.2	17.2	0.028
clcn7	553351	chloride channel 7	1.11E-01	3.39E-01	15.0	17.3	16.2	0.028
tatdn3	553592	TatD DNase domain containing 3	1.62E-01	3.20E-01	4.8	3.0	3.9	0.028
eri1	553641	exoribonuclease 1	5.00E-02	3.44E-01	23.5	0.8	0.2	0.028
cyb5r4	553685	cytochrome b5 reductase 4	3.47E-01	8.21E-03	15.0	17.5	16.3	0.028
zgc:112231	553692	zgc:112231	3.12E-01	2.59E-02	19.8	21.5	20.7	0.028
creb3l3	553700	cAMP responsive element binding protein 3-like 3	3.04E-01	6.16E-02	5.8	8.2	7.0	0.028
zgc:112365	553720	zgc:112365	1.75E-01	3.18E-01	22.7	1.2	24.0	0.028
mtmr1a	553750	myotubularin related protein 1a	3.49E-01	4.52E-02	12.6	17.6	15.1	0.028
fahd1	553762	fumarylacetoacetate hydrolase domain containing 1	1.55E-01	3.45E-01	1.3	2.7	2.0	0.028
zgc:112089	553799	zgc:112089	7.27E-02	3.14E-01	1.2	3.7	2.5	0.028
hbp1	554137	HMG-box transcription factor 1	3.10E-01	5.87E-02	11.5	13.5	12.5	0.028
si:rp71-1i20.2	554678	si:rp71-1i20.2	1.43E-01	3.35E-01	23.5	2.0	0.8	0.028
wu:fa07b03	555414	wu:fa07b03	7.22E-02	3.38E-01	14.7	15.2	15.0	0.028
si:dkey-266j9.3	555595	si:dkey-266j9.3	2.24E-01	3.15E-01	19.3	22.0	20.7	0.028
zgc:153293	555710	zgc:153293	1.95E-03	3.20E-01	12.5	15.7	14.1	0.028
LOC555911	555911	atrial natriuretic peptide receptor 1-like	6.65E-02	3.26E-01	16.2	16.0	16.1	0.028
si:ch211-214j24.7	556080	si:ch211-214j24.7	3.02E-01	4.24E-02	14.3	14.2	14.3	0.028
gxylt1b	556315	glucoside xylosyltransferase 1b	3.41E-01	3.07E-01	14.2	15.8	15.0	0.028
si:ch211-238n5.4	556432	si:ch211-238n5.4	3.27E-01	8.52E-02	16.0	17.8	16.9	0.028
zgc:172324	556797	zgc:172324	3.48E-01	2.30E-01	13.8	16.2	15.0	0.028
abcg5	557317	ATP-binding cassette, sub-family G (WHITE), member 5	4.83E-02	3.03E-01	20.0	22.2	21.1	0.028
tmem181	557799	transmembrane protein 181	1.39E-01	3.17E-01	1.4	1.5	1.5	0.028
abca12	558335	ATP-binding cassette, sub-family A (ABC1), member 12	1.25E-02	3.46E-01	1.8	1.5	1.7	0.028
LOC559192	559192	novel protein similar to human and mouse peptidyl-tRNA	1.68E-02	3.16E-01	21.7	22.8	22.3	0.028
LOC559258	559258	uncharacterized protein C3orf23-like	1.14E-03	3.17E-01	11.3	13.8	12.6	0.028
LOC560089	560089	multidrug and toxin extrusion protein 1-like	3.50E-01	1.16E-01	10.5	11.0	10.8	0.028
esrp1	560190	epithelial splicing regulatory protein 1	8.07E-02	3.35E-01	0.5	6.0	3.3	0.028
eef1db	560745	elongation factor-1, delta, b	2.29E-01	3.05E-01	18.5	17.2	17.9	0.028
antxr2b	560841	anthrax toxin receptor 2b	1.24E-01	3.47E-01	14.5	16.5	15.5	0.028

Table S1: Zebrafish circadian oscillating genes

Gene Symbol	Gene ID	Gene Description	Fisher's g test p value (LD)	Fisher's g test p value (DD)	LD Phase	DD Phase	Phase *	FDR **
zgc:158649	561239	zgc:158649	3.37E-01	2.71E-01	16.3	19.2	17.8	0.028
LOC561497	561497	transmembrane channel-like protein 5-like	3.38E-01	4.04E-02	19.0	19.7	19.4	0.028
zgc:153718	561624	zgc:153718	5.91E-02	3.09E-01	15.3	18.5	16.9	0.028
si:dkey-275b16.2	562105	si:dkey-275b16.2	3.09E-01	2.34E-01	14.0	12.8	13.4	0.028
zgc:158784	562138	zgc:158784	7.23E-02	3.12E-01	23.5	4.3	1.9	0.028
uhrf1bp1l	562208	UHRF1 (ICBP90) binding protein 1-like	3.45E-01	8.80E-02	15.8	16.0	15.9	0.028
LOC562364	562364	adenosine receptor A3-like	6.01E-02	3.25E-01	13.2	18.5	15.9	0.028
LOC562403	562403	hypothetical LOC562403	3.28E-01	5.99E-02	21.7	10.5	16.1	0.028
cldn2	562525	claudin 2	1.65E-01	3.03E-01	3.5	21.1	0.3	0.028
zgc:194780	562626	zgc:194780	3.37E-01	2.21E-01	14.8	16.0	15.4	0.028
LOC563450	563450	similar to Potential phospholipid-transporting ATPase ID	2.52E-02	3.29E-01	19.2	19.7	19.5	0.028
fam63a	563470	family with sequence similarity 63, member A	3.40E-01	3.50E-01	14.3	16.7	15.5	0.028
tiparp	563506	TCDD-inducible poly(ADP-ribose) polymerase	2.43E-01	3.48E-01	15.2	17.2	16.2	0.028
nppcl	563937	natriuretic peptide precursor C-like protein	3.46E-01	3.19E-01	16.2	15.7	16.0	0.028
pik3r4	564088	phosphoinositide-3-kinase, regulatory subunit 4	3.43E-01	2.85E-02	14.0	15.0	14.5	0.028
LOC564392	564392	acid sphingomyelinase-like phosphodiesterase 3b-like	3.12E-01	1.45E-02	4.3	4.0	4.2	0.028
zgc:113028	565041	zgc:113028	3.35E-01	2.28E-03	11.2	15.7	13.5	0.028
comtb	565370	catechol-O-methyltransferase b	6.74E-02	3.45E-01	14.0	16.3	15.2	0.028
slc4a2a	566075	solute carrier family 4, anion exchanger, member 2a	3.32E-01	1.63E-01	15.0	19.5	17.3	0.028
mcm6	566409	MCM6 minichromosome maintenance deficient 6, mitotir	2.87E-01	3.21E-01	2.1	3.9	3.0	0.028
gnsa	566506	glucosamine (N-acetyl)-6-sulfatase a	2.33E-01	3.15E-01	14.5	16.8	15.7	0.028
si:dkey-190g11.7	567309	si:dkey-190g11.7	1.46E-01	3.25E-01	0.7	2.0	1.4	0.028
si:dkey-106c17.2	567437	si:dkey-106c17.2	3.23E-01	2.32E-01	13.8	13.3	13.6	0.028
agl	567798	amylo-1, 6-glucosidase, 4-alpha-glucanotransferase	2.18E-01	3.48E-01	18.5	17.7	18.1	0.028
wu:fj02d07	567862	wu:fj02d07	3.15E-01	3.30E-01	17.7	18.5	18.1	0.028
tecpr2	569212	tectonin beta-propeller repeat containing 2	3.04E-01	2.36E-01	16.5	17.3	16.9	0.028
LOC569234	569234	hypothetical LOC569234	1.34E-02	3.08E-01	16.5	17.8	17.2	0.028
si:ch211-202b2.2	569550	si:ch211-202b2.2	1.17E-01	3.15E-01	11.2	14.2	12.7	0.028
LOC569770	569770	cytochrome c oxidase subunit 8B, mitochondrial-like	3.34E-01	1.91E-01	5.0	7.3	6.2	0.028
chd2	569795	chromodomain helicase DNA binding protein 2	3.31E-01	2.98E-01	15.3	16.8	16.1	0.028
igsf21a	569872	immunoglobin superfamily, member 21a	7.61E-02	3.15E-01	22.2	21.2	21.7	0.028

Table S1: Zebrafish circadian oscillating genes

Gene Symbol	Gene ID	Gene Description	Fisher's g test p value (LD)	Fisher's g test p value (DD)	LD Phase	DD Phase	Phase *	FDR **
si:dkey-18f7.1	570779	si:dkey-18f7.1	3.01E-01	1.54E-01	20.5	0.8	22.7	0.028
trap1	571959	TNF receptor-associated protein 1	2.25E-01	3.33E-01	0.3	0.5	0.4	0.028
im:7159197	606628	im:7159197	3.40E-01	1.12E-01	19.8	17.7	18.8	0.028
nedd4a	619412	neural precursor cell expressed, developmentally down-r	3.00E-01	5.80E-02	18.2	17.5	17.9	0.028
ihha	619520	Indian hedgehog homolog a	2.09E-01	3.29E-01	19.4	19.0	19.2	0.028
gsg1l	664746	gsg1-like	2.13E-01	3.44E-01	3.0	3.8	3.4	0.028
id4	664761	inhibitor of DNA binding 4	7.40E-02	3.14E-01	14.8	16.0	15.4	0.028
f9b	678552	coagulation factor IXb	3.33E-01	1.58E-03	6.0	4.0	5.0	0.028
fam69a	678606	family with sequence similarity 69, member A	2.18E-03	3.10E-01	13.7	16.7	15.2	0.028
pmp22b	678607	peripheral myelin protein 22b	3.38E-01	2.66E-02	1.0	2.8	1.9	0.028
crb3b	724019	crumbs homolog 3b	3.23E-01	4.03E-02	21.2	22.8	22.0	0.028
cyb5r2	751641	cytochrome b5 reductase 2	3.12E-01	8.49E-02	2.1	3.6	2.8	0.028
zgc:153753	767716	zgc:153753	5.21E-03	3.33E-01	15.7	17.2	16.5	0.028
zgc:153894	767769	zgc:153894	3.42E-01	2.42E-01	10.3	9.0	9.7	0.028
sult2st3	777792	sulfotransferase family 2, cytosolic sulfotransferase 3	1.33E-01	3.03E-01	12.3	12.8	12.6	0.028
zgc:158263	791197	zgc:158263	1.87E-02	3.19E-01	3.5	4.8	4.2	0.028
LOC794494	794494	protein phosphatase 1 regulatory subunit 16A-like	3.07E-01	1.70E-02	1.0	2.5	1.8	0.028
metap2l	794589	methionyl aminopeptidase 2 like	3.01E-01	1.29E-01	18.2	20.7	19.5	0.028
zgc:172238	796270	zgc:172238	4.20E-02	3.23E-01	14.7	16.8	15.8	0.028
arhgap20	796434	Rho GTPase activating protein 20	1.25E-01	3.45E-01	16.5	17.3	16.9	0.028
hmx1	797503	H6 homeo box 1	1.60E-01	3.30E-01	14.8	15.8	15.3	0.028
LOC797939	797939	Fbxl16 protein-like	2.01E-01	3.28E-01	1.5	0.3	0.9	0.028
slc2a9l2	798023	solute carrier family 2 (facilitated glucose transporter), m	1.22E-01	3.02E-01	7.8	9.2	8.5	0.028
si:ch211-169p10.1	799619	si:ch211-169p10.1	5.90E-02	3.42E-01	13.8	15.3	14.6	0.028
LOC799886	799886	hypothetical LOC799886	1.78E-02	3.40E-01	16.3	14.2	15.3	0.028
march6	100002072	membrane-associated ring finger (C3HC4) 6	3.25E-01	3.87E-02	16.0	17.0	16.5	0.028
ar	100005148	androgen receptor	3.56E-02	3.09E-01	5.0	5.3	5.2	0.028
cox11	100005158	COX11 cytochrome c oxidase assembly homolog (yeast)	2.39E-03	3.37E-01	4.0	1.5	2.8	0.028
LOC100005645	100005645	hypothetical protein LOC100005645	1.59E-01	3.41E-01	0.8	3.5	2.2	0.028
LOC100007768	100007768	similar to mitochondrial ribosomal protein L52	1.64E-01	3.30E-01	0.5	1.2	0.9	0.028
si:ch211-51f10.1	100034470	si:ch211-51f10.1	2.60E-02	3.41E-01	16.7	18.2	17.5	0.028

Table S1: Zebrafish circadian oscillating genes

Gene Symbol	Gene ID	Gene Description	Fisher's g test p value (LD)	Fisher's g test p value (DD)	LD Phase	DD Phase	Phase *	FDR **
zgc:165647	100101649	zgc:165647	5.77E-03	3.00E-01	7.0	9.7	8.4	0.028
fam177a1	100126028	family with sequence similarity 177, member A1	4.38E-02	3.38E-01	13.7	15.8	14.8	0.028
cyth4b	100141338	cytohesin 4b	8.32E-02	3.08E-01	13.8	17.2	15.5	0.028
LOC100148286	100148286	radial spokehead-like 1-like	3.12E-01	3.38E-01	16.3	20.0	18.2	0.028
LOC100148604	100148604	fizzy-related protein homolog	1.86E-01	3.35E-01	15.3	16.3	15.8	0.028
cmbl	100151256	carboxymethylenebutenolidase-like (Pseudomonas)	2.09E-01	3.45E-01	9.2	14.8	12.0	0.028
abcd3b	100170781	ATP-binding cassette, sub-family D (ALD), member 3b	3.44E-01	2.64E-01	17.5	23.2	20.4	0.028
zgc:194398	100189615	zgc:194398	7.79E-02	3.08E-01	5.7	6.2	6.0	0.028
mmp17b	100322027	matrix metallopeptidase 17b	2.21E-02	3.21E-01	22.0	23.7	22.9	0.028
LOC100331289	100331289	sucrase-isomaltase, intestinal-like	5.96E-02	3.06E-01	1.3	3.5	2.4	0.028
LOC100332086	100332086	hypothetical protein LOC100332086	1.09E-01	3.14E-01	14.5	14.7	14.6	0.028
LOC100332166	100332166	polymeric immunoglobulin receptor-like	3.25E-01	5.31E-02	16.2	17.3	16.8	0.028
jak2b	30298	Janus kinase 2b	3.54E-01	2.80E-01	12.5	16.8	14.7	0.034
hoxc11a	30403	homeo box C11a	3.74E-01	2.36E-01	20.0	15.3	17.7	0.034
exorh	30459	extra-ocular rhodopsin	6.98E-03	3.50E-01	17.3	18.0	17.7	0.034
ck2a2a	30488	casein kinase 2 alpha 2a	4.12E-02	3.86E-01	16.3	19.5	17.9	0.034
mef2a	30564	myocyte enhancer factor 2a	5.76E-02	3.64E-01	0.8	2.2	1.5	0.034
nkx2.3	30698	NK2 transcription factor related 3	9.30E-02	3.91E-01	15.0	16.8	15.9	0.034
spon1a	58027	spondin 1a	3.68E-01	8.92E-02	18.5	10.2	14.4	0.034
trpc4apa	58090	transient receptor potential cation channel, subfamily C,	3.06E-01	3.68E-01	12.9	14.4	13.6	0.034
pbx1a	58138	pre-B-cell leukemia transcription factor 1a	5.63E-02	3.96E-01	14.5	8.7	11.6	0.034
six2a	83415	sine oculis-related homeobox 2a	3.99E-02	3.72E-01	16.8	17.8	17.3	0.034
igf1	114433	insulin-like growth factor 1	2.05E-01	3.77E-01	16.2	23.7	20.0	0.034
adh5	116517	alcohol dehydrogenase 5	1.52E-01	3.72E-01	16.8	17.5	17.2	0.034
uxs1	192315	UDP-glucuronic acid decarboxylase 1	2.77E-01	3.92E-01	16.5	19.2	17.9	0.034
pcdh10a	259186	protocadherin 10a	2.13E-01	3.91E-01	15.2	16.7	16.0	0.034
unc45b	266640	unc-45 homolog B (C. elegans)	3.18E-01	3.51E-01	2.2	3.3	2.8	0.034
lmo1	280646	LIM domain only 1	3.87E-01	3.62E-01	16.3	15.2	15.8	0.034
si:ch211-245h7.2	322801	si:ch211-245h7.2	3.62E-01	1.74E-01	23.5	2.2	0.9	0.034
slc35b4	323737	solute carrier family 35, member B4	3.03E-01	3.80E-01	23.8	2.8	1.3	0.034
st5	323827	suppression of tumorigenicity 5	2.42E-01	3.71E-01	17.3	16.5	16.9	0.034

Table S1: Zebrafish circadian oscillating genes

Gene Symbol	Gene ID	Gene Description	Fisher's g test p value (LD)	Fisher's g test p value (DD)	LD Phase	DD Phase	Phase *	FDR **
stub1	324243	STIP1 homology and U-Box containing protein 1	2.12E-01	3.63E-01	15.7	17.8	16.8	0.034
eif2s2	324365	eukaryotic translation initiation factor 2, subunit 2 beta	6.99E-03	3.65E-01	23.7	23.7	23.7	0.034
thoc5	325064	THO complex 5	1.50E-01	3.77E-01	17.5	18.4	17.9	0.034
ttpa	325906	tocopherol (alpha) transfer protein (ataxia (Friedreich-like	3.87E-02	3.63E-01	7.3	10.3	8.8	0.034
ccnl1	326088	cyclin L1	3.92E-03	3.52E-01	19.0	20.0	19.5	0.034
rnf146	334065	ring finger protein 146	2.49E-02	3.92E-01	15.3	15.3	15.3	0.034
mark3	334300	MAP/microtubule affinity-regulating kinase 3	5.23E-03	3.55E-01	16.7	17.8	17.3	0.034
mbd1	334386	methyl-CpG binding domain protein 1	1.02E-01	3.57E-01	16.2	16.7	16.5	0.034
eif3m	334626	eukaryotic translation initiation factor 3, subunit M	3.53E-01	1.68E-01	17.0	17.7	17.4	0.034
wdr48	334682	WD repeat domain 48	1.45E-01	3.78E-01	14.2	13.5	13.9	0.034
wu:fa10g06	334957	wu:fa10g06	2.06E-01	3.52E-01	21.7	21.6	21.6	0.034
zgc:73136	334982	zgc:73136	3.75E-01	1.89E-01	0.6	1.2	0.9	0.034
hspa4a	335865	heat shock protein 4a	3.76E-02	3.83E-01	1.3	2.3	1.8	0.034
brd8	337414	bromodomain containing 8	3.32E-01	3.71E-01	15.2	14.0	14.6	0.034
wu:fj81c05	337526	wu:fj81c05	1.19E-02	3.79E-01	7.2	3.5	5.4	0.034
bsg	337692	basigin	3.76E-01	2.14E-01	3.6	20.9	0.3	0.034
cntn1a	353150	contactin 1a	3.15E-01	3.99E-01	20.5	18.0	19.3	0.034
opn1mw2	360151	opsin 1 (cone pigments), medium-wave-sensitive, 2	3.75E-01	2.68E-01	8.7	9.3	9.0	0.034
dhrs1	368670	dehydrogenase/reductase (SDR family) member 1	1.62E-01	3.77E-01	5.5	4.3	4.9	0.034
p2rx5	373879	purinergic receptor P2X, ligand-gated ion channel, 5	3.95E-01	2.71E-01	17.0	18.2	17.6	0.034
rdh1	378440	retinol dehydrogenase 1	3.62E-01	4.29E-02	7.1	8.3	7.7	0.034
taf12	386637	TAF12 RNA polymerase II, TATA box binding protein (TE	1.42E-01	3.78E-01	15.0	16.0	15.5	0.034
rbm19	387255	RNA binding motif protein 19	3.88E-01	1.78E-01	12.1	3.0	7.5	0.034
sybl1	393236	synaptobrevin-like 1	3.97E-01	5.00E-02	16.0	18.2	17.1	0.034
chd1l	393283	chromodomain helicase DNA binding protein 1-like	3.58E-01	3.44E-02	7.3	8.5	7.9	0.034
pygmb	393444	phosphorylase, glycogen (muscle) b	1.40E-02	3.86E-01	3.2	4.3	3.8	0.034
tmem30c	393568	transmembrane protein 30C	3.95E-01	1.93E-01	16.2	11.0	13.6	0.034
fhit	393713	fragile histidine triad gene	3.56E-01	3.39E-02	23.2	2.2	0.7	0.034
zgc:77816	393843	zgc:77816	9.75E-02	3.63E-01	13.9	16.4	15.2	0.034
psmd13	393922	proteasome (prosome, macropain) 26S subunit, non-ATF	1.52E-02	3.55E-01	17.8	19.7	18.8	0.034
upf3b	393929	UPF3 regulator of nonsense transcripts homolog B (yeas	1.13E-01	3.98E-01	13.5	16.5	15.0	0.034

Table S1: Zebrafish circadian oscillating genes

Gene Symbol	Gene ID	Gene Description	Fisher's g test p value (LD)	Fisher's g test p value (DD)	LD Phase	DD Phase	Phase *	FDR **
efhc1	393942	EF-hand domain (C-terminal) containing 1	2.76E-01	3.97E-01	14.7	18.0	16.4	0.034
mcts1	394046	malignant T cell amplified sequence 1	2.14E-01	3.92E-01	0.8	2.0	1.4	0.034
sars2	394154	seryl-tRNA synthetase 2	4.93E-02	3.63E-01	2.0	3.8	2.9	0.034
zgc:73226	402792	zgc:73226	3.85E-01	3.04E-01	4.0	2.2	3.1	0.034
zgc:174506	402825	zgc:174506	2.97E-01	3.75E-01	4.5	5.8	5.2	0.034
si:ch211-35n24.1	402857	si:ch211-35n24.1	2.99E-01	3.73E-01	14.2	16.2	15.2	0.034
mgp	402937	matrix Gla protein	2.28E-01	3.75E-01	2.7	2.2	2.4	0.034
papolb	404041	poly(A) polymerase beta (testis specific)	1.82E-01	3.78E-01	17.2	18.8	18.0	0.034
pacsin2	404601	protein kinase C and casein kinase substrate in neurons	3.87E-01	3.47E-01	19.8	21.2	20.5	0.034
cyyr1	405818	cysteine and tyrosine-rich protein 1	1.29E-01	3.90E-01	0.3	1.8	1.1	0.034
gmfb	406384	glia maturation factor, beta	3.59E-01	1.31E-01	3.8	4.5	4.2	0.034
ak3	406404	adenylate kinase 3	3.73E-01	2.63E-01	11.8	13.0	12.4	0.034
vta1	406414	Vps20-associated 1 homolog (S. cerevisiae)	2.51E-02	3.84E-01	14.2	16.2	15.2	0.034
dhdhl	406433	dihydrodiol dehydrogenase (dimeric), like	8.65E-03	3.96E-01	11.3	10.5	10.9	0.034
amfr	406442	autocrine motility factor receptor	2.06E-01	3.60E-01	13.5	14.3	13.9	0.034
eif5a2	406546	eukaryotic translation initiation factor 5A2	3.06E-01	3.80E-01	0.5	21.5	23.0	0.034
rpf2	406587	ribosome production factor 2 homolog (S. cerevisiae)	3.61E-01	1.43E-01	0.8	1.0	0.9	0.034
nsun5	406690	NOP2/Sun domain family, member 5	3.71E-01	3.39E-01	20.5	23.2	21.9	0.034
nedd1	406827	neural precursor cell expressed, developmentally down-r	2.62E-02	3.50E-01	13.3	13.7	13.5	0.034
sccpdhb	407665	saccharopine dehydrogenase b	1.16E-02	3.95E-01	15.7	17.7	16.7	0.034
fabp7b	407736	fatty acid binding protein 7, brain, b	3.59E-01	2.91E-02	4.8	3.5	4.2	0.034
rfng	414848	radical fringe homolog (Drosophila)	1.60E-02	3.58E-01	13.3	15.5	14.4	0.034
nr4a1	431720	nuclear receptor subfamily 4, group A, member 1	2.65E-01	3.65E-01	1.7	2.3	2.0	0.034
slc30a5	436594	solute carrier family 30 (zinc transporter), member 5	4.45E-02	3.54E-01	16.0	19.2	17.6	0.034
cd36	436636	CD36 antigen	1.18E-02	3.74E-01	1.5	2.8	2.2	0.034
zgc:92647	436707	zgc:92647	7.32E-02	3.97E-01	16.0	18.8	17.4	0.034
zgc:92851	436766	zgc:92851	3.94E-03	3.66E-01	3.8	3.0	3.4	0.034
atxn7l2b	436928	ataxin 7-like 2b	3.78E-01	2.71E-03	14.4	18.0	16.2	0.034
zgc:100799	437005	zgc:100799	3.06E-02	3.67E-01	22.7	22.5	22.6	0.034
fkbp14	445077	FK506 binding protein 14	3.93E-01	2.95E-01	2.3	3.5	2.9	0.034
paip1	445110	poly(A) binding protein interacting protein 1	1.73E-01	3.60E-01	16.0	18.0	17.0	0.034

Table S1: Zebrafish circadian oscillating genes

Gene Symbol	Gene ID	Gene Description	Fisher's g test p value (LD)	Fisher's g test p value (DD)	LD Phase	DD Phase	Phase *	FDR **
rfc5	445385	replication factor C (activator 1) 5	3.78E-01	3.15E-01	5.0	6.3	5.7	0.034
nop10	445391	NOP10 ribonucleoprotein homolog (yeast)	2.50E-01	3.85E-01	1.8	3.7	2.8	0.034
myo6a	445473	myosin VIa	3.81E-01	3.86E-02	16.7	13.2	15.0	0.034
zgc:92375	445475	zgc:92375	1.84E-01	3.83E-01	4.0	5.0	4.5	0.034
fuca2	445487	fucosidase, alpha-L- 2, plasma	1.34E-01	3.75E-01	13.6	14.2	13.9	0.034
rnf103	447861	ring finger protein 103	3.20E-01	3.84E-01	13.8	15.8	14.8	0.034
dscc1	447872	defective in sister chromatid cohesion 1 homolog (S. cer	1.67E-02	3.65E-01	4.0	5.5	4.8	0.034
zgc:103761	447915	zgc:103761	3.30E-01	3.94E-01	1.5	3.0	2.3	0.034
zgc:103670	447926	zgc:103670	3.11E-02	3.67E-01	15.3	15.0	15.1	0.034
park7	449674	parkinson disease (autosomal recessive, early onset) 7	1.34E-02	3.64E-01	5.2	4.6	4.9	0.034
ap3s1	449791	adaptor-related protein complex 3, sigma 1 subunit	2.90E-01	3.55E-01	23.8	0.5	0.2	0.034
rbx1	449846	ring-box 1	3.60E-01	1.28E-01	23.0	0.5	23.8	0.034
tlcd1	450051	TLC domain containing 1	3.77E-01	3.27E-01	1.0	3.5	2.3	0.034
zgc:103668	492489	zgc:103668	3.86E-01	3.35E-01	7.5	7.7	7.6	0.034
si:dkey-42i9.4	492503	si:dkey-42i9.4	4.31E-02	3.92E-01	11.7	13.5	12.6	0.034
casp9	492763	caspase 9, apoptosis-related cysteine protease	3.71E-01	3.11E-01	13.5	16.0	14.8	0.034
gpr137ba	492792	G protein-coupled receptor 137ba	3.69E-01	2.06E-01	12.8	14.0	13.4	0.034
zgc:101609	492814	zgc:101609	3.62E-01	2.90E-01	0.5	2.7	1.6	0.034
zgc:103710	494039	zgc:103710	5.43E-02	3.89E-01	16.7	18.2	17.5	0.034
slc7a2	494041	solute carrier family 7 (cationic amino acid transporter, y-	3.53E-01	1.04E-01	0.7	2.2	1.5	0.034
arhgef7b	494081	Rho guanine nucleotide exchange factor (GEF) 7b	8.53E-04	3.50E-01	13.3	13.9	13.6	0.034
nop58	494152	NOP58 ribonucleoprotein homolog (yeast)	1.11E-02	3.88E-01	21.2	23.3	22.3	0.034
crygs1	503587	crystallin, gamma S1	3.51E-01	1.11E-01	21.8	5.2	1.5	0.034
bmp6	503761	bone morphogenetic protein 6	9.69E-03	3.78E-01	14.7	16.2	15.5	0.034
zgc:113409	541361	zgc:113409	2.52E-01	3.61E-01	14.0	16.8	15.4	0.034
zgc:103561	541403	zgc:103561	3.95E-01	3.56E-01	15.7	13.5	14.6	0.034
zgc:103511	541407	zgc:103511	3.70E-03	3.89E-01	23.2	0.0	23.6	0.034
zgc:113425	541425	zgc:113425	7.07E-03	3.72E-01	12.8	14.5	13.7	0.034
pik3c3	548346	phosphoinositide-3-kinase, class 3	1.74E-03	3.83E-01	15.0	16.3	15.7	0.034
zgc:112515	550458	zgc:112515	1.84E-01	3.74E-01	17.2	18.8	18.0	0.034
zgc:110185	550532	zgc:110185	3.70E-01	3.12E-01	14.5	17.8	16.2	0.034

Table S1: Zebrafish circadian oscillating genes

Gene Symbol	Gene ID	Gene Description	Fisher's g test p value (LD)	Fisher's g test p value (DD)	LD Phase	DD Phase	Phase *	FDR **
si:ch211-79h18.3	553029	si:ch211-79h18.3	3.66E-01	9.29E-02	5.5	6.3	5.9	0.034
nova2	553201	neuro-oncological ventral antigen 2	3.89E-01	3.18E-01	22.7	17.5	20.1	0.034
crot	553204	carnitine O-octanoyltransferase	3.57E-01	2.98E-01	12.1	12.5	12.3	0.034
LOC553492	553492	hypothetical protein LOC553492	1.47E-01	3.81E-01	21.4	22.8	22.1	0.034
zgc:110158	553590	zgc:110158	3.77E-01	3.40E-02	17.0	14.8	15.9	0.034
nip7	553616	nuclear import 7 homolog (S. cerevisiae)	3.55E-01	2.50E-01	23.3	1.5	0.4	0.034
cysltr1	553675	cysteinyl leukotriene receptor 1	3.06E-01	3.72E-01	14.8	16.5	15.7	0.034
zgc:112102	553678	zgc:112102	3.55E-01	4.15E-03	17.7	20.3	19.0	0.034
gdap1	553702	ganglioside-induced differentiation-associated protein 1	2.38E-01	3.92E-01	14.8	16.2	15.5	0.034
mrps24	553711	mitochondrial ribosomal protein S24	3.91E-01	9.43E-02	2.2	3.1	2.6	0.034
tbc1d17	554092	TBC1 domain family, member 17	2.07E-01	3.53E-01	12.0	14.8	13.4	0.034
pdlim3a	554148	PDZ and LIM domain 3a	3.59E-01	3.58E-01	5.5	5.3	5.4	0.034
im:6912374	555254	im:6912374	2.07E-01	3.97E-01	17.0	18.0	17.5	0.034
fam184a	555640	family with sequence similarity 184, member A	6.45E-02	3.66E-01	21.2	19.3	20.3	0.034
wfs1b	556580	Wolfram syndrome 1b (wolframin)	5.05E-02	3.51E-01	14.2	16.8	15.5	0.034
irgq2	556650	immunity-related GTPase family, q2	3.76E-01	2.94E-01	1.2	1.7	1.5	0.034
im:7140055	556704	im:7140055	6.30E-02	3.78E-01	1.2	3.2	2.2	0.034
si:ch211-149a22.1	556993	si:ch211-149a22.1	3.63E-01	1.00E-02	15.7	16.3	16.0	0.034
LOC557008	557008	novel protein similar to vertebrate aldehyde dehydrogena	3.57E-01	1.18E-02	4.8	2.3	3.6	0.034
fam102bb	557909	family with sequence similarity 102, member B, b	2.64E-01	3.75E-01	13.8	15.3	14.6	0.034
atf6	558071	activating transcription factor 6	1.45E-03	3.77E-01	2.8	2.3	2.6	0.034
zgc:153606	558558	zgc:153606	9.04E-03	3.66E-01	14.7	16.7	15.7	0.034
si:dkey-42i9.10	558947	si:dkey-42i9.10	3.36E-01	3.68E-01	7.8	13.0	10.4	0.034
LOC559569	559569	egl nine homolog 2-like	2.88E-02	3.67E-01	20.0	0.0	22.0	0.034
kbtbd8	559966	kelch repeat and BTB (POZ) domain containing 8	3.83E-01	3.70E-01	18.2	17.0	17.6	0.034
trim8	559967	tripartite motif-containing 8	3.72E-01	1.08E-01	17.0	16.3	16.7	0.034
loxl1	560115	lysyl oxidase-like 1	3.87E-01	1.77E-01	18.7	19.5	19.1	0.034
zfp36l1a	561280	zinc finger protein 36, C3H type-like 1a	3.90E-01	1.38E-01	12.7	17.7	15.2	0.034
plcg2	561747	phospholipase C, gamma 2	3.60E-01	2.38E-01	21.5	17.3	19.4	0.034
si:dkey-174m14.3	563117	si:dkey-174m14.3	1.44E-01	3.54E-01	19.8	18.7	19.3	0.034
arhgap12a	563267	Rho GTPase activating protein 12a	1.67E-02	3.89E-01	2.3	1.7	2.0	0.034

Table S1: Zebrafish circadian oscillating genes

Gene Symbol	Gene ID	Gene Description	Fisher's g test p value (LD)	Fisher's g test p value (DD)	LD Phase	DD Phase	Phase *	FDR **
LOC563806	563806	lipid phosphate phosphohydrolase 2-like	1.99E-01	3.51E-01	2.2	1.2	1.7	0.034
cbln4	563847	cerebellin 4 precursor	2.95E-01	3.55E-01	17.7	19.0	18.4	0.034
sgsh	563849	N-sulfoglucosamine sulfohydrolase (sulfamidase)	3.72E-01	2.58E-01	14.0	17.7	15.9	0.034
setd4	564058	SET domain containing 4	3.81E-01	1.70E-01	20.5	23.3	21.9	0.034
pias2	564149	protein inhibitor of activated STAT, 2	3.84E-01	1.24E-01	14.7	15.8	15.3	0.034
zgc:153635	564210	zgc:153635	3.90E-01	3.55E-01	16.2	18.2	17.2	0.034
si:dkey-91f15.6	564304	si:dkey-91f15.6	3.66E-01	2.64E-02	6.8	6.4	6.6	0.034
ogdh	564552	oxoglutarate (alpha-ketoglutarate) dehydrogenase (lipoa	3.04E-01	3.56E-01	0.7	21.3	23.0	0.034
si:ch211-137i24.5	564882	si:ch211-137i24.5	3.65E-01	3.21E-02	3.3	4.5	3.9	0.034
adamts1	565145	ADAM metallopeptidase with thrombospondin type 1 mo	3.94E-01	3.49E-01	14.0	19.0	16.5	0.034
si:ch211-163l21.10	565282	si:ch211-163l21.10	1.52E-01	3.52E-01	14.5	15.5	15.0	0.034
si:dkey-221j11.2	565437	si:dkey-221j11.2	7.84E-02	3.57E-01	4.7	2.3	3.5	0.034
pde4b	565706	phosphodiesterase 4B, cAMP-specific	3.92E-01	1.14E-01	5.5	7.7	6.6	0.034
si:dkeyp-84g1.1	565760	si:dkeyp-84g1.1	3.58E-01	5.87E-02	3.2	3.8	3.5	0.034
LOC565849	565849	novel protein similar to vertebrate protein phosphatase 2	8.20E-02	3.56E-01	0.5	23.5	0.0	0.034
mfi2	565942	antigen p97 (melanoma associated) identified by monocl	3.79E-01	2.98E-01	7.3	8.3	7.8	0.034
mthfsd	566203	methenyltetrahydrofolate synthetase domain containing	9.16E-02	3.72E-01	3.8	4.3	4.1	0.034
dclre1c	566285	DNA cross-link repair 1C, PSO2 homolog (S. cerevisiae)	1.45E-03	3.50E-01	17.0	19.0	18.0	0.034
tmem106bb	566699	transmembrane protein 106Bb	4.00E-01	1.83E-01	15.8	17.3	16.6	0.034
si:busm1-189a20.4	566732	si:busm1-189a20.4	8.92E-03	3.51E-01	17.8	17.5	17.7	0.034
afap1l1	566757	actin filament associated protein 1-like 1	3.53E-01	1.79E-01	14.8	14.7	14.8	0.034
clvs2	566769	clavesin 2	4.50E-02	3.62E-01	20.3	17.5	18.9	0.034
LOC567031	567031	WD repeat-containing protein 47-like	3.81E-01	1.20E-01	16.8	17.5	17.2	0.034
scn4bb	567239	sodium channel, voltage-gated, type IV, beta b	3.52E-01	3.33E-01	3.8	2.0	2.9	0.034
akap12b	567365	A kinase (PRKA) anchor protein (gravin) 12b	3.67E-01	1.84E-01	16.8	16.7	16.8	0.034
si:ch211-155m12.3	567533	si:ch211-155m12.3	1.12E-02	3.96E-01	15.5	16.8	16.2	0.034
slc6a8	567544	solute carrier family 6 (neurotransmitter transporter, crea	7.19E-02	3.56E-01	0.3	22.5	23.4	0.034
si:dkey-193c22.1	567837	si:dkey-193c22.1	3.94E-02	3.75E-01	6.0	2.3	4.2	0.034
serac1	568022	serine active site containing 1	1.16E-01	3.82E-01	12.7	15.5	14.1	0.034
sdc4	568593	syndecan 4	1.58E-01	3.56E-01	15.8	17.7	16.8	0.034
si:dkey-110c1.7	568618	si:dkey-110c1.7	2.83E-02	3.86E-01	19.8	18.5	19.2	0.034

Table S1: Zebrafish circadian oscillating genes

Gene Symbol	Gene ID	Gene Description	Fisher's g test p value (LD)	Fisher's g test p value (DD)	LD Phase	DD Phase	Phase *	FDR **
LOC568719	568719	hypothetical LOC568719	1.95E-01	3.66E-01	20.5	20.5	20.5	0.034
LOC568809	568809	transmembrane protein 82-like	3.05E-03	3.84E-01	10.2	11.7	11.0	0.034
tprg1l	568867	tumor protein p63 regulated 1-like	2.62E-01	3.94E-01	19.2	15.0	17.1	0.034
zgc:136860	569049	zgc:136860	3.86E-01	2.28E-01	15.8	19.0	17.4	0.034
si:dkey-71l1.1	569883	si:dkey-71l1.1	3.31E-01	3.79E-01	23.5	21.2	22.4	0.034
wdr4	570351	WD repeat domain 4	3.90E-02	3.60E-01	22.0	0.2	23.1	0.034
si:ch211-146f4.3	570368	si:ch211-146f4.3	3.90E-01	2.08E-01	22.8	3.2	1.0	0.034
fgf4	570435	fibroblast growth factor 4	1.49E-01	3.74E-01	18.7	21.3	20.0	0.034
LOC570917	570917	actin-binding Rho-activating protein-like	3.84E-01	1.12E-01	15.5	20.8	18.2	0.034
si:dkey-20i10.6	571658	si:dkey-20i10.6	1.60E-02	3.77E-01	20.3	21.3	20.8	0.034
asz1	572911	ankyrin repeat, SAM and basic leucine zipper domain co	3.77E-01	2.34E-01	3.0	1.7	2.4	0.034
srp14	619260	signal recognition particle 14	3.77E-01	2.84E-02	1.8	2.8	2.3	0.034
as3mt	664699	arsenic (+3 oxidation state) methyltransferase	3.63E-01	1.62E-01	18.3	19.7	19.0	0.034
zgc:136909	678609	zgc:136909	1.69E-01	3.76E-01	1.2	3.5	2.4	0.034
zgc:136902	678611	zgc:136902	1.61E-02	3.82E-01	4.3	6.0	5.2	0.034
stmn1a	678630	stathmin 1a	3.13E-01	3.83E-01	6.8	6.8	6.8	0.034
zgc:136560	692331	zgc:136560	2.72E-01	3.99E-01	14.7	17.7	16.2	0.034
setd8a	751629	SET domain containing (lysine methyltransferase) 8a	3.67E-01	7.58E-02	1.3	2.8	2.1	0.034
zgc:153151	751646	zgc:153151	3.03E-03	3.74E-01	14.8	17.3	16.1	0.034
zgc:153989	767796	zgc:153989	9.65E-04	3.85E-01	0.7	0.8	0.8	0.034
zgc:152997	768176	zgc:152997	4.28E-02	3.54E-01	15.7	17.5	16.6	0.034
cyp2x12	790965	cytochrome P450 CYP2X12	8.81E-02	3.92E-01	10.8	10.2	10.5	0.034
LOC793364	793364	FYVE, RhoGEF and PH domain-containing protein 4-like	3.57E-01	2.12E-01	14.7	17.3	16.0	0.034
hip1	793823	huntingtin interacting protein 1	3.31E-01	3.84E-01	12.7	15.0	13.9	0.034
bco2b	793849	beta-carotene oxygenase 2b	4.00E-01	2.89E-03	11.7	17.0	14.4	0.034
si:dkey-17e16.10	793878	si:dkey-17e16.10	2.42E-02	3.66E-01	0.0	23.2	23.6	0.034
notch1b	794892	notch homolog 1b	3.80E-01	1.28E-01	4.0	6.0	5.0	0.034
cetn2	795310	centrin, EF-hand protein, 2	1.42E-01	3.82E-01	13.5	16.7	15.1	0.034
mrps34	795846	mitochondrial ribosomal protein S34	3.00E-01	3.77E-01	0.3	1.2	0.8	0.034
si:dkeyp-110e4.11	797016	si:dkeyp-110e4.11	3.84E-01	6.79E-02	13.3	17.2	15.3	0.034
si:ch211-197k23.4	797201	si:ch211-197k23.4	3.79E-01	3.63E-01	10.7	13.5	12.1	0.034

Table S1: Zebrafish circadian oscillating genes

Gene Symbol	Gene ID	Gene Description	Fisher's g test p value (LD)	Fisher's g test p value (DD)	LD Phase	DD Phase	Phase *	FDR **
tada2a	797303	transcriptional adaptor 2A	1.02E-02	3.64E-01	12.8	15.3	14.1	0.034
LOC797682	797682	protein FAM69A-like	3.50E-01	3.46E-01	20.2	22.0	21.1	0.034
apln	798375	apelin	2.40E-01	3.60E-01	15.8	16.0	15.9	0.034
zgc:194392	798626	zgc:194392	6.71E-03	3.59E-01	4.7	2.7	3.7	0.034
abca4a	798993	ATP-binding cassette, sub-family A (ABC1), member 4a	5.34E-05	3.83E-01	6.0	6.5	6.3	0.034
ptgesl	799964	prostaglandin E synthase 2-like	3.31E-02	3.52E-01	4.7	0.7	2.7	0.034
nrip1b	100001853	nuclear receptor interacting protein 1b	5.27E-02	3.73E-01	11.3	13.5	12.4	0.034
wu:fa99e11	100002536	wu:fa99e11	3.45E-01	3.65E-01	15.2	15.5	15.4	0.034
ppp1cb	100003223	protein phosphatase 1, catalytic subunit, beta isoform	3.43E-02	3.56E-01	15.5	18.2	16.9	0.034
LOC100004428	100004428	vasorin like	3.50E-02	3.77E-01	17.3	19.2	18.3	0.034
tmem174	100005615	transmembrane protein 174	1.10E-01	3.75E-01	1.2	2.3	1.8	0.034
cited3	100006037	Cbp/p300-interacting transactivator, with Glu/Asp-rich ca	3.97E-01	1.86E-01	10.5	14.7	12.6	0.034
arl16	100037345	ADP-ribosylation factor-like 16	3.70E-01	3.74E-01	15.2	15.8	15.5	0.034
zgc:162420	100037388	zgc:162420	6.59E-02	4.00E-01	14.4	16.3	15.3	0.034
LOC100148657	100148657	neurotactin-like	3.92E-01	3.51E-01	10.8	16.5	13.7	0.034
LOC100149258	100149258	hypothetical protein LOC100149258	2.98E-01	3.92E-01	13.5	12.2	12.9	0.034
LOC100150431	100150431	leucine-rich repeat neuronal protein 2-like	1.39E-01	3.95E-01	17.2	17.5	17.4	0.034
LOC100151157	100151157	similar to bin3, bicoid-interacting 3	7.30E-02	3.98E-01	18.2	17.7	18.0	0.034
serp2	100319240	stress-associated endoplasmic reticulum protein family n	1.56E-01	3.69E-01	14.8	15.3	15.1	0.034
LOC100329742	100329742	zinc finger protein 653-like	5.70E-02	3.84E-01	15.2	17.3	16.3	0.034
LOC100330648	100330648	myosin-Ib-like	3.62E-01	2.09E-02	3.5	5.5	4.5	0.034
LOC100331800	100331800	photoreceptor outer segment membrane glycoprotein 2-l	2.21E-02	3.94E-01	17.5	19.0	18.3	0.034
LOC100332541	100332541	hypothetical protein LOC100332541	3.82E-01	1.42E-01	23.7	23.0	23.4	0.034
LOC100334140	100334140	hypothetical protein LOC100334140	3.83E-01	3.28E-01	16.3	18.8	17.6	0.034
ptch2	30181	patched 2	4.86E-02	4.07E-01	20.0	18.3	19.2	0.041
copg2	30187	coatomer protein complex, subunit gamma 2	2.67E-01	4.35E-01	21.3	22.0	21.7	0.041
efnb2a	30219	ephrin B2a	2.08E-02	4.35E-01	17.0	16.0	16.5	0.041
pax3a	30532	paired box gene 3a	4.13E-01	3.70E-02	10.7	20.0	15.4	0.041
raf1a	30716	v-raf-1 murine leukemia viral oncogene homolog 1a	3.52E-01	4.04E-01	14.7	17.3	16.0	0.041
chrna1	30725	cholinergic receptor, nicotinic, alpha polypeptide 1	4.37E-01	2.02E-02	11.7	18.5	15.1	0.041
ngf	58133	nerve growth factor (beta polypeptide)	1.18E-01	4.10E-01	15.0	18.5	16.8	0.041

Table S1: Zebrafish circadian oscillating genes

Gene Symbol	Gene ID	Gene Description	Fisher's g test p value (LD)	Fisher's g test p value (DD)	LD Phase	DD Phase	Phase *	FDR **
zp2.3	114439	zona pellucida glycoprotein 2.3	4.48E-01	1.17E-01	2.2	1.7	2.0	0.041
atic	140622	5-aminoimidazole-4-carboxamide ribonucleotide formyltr	4.08E-01	4.72E-02	3.2	4.8	4.0	0.041
ahr1a	246224	aryl hydrocarbon receptor 1a	3.00E-01	4.04E-01	2.7	3.0	2.9	0.041
caspb	259303	caspase b	4.17E-01	2.94E-01	3.8	6.0	4.9	0.041
ruvbl1	317679	RuvB-like 1 (E. coli)	4.04E-01	2.34E-01	0.3	4.0	2.2	0.041
zgc:73187	321203	zgc:73187	4.28E-01	1.70E-01	15.3	19.7	17.5	0.041
tomm22	321232	translocase of outer mitochondrial membrane 22 homolo	1.25E-01	4.47E-01	22.7	1.5	0.1	0.041
smyd1a	321245	SET and MYND domain containing 1a	1.32E-01	4.41E-01	16.4	18.1	17.2	0.041
ift88	321855	intraflagellar transport 88 homolog	5.12E-02	4.38E-01	18.3	19.2	18.8	0.041
eif1b	322237	eukaryotic translation initiation factor 1B	9.20E-02	4.24E-01	16.3	17.0	16.7	0.041
zgc:77126	322780	zgc:77126	1.90E-01	4.33E-01	1.3	2.7	2.0	0.041
mtmr14	323929	myotubularin related protein 14	1.36E-01	4.09E-01	14.3	15.5	14.9	0.041
wu:fc18h07	324108	wu:fc18h07	5.68E-02	4.12E-01	11.7	14.0	12.9	0.041
tmem165	324501	transmembrane protein 165	1.16E-02	4.03E-01	15.2	16.8	16.0	0.041
gpr56	324948	G protein-coupled receptor 56	2.55E-01	4.29E-01	15.0	17.0	16.0	0.041
pars2	325494	prolyl-tRNA synthetase (mitochondrial)	1.30E-02	4.19E-01	20.8	23.3	22.1	0.041
zmynd19	325735	zinc finger, MYND domain containing 19	1.81E-01	4.37E-01	23.7	1.0	0.4	0.041
plxna4	325938	plexin A4	1.75E-01	4.42E-01	22.2	17.3	19.8	0.041
slc14a2	326634	solute carrier family 14 (urea transporter), member 2	6.09E-02	4.32E-01	6.8	8.0	7.4	0.041
tlk1b	326734	tousled-like kinase 1b	4.15E-01	4.41E-01	15.5	16.3	15.9	0.041
psmc4	326884	proteasome (prosome, macropain) 26S subunit, ATPase	7.40E-02	4.02E-01	17.5	19.0	18.3	0.041
fam3c	334205	family with sequence similarity 3, member C	7.80E-02	4.29E-01	2.5	2.0	2.3	0.041
si:dkey-18f23.10	334465	si:dkey-18f23.10	2.34E-02	4.05E-01	19.0	16.3	17.7	0.041
mdh1b	335715	malate dehydrogenase 1b, NAD (soluble)	4.14E-01	2.08E-01	3.0	3.4	3.2	0.041
sema6a	336060	sema domain, transmembrane domain (TM), and cytopla	1.75E-01	4.48E-01	18.3	17.5	17.9	0.041
optn	336159	optineurin	3.96E-01	4.47E-01	12.8	16.2	14.5	0.041
cdr2l	336227	cerebellar degeneration-related protein 2-like	2.44E-02	4.47E-01	18.3	17.3	17.8	0.041
sdf2	336879	stromal cell-derived factor 2	2.22E-02	4.33E-01	1.8	1.9	1.8	0.041
tbl2	337068	transducin (beta)-like 2	1.70E-01	4.22E-01	23.5	23.8	23.7	0.041
zgc:85910	337459	zgc:85910	1.34E-01	4.34E-01	1.2	0.2	0.7	0.041
wu:fj87a11	337614	wu:fj87a11	1.23E-02	4.17E-01	15.2	17.2	16.2	0.041

Table S1: Zebrafish circadian oscillating genes

Gene Symbol	Gene ID	Gene Description	Fisher's g test p value (LD)	Fisher's g test p value (DD)	LD Phase	DD Phase	Phase *	FDR **
timp2a	359835	tissue inhibitor of metalloproteinase 2a	1.20E-02	4.11E-01	16.5	17.2	16.9	0.041
tbx1	368206	T-box 1	3.79E-01	4.48E-01	14.3	17.2	15.8	0.041
adka	368220	adenosine kinase a	4.26E-01	4.07E-01	2.8	3.5	3.2	0.041
yars	368235	tyrosyl-tRNA synthetase	5.10E-02	4.36E-01	22.7	0.8	23.8	0.041
xiap	373108	X-linked inhibitor of apoptosis	7.16E-02	4.08E-01	22.7	1.2	24.0	0.041
cdc14b	393148	CDC14 cell division cycle 14 homolog B	4.05E-01	5.74E-02	14.0	13.5	13.8	0.041
fzr1	393223	fizzy/cell division cycle 20 related 1 (Drosophila)	4.19E-01	1.18E-01	12.8	14.7	13.8	0.041
slc25a32a	393226	solute carrier family 25, member 32a	3.00E-01	4.44E-01	12.7	14.8	13.8	0.041
zgc:56706	393244	zgc:56706	4.18E-01	4.10E-01	4.2	4.5	4.4	0.041
zgc:56676	393273	zgc:56676	1.46E-01	4.08E-01	0.5	23.5	0.0	0.041
chuk	393287	conserved helix-loop-helix ubiquitous kinase	7.06E-02	4.27E-01	13.7	15.3	14.5	0.041
cog6	393290	component of oligomeric golgi complex 6	6.60E-02	4.08E-01	22.7	19.3	21.0	0.041
zgc:56585	393297	zgc:56585	4.34E-01	1.88E-01	2.8	3.0	2.9	0.041
ero1l	393321	ERO1-like (S. cerevisiae)	4.25E-01	1.99E-01	0.3	23.7	0.0	0.041
ncs1a	393437	neuronal calcium sensor 1a	4.12E-01	7.86E-02	11.3	11.3	11.3	0.041
zgc:65780	393506	zgc:65780	4.15E-01	3.86E-01	3.7	4.8	4.3	0.041
mtap	393526	methylthioadenosine phosphorylase	1.16E-01	4.38E-01	1.8	4.0	2.9	0.041
tsc22d3	393541	TSC22 domain family, member 3	4.62E-03	4.23E-01	7.8	9.3	8.6	0.041
skp1	393716	S-phase kinase-associated protein 1	2.48E-01	4.12E-01	22.8	19.8	21.3	0.041
panx1	393890	pannexin 1	1.52E-01	4.44E-01	18.8	20.5	19.7	0.041
cdc5l	394059	CDC5 cell division cycle 5-like (S. pombe)	4.41E-01	2.26E-01	19.7	18.7	19.2	0.041
zgc:56376	394070	zgc:56376	4.27E-01	1.38E-01	11.6	15.4	13.5	0.041
kdsr	394114	3-ketodihydrosphingosine reductase	2.15E-01	4.47E-01	4.7	2.3	3.5	0.041
ubxn7	402913	UBX domain protein 7	4.43E-01	4.36E-01	14.3	16.7	15.5	0.041
zswim6	403057	zinc finger, SWIM-type containing 6	4.12E-01	1.97E-01	22.5	19.0	20.8	0.041
araf	403063	v-raf murine sarcoma 3611 viral oncogene homolog	2.24E-01	4.39E-01	15.7	17.5	16.6	0.041
dnajc2	403080	DnaJ (Hsp40) homolog, subfamily C, member 2	4.42E-02	4.43E-01	22.3	23.8	23.1	0.041
slc13a3	405838	solute carrier family 13 (sodium-dependent dicarboxylate	4.19E-01	4.39E-01	1.2	2.3	1.8	0.041
prkag1	406440	protein kinase, AMP-activated, gamma 1 non-catalytic su	4.01E-01	3.90E-01	16.5	17.5	17.0	0.041
orc5	406479	origin recognition complex, subunit 5	4.10E-01	1.54E-01	3.0	4.8	3.9	0.041
rftn2	406729	raftlin family member 2	3.60E-01	4.31E-01	14.4	15.4	14.9	0.041

Table S1: Zebrafish circadian oscillating genes

Gene Symbol	Gene ID	Gene Description	Fisher's g test p value (LD)	Fisher's g test p value (DD)	LD Phase	DD Phase	Phase *	FDR **
tax1bp3	406779	Tax1 (human T-cell leukemia virus type I) binding proteir	7.80E-02	4.42E-01	5.3	4.0	4.7	0.041
skiv2l2	406795	superkiller viralicidic activity 2-like 2	4.70E-02	4.06E-01	23.3	2.2	0.8	0.041
LOC407656	407656	hypothetical protein LOC407656	7.40E-02	4.02E-01	9.8	15.5	12.7	0.041
hprt1l	415146	hypoxanthine phosphoribosyltransferase 1, like	2.48E-02	4.04E-01	1.3	2.8	2.1	0.041
tjp2b	436639	tight junction protein 2b (zona occludens 2)	6.67E-02	4.17E-01	14.3	15.2	14.8	0.041
ctsl.1	436641	cathepsin L.1	4.10E-01	6.13E-02	12.7	11.8	12.3	0.041
zgc:92061	436656	zgc:92061	6.58E-02	4.20E-01	10.8	13.8	12.3	0.041
psmb2	436882	proteasome (prosome, macropain) subunit, beta type, 2	4.11E-01	8.32E-02	20.5	21.7	21.1	0.041
gins3	442931	GINS complex subunit 3	3.64E-01	4.49E-01	5.0	6.0	5.5	0.041
pdcd7	445020	programmed cell death 7	1.43E-01	4.23E-01	15.5	11.0	13.3	0.041
zgc:92380	445086	zgc:92380	1.53E-01	4.18E-01	16.5	19.0	17.8	0.041
g6pca	445118	glucose-6-phosphatase a, catalytic	4.34E-01	1.75E-02	5.5	4.3	4.9	0.041
hspbap1	445320	hspb associated protein 1	2.11E-01	4.11E-01	13.2	16.0	14.6	0.041
ddx51	445387	DEAD (Asp-Glu-Ala-Asp) box polypeptide 51	4.01E-01	2.71E-01	22.7	0.5	23.6	0.041
s100s	445481	S100 calcium binding protein S	2.04E-01	4.46E-01	14.5	19.3	16.9	0.041
morc2	445488	MORC family CW-type zinc finger 2	6.72E-02	4.13E-01	23.8	23.2	23.5	0.041
zgc:92111	447807	zgc:92111	4.34E-01	3.28E-01	21.7	1.2	23.5	0.041
ccdc47	447812	coiled-coil domain containing 47	2.08E-01	4.21E-01	21.3	23.8	22.6	0.041
gbgt1l3	447881	globoside alpha-1,3-N-acetylgalactosaminyltransferase 1	1.32E-01	4.24E-01	22.3	0.7	23.5	0.041
hint1	449551	histidine triad nucleotide binding protein 1	4.11E-01	7.46E-02	3.2	5.2	4.2	0.041
pex10	449821	peroxisome biogenesis factor 10	4.44E-01	3.41E-01	3.0	22.8	0.9	0.041
zgc:101765	450036	zgc:101765	1.77E-01	4.10E-01	6.0	6.8	6.4	0.041
gcga	494052	glucagon a	4.16E-01	1.69E-01	23.7	22.7	23.2	0.041
rab25	494098	RAB25, member RAS oncogene family	3.14E-02	4.47E-01	1.8	3.0	2.4	0.041
prkar1aa	494533	protein kinase, cAMP-dependent, regulatory, type I, alph	2.36E-01	4.01E-01	14.5	16.2	15.3	0.041
mecom	497407	MDS1 and EVI1 complex locus	1.69E-01	4.23E-01	15.7	9.5	12.6	0.041
numbl	497616	numb homolog (Drosophila)-like	4.08E-01	2.95E-01	11.8	12.3	12.1	0.041
scube2	503728	signal peptide, CUB domain, EGF-like 2	1.43E-01	4.17E-01	14.8	16.2	15.5	0.041
zgc:113114	503732	zgc:113114	9.25E-02	4.44E-01	7.0	7.2	7.1	0.041
zgc:113263	503753	zgc:113263	2.16E-01	4.07E-01	14.5	16.0	15.3	0.041
zgc:113036	503762	zgc:113036	2.26E-02	4.33E-01	23.3	2.3	0.8	0.041

Table S1: Zebrafish circadian oscillating genes

Gene Symbol	Gene ID	Gene Description	Fisher's g test p value (LD)	Fisher's g test p value (DD)	LD Phase	DD Phase	Phase *	FDR **
phf23a	541372	PHD finger protein 23a	8.77E-02	4.49E-01	21.5	22.7	22.1	0.041
eif4enif1	541411	eukaryotic translation initiation factor 4E nuclear import fa	4.20E-01	2.65E-01	14.7	12.0	13.4	0.041
zgc:112015	550376	zgc:112015	1.88E-01	4.35E-01	2.0	5.8	3.9	0.041
baiap2l1b	550528	BAI1-associated protein 2-like 1b	4.43E-01	3.97E-01	14.5	12.8	13.7	0.041
LOC553259	553259	hypothetical protein LOC553259	4.24E-01	8.74E-02	12.2	23.3	17.7	0.041
setdb1a	553292	SET domain, bifurcated 1a	4.27E-01	2.01E-01	2.2	6.2	4.2	0.041
ngly1	553627	N-glycanase 1	4.12E-01	4.17E-01	16.8	19.5	18.2	0.041
zgc:112367	554110	zgc:112367	4.12E-01	3.14E-01	3.7	5.5	4.6	0.041
asb1	554673	ankyrin repeat and SOCS box-containing 1	1.60E-01	4.45E-01	10.5	14.8	12.7	0.041
gpr157	555274	G protein-coupled receptor 157	1.14E-01	4.08E-01	14.7	17.7	16.2	0.041
taf1	555452	TAF1 RNA polymerase II, TATA box binding protein (TBI	4.39E-01	2.88E-01	15.3	17.1	16.2	0.041
pcdh19	555688	protocadherin 19	8.73E-02	4.30E-01	18.2	17.3	17.8	0.041
wbscr16	555972	Williams-Beuren syndrome chromosome region 16 homo	3.97E-01	4.11E-01	0.0	1.7	0.9	0.041
tmem69	557512	transmembrane protein 69	4.64E-02	4.28E-01	1.8	2.2	2.0	0.041
LOC557524	557524	serine/threonine-protein kinase SBK1-like	2.49E-01	4.18E-01	0.3	23.3	23.8	0.041
zgc:153031	557552	zgc:153031	4.45E-01	5.05E-02	19.3	22.7	21.0	0.041
nt5c2b	557972	5'-nucleotidase, cytosolic IIb	3.08E-02	4.46E-01	18.0	18.3	18.2	0.041
si:dkey-19f21.3	557996	si:dkey-19f21.3	1.02E-01	4.35E-01	15.2	17.2	16.2	0.041
si:ch211-86h15.1	558435	si:ch211-86h15.1	2.45E-01	4.14E-01	12.5	16.0	14.3	0.041
selo	558648	selenoprotein O	4.31E-02	4.28E-01	5.5	5.7	5.6	0.041
cacnb2b	558806	calcium channel, voltage-dependent, beta 2b	1.79E-01	4.33E-01	17.8	12.8	15.3	0.041
zgc:165467	558833	zgc:165467	4.46E-01	3.34E-02	16.7	17.3	17.0	0.041
LOC558930	558930	UDP-GlcNAc:betaGal beta-1,3-N-acetylglucosaminyltran	4.21E-01	3.50E-01	9.0	10.3	9.7	0.041
lingo4b	559074	leucine rich repeat and Ig domain containing 4b	4.49E-03	4.28E-01	15.2	15.3	15.3	0.041
LOC559239	559239	hypothetical LOC559239	4.14E-02	4.20E-01	12.7	14.8	13.7	0.041
LOC559281	559281	insulin receptor substrate 1-B-like	3.54E-01	4.00E-01	6.3	7.0	6.7	0.041
syne2b	559348	spectrin repeat containing, nuclear envelope 2b	5.52E-04	4.38E-01	15.2	14.5	14.9	0.041
si:dkey-246j7.1	559429	si:dkey-246j7.1	1.01E-01	4.34E-01	5.3	3.3	4.3	0.041
LOC559643	559643	novel protein similar to vertebrate erythrocyte membrane	1.83E-01	4.33E-01	15.8	12.0	13.9	0.041
si:dkeyp-11g8.6	559657	si:dkeyp-11g8.6	1.74E-01	4.38E-01	5.2	6.7	6.0	0.041
illr4	559737	immune-related, lectin-like receptor 4	1.81E-01	4.42E-01	12.0	18.2	15.1	0.041

Table S1: Zebrafish circadian oscillating genes

Gene Symbol	Gene ID	Gene Description	Fisher's g test p value (LD)	Fisher's g test p value (DD)	LD Phase	DD Phase	Phase *	FDR **
armc3	560136	armadillo repeat containing 3	1.94E-01	4.12E-01	15.3	16.0	15.7	0.041
atpaf2	560253	ATP synthase mitochondrial F1 complex assembly factor	1.29E-02	4.39E-01	0.2	1.8	1.0	0.041
LOC560304	560304	sushi domain-containing protein 4-like	4.19E-01	9.62E-02	17.9	17.8	17.8	0.041
si:dkey-1h24.3	560319	si:dkey-1h24.3	4.27E-01	4.32E-01	23.5	1.0	0.3	0.041
cspg4	560393	chondroitin sulfate proteoglycan 4	1.07E-02	4.11E-01	4.2	4.8	4.5	0.041
gfer	560433	growth factor, augmenter of liver regeneration (ERV1 hor	4.41E-01	4.01E-01	23.8	1.3	0.6	0.041
eif3eb	561049	eukaryotic translation initiation factor 3, subunit E, b	7.66E-02	4.35E-01	16.3	18.2	17.3	0.041
si:dkey-149j18.2	561142	si:dkey-149j18.2	1.93E-01	4.30E-01	23.7	2.2	1.0	0.041
zgc:153898	561339	zgc:153898	2.34E-01	4.46E-01	23.7	20.3	22.0	0.041
wu:fc76b11	561802	wu:fc76b11	6.68E-02	4.31E-01	12.7	12.7	12.7	0.041
snx18a	562372	sorting nexin 18a	1.01E-01	4.47E-01	4.0	3.7	3.9	0.041
si:ch211-234g24.1	562516	si:ch211-234g24.1	4.06E-01	9.04E-02	14.7	15.8	15.3	0.041
ahi1	562701	Abelson helper integration site 1	1.55E-01	4.33E-01	10.5	11.7	11.1	0.041
a1cf	562916	apobec1 complementation factor	9.61E-02	4.32E-01	23.0	23.8	23.4	0.041
zgc:163107	563454	zgc:163107	3.05E-01	4.35E-01	17.8	19.5	18.7	0.041
ttll6	564075	tubulin tyrosine ligase-like family, member 6	3.35E-02	4.47E-01	13.5	14.5	14.0	0.041
si:ch211-51n14.1	564336	si:ch211-51n14.1	1.46E-01	4.45E-01	3.2	4.2	3.7	0.041
si:ch211-145m1.1	564423	si:ch211-145m1.1	2.84E-01	4.26E-01	1.9	1.3	1.6	0.041
rassf7a	564485	Ras association (RalGDS/AF-6) domain family (N-termin	9.78E-02	4.17E-01	15.2	15.0	15.1	0.041
tmem198b	564608	transmembrane protein 198b	2.90E-03	4.29E-01	16.0	16.2	16.1	0.041
si:ch211-153j24.6	564794	si:ch211-153j24.6	4.25E-01	3.29E-01	10.0	14.2	12.1	0.041
akap9	565037	A kinase (PRKA) anchor protein (yotiao) 9	4.08E-01	1.00E-01	17.7	17.7	17.7	0.041
dgat2	565316	diacylglycerol O-acyltransferase 2	1.37E-01	4.14E-01	8.4	10.2	9.3	0.041
si:ch211-11c15.3	566329	si:ch211-11c15.3	4.17E-01	1.53E-01	5.7	5.8	5.8	0.041
uqcrh	566723	ubiquinol-cytochrome c reductase hinge protein	4.16E-01	1.19E-01	23.8	3.0	1.4	0.041
mical3b	566850	molecule interacting with CasL 3b	1.43E-02	4.48E-01	14.0	14.9	14.4	0.041
mia3	567041	melanoma inhibitory activity family, member 3	4.18E-01	1.71E-01	18.0	16.7	17.4	0.041
zgc:162576	567100	zgc:162576	4.16E-02	4.31E-01	14.2	15.7	15.0	0.041
si:ch211-241o7.4	567267	si:ch211-241o7.4	2.23E-02	4.06E-01	17.2	16.8	17.0	0.041
nlgn3b	567415	neuroligin 3b	3.34E-01	4.02E-01	22.2	20.2	21.2	0.041
zgc:123177	567680	zgc:123177	2.67E-01	4.15E-01	12.8	16.0	14.4	0.041

Table S1: Zebrafish circadian oscillating genes

Gene Symbol	Gene ID	Gene Description	Fisher's g test p value (LD)	Fisher's g test p value (DD)	LD Phase	DD Phase	Phase *	FDR **
mll3b	568162	myeloid/lymphoid or mixed-lineage leukemia 3b	4.20E-01	3.36E-01	22.0	16.3	19.2	0.041
lrrn1	568527	leucine rich repeat neuronal 1	2.71E-01	4.12E-01	16.8	16.2	16.5	0.041
LOC568811	568811	similar to Protein kinase C and casein kinase substrate i	1.26E-01	4.18E-01	14.3	14.0	14.2	0.041
reep2	568927	receptor accessory protein 2	1.46E-02	4.21E-01	2.8	3.2	3.0	0.041
im:7147183	569546	im:7147183	3.37E-03	4.12E-01	12.6	12.5	12.6	0.041
tox	569666	thymocyte selection-associated high mobility group box	1.61E-02	4.36E-01	19.8	16.2	18.0	0.041
slc24a5	570312	solute carrier family 24, member 5	1.16E-03	4.23E-01	23.1	11.8	17.4	0.041
bbs12	570583	Bardet-Biedl syndrome 12	2.97E-01	4.40E-01	13.3	15.8	14.6	0.041
moxd1	570584	monooxygenase, DBH-like 1	2.90E-01	4.38E-01	8.7	21.2	3.0	0.041
epha8	570677	eph receptor A8	3.02E-01	4.25E-01	21.0	16.7	18.9	0.041
cyp2aa1	572000	cytochrome P450, family 2, subfamily AA, polypeptide 1	3.62E-01	4.14E-01	15.3	15.0	15.2	0.041
tnnt2d	574000	troponin T2d, cardiac	4.89E-02	4.22E-01	13.8	17.8	15.8	0.041
reep3l	641577	receptor accessory protein 3, like	4.38E-01	8.09E-02	9.5	9.8	9.7	0.041
trim55b	664753	tripartite motif-containing 55b	3.55E-01	4.07E-01	13.8	16.2	15.0	0.041
tbce	664760	tubulin folding cofactor E	1.13E-01	4.46E-01	15.5	17.3	16.4	0.041
cirbp	678563	cold inducible RNA binding protein	2.16E-02	4.14E-01	16.2	16.8	16.5	0.041
dab1b	678641	disabled homolog 1b (Drosophila)	2.80E-01	4.06E-01	14.0	16.7	15.4	0.041
pde12	692319	phosphodiesterase 12	6.79E-02	4.43E-01	23.7	1.3	0.5	0.041
zgc:153763	751717	zgc:153763	4.46E-01	1.34E-02	2.2	2.5	2.4	0.041
abhd12	767657	abhydrolase domain containing 12	1.27E-02	4.22E-01	4.7	3.0	3.9	0.041
zgc:154040	767688	zgc:154040	4.00E-01	1.67E-01	23.3	23.3	23.3	0.041
zgc:153723	767718	zgc:153723	4.12E-01	4.44E-01	2.5	23.3	0.9	0.041
klhl24	768198	kelch-like 24 (Drosophila)	4.09E-01	7.63E-02	15.3	18.7	17.0	0.041
fbxo11	777607	F-box protein 11	5.19E-02	4.43E-01	20.8	16.7	18.8	0.041
zfyve28	777615	zinc finger, FYVE domain containing 28	4.41E-01	1.37E-01	21.2	19.3	20.3	0.041
zgc:153012	777626	zgc:153012	4.17E-01	1.98E-01	14.8	16.7	15.8	0.041
crfb7	777651	cytokine receptor family member b7	9.45E-02	4.17E-01	13.2	16.8	15.0	0.041
fam46ba	777768	family with sequence similarity 46, member Ba	4.21E-01	4.87E-02	8.2	8.5	8.4	0.041
ldlrap1b	791153	low density lipoprotein receptor adaptor protein 1b	2.76E-01	4.12E-01	0.7	21.3	23.0	0.041
zgc:158689	791177	zgc:158689	3.32E-01	4.31E-01	12.3	16.7	14.5	0.041
sepp1b	791479	selenoprotein P, plasma, 1b	4.38E-01	1.92E-01	5.5	6.7	6.1	0.041

Table S1: Zebrafish circadian oscillating genes

Gene Symbol	Gene ID	Gene Description	Fisher's g test p value (LD)	Fisher's g test p value (DD)	LD Phase	DD Phase	Phase *	FDR **
galk2	792268	galactokinase 2	1.75E-01	4.36E-01	13.7	12.3	13.0	0.041
wu:fd14a06	794433	wu:fd14a06	1.13E-01	4.44E-01	23.0	22.5	22.8	0.041
zgc:163143	795946	zgc:163143	2.41E-02	4.46E-01	14.2	15.8	15.0	0.041
dnajc6	796345	DnaJ (Hsp40) homolog, subfamily C, member 6	2.72E-01	4.39E-01	0.5	20.5	22.5	0.041
si:dkey-286j15.1	796378	si:dkey-286j15.1	4.43E-01	7.73E-02	16.2	18.3	17.3	0.041
LOC797085	797085	zinc metalloproteinase nas-15-like	4.09E-01	1.06E-01	5.3	7.2	6.3	0.041
LOC798075	798075	fascin 2A	1.75E-01	4.25E-01	0.8	1.3	1.1	0.041
LOC799022	799022	galactosidase, beta 1-like	1.06E-01	4.46E-01	12.5	8.7	10.6	0.041
oip5	799087	opa interacting protein 5	1.27E-01	4.47E-01	11.8	11.2	11.5	0.041
ppp1r3da	799144	protein phosphatase 1, regulatory (inhibitor) subunit 3D a	3.24E-03	4.13E-01	11.3	14.7	13.0	0.041
gbp4	100001368	guanylate binding protein 4	8.36E-02	4.33E-01	2.8	3.2	3.0	0.041
LOC100002786	100002786	probable G-protein coupled receptor 83-like	7.13E-02	4.08E-01	23.3	18.8	21.1	0.041
zgc:162701	100003383	zgc:162701	4.49E-01	4.48E-01	21.8	2.5	0.2	0.041
zgc:162898	100003490	zgc:162898	5.43E-02	4.08E-01	17.3	18.3	17.8	0.041
zeb2a	100003889	zinc finger E-box binding homeobox 2a	1.39E-01	4.42E-01	19.0	16.2	17.6	0.041
LOC100004307	100004307	hypothetical protein LOC100004307	4.47E-01	2.45E-01	3.7	4.3	4.0	0.041
phyhiplb	100004913	phytanoyl-CoA 2-hydroxylase interacting protein-like b	9.26E-05	4.02E-01	3.5	2.5	3.0	0.041
LOC100004941	100004941	grpE protein homolog 2, mitochondrial-like	4.94E-03	4.33E-01	15.7	17.8	16.8	0.041
LOC100007598	100007598	relaxin-3 receptor 1-like	4.09E-01	3.43E-01	12.5	15.2	13.9	0.041
si:dkey-13i19.7	100034649	si:dkey-13i19.7	4.01E-01	2.95E-01	1.0	22.3	23.7	0.041
zgc:162999	100038772	zgc:162999	2.30E-02	4.22E-01	14.8	13.0	13.9	0.041
si:ch211-284o19.6	100144567	si:ch211-284o19.6	3.14E-01	4.38E-01	15.2	17.8	16.5	0.041
LOC100148274	100148274	hypothetical protein LOC100148274	4.27E-01	3.23E-01	21.7	18.5	20.1	0.041
LOC100148281	100148281	novel protein similar to H.sapiens WDR89, WD repeat dc	4.28E-01	4.10E-01	23.5	2.5	1.0	0.041
LOC100148910	100148910	hypothetical protein LOC100148910	2.63E-01	4.09E-01	14.2	15.8	15.0	0.041
LOC100151174	100151174	heparan sulfate 2-O-sulfotransferase 1-like	1.18E-01	4.29E-01	16.2	14.3	15.3	0.041
si:dkeyp-115d2.5	100320541	si:dkeyp-115d2.5	4.42E-01	5.56E-03	21.5	15.8	18.7	0.041
si:dkey-256e7.8	100321607	si:dkey-256e7.8	6.40E-02	4.08E-01	10.7	12.2	11.5	0.041
LOC100332407	100332407	hypothetical protein LOC100332407	4.36E-01	1.15E-01	7.2	12.7	10.0	0.041
LOC100333859	100333859	hypothetical protein LOC100333859	4.07E-01	1.08E-01	2.3	2.5	2.4	0.041
LOC100334535	100334535	ubl carboxyl-terminal hydrolase 18-like	3.43E-01	4.01E-01	16.5	18.3	17.4	0.041

Table S1: Zebrafish circadian oscillating genes

Gene Symbol	Gene ID	Gene Description	Fisher's g test p value (LD)	Fisher's g test p value (DD)	LD Phase	DD Phase	Phase *	FDR **
hoxb2a	30338	homeo box B2a	1.26E-01	4.79E-01	16.5	15.8	16.2	0.049
hoxb1b	30374	homeo box B1b	4.30E-02	4.87E-01	16.3	16.7	16.5	0.049
nr2f5	30427	nuclear receptor subfamily 2, group F, member 5	3.06E-01	4.94E-01	17.2	20.0	18.6	0.049
tmod4	30441	tropomodulin 4 (muscle)	4.81E-01	3.70E-01	4.2	5.0	4.6	0.049
smad1	30628	MAD homolog 1 (Drosophila)	1.84E-02	4.83E-01	19.8	20.8	20.3	0.049
bmp2b	30632	bone morphogenetic protein 2b	2.58E-01	4.83E-01	16.3	19.0	17.7	0.049
psen2	58026	presenilin 2	3.58E-02	4.84E-01	15.8	16.8	16.3	0.049
hoxa4a	58050	homeo box A4a	8.07E-02	4.91E-01	16.2	17.7	17.0	0.049
twsg1a	84037	twisted gastrulation homolog 1a (Drosophila)	3.77E-02	4.71E-01	12.0	11.2	11.6	0.049
ugdh	116991	UDP-glucose dehydrogenase	4.64E-01	1.41E-01	2.2	3.5	2.9	0.049
gnat2	140429	guanine nucleotide binding protein (G protein), alpha trar	7.81E-02	4.57E-01	2.0	3.7	2.9	0.049
ND1	140531	NADH dehydrogenase subunit 1	1.09E-01	4.86E-01	1.3	3.0	2.2	0.049
lrrc17	321202	leucine rich repeat containing 17	2.48E-01	4.99E-01	3.8	5.5	4.7	0.049
h1fx	322508	H1 histone family, member X	4.74E-01	4.92E-01	12.7	15.2	14.0	0.049
pfdn2	323012	prefoldin subunit 2	2.53E-01	4.83E-01	0.3	2.3	1.3	0.049
zgc:165515	323052	zgc:165515	1.20E-02	4.52E-01	4.7	4.7	4.7	0.049
wbp2	325287	WW domain binding protein 2	4.66E-01	2.02E-02	17.3	18.0	17.7	0.049
gclc	326857	glutamate-cysteine ligase, catalytic subunit	4.72E-01	4.69E-01	2.2	2.3	2.3	0.049
rasgef1ba	327572	RasGEF domain family, member 1Ba	6.43E-02	4.55E-01	4.5	4.8	4.7	0.049
zgc:66432	327616	zgc:66432	1.35E-01	4.99E-01	20.3	23.8	22.1	0.049
gnsb	327635	glucosamine (N-acetyl)-6-sulfatase (Sanfilippo disease II	4.68E-01	8.63E-02	16.2	17.2	16.7	0.049
wu:fi28f11	334054	wu:fi28f11	4.82E-01	4.62E-01	13.2	15.7	14.5	0.049
mbtps2	334658	membrane-bound transcription factor protease, site 2	2.49E-01	4.91E-01	14.8	16.0	15.4	0.049
wu:fk47b06	334660	wu:fk47b06	1.09E-01	4.78E-01	1.0	1.2	1.1	0.049
acsbg2	335159	acyl-CoA synthetase bubblegum family member 2	4.94E-01	6.01E-02	15.8	16.8	16.3	0.049
atp5o	335191	ATP synthase, H+ transporting, mitochondrial F1 comple	4.32E-01	4.97E-01	2.3	5.0	3.7	0.049
fbxo42	335459	F-box protein 42	3.44E-01	4.81E-01	15.7	15.5	15.6	0.049
sod2	335799	superoxide dismutase 2, mitochondrial	6.72E-02	4.79E-01	4.7	2.5	3.6	0.049
nkapl	336120	NFKB activating protein-like	3.36E-01	4.87E-01	16.0	16.7	16.4	0.049
slc38a6	336741	solute carrier family 38, member 6	3.87E-03	4.77E-01	15.3	18.7	17.0	0.049
si:dkey-256h2.1	337520	si:dkey-256h2.1	1.71E-01	4.60E-01	13.8	15.8	14.8	0.049

Table S1: Zebrafish circadian oscillating genes

Gene Symbol	Gene ID	Gene Description	Fisher's g test p value (LD)	Fisher's g test p value (DD)	LD Phase	DD Phase	Phase *	FDR **
wu:fj81e10	337532	wu:fj81e10	2.76E-02	4.51E-01	14.7	16.7	15.7	0.049
unc119.1	338230	unc-119 homolog 1	1.72E-01	4.75E-01	11.8	16.3	14.1	0.049
echdc2	338247	enoyl CoA hydratase domain containing 2	1.69E-01	4.69E-01	17.5	21.7	19.6	0.049
nptna	368305	neuroplastin a	1.94E-01	4.67E-01	17.5	18.0	17.8	0.049
inadl	368723	InaD-like (Drosophila)	4.78E-01	2.71E-02	22.0	19.0	20.5	0.049
cecr1a	373884	cat eye syndrome chromosome region, candidate 1a	2.02E-01	4.52E-01	13.3	15.3	14.3	0.049
zgc:64114	378866	zgc:64114	4.62E-01	7.18E-02	23.3	0.0	23.7	0.049
zgc:56112	393164	zgc:56112	1.10E-01	4.71E-01	14.0	16.0	15.0	0.049
scg3	393730	secretogranin III	4.65E-02	4.88E-01	15.2	14.8	15.0	0.049
zgc:73273	393744	zgc:73273	1.13E-02	4.70E-01	3.2	4.7	4.0	0.049
eif4a3	394053	eukaryotic translation initiation factor 4A, isoform 3	7.11E-02	4.61E-01	21.5	20.8	21.2	0.049
zgc:63907	394112	zgc:63907	4.61E-01	4.01E-01	10.8	14.0	12.4	0.049
zgc:110591	402849	zgc:110591	2.42E-01	4.56E-01	15.3	16.7	16.0	0.049
evlb	402929	Enah/Vasp-like b	4.81E-01	4.36E-01	12.5	10.2	11.4	0.049
dao.3	402994	D-amino-acid oxidase 3	4.64E-01	1.24E-05	3.2	5.5	4.4	0.049
igfbp5a	403039	insulin-like growth factor binding protein 5a	1.97E-01	4.57E-01	23.0	21.7	22.4	0.049
b3galt2	404633	UDP-Gal:betaGlcNAc beta 1,3-galactosyltransferase, po	2.82E-01	4.96E-01	14.8	15.3	15.1	0.049
uros	404731	uroporphyrinogen III synthase	2.62E-01	4.52E-01	14.0	16.2	15.1	0.049
mettl11a	405834	methyltransferase like 11A	4.68E-01	4.92E-01	23.3	2.5	0.9	0.049
hnrnpk	406267	heterogeneous nuclear ribonucleoprotein K	3.60E-02	4.95E-01	4.8	4.8	4.8	0.049
rbm4.3	406277	RNA binding motif protein 4.3	4.27E-01	4.98E-01	1.7	21.0	23.4	0.049
fubp1	406418	far upstream element (FUSE) binding protein 1	4.24E-01	4.96E-01	17.2	20.5	18.9	0.049
ptp4a3	406460	protein tyrosine phosphatase type IVA, member 3	1.18E-01	4.50E-01	17.7	19.0	18.4	0.049
zgc:63629	406676	zgc:63629	1.20E-02	4.58E-01	15.8	17.8	16.8	0.049
fam136a	406709	family with sequence similarity 136, member A	8.10E-02	4.95E-01	0.5	2.0	1.3	0.049
pla2g12b	406739	phospholipase A2, group XIIB	4.95E-01	2.46E-01	3.5	6.3	4.9	0.049
serinc5	406835	serine incorporator 5	1.90E-01	4.63E-01	12.3	13.2	12.8	0.049
accn2a	407672	amiloride-sensitive cation channel 2 a	4.78E-01	4.38E-01	20.5	19.3	19.9	0.049
zgc:91874	431735	zgc:91874	6.45E-02	4.62E-01	22.8	0.5	23.7	0.049
dusp22a	436787	dual specificity phosphatase 22a	4.69E-01	4.32E-02	11.2	15.7	13.5	0.049
pir	436823	pirin	2.50E-02	4.78E-01	14.8	18.0	16.4	0.049

Table S1: Zebrafish circadian oscillating genes

Gene Symbol	Gene ID	Gene Description	Fisher's g test p value (LD)	Fisher's g test p value (DD)	LD Phase	DD Phase	Phase *	FDR **
egln1	436868	egl nine homolog 1 (C. elegans)	4.80E-01	4.51E-01	17.0	19.3	18.2	0.049
lrrc57	436900	leucine rich repeat containing 57	1.47E-02	4.57E-01	11.5	13.2	12.4	0.049
wipi2	437002	WD repeat domain, phosphoinositide interacting 2	3.06E-01	4.86E-01	13.8	16.0	14.9	0.049
suds3	445099	suppressor of defective silencing 3 homolog (SDS3, S. c	4.06E-01	4.62E-01	12.8	15.2	14.0	0.049
capn9	445107	calpain 9	1.12E-01	4.67E-01	1.8	4.7	3.3	0.049
camk2d1	445208	calcium/calmodulin-dependent protein kinase (CaM kina	4.50E-02	4.79E-01	6.8	8.8	7.8	0.049
zgc:100942	445232	zgc:100942	1.40E-01	4.77E-01	15.3	18.0	16.7	0.049
cldn8	445278	claudin 8	5.28E-02	4.68E-01	9.8	9.2	9.5	0.049
zgc:92806	447829	zgc:92806	4.60E-01	4.28E-01	18.7	19.7	19.2	0.049
abhd3	447830	abhydrolase domain containing 3	8.27E-02	4.70E-01	1.2	1.8	1.5	0.049
zgc:92353	447834	zgc:92353	4.91E-01	3.63E-02	21.5	21.8	21.7	0.049
cx47.1	447835	connexin 47.1	4.53E-01	2.95E-01	22.0	0.7	23.4	0.049
uck1	447928	uridine-cytidine kinase 1	1.73E-01	4.99E-01	16.2	14.5	15.4	0.049
chga	450039	chromogranin A	8.89E-02	4.58E-01	14.7	16.3	15.5	0.049
alg14	450072	asparagine-linked glycosylation 14 homolog (S. cerevisia	6.49E-03	4.62E-01	1.7	0.7	1.2	0.049
zgc:92107	492329	zgc:92107	4.55E-01	3.70E-01	16.2	17.7	17.0	0.049
gga3	492643	golgi associated, gamma adaptin ear containing, ARF bi	2.74E-01	4.58E-01	15.2	13.7	14.5	0.049
aldh16a1	492710	aldehyde dehydrogenase 16 family, member A1	4.73E-01	3.36E-01	0.7	2.7	1.7	0.049
pcdh2ab1	493589	protocadherin 2 alpha b 1	6.03E-02	4.76E-01	14.0	15.0	14.5	0.049
stip1	493606	stress-induced-phosphoprotein 1 (Hsp70/Hsp90-organizi	9.27E-02	4.98E-01	3.2	3.5	3.4	0.049
net1	493607	neuroepithelial cell transforming gene 1	3.21E-01	4.69E-01	4.2	3.3	3.8	0.049
xrg5	497102	XK-related protein 5	2.02E-01	4.96E-01	15.0	17.2	16.1	0.049
opa3	497278	optic atrophy 3	3.54E-02	4.90E-01	0.0	0.8	0.4	0.049
zgc:113200	503759	zgc:113200	1.01E-01	4.98E-01	15.5	18.0	16.8	0.049
im:7152917	504109	im:7152917	4.86E-02	4.52E-01	16.8	18.7	17.8	0.049
gpr22	504167	G protein-coupled receptor 22	2.37E-01	4.51E-01	13.5	14.6	14.0	0.049
ghrhra	541314	growth hormone releasing hormone receptor a	4.96E-01	3.28E-01	13.3	14.8	14.1	0.049
gcsha	541329	glycine cleavage system protein H (aminomethyl carrier)	4.94E-01	3.18E-01	0.3	2.7	1.5	0.049
zgc:112998	541430	zgc:112998	4.72E-01	3.45E-01	14.3	12.3	13.3	0.049
sorbs3	541478	sorbin and SH3 domain containing 3	4.71E-01	4.36E-03	4.3	4.2	4.3	0.049
zgc:113119	541480	zgc:113119	4.76E-01	4.17E-01	14.0	14.2	14.1	0.049

Table S1: Zebrafish circadian oscillating genes

Gene Symbol	Gene ID	Gene Description	Fisher's g test p value (LD)	Fisher's g test p value (DD)	LD Phase	DD Phase	Phase *	FDR **
snrpd2	550244	small nuclear ribonucleoprotein D2 polypeptide	4.99E-01	4.37E-01	1.0	3.7	2.4	0.049
cdk10	550285	cyclin-dependent kinase 10	4.33E-01	4.66E-01	21.2	23.8	22.5	0.049
asb4	550321	ankyrin repeat and SOCS box-containing 4	3.52E-01	4.73E-01	15.7	18.3	17.0	0.049
cyr61l1	550346	cysteine-rich, angiogenic inducer 61 like 1	4.94E-01	2.98E-02	11.5	18.5	15.0	0.049
zgc:112104	550440	zgc:112104	4.29E-02	4.97E-01	21.5	23.3	22.4	0.049
zgc:110333	550497	zgc:110333	2.42E-01	4.56E-01	10.3	11.3	10.8	0.049
guf1	553030	GUF1 GTPase homolog (S. cerevisiae)	3.68E-02	4.90E-01	0.2	23.8	0.0	0.049
dnmt8	553187	DNA (cytosine-5-)-methyltransferase 8	4.19E-02	4.55E-01	2.0	3.8	2.9	0.049
bms1l	553241	BMS1-like, ribosome assembly protein (yeast)	2.64E-02	4.53E-01	1.0	2.8	1.9	0.049
zgc:152896	553288	zgc:152896	4.71E-01	3.79E-01	22.8	1.3	0.1	0.049
si:ch73-167c12.2	553421	si:ch73-167c12.2	3.56E-02	4.68E-01	15.7	16.2	16.0	0.049
si:ch211-235e18.3	553465	si:ch211-235e18.3	2.82E-01	4.51E-01	17.8	18.3	18.1	0.049
trit1	553523	tRNA isopentenyltransferase 1	2.36E-01	4.70E-01	21.3	0.7	23.0	0.049
zgc:110664	553647	zgc:110664	2.60E-01	4.91E-01	23.7	2.8	1.3	0.049
zgc:112332	553712	zgc:112332	3.77E-01	4.90E-01	9.0	11.7	10.4	0.049
nr3c1	553740	nuclear receptor subfamily 3, group C, member 1 (glucoc	9.27E-03	4.58E-01	13.1	14.4	13.7	0.049
zgc:113184	553745	zgc:113184	2.16E-02	4.81E-01	9.2	10.2	9.7	0.049
zgc:110708	553793	zgc:110708	5.00E-01	4.01E-01	13.8	15.3	14.6	0.049
si:ch211-279l9.6	555701	si:ch211-279l9.6	3.37E-01	4.98E-01	1.3	0.7	1.0	0.049
akap1b	556208	A kinase (PRKA) anchor protein 1b	4.98E-02	4.90E-01	21.3	22.0	21.7	0.049
si:ch211-192p3.1	556298	si:ch211-192p3.1	3.36E-02	4.83E-01	10.5	13.5	12.0	0.049
zgc:161973	556635	zgc:161973	7.14E-03	4.81E-01	4.0	0.5	2.3	0.049
irf2bp1	556852	interferon regulatory factor 2 binding protein 1	4.86E-01	2.03E-01	15.2	19.3	17.3	0.049
si:dkey-120m5.1	557744	si:dkey-120m5.1	2.37E-02	4.64E-01	16.3	18.7	17.5	0.049
emd	558212	emerin (Emery-Dreifuss muscular dystrophy)	1.28E-01	4.70E-01	5.2	5.0	5.1	0.049
cdc42ep1	558275	CDC42 effector protein (Rho GTPase binding) 1	4.65E-01	2.10E-01	6.8	6.8	6.8	0.049
LOC558375	558375	tripartite motif-containing protein 16-like	4.43E-01	4.62E-01	15.0	17.0	16.0	0.049
LOC558402	558402	novel protein similar to vertebrate anaphase promoting c	4.99E-01	6.30E-02	15.3	14.3	14.8	0.049
si:ch211-127h20.2	558635	si:ch211-127h20.2	4.69E-01	9.22E-03	16.3	18.5	17.4	0.049
macc1	558777	metastasis associated in colon cancer 1	4.64E-01	4.82E-01	5.8	5.8	5.8	0.049
im:7136185	559152	im:7136185	3.19E-01	4.93E-01	10.8	14.8	12.8	0.049

Table S1: Zebrafish circadian oscillating genes

Gene Symbol	Gene ID	Gene Description	Fisher's g test p value (LD)	Fisher's g test p value (DD)	LD Phase	DD Phase	Phase *	FDR **
si:dkey-205h13.2	561081	si:dkey-205h13.2	1.20E-02	4.90E-01	16.0	18.2	17.1	0.049
zgc:153218	561673	zgc:153218	4.69E-01	4.23E-01	17.3	19.8	18.6	0.049
zgc:114097	561675	zgc:114097	2.27E-01	4.81E-01	18.5	15.8	17.2	0.049
ints1	562131	integrator complex subunit 1	8.60E-02	4.98E-01	15.8	17.7	16.8	0.049
zgc:154088	562137	zgc:154088	1.23E-01	4.69E-01	14.5	16.2	15.4	0.049
si:dkeyp-59a8.4	562918	si:dkeyp-59a8.4	1.12E-01	4.64E-01	14.2	15.7	15.0	0.049
ankhd1	563327	ankyrin repeat and KH domain containing 1	4.32E-01	4.56E-01	22.2	19.3	20.8	0.049
rhcg2a	563826	Rh type C glycoprotein2a	2.81E-01	4.54E-01	5.2	4.3	4.8	0.049
shdb	564537	Src homology 2 domain containing transforming protein I	2.90E-01	4.79E-01	16.7	17.7	17.2	0.049
zgc:114081	564984	zgc:114081	4.93E-01	1.80E-01	15.3	10.3	12.8	0.049
tjp2a	565446	tight junction protein 2a (zona occludens 2)	5.96E-02	4.51E-01	8.2	8.2	8.2	0.049
apba1	565507	amyloid beta (A4) precursor protein-binding, family A, me	4.81E-01	7.12E-02	17.7	15.3	16.5	0.049
si:ch211-206k20.5	565648	si:ch211-206k20.5	4.57E-01	1.57E-01	12.8	11.3	12.1	0.049
zgc:114173	566797	zgc:114173	4.45E-01	4.69E-01	16.2	11.2	13.7	0.049
gfpt1	567861	glutamine-fructose-6-phosphate transaminase 1	1.23E-01	4.63E-01	2.2	3.3	2.8	0.049
cog5	567886	component of oligomeric golgi complex 5	8.42E-03	4.99E-01	20.7	20.3	20.5	0.049
bcar1	568309	breast cancer anti-estrogen resistance 1	4.07E-01	4.77E-01	16.5	17.7	17.1	0.049
ctsbb	569298	capthepsin B, b	2.46E-01	4.67E-01	14.5	13.5	14.0	0.049
serinc2	569338	serine incorporator 2	7.35E-02	4.51E-01	15.7	18.0	16.9	0.049
dmxl2	569435	Dmx-like 2	4.68E-01	7.45E-02	23.3	18.0	20.7	0.049
usp16	569700	ubiquitin specific protease 16	4.65E-01	8.47E-04	0.2	22.8	23.5	0.049
zgc:165534	569719	zgc:165534	4.13E-02	4.54E-01	7.0	4.2	5.6	0.049
ttc21b	569823	tetratricopeptide repeat domain 21B	9.97E-02	4.83E-01	11.2	12.8	12.0	0.049
si:dkey-153k10.12	570295	si:dkey-153k10.12	4.84E-01	4.60E-02	12.8	15.8	14.3	0.049
lsm7	573047	LSM7 homolog, U6 small nuclear RNA associated (S. ce	1.86E-01	4.58E-01	4.7	5.0	4.9	0.049
ripply3	654448	ripply3	4.36E-01	4.97E-01	4.5	2.2	3.4	0.049
zgc:136963	664694	zgc:136963	4.80E-01	1.07E-02	1.3	2.7	2.0	0.049
zgc:136971	678599	zgc:136971	4.89E-01	3.03E-01	15.0	16.8	15.9	0.049
zgc:136597	692315	zgc:136597	4.55E-01	4.15E-01	15.7	17.0	16.4	0.049
mrps14	751639	mitochondrial ribosomal protein S14	4.71E-01	1.03E-01	1.0	4.8	2.9	0.049
zgc:153184	751652	zgc:153184	1.88E-01	4.96E-01	23.5	5.2	2.4	0.049

Table S1: Zebrafish circadian oscillating genes

Gene Symbol	Gene ID	Gene Description	Fisher's g test p value (LD)	Fisher's g test p value (DD)	LD Phase	DD Phase	Phase *	FDR **
zgc:153662	751665	zgc:153662	4.94E-01	4.86E-02	8.8	9.7	9.3	0.049
zgc:153251	751671	zgc:153251	2.64E-02	4.75E-01	12.5	15.3	13.9	0.049
tmem14c	751728	transmembrane protein 14C	2.97E-01	4.91E-01	2.5	3.2	2.9	0.049
urah	751742	HIU hydrolase	1.81E-02	4.64E-01	6.2	5.5	5.9	0.049
mrps6	751746	mitochondrial ribosomal protein S6	1.57E-01	4.68E-01	0.0	2.2	1.1	0.049
zgc:153704	767759	zgc:153704	1.13E-01	4.70E-01	9.7	9.0	9.4	0.049
zgc:154086	768184	zgc:154086	4.70E-01	2.59E-01	1.2	2.5	1.9	0.049
zgc:153060	768200	zgc:153060	8.70E-02	4.52E-01	23.3	1.8	0.6	0.049
zgc:153377	768305	zgc:153377	2.03E-01	4.76E-01	23.0	2.7	0.9	0.049
rnf115	790928	ring finger protein 115	1.75E-03	4.89E-01	15.2	16.0	15.6	0.049
LOC793689	793689	hypothetical protein LOC793689	4.58E-01	3.34E-04	11.7	14.7	13.2	0.049
LOC793763	793763	transmembrane protein 151B-like	4.21E-01	4.77E-01	15.7	16.0	15.9	0.049
im:6894391	793937	im:6894391	3.49E-01	4.62E-01	7.7	6.2	7.0	0.049
LOC794214	794214	protein NLRC3-like	3.50E-01	4.58E-01	13.8	15.7	14.8	0.049
tfpt	796934	TCF3 fusion partner homolog	1.16E-02	4.64E-01	12.7	15.8	14.3	0.049
zgc:171711	797287	zgc:171711	4.83E-01	4.02E-01	13.8	14.7	14.3	0.049
si:dkey-236e20.7	798655	si:dkey-236e20.7	2.95E-01	4.64E-01	16.2	16.8	16.5	0.049
LOC100000832	100000832	zinc-binding protein A33-like	5.92E-04	4.79E-01	13.3	16.0	14.7	0.049
org	100001110	oogenesis-related gene	4.77E-01	7.21E-02	2.0	2.3	2.2	0.049
taf7	100002261	TAF7 RNA polymerase II, TATA box binding protein (TBI	1.06E-01	4.56E-01	16.7	18.3	17.5	0.049
LOC100002505	100002505	MAP7 domain-containing protein 2-like	3.31E-01	4.82E-01	23.3	21.5	22.4	0.049
si:ch73-78i5.3	100003752	si:ch73-78i5.3	1.42E-01	4.96E-01	14.8	15.5	15.2	0.049
cdx1b	100004956	caudal type homeobox 1 b	6.30E-02	4.76E-01	13.7	15.0	14.4	0.049
LOC100005871	100005871	tripartite motif-containing protein 16-like	1.35E-01	4.61E-01	14.5	18.3	16.4	0.049
LOC100007966	100007966	zinc finger protein 567-like	4.92E-01	5.42E-02	9.7	8.7	9.2	0.049
zgc:162612	100037333	zgc:162612	9.02E-02	4.56E-01	16.7	18.5	17.6	0.049
zgc:165608	100073339	zgc:165608	3.49E-02	4.50E-01	15.7	19.3	17.5	0.049
zgc:165601	100073344	zgc:165601	2.44E-01	4.53E-01	15.7	18.0	16.9	0.049
fdx1	100126232	ferredoxin 1	1.75E-01	4.74E-01	2.3	2.3	2.3	0.049
si:dkey-170o10.3	100137123	si:dkey-170o10.3	2.10E-01	4.87E-01	0.8	1.3	1.1	0.049
zgc:171356	100144565	zgc:171356	6.68E-02	4.70E-01	1.2	0.3	0.8	0.049

Table S1: Zebrafish circadian oscillating genes

Gene Symbol	Gene ID	Gene Description	Fisher's g test p value (LD)	Fisher's g test p value (DD)	LD Phase	DD Phase	Phase *	FDR **
si:ch211-202h22.8	100307107	si:ch211-202h22.8	3.72E-01	4.71E-01	2.5	3.8	3.2	0.049
LOC100329265	100329265	hypothetical protein LOC100329265	1.05E-03	4.86E-01	23.0	0.2	23.6	0.049
gpam	100332911	glycerol-3-phosphate acyltransferase, mitochondrial	4.62E-01	1.17E-01	15.0	16.2	15.6	0.049
LOC100333916	100333916	myomegalin-like	4.61E-01	3.11E-01	16.7	17.3	17.0	0.049

* The mean circadian phases under LD and DD are used to represent the circadian phases of ZCOGs

** False Discovery Rate
