# Supplementary material for: Analysis of a Gene Regulatory Cascade Mediating Circadian Rhythm in Zebrafish
Source: PLoS Comput Biol. 2013 Feb 28;9(2):e1002940. doi: 10.1371/journal.pcbi.1002940 (PMC3585402; doi:10.1371/journal.pcbi.1002940)
Supplement: Table S2 — Functional enrichment of ZCOGs (P<0.01). (PDF) [file pcbi.1002940.s007.pdf]

Table S2: Functional enrichment of ZCOGs (P <0.01)

Term Type	Term ID	Included gene number	Fisher P_Value	Odds Ratio	Term Name
Gene Ontology	GO:0009416	20	2.17E-06	4.95	response to light stimulus
Gene Ontology	GO:0009648	9	9.61E-06	19.92	photoperiodism
Gene Ontology	GO:0009628	26	1.96E-03	2.18	response to abiotic stimulus
Gene Ontology	GO:0007623	7	2.44E-03	6.18	circadian rhythm
Gene Ontology	GO:0022857	33	4.46E-03	1.86	transmembrane transporter activity
Gene Ontology	GO:0005215	34	5.68E-03	1.80	transporter activity
Gene Ontology	GO:0017076	9	5.88E-03	3.62	purine nucleotide binding
Gene Ontology	GO:0022890	13	6.44E-03	2.74	inorganic cation transmembrane transporter activity
Gene Ontology	GO:0048546	8	6.74E-03	3.93	digestive tract morphogenesis
Gene Ontology	GO:0015840	6	7.88E-03	5.30	urea transport
Gene Ontology	GO:0030522	6	7.88E-03	5.30	intracellular receptor mediated signaling pathway
Gene Ontology	GO:0042886	6	7.88E-03	5.30	amide transport
Gene Ontology	GO:0022891	29	8.60E-03	1.84	substrate-specific transmembrane transporter activity
KEGG Pathway	dre04710	21	2.87E-10	11.18	Circadian rhythm - mammal
KEGG Pathway	dre03050	22	8.88E-05	3.19	Proteasome
KEGG Pathway	dre04146	27	6.09E-04	2.35	Peroxisome
KEGG Pathway	dre03008	24	1.90E-03	2.25	Ribosome biogenesis in eukaryotes
KEGG Pathway	dre00980	15	2.02E-03	2.99	Metabolism of xenobiotics by cytochrome P450
Tissues	ZFA:0000143	46	2.98E-09	3.45	retinal photoreceptor layer
Tissues	ZFA:0001076	68	3.81E-07	2.24	intestinal bulb
Tissues	ZFA:0001678	160	2.73E-06	1.61	immature eye
Tissues	ZFA:0000152	267	3.00E-06	1.46	retina
Tissues	ZFA:0009262	16	3.59E-06	6.34	retinal cone cell
Tissues	ZFA:0000279	86	4.81E-05	1.74	segmental plate
Tissues	ZFA:0000088	120	5.06E-05	1.60	YSL
Tissues	ZFA:0001357	96	5.84E-05	1.68	alar plate midbrain region
Tissues	ZFA:0000155	224	7.75E-05	1.40	somite
Tissues	ZFA:0001056	146	1.29E-04	1.49	myotome
Tissues	ZFA:0000123	211	1.33E-04	1.40	liver
Tissues	ZFA:0001464	17	3.18E-04	3.37	retinal outer nuclear layer
Tissues	ZFA:0000019	101	5.19E-04	1.54	epiphysis
Tissues	ZFA:0000095	137	1.14E-03	1.41	pharyngeal arch 3-7 skeleton
Tissues	ZFA:0000112	134	1.93E-03	1.39	gut
Tissues	ZFA:0001130	4	5.30E-03	17.30	pharyngeal pouch 2

Table S2: Functional enrichment of ZCOGs (P <0.01)

Term Type	Term ID	Included gene number	Fisher P_Value	Odds Ratio	Term Name
Tissues	ZFA:0001390	30	6.36E-03	1.87	pancreatic bud
Tissues	ZFA:0000119	55	6.78E-03	1.57	retinal inner nuclear layer
Tissues	ZFA:0000132	110	8.31E-03	1.36	neural plate
Tissues	ZFA:0000173	6	8.51E-03	5.20	bulbus arteriosus
Tissues	ZFA:0001621	6	8.51E-03	5.20	pronephric proximal straight tubule
