# Supplementary material for: Analysis of a Gene Regulatory Cascade Mediating Circadian Rhythm in Zebrafish
Source: PLoS Comput Biol. 2013 Feb 28;9(2):e1002940. doi: 10.1371/journal.pcbi.1002940 (PMC3585402; doi:10.1371/journal.pcbi.1002940)
Supplement: Table S3 — ZCOGs with mouse circadian gene homologs. (PDF) [file pcbi.1002940.s008.pdf]

Table S3: ZCOGs with mouse circadian gene homologs.

Mouse Gene Symbol	Zebrafish Gene Symbol	Evidence	Mouse Gene phase	Zebrafish Gene phase
Acsl1	zgc:101071	NCBI HomoloGene,Ensembl Orthology	7.2	0.7
Acsl1	acsl1	Inparanoid,NCBI HomoloGene,Ensembl Orthology	7.2	4.0
Acsl5	acsl5	Inparanoid,NCBI HomoloGene,ZFIN,Ensembl Orthology	1.4	5.1
Arntl	arntl1b	ZFIN,Ensembl Orthology	22.4	12.8
Arntl	arntl1a	Inparanoid,ZFIN,Ensembl Orthology	22.4	13.4
Cdkn1a	LOC100004175	Ensembl Possible Orthology	22.4	3.5
Cirbp	cirbp	Ensembl Orthology	5.8	16.5
Clock	clock3	ZFIN,Ensembl Orthology	23.4	15.1
Clock	clock	Inparanoid,NCBI HomoloGene,ZFIN,Ensembl Orthology	23.4	15.1
Cry1	cry1b	ZFIN,Ensembl Possible Orthology	18.0	6.5
Cry1	cry5	Ensembl Possible Orthology	18.0	4.0
Cry1	cry-dash	Ensembl Possible Orthology	18.0	3.6
Cry1	cry2a	NCBI HomoloGene,Ensembl Orthology	18.0	13.3
Cry1	cry4	Ensembl Possible Orthology	18.0	14.3
Cry1	cry2b	Ensembl Possible Orthology	18.0	14.1
Dbp	dbpb	Ensembl Orthology	9.2	23.3
Dnaja1	dnaja1l	Inparanoid,NCBI HomoloGene,Ensembl Orthology	17.0	4.9
Ei24	zgc:110605	Inparanoid,NCBI HomoloGene,Ensembl Orthology	1.1	13.6
Eif1a	eif1axa	Ensembl Possible Orthology	20.9	1.8
Elovl5	zgc:153394	Ensembl Possible Orthology	21.8	2.3
Ephx1	ephx1	Inparanoid,NCBI HomoloGene,Ensembl Orthology	9.7	6.8
Fus	fus	Inparanoid,NCBI HomoloGene,ZFIN,Ensembl Orthology	4.8	15.0
Gja1	cx43	Inparanoid,NCBI HomoloGene,ZFIN,Ensembl Orthology	1.1	1.8
Gja1	cx28.9	Ensembl Possible Orthology	1.1	0.4
Gja1	cx32.3	Ensembl Possible Orthology	1.1	0.3
Glul	glulb	Inparanoid,NCBI HomoloGene,ZFIN,Ensembl Orthology	16.9	6.3
Glul	glula	ZFIN,Ensembl Possible Orthology	16.9	4.9
Hmgb3	hmgb1a	Ensembl Orthology	6.1	17.8
Hmgb3	hmgb3b	NCBI HomoloGene,ZFIN	6.1	3.7
Hsp90aa1	hsp90a.1	Ensembl Orthology	19.7	2.4
Hsp90aa1	hsp90a.2	Inparanoid,NCBI HomoloGene,Ensembl Orthology	19.7	4.3
Hsp90ab1	hsp90ab1	Inparanoid,NCBI HomoloGene,ZFIN,Ensembl Orthology	19.8	16.7
Litaf	si:ch211-202h22.8	Ensembl Possible Orthology	1.4	3.2
Mid1ip1	mid1ip1	Ensembl Possible Orthology	14.3	3.7

Table S3: ZCOGs with mouse circadian gene homologs.

Mouse Gene Symbol	Zebrafish Gene Symbol	Evidence	Mouse Gene phase	Zebrafish Gene phase
Mmp14	mmp14a	ZFIN,Ensembl Orthology	1.0	17.7
Mmp14	mmp14b	Inparanoid,NCBI HomoloGene,ZFIN,Ensembl Orthology	1.0	15.5
Net1	net1	Inparanoid,NCBI HomoloGene,Ensembl Orthology	6.5	3.8
Nfe2l2	nfe2l2	Inparanoid,NCBI HomoloGene,ZFIN,Ensembl Orthology	12.8	7.1
Nfil3	nfil3-6	Ensembl Possible Orthology	20.3	5.9
Nfil3	nfil3	Inparanoid,NCBI HomoloGene,ZFIN,Ensembl Orthology	20.3	14.4
Nr1d1	nr1d4b	Ensembl Possible Orthology	6.2	19.8
Nr1d1	nr1d1	Inparanoid,NCBI HomoloGene,ZFIN,Ensembl Orthology	6.2	23.8
Nr1d1	nr1d4a	Ensembl Possible Orthology	6.2	19.8
Nr1d2	nr1d4a	Ensembl Possible Orthology	9.6	19.8
Nr1d2	nr1d4b	Ensembl Possible Orthology	9.6	19.8
P4ha1	p4ha1b	Inparanoid,Ensembl Possible Orthology	17.5	1.3
Pdxk	pdxka	Inparanoid,NCBI HomoloGene,ZFIN,Ensembl Orthology	13.3	6.3
Per1	per1b	Inparanoid,NCBI HomoloGene,ZFIN,Ensembl Orthology	11.4	3.3
Per1	per1a	NCBI HomoloGene,ZFIN,Ensembl Orthology	11.4	1.0
Per2	per2	Inparanoid,NCBI HomoloGene,ZFIN,Ensembl Orthology	13.2	7.5
Per3	per3	Inparanoid,NCBI HomoloGene,ZFIN,Ensembl Orthology	11.4	4.2
Pim3	zgc:113028	Ensembl Orthology	8.1	13.4
Pnp	pnp5b	NCBI HomoloGene,Ensembl Orthology	19.9	0.9
Pnp	pnp4b	NCBI HomoloGene,Ensembl Possible Orthology	19.9	6.1
Pnp	pnp5a	NCBI HomoloGene,Ensembl Orthology	19.9	6.5
Pnp	pnp6	NCBI HomoloGene,Ensembl Possible Orthology	19.9	2.8
Ppp1r3c	ppp1r3cb	Inparanoid,NCBI HomoloGene,ZFIN,Ensembl Orthology	20.8	10.6
Prkar1a	prkar1aa	Ensembl Orthology	2.5	15.3
Psmd11	psmd11b	NCBI HomoloGene,ZFIN,Ensembl Orthology	10.0	16.5
Rorc	rorca	ZFIN	18.3	10.8
Rorc	rorcb	ZFIN	18.3	12.4
Sbk1	LOC557524	Ensembl Possible Orthology	17.8	23.8
Serpinh1	hsp47	NCBI HomoloGene,ZFIN,Ensembl Orthology	20.1	5.0
Serpinh1	zgc:171630	Inparanoid,NCBI HomoloGene,Ensembl Orthology	20.1	4.6
Slc4a4	slc4a4a	Inparanoid,NCBI HomoloGene,ZFIN,Ensembl Orthology	6.8	4.5
St13	st13	Inparanoid,NCBI HomoloGene,ZFIN,Ensembl Orthology	19.3	7.6
Sult1a1	sult1st2	NCBI HomoloGene	17.1	5.6
Sult1a1	sult1st6	Ensembl Possible Orthology	17.1	4.2

Table S3: ZCOGs with mouse circadian gene homologs.

Mouse Gene Symbol	Zebrafish Gene Symbol	Evidence	Mouse Gene phase	Zebrafish Gene phase
Sult1a1	sult1st3	NCBI HomoloGene	17.1	5.2
Tef	tefa	Inparanoid,NCBI HomoloGene,ZFIN,Ensembl Orthology	11.5	3.4
Tef	tefb	Ensembl Orthology	11.5	1.9
Tsc22d3	tsc22d3	Inparanoid,ZFIN,Ensembl Orthology	15.7	8.6
Ubc	ubc	ZFIN,Ensembl Orthology	20.8	15.8
Ubc	ubb	NCBI HomoloGene	20.8	13.8
Xbp1	xbp1	Inparanoid,NCBI HomoloGene,ZFIN,Ensembl Orthology	16.4	2.9
Znrf2	znrf2b	Inparanoid,ZFIN	2.8	3.3
