# Supplementary material for: Analysis of a Gene Regulatory Cascade Mediating Circadian Rhythm in Zebrafish
Source: PLoS Comput Biol. 2013 Feb 28;9(2):e1002940. doi: 10.1371/journal.pcbi.1002940 (PMC3585402; doi:10.1371/journal.pcbi.1002940)
Supplement: Table S4 — Genes affected by light entrainment. (PDF) [file pcbi.1002940.s009.pdf]

Table S4: Genes affected by light entrainment.

Gene ID	Symbol	LD Phase	P value *	Light-Entrainment
336492	ptgds	7.8	1.56E-13	Fast Light-Induced
492491	zgc:103654	7.7	4.29E-13	Fast Light-Induced
450011	tspo	7.8	1.88E-12	Fast Light-Induced
402868	pnpo	4.8	1.42E-11	Fast Light-Induced
550383	npepl1	5.8	1.64E-11	Fast Light-Induced
569875	zgc:112356	7.7	2.20E-11	Fast Light-Induced
751701	zgc:153154	7.8	5.21E-11	Fast Light-Induced
337526	wu:fj81c05	7.2	8.23E-11	Fast Light-Induced
394146	dusp4	6.5	1.62E-10	Fast Light-Induced
373866	cbr1	5.8	3.50E-10	Fast Light-Induced
553538	loxl3	7.3	5.16E-10	Fast Light-Induced
557970	surf1	5.2	6.28E-10	Fast Light-Induced
324373	zgc:77513	8	6.70E-10	Fast Light-Induced
100000596	LOC100000596	8	1.14E-09	Fast Light-Induced
140431	guca1b	7	1.29E-09	Fast Light-Induced
560667	mtrr	3.8	3.44E-09	Fast Light-Induced
57969	copz2	6.8	3.55E-09	Fast Light-Induced
768159	si:ch211-195b13.1	5.8	3.79E-09	Fast Light-Induced
664750	ppox	6	5.99E-09	Fast Light-Induced
563732	uts1	6.7	6.09E-09	Fast Light-Induced
393186	zgc:56235	7.2	6.69E-09	Fast Light-Induced
797793	LOC797793	6.5	7.81E-09	Fast Light-Induced
352918	opn4l	7	1.16E-08	Fast Light-Induced
84041	bcdo2l	6.8	1.18E-08	Fast Light-Induced
393666	arl3l1	6.5	1.51E-08	Fast Light-Induced
393884	sdha	5.3	1.60E-08	Fast Light-Induced
447914	cox17	5.2	1.65E-08	Fast Light-Induced
798993	abca4a	5.8	1.67E-08	Fast Light-Induced
557634	LOC557634	3.7	1.68E-08	Fast Light-Induced
393253	neil1	7.8	2.16E-08	Fast Light-Induced
446130	si:ch211-237l4.6	6.2	2.43E-08	Fast Light-Induced
572207	zgc:73075	4.8	2.95E-08	Fast Light-Induced
58215	fech	6.8	2.99E-08	Fast Light-Induced
450030	zgc:101803	6.2	4.20E-08	Fast Light-Induced
791143	zgc:158419	5.8	4.34E-08	Fast Light-Induced
641489	zgc:123305	7.3	5.26E-08	Fast Light-Induced
767661	zgc:153046	5.5	5.71E-08	Fast Light-Induced
415184	msrb3	7.2	5.81E-08	Fast Light-Induced
553695	zgc:112255	5.2	5.86E-08	Fast Light-Induced
494078	zgc:101800	7.8	6.02E-08	Fast Light-Induced
394039	zgc:66475	6.7	6.60E-08	Fast Light-Induced

Table S4: Genes affected by light entrainment.

Gene ID	Symbol	LD Phase	P value *	Light-Entrainment
795535	LOC795535	6.7	6.67E-08	Fast Light-Induced
556575	LOC556575	5.3	8.09E-08	Fast Light-Induced
550575	rgra	6.3	8.87E-08	Fast Light-Induced
558071	atf6	2.8	9.61E-08	Fast Light-Induced
406605	zgc:85644	7.5	9.86E-08	Fast Light-Induced
568750	si:ch211-154o6.7	6.5	1.06E-07	Fast Light-Induced
791628	zgc:103539	6.7	1.47E-07	Fast Light-Induced
100003670	sepx1b	7.7	1.50E-07	Fast Light-Induced
436678	arr3a	4.7	1.58E-07	Fast Light-Induced
393762	zgc:73324	7.2	1.64E-07	Fast Light-Induced
445492	flad1	5.2	1.98E-07	Fast Light-Induced
556875	tmem177	5.8	1.98E-07	Fast Light-Induced
323679	srpk1a	6	2.05E-07	Fast Light-Induced
562149	sdhb	6.3	2.08E-07	Fast Light-Induced
445192	zgc:101042	3	2.51E-07	Fast Light-Induced
140633	per2	6.3	2.73E-07	Fast Light-Induced
541408	zgc:101788	4.3	3.30E-07	Fast Light-Induced
568835	slc27a6	7.3	3.60E-07	Fast Light-Induced
799177	LOC799177	6.3	3.68E-07	Fast Light-Induced
565126	zgc:153043	6.7	3.91E-07	Fast Light-Induced
550436	camk2a	4.3	3.93E-07	Fast Light-Induced
352916	sepx1a	8	3.94E-07	Fast Light-Induced
550573	c1qtnf1	6.7	4.00E-07	Fast Light-Induced
550345	syt13	6.7	4.09E-07	Fast Light-Induced
767788	zgc:153206	6.2	5.24E-07	Fast Light-Induced
791167	zgc:158222	3.5	5.91E-07	Fast Light-Induced
436771	hccsa	4.5	6.19E-07	Fast Light-Induced
394160	bokb	4.7	7.12E-07	Fast Light-Induced
541386	xpc	5.7	7.69E-07	Fast Light-Induced
556393	zgc:153679	5.5	7.89E-07	Fast Light-Induced
447802	zgc:92445	3.2	9.16E-07	Fast Light-Induced
373085	hspa9	2.7	9.19E-07	Fast Light-Induced
504039	im:7150988	5.2	9.21E-07	Fast Light-Induced
407654	mobkl1ab	4.7	1.02E-06	Fast Light-Induced
436959	blvrb	6.7	1.26E-06	Fast Light-Induced
798022	nt5c1ba	5.2	1.27E-06	Fast Light-Induced
30452	etv5b	6.5	1.49E-06	Fast Light-Induced
84039	bcmo1	4.7	1.63E-06	Fast Light-Induced
559429	si:dkey-246j7.1	5.3	1.65E-06	Fast Light-Induced
494065	dhodh	7	1.70E-06	Fast Light-Induced
566659	LOC566659	6	1.88E-06	Fast Light-Induced

Table S4: Genes affected by light entrainment.

Gene ID	Symbol	LD Phase	P value *	Light-Entrainment
553766	zgc:109977	4.8	2.41E-06	Fast Light-Induced
334202	plk3	6.3	2.42E-06	Fast Light-Induced
387304	cwf19l1	7.2	2.48E-06	Fast Light-Induced
799964	ptgesl	4.7	2.52E-06	Fast Light-Induced
550546	necab1	3	2.58E-06	Fast Light-Induced
564023	si:ch211-210c8.6	5.7	2.62E-06	Fast Light-Induced
570063	wdr76	5.7	2.67E-06	Fast Light-Induced
100005158	cox11	4	2.72E-06	Fast Light-Induced
436704	rgs20	2.2	2.80E-06	Fast Light-Induced
556360	stim1a	5.3	3.00E-06	Fast Light-Induced
436894	zgc:92254	7.5	3.17E-06	Fast Light-Induced
553553	cfdl	7	3.30E-06	Fast Light-Induced
405817	zgc:73371	4.2	3.51E-06	Fast Light-Induced
393426	ptpn11b	3.7	3.96E-06	Fast Light-Induced
402831	cyp27a7	6.8	4.04E-06	Fast Light-Induced
393146	slmapb	4.2	4.19E-06	Fast Light-Induced
568061	napepld	7.8	4.35E-06	Fast Light-Induced
100004133	ddb2	5.2	4.38E-06	Fast Light-Induced
393167	zgc:56136	6	4.40E-06	Fast Light-Induced
406662	sc4mol	6.5	4.51E-06	Fast Light-Induced
100334439	LOC100334439	3.5	4.76E-06	Fast Light-Induced
678653	unc119b	3	4.80E-06	Fast Light-Induced
394043	ephx1	6.8	5.21E-06	Fast Light-Induced
767700	zgc:153032	6.2	5.40E-06	Fast Light-Induced
556789	stxbp5a	3.7	5.40E-06	Fast Light-Induced
373099	guca1c	3.7	5.98E-06	Fast Light-Induced
30735	irbp	5.8	6.68E-06	Fast Light-Induced
494490	slc6a9	2.5	6.83E-06	Fast Light-Induced
777612	zgc:153914	3.7	6.91E-06	Fast Light-Induced
565437	si:dkey-221j11.2	4.7	7.29E-06	Fast Light-Induced
561272	ppcs	3.8	7.30E-06	Fast Light-Induced
564951	roraa	3.7	7.34E-06	Fast Light-Induced
100000576	LOC100000576	3.2	7.49E-06	Fast Light-Induced
767657	abhd12	4.7	7.49E-06	Fast Light-Induced
724007	stra6	3.7	8.17E-06	Fast Light-Induced
58134	adarb1	3.7	8.23E-06	Fast Light-Induced
556528	rdh5	5.2	8.31E-06	Fast Light-Induced
796867	arr3b	3.3	8.46E-06	Fast Light-Induced
554962	LOC554962	3.2	8.50E-06	Fast Light-Induced
562019	wu:fc07b10	3.5	8.79E-06	Fast Light-Induced
387527	cyp4t8	7	9.18E-06	Fast Light-Induced

Table S4: Genes affected by light entrainment.

Gene ID	Symbol	LD Phase	P value *	Light-Entrainment
437016	tk2	4.7	9.37E-06	Fast Light-Induced
407988	zgc:85746	4.5	9.60E-06	Fast Light-Induced
323326	zgc:73225	6.8	9.69E-06	Fast Light-Induced
83776	cry5	5	9.95E-06	Fast Light-Induced
724016	npas4	7.2	1.01E-05	Fast Light-Induced
550477	zgc:112432	5.7	1.03E-05	Fast Light-Induced
563405	gprc5c	2.7	1.04E-05	Fast Light-Induced
393322	tnfaip8l3	3.8	1.05E-05	Fast Light-Induced
445114	cdk5r2b	3.2	1.07E-05	Fast Light-Induced
767704	zgc:153118	5.3	1.14E-05	Fast Light-Induced
572463	ibtk	5	1.25E-05	Fast Light-Induced
565155	hsp90a.2	4.3	1.27E-05	Fast Light-Induced
100037332	zgc:162608	5.2	1.31E-05	Fast Light-Induced
100101646	zgc:165423	3.7	1.42E-05	Fast Light-Induced
100073345	zgc:165670	2.7	1.42E-05	Fast Light-Induced
569518	LOC569518	3.5	1.47E-05	Fast Light-Induced
799937	dio1	5.3	1.49E-05	Fast Light-Induced
100125912	prkg2	5.5	1.49E-05	Fast Light-Induced
100005148	ar	5	1.54E-05	Fast Light-Induced
100004913	phyhiplb	3.5	1.54E-05	Fast Light-Induced
449656	osbpl7	3.2	1.56E-05	Fast Light-Induced
791176	camk1d	3.2	1.62E-05	Fast Light-Induced
445044	rs1	2.2	1.76E-05	Fast Light-Induced
324822	actr6	3.5	1.79E-05	Fast Light-Induced
563956	si:busm1-265n4.4	5.8	1.90E-05	Fast Light-Induced
368857	si:busm1-241h12.4	2.3	1.92E-05	Fast Light-Induced
777708	zgc:153892	6	2.06E-05	Fast Light-Induced
378855	slmo2	2.8	2.07E-05	Fast Light-Induced
393580	zgc:63614	6.2	2.07E-05	Fast Light-Induced
64265	aox3	6.5	2.27E-05	Fast Light-Induced
327556	zgc:63480	2.8	2.30E-05	Fast Light-Induced
373084	hig1	2.7	2.53E-05	Fast Light-Induced
563180	si:ch211-215l11.5	4.2	2.57E-05	Fast Light-Induced
100003999	LOC100003999	2.2	2.66E-05	Fast Light-Induced
567667	slc12a7b	4.2	2.77E-05	Fast Light-Induced
557799	tmem181	2.3	2.91E-05	Fast Light-Induced
767653	zgc:153394	3.5	3.02E-05	Fast Light-Induced
368775	ivd	7.7	3.09E-05	Fast Light-Induced
568927	reep2	2.8	3.68E-05	Fast Light-Induced
550521	phyh	8	3.76E-05	Fast Light-Induced
140744	ckbb	3.3	3.82E-05	Fast Light-Induced

Table S4: Genes affected by light entrainment.

Gene ID	Symbol	LD Phase	P value *	Light-Entrainment
436969	zgc:92630	5.8	4.13E-05	Fast Light-Induced
394242	dnaja3b	2.7	4.14E-05	Fast Light-Induced
58137	celf1	4	4.14E-05	Fast Light-Induced
567157	acsl3a	3	4.24E-05	Fast Light-Induced
557975	LOC557975	3.5	4.35E-05	Fast Light-Induced
566032	si:ch211-269e2.2	6	4.61E-05	Fast Light-Induced
664766	zgc:123105	3.2	4.76E-05	Fast Light-Induced
100137113	zgc:171474	4.8	4.92E-05	Fast Light-Induced
387592	slc13a1	4.7	4.96E-05	Fast Light-Induced
553244	tom1	5	5.20E-05	Fast Light-Induced
569072	si:dkey-119o24.1	5.3	5.21E-05	Fast Light-Induced
792544	si:dkey-67c22.2	3	5.46E-05	Fast Light-Induced
432385	zgc:111821	7.8	5.49E-05	Fast Light-Induced
393096	lipg	3.8	5.57E-05	Fast Light-Induced
402986	cry-dash	4.3	5.88E-05	Fast Light-Induced
558648	selo	5.7	5.93E-05	Fast Light-Induced
445177	dnajb1a	3.5	6.46E-05	Fast Light-Induced
447937	zgc:101851	7.7	6.78E-05	Fast Light-Induced
619268	sagb	6	7.20E-05	Fast Light-Induced
326964	mg:cb01g09	3.2	7.29E-05	Fast Light-Induced
431765	zgc:77060	5.8	8.07E-05	Fast Light-Induced
406308	grb2	3	8.16E-05	Fast Light-Induced
567414	agpat9l	4.7	8.28E-05	Fast Light-Induced
569958	LOC569958	6.8	8.44E-05	Fast Light-Induced
560210	hsp70l	5.3	8.70E-05	Fast Light-Induced
568862	si:ch73-266o15.4	5.2	8.81E-05	Fast Light-Induced
567762	mycl1b	4.3	8.87E-05	Fast Light-Induced
393444	pygmb	3.2	9.05E-05	Fast Light-Induced
566120	grk7a	2.2	9.10E-05	Fast Light-Induced
402833	camsap1a	2.8	9.17E-05	Fast Light-Induced
30674	tef	3.5	9.51E-05	Fast Light-Induced
334203	zgc:114043	4.7	9.56E-05	Fast Light-Induced
406623	decr2	4.8	9.96E-05	Fast Light-Induced
100332249	LOC100332249	11.7	5.01E-13	Slow Light-Induced
678554	bco2a	8.5	5.37E-11	Slow Light-Induced
406433	dhdhl	11.3	1.11E-10	Slow Light-Induced
553611	six6b	8.5	4.85E-10	Slow Light-Induced
559626	crtac1a	9.5	1.91E-09	Slow Light-Induced
83780	cry2b	12.7	2.17E-09	Slow Light-Induced
494085	pink1	14	2.72E-09	Slow Light-Induced
554067	pcdh2g20	14.7	3.01E-09	Slow Light-Induced

Table S4: Genes affected by light entrainment.

Gene ID	Symbol	LD Phase	P value *	Light-Entrainment
573209	cry2a	12.3	8.55E-09	Slow Light-Induced
447842	zgc:92303	12.8	8.79E-09	Slow Light-Induced
58118	bdnf	10.2	9.38E-09	Slow Light-Induced
751751	zgc:153383	12.5	1.42E-08	Slow Light-Induced
337574	nfatc2ip	10.3	1.82E-08	Slow Light-Induced
100333879	LOC100333879	12.5	1.83E-08	Slow Light-Induced
406625	lgmn	15.5	2.93E-08	Slow Light-Induced
445278	cldn8	9.8	3.28E-08	Slow Light-Induced
407688	sft2d3	13.7	3.89E-08	Slow Light-Induced
324373	zgc:77513	8.7	4.49E-08	Slow Light-Induced
797680	si:dkey-273o13.1	15.7	5.70E-08	Slow Light-Induced
393767	zgc:73336	10.8	7.03E-08	Slow Light-Induced
393283	chd1l	8.8	9.03E-08	Slow Light-Induced
566557	furina	11.5	9.94E-08	Slow Light-Induced
282554	drd3	12.2	1.20E-07	Slow Light-Induced
30517	ahr2	14.2	1.30E-07	Slow Light-Induced
352915	sepw1	9.2	1.72E-07	Slow Light-Induced
404207	cx52.6	9	1.83E-07	Slow Light-Induced
245946	invs	8.8	1.99E-07	Slow Light-Induced
352935	arntl1b	12.2	2.01E-07	Slow Light-Induced
564746	glsb	10.5	2.28E-07	Slow Light-Induced
550490	zgc:110339	8.3	2.84E-07	Slow Light-Induced
791179	si:dkey-19f23.3	10.5	3.43E-07	Slow Light-Induced
352916	sepx1a	9.8	3.52E-07	Slow Light-Induced
259250	trh1	14.7	3.66E-07	Slow Light-Induced
560362	dis3l	12	4.14E-07	Slow Light-Induced
335656	gngt1	12.2	4.56E-07	Slow Light-Induced
550515	crlf3	11.3	5.19E-07	Slow Light-Induced
799219	si:ch211-240l19.7	13.7	5.56E-07	Slow Light-Induced
445091	zgc:92480	8.5	5.85E-07	Slow Light-Induced
541550	ablim1b	8.8	6.05E-07	Slow Light-Induced
368367	si:ch211-284e13.2	14.5	6.39E-07	Slow Light-Induced
30077	rbp4	8.3	6.86E-07	Slow Light-Induced
322152	wu:fb51f10	12.2	8.13E-07	Slow Light-Induced
60635	cldn7b	10.2	8.37E-07	Slow Light-Induced
555304	clasrp	12.2	8.84E-07	Slow Light-Induced
449665	gpsm2l	15.3	8.93E-07	Slow Light-Induced
570286	ftr02	12.3	9.07E-07	Slow Light-Induced
100002010	abcc1	10	9.21E-07	Slow Light-Induced
562478	zufsp	8.8	9.69E-07	Slow Light-Induced
557451	daam1a	13.5	1.01E-06	Slow Light-Induced

Table S4: Genes affected by light entrainment.

Gene ID	Symbol	LD Phase	P value *	Light-Entrainment
492473	zgc:103488	14.7	1.05E-06	Slow Light-Induced
393481	cyp2k6	11.3	1.20E-06	Slow Light-Induced
553745	zgc:113184	9.2	1.25E-06	Slow Light-Induced
565658	sema3e	13	1.40E-06	Slow Light-Induced
570622	gbp2	14.2	1.47E-06	Slow Light-Induced
566858	mov10a	9.7	1.49E-06	Slow Light-Induced
797834	si:dkeyp-110c7.1	14	1.51E-06	Slow Light-Induced
100332121	LOC100332121	13.5	1.67E-06	Slow Light-Induced
393541	tsc22d3	8.8	1.71E-06	Slow Light-Induced
406648	ppt1	14	1.88E-06	Slow Light-Induced
561635	mylk3	12.2	2.07E-06	Slow Light-Induced
569007	LOC569007	8.5	2.13E-06	Slow Light-Induced
394056	zgc:56010	14.5	2.41E-06	Slow Light-Induced
571066	LOC571066	9.7	2.57E-06	Slow Light-Induced
325724	etfa	8.3	2.60E-06	Slow Light-Induced
402892	orai1	15.8	3.05E-06	Slow Light-Induced
445120	cutl1	11.3	3.26E-06	Slow Light-Induced
497449	ghdcl	11.2	3.50E-06	Slow Light-Induced
100137128	hbl4	11.2	3.63E-06	Slow Light-Induced
557454	wu:fk57d06	10	3.91E-06	Slow Light-Induced
324148	wu:fc20c04	15.5	3.95E-06	Slow Light-Induced
559850	neu1	14.5	4.09E-06	Slow Light-Induced
492783	dhrs13	9.8	4.41E-06	Slow Light-Induced
494087	zgc:101722	11.2	4.55E-06	Slow Light-Induced
324501	tmem165	15.2	4.62E-06	Slow Light-Induced
58099	arntl2	14.7	5.17E-06	Slow Light-Induced
405849	foxd1l	12.7	5.39E-06	Slow Light-Induced
406579	dnajc7	15.7	5.49E-06	Slow Light-Induced
327592	micall2b	15.5	5.74E-06	Slow Light-Induced
100331902	zgc:110784	11.8	5.77E-06	Slow Light-Induced
327360	memo1	10	5.80E-06	Slow Light-Induced
767759	zgc:153704	9.7	6.60E-06	Slow Light-Induced
492812	nap1l4b	14	8.01E-06	Slow Light-Induced
550409	ms4a17a.7	12.7	8.02E-06	Slow Light-Induced
449859	si:ch211-251j10.4	13.8	8.13E-06	Slow Light-Induced
553634	casp7	11.8	8.27E-06	Slow Light-Induced
797384	btr29	14.5	8.55E-06	Slow Light-Induced
565154	rspry1	11	8.55E-06	Slow Light-Induced
559258	LOC559258	11.3	8.60E-06	Slow Light-Induced
569467	si:dkey-33i11.9	9.3	8.77E-06	Slow Light-Induced
492507	crh	9.3	9.94E-06	Slow Light-Induced

Table S4: Genes affected by light entrainment.

Gene ID	Symbol	LD Phase	P value *	Light-Entrainment
393249	zgc:56525	12.8	1.03E-05	Slow Light-Induced
394183	grina	14.2	1.03E-05	Slow Light-Induced
791738	zgc:103591	15.3	1.21E-05	Slow Light-Induced
564249	lyve1	10.2	1.25E-05	Slow Light-Induced
735291	hspa12b	11.7	1.26E-05	Slow Light-Induced
571356	si:dkey-24l11.7	13.8	1.27E-05	Slow Light-Induced
797303	tada2a	12.8	1.36E-05	Slow Light-Induced
567578	dcaf11	13.2	1.38E-05	Slow Light-Induced
768291	ppm1h	15.7	1.57E-05	Slow Light-Induced
324109	sp1	12.8	1.65E-05	Slow Light-Induced
402869	mbip	8.2	1.70E-05	Slow Light-Induced
394012	zgc:63474	15.8	1.74E-05	Slow Light-Induced
402935	chm	14.7	1.78E-05	Slow Light-Induced
445495	tmem144a	13.5	1.79E-05	Slow Light-Induced
798401	tex2	13.7	1.85E-05	Slow Light-Induced
550341	dnajc28	9.8	1.93E-05	Slow Light-Induced
324412	fam164a	13	2.15E-05	Slow Light-Induced
100004079	LOC100004079	11.5	2.19E-05	Slow Light-Induced
570476	si:dkeyp-84f11.5	14.2	2.49E-05	Slow Light-Induced
387531	cpvl	11.7	2.64E-05	Slow Light-Induced
553351	clcn7	15	2.65E-05	Slow Light-Induced
436881	rgp1	14.7	2.75E-05	Slow Light-Induced
282677	bckdk	13.8	2.83E-05	Slow Light-Induced
541415	zgc:101577	9.3	2.83E-05	Slow Light-Induced
777614	zgc:152898	12.2	2.91E-05	Slow Light-Induced
386967	stam	14.8	3.05E-05	Slow Light-Induced
553781	zmynd11	13.5	3.11E-05	Slow Light-Induced
724003	zgc:136474	8.2	3.20E-05	Slow Light-Induced
556409	g3bp2	13.5	3.22E-05	Slow Light-Induced
324140	sgk1	8.7	3.37E-05	Slow Light-Induced
504151	im:7155152	15.3	3.48E-05	Slow Light-Induced
337153	zgc:77151	13	3.51E-05	Slow Light-Induced
494533	prkar1aa	14.5	3.56E-05	Slow Light-Induced
30221	psen1	15.5	3.71E-05	Slow Light-Induced
561802	wu:fc76b11	12.7	3.76E-05	Slow Light-Induced
566632	med12	13.8	3.86E-05	Slow Light-Induced
100002272	dusp22b	16	3.94E-05	Slow Light-Induced
352927	clock3	15	3.95E-05	Slow Light-Induced
566811	zgc:152984	11.5	3.97E-05	Slow Light-Induced
568256	zgc:162232	10.5	4.10E-05	Slow Light-Induced
794083	LOC794083	8.8	4.15E-05	Slow Light-Induced

Table S4: Genes affected by light entrainment.

Gene ID	Symbol	LD Phase	P value *	Light-Entrainment
352928	gpx4a	11.7	4.62E-05	Slow Light-Induced
325042	tnika	14.2	4.82E-05	Slow Light-Induced
327277	sec22bb	14.8	4.84E-05	Slow Light-Induced
393164	zgc:56112	14	4.87E-05	Slow Light-Induced
100007897	zgc:162290	14.5	5.64E-05	Slow Light-Induced
100037363	zgc:162228	12.8	5.73E-05	Slow Light-Induced
402949	slc30a6	15.2	5.75E-05	Slow Light-Induced
406578	stxbp2	11.3	5.85E-05	Slow Light-Induced
368775	ivd	8.7	5.92E-05	Slow Light-Induced
492523	def8	14.7	6.03E-05	Slow Light-Induced
436640	zgc:92090	11.5	6.09E-05	Slow Light-Induced
777759	si:dkey-228a15.3	9.3	6.28E-05	Slow Light-Induced
436886	nrbf2	13.7	6.49E-05	Slow Light-Induced
368704	ctsc	15.3	6.49E-05	Slow Light-Induced
100002945	zgc:110779	14.2	6.60E-05	Slow Light-Induced
393287	chuk	14	6.74E-05	Slow Light-Induced
378456	cmt4b2	13	6.87E-05	Slow Light-Induced
406645	ctsba	15.5	7.10E-05	Slow Light-Induced
664760	tbce	15.5	7.32E-05	Slow Light-Induced
436626	dnajb6a	13.2	7.45E-05	Slow Light-Induced
565588	ctsf	14.3	7.57E-05	Slow Light-Induced
562682	si:dkey-251i10.2	9.2	7.58E-05	Slow Light-Induced
557337	prkd3	8.7	7.76E-05	Slow Light-Induced
557682	zgc:154015	15.7	7.88E-05	Slow Light-Induced
573382	mns1	13.2	8.07E-05	Slow Light-Induced
335650	rcv1	9.2	8.13E-05	Slow Light-Induced
394052	ftcd	10.8	8.44E-05	Slow Light-Induced
378969	rab32a	12.5	8.49E-05	Slow Light-Induced
553363	fto	15	8.75E-05	Slow Light-Induced
558558	zgc:153606	14.7	8.77E-05	Slow Light-Induced
100000104	zgc:162946	13.8	8.87E-05	Slow Light-Induced
723997	mgst2	14.8	9.12E-05	Slow Light-Induced
405774	uts2a	10.2	9.17E-05	Slow Light-Induced
338306	impdh1b	15	9.23E-05	Slow Light-Induced
394085	zgc:66323	9	9.35E-05	Slow Light-Induced
564649	LOC564649	12.3	9.41E-05	Slow Light-Induced
550287	psmd4b	15.7	9.53E-05	Slow Light-Induced
100000303	zplxdc2	14.2	9.60E-05	Slow Light-Induced
100037388	zgc:162420	14	9.65E-05	Slow Light-Induced
336426	chmp1b	15.8	9.69E-05	Slow Light-Induced
436961	cdc34b	12.8	9.81E-05	Slow Light-Induced

Table S4: Genes affected by light entrainment.

Gene ID	Symbol	LD Phase	P value *	Light-Entrainment
140423	jag1b	18	5.60E-09	Dark-Lnduced
799423	si:ch211-191i18.3	1.3	2.01E-07	Dark-Lnduced
446119	srp68	23.3	2.16E-07	Dark-Lnduced
568985	slc6a1	21.3	6.15E-07	Dark-Lnduced
100003274	rab38	22.7	7.36E-07	Dark-Lnduced
266599	id2a	16.7	8.08E-07	Dark-Lnduced
561606	atoh8	17.8	1.47E-06	Dark-Lnduced
431721	mrrf	1.3	1.50E-06	Dark-Lnduced
503564	drd4a	18	1.78E-06	Dark-Lnduced
791773	gpr39	20.7	2.63E-06	Dark-Lnduced
678556	cldn15b	17	2.80E-06	Dark-Lnduced
553574	pin4	1.7	2.81E-06	Dark-Lnduced
326033	zgc:56622	20.5	3.26E-06	Dark-Lnduced
570312	slc24a5	23.2	3.26E-06	Dark-Lnduced
768157	cplx4a	22.5	3.59E-06	Dark-Lnduced
100331574	LOC100331574	22.2	5.97E-06	Dark-Lnduced
100149301	LOC100149301	21.2	6.70E-06	Dark-Lnduced
568102	LOC568102	1.3	7.45E-06	Dark-Lnduced
393761	rdh8l	21.8	7.84E-06	Dark-Lnduced
336034	wu:fj44c04	0.7	8.56E-06	Dark-Lnduced
557648	mapkapk2b	21.8	9.87E-06	Dark-Lnduced
406599	atp5j	1.2	9.95E-06	Dark-Lnduced
368722	pdzk1ip1l	18	1.21E-05	Dark-Lnduced
569894	LOC569894	0.3	1.63E-05	Dark-Lnduced
100002108	npvf	1.7	1.68E-05	Dark-Lnduced
559618	LOC559618	23.7	1.76E-05	Dark-Lnduced
393815	zgc:65890	20.5	1.99E-05	Dark-Lnduced
259258	p2rx4a	18.2	2.33E-05	Dark-Lnduced
100322009	si:ch1073-66c17.4	1.3	2.39E-05	Dark-Lnduced
368863	fkbp3	18.8	2.42E-05	Dark-Lnduced
436850	suclg1	1.7	2.47E-05	Dark-Lnduced
327079	sap30l	18.7	2.52E-05	Dark-Lnduced
30080	mitfa	20.7	2.60E-05	Dark-Lnduced
245703	atp1a1a.5	17.8	2.86E-05	Dark-Lnduced
571626	zgc:158742	16.3	2.92E-05	Dark-Lnduced
334465	si:dkey-18f23.10	19	3.23E-05	Dark-Lnduced
322462	sult6b1	0.8	3.54E-05	Dark-Lnduced
100003734	fastkd3	16.8	3.88E-05	Dark-Lnduced
100007318	LOC100007318	18.5	4.40E-05	Dark-Lnduced
798921	si:ch211-217k17.9	1.7	5.41E-05	Dark-Lnduced
393515	stx3a	16.8	5.76E-05	Dark-Lnduced

Table S4: Genes affected by light entrainment.

Gene ID	Symbol	LD Phase	P value *	Light-Entrainment
402976	si:dkey-194e6.1	22.8	5.88E-05	Dark-Lnduced
58076	gata6	20.5	6.02E-05	Dark-Lnduced
327379	calm3a	0.2	6.57E-05	Dark-Lnduced
393677	aanat1	19.3	7.09E-05	Dark-Lnduced
58048	hoxa9b	16.5	7.98E-05	Dark-Lnduced
550415	ap1s3a	0.3	8.39E-05	Dark-Lnduced
436650	hip1r	16.5	8.77E-05	Dark-Lnduced
405875	zgc:85932	22.3	9.52E-05	Dark-Lnduced
570276	vsg1	20	9.84E-05	Dark-Lnduced

*** We fit the joint LD/DD gene expression values to a set of cosine curves with 24h periods of shifting phases in LD but constant levels at either +1 (elevated troughs) or -1 (decreased peaks) in DD. P value less than 0.0001 was used in the fit of joint LD/DD expression value.**
