# Supplementary material for: Analysis of a Gene Regulatory Cascade Mediating Circadian Rhythm in Zebrafish
Source: PLoS Comput Biol. 2013 Feb 28;9(2):e1002940. doi: 10.1371/journal.pcbi.1002940 (PMC3585402; doi:10.1371/journal.pcbi.1002940)
Supplement: Table S5 — Primers used for real-time PCR. (PDF) [file pcbi.1002940.s010.pdf]

Table S5: Primers used for real-time PCR.

Gene Symbol	Forward	Reverse	Product Size	Ensemble ID
aanat1	GGACCAGGACCGTCTGACT	CTGCAAGTAACGCCACAAGA	199bp	ENSDART00000053126
cry2a	CGGGCCACAACAGTAGATTT	TCTCTCCCTCTTCCCTGTCA	158bp	ENSDART00000129210
per2	CTCTGGACGGCAGTGAGAAT	CACAGCACCTTCTGGATGTC	190bp	ENSDART00000039735
gapdh	TGCTGGTATTGCTCTCAACG	GCCATCAGGTCACATACACG	153bp	ENSDART00000063800
per3	CTCGTCCTCCACCTCAGAAG	CTGAACCTGCAGACACCTGA	173bp	ENSDART00000024304
bhlhe40	TGCCCGAACACCTTAAACTC	CCAAGACAACAGCCTTCTCC	117bp	ENSDART00000026017
bhlhe41	TGACGCTGAAGCATTTGAAC	GCGTCTAAGTCAGCCTGGAG	178bp	ENSDART00000061106
smad1	GCTCTTGGCACAGTCAGTCA	ACATCCTGCCGATGGTACTC	177bp	ENSDART00000125043
smad3a	ACCAAACCCTGTGTCTCCTG	GCTGTGAGGCATGGAAAGTT	194bp	ENSDART00000045374
ppargc1b	TGTCCTGTTCACCTCCTTCC	TCCATGACACGTCTCTGAGC	131bp	ENSDART00000079080
cdx1b	GAAAAGCAGAACTGGCAACC	GAGGCTGCTGCATTTTCTTC	174bp	ENSDART00000081993
crx	ACCTTCCCGAGTCCAGAGTT	GCATTTAGCACGACGGTTCT	113bp	ENSDART00000037879
myog	GCCTTGGAGGGCTTAATTTC	GAATCAGCCTTCCTGACTGC	136bp	ENSDART00000014062
hnf1a	ATTGCCCCAAGCTCCTTTAT	TACTGCTGTCTGCGATCACC	187bp	ENSDART00000018862
maf	CAGCAGAGGCACATACTGGA	CGATGAGTTTTCTCGGAAGC	153bp	ENSDART00000014703
pax6a	CTGACGTTTTTGCACGAGAA	AACTTTTCCTCCCTCCTCCA	169bp	ENSDART00000148420
mef2a	GGCTCTCCAGGGCTCTCTAT	CATTCTGGCTGGTGTTGATG	196bp	ENSDART00000148106
clock	AGACCACCAACCTCAACCAG	CTGTGTCGGCTGAGAGATCA	173bp	ENSDART00000025575
mitfa	GCAGCAGAAAGCAAAAGAGC	GGCTGGAAGAAGCTACAACG	194bp	ENSDART00000056457
