# Supplementary material for: Analysis of a Gene Regulatory Cascade Mediating Circadian Rhythm in Zebrafish
Source: PLoS Comput Biol. 2013 Feb 28;9(2):e1002940. doi: 10.1371/journal.pcbi.1002940 (PMC3585402; doi:10.1371/journal.pcbi.1002940)
Supplement: Table S6 — Transcription factors in zebrafish circadian oscillating genes. (PDF) [file pcbi.1002940.s011.pdf]

Table S6: Transcription factors in zebrafish circadian oscillating genes.

TF	TRANSFAC MOTIF	Circadian Phase
AHR1A	AhR_HIF	2.833
AHR1A	AhR	2.833
AR	AR	5.167
ARID2	NA	20.000
ARID3B	NA	14.875
ARNTL1A	EBOX_CLOCK	13.420
ARNTL1B	EBOX_CLOCK	12.790
ARNTL2	EBOX_CLOCK	16.290
ATF4B1	CRE	15.750
ATF6	CRE	2.583
BHLHE40	Ebox_DEC	5.250
BHLHE41	Ebox_DEC	0.542
BLZF1	NA	15.833
BMI1	NA	16.792
BRD8	NA	14.583
CALCOCO1	NA	15.125
CBX3B	NA	6.500
CBX4	NA	16.917
CDX1A	CDX	15.833
CDX1B	CDX	14.330
CDX4	NA	20.667
CEBPA	C/EBP	22.417
CHD2	NA	16.083
CLOCK	EBOX_CLOCK	15.080
CLOCK3	EBOX_CLOCK	15.080
CREB3L3	CRE	7.000
CRSP6	NA	16.417
CRX	PRD/Crx	16.625
DCP1A	NA	14.583
DLX3B	NA	19.417
DRG1	NA	0.750
ELL2	NA	3.042
EPAS1A	NA	3.542
ERCC2	NA	17.333
ETV5B	NA	6.500
FLI1B	ETS	14.167
FOXA1	HNF3	14.670
FOXK2	NA	18.750
FOXO3B	FOXO	12.420
FUBP1	NA	18.833
FUS	NA	15.042
GATAD2A	NA	19.000
GLI1	NA	14.417
GTF2A1	TFIIA	3.920
GTF3AA	NA	0.500
HBP1	NA	12.500
HIF1AB	AhR_HIF	23.420
HIF1AB	HIF	23.420
HMGB3B	NA	3.667
HMX1	NA	15.333
HNF1A	HNF1	4.417
HOXA10B	NA	16.250
HOXA11B	NA	19.917
HOXA2B	NA	17.500
HOXA4A	HOX1/HOXA	16.917

Table S6: Transcription factors in zebrafish circadian oscillating genes.

TF	TRANSFAC MOTIF	Circadian Phase
HOXB1A	NA	16.500
HOXB1B	NA	16.500
HOXB2A	NA	16.167
HOXC11A	NA	21.708
HSF2	HSF	3.750
HTATIP2	NA	8.500
ID2A	NA	17.292
ID4	NA	15.417
KCNIP3	NA	3.083
KLF2B	NA	13.500
LBX1A	NA	14.250
LMO1	NA	15.750
LRRFIP1A	NA	4.125
LZTR1	NA	14.417
MAF	MAF	1.500
MAX	Ebox_general	22.880
MAX	Ebox_MycMax	22.880
MBD1	NA	16.417
MECOM	Evi	12.583
MED12	NA	15.500
MEF2A	MEF2	1.500
MEF2A	RSRFC4	1.500
MITFA	Ebox_TFE	21.420
MSRB2	NA	14.833
MXD3	Ebox_general	12.670
MYCB	Ebox_general	4.000
MYCL1B	NA	5.833
MYOG	Ebox_E2A_MyoD	10.625
NFE2L1	MAF	9.500
NFE2L2	MAF	7.080
NFIL3	DBOX	14.420
NFYBB	NFY	16.920
NR1D1	RRE	23.750
NR1D4A	RRE	19.830
NR1D4B	RRE	19.750
NR3C1	GRE	13.710
NR4A1	NA	2.000
NRBF2	NA	14.333
NRIP1B	NA	12.417
PAX6A	PAX6	15.500
PAX6A	PAX	15.500
PBXIP1A	NA	1.167
PER1A	NA	1.042
PER1B	NA	3.333
PER2	NA	7.500
PER3	NA	4.167
PIAS2	NA	15.250
PIR	NA	16.417
POLR2EB	NA	1.667
POLR3C	NA	0.167
PPARGC1B	PPAR	22.170
PRRX1A	NA	17.417
PTF1A	NA	19.583
RARAA	DR4	12.167
RNF2	NA	15.083

Table S6: Transcription factors in zebrafish circadian oscillating genes.

TF	TRANSFAC MOTIF	Circadian Phase
RORAB	RRE	7.500
RORCA	NA	10.750
RORCB	NA	12.417
SI:CH211-199M3.9	CRE	2.580
SIX2A	NA	17.333
SIX3A	NA	17.417
SLA2	NA	16.917
SMAD1	SMAD	20.333
SMAD3A	SMAD	4.417
SMYD1A	NA	17.208
SNAPC3	NA	0.667
STAT6	NA	12.417
TAF1	NA	16.250
TAF12	NA	15.500
TAF7	NA	17.500
TBPL1	NA	16.000
TBX1	NA	15.750
TCF12	Ebox_HEB	13.750
TCF12	Ebox_general	13.750
TCF12	Ebox_E2A_MyoD	13.750
TCF25	NA	0.083
TCF3	NA	12.917
TEF	DBOX	3.417
TFB2M	NA	19.333
TFCP2L1	CP2/LSF	14.667
TGIF1	TGIF	13.667
TOX	NA	18.000
TSC22D3	NA	8.583
UHRF1	NA	4.250
USF1	Ebox_USF	13.167
VSX1	NA	15.833
XBP1	XBP1	2.917
YY1A	YY1	12.667
YY1B	NA	16.708
ZBTB16A	NA	15.250
ZDHHC3	NA	4.417
ZFP36L2	NA	7.917
ZNRD1	NA	1.250

TF: gene symbol of the transcription factor.
MOTIF: position weight matrices corresponding to the transcription factor.
circadian_phase:circadian phase of the transcription factor.
