# Supplementary material for: Analysis of a Gene Regulatory Cascade Mediating Circadian Rhythm in Zebrafish
Source: PLoS Comput Biol. 2013 Feb 28;9(2):e1002940. doi: 10.1371/journal.pcbi.1002940 (PMC3585402; doi:10.1371/journal.pcbi.1002940)
Supplement: Table S7 — Circadian phase of transcription factor and their targets. (PDF) [file pcbi.1002940.s012.pdf]

Table S7: Circadian phase of transcription factor and their targets.

TF	TF PK	TRANSFAC MOTIF	Target PK	P value	Odd Ratio
YY1A	12.67	YY1_Q6_02	22.68	7.78E-10	3.50
NFIL3	14.42	E4BP4_01	3.96	1.08E-08	3.17
TEF	3.42	E4BP4_01	3.96	1.08E-08	3.17
MAX	22.88	MYC_Q2	23.81	2.05E-08	3.31
PPARGC1B	22.17	PPARG_03	3.82	1.81E-07	3.26
YY1A	12.67	YY1_02	0.65	5.95E-07	3.00
ARNTL1A	13.42	CLOCKBMAL_Q6	23.52	1.56E-06	2.53
ARNTL1B	12.79	CLOCKBMAL_Q6	23.52	1.56E-06	2.53
ARNTL2	16.29	CLOCKBMAL_Q6	23.52	1.56E-06	2.53
CLOCK	15.08	CLOCKBMAL_Q6	23.52	1.56E-06	2.53
CLOCK3	15.08	CLOCKBMAL_Q6	23.52	1.56E-06	2.53
NFIL3	14.42	HLF_01	4.63	6.85E-06	2.80
TEF	3.42	HLF_01	4.63	6.85E-06	2.80
MAX	22.88	NMYC_01	0.77	9.19E-06	2.47
USF1	13.17	USF_02	17.14	1.85E-05	2.65
FOXO3B	12.42	FOXO1_02	15.89	2.35E-05	2.70
YY1A	12.67	YY1_Q6	0.10	3.31E-05	2.25
ATF4B1	15.75	CREBP1_01	3.72	3.95E-05	2.49
ATF6	2.58	CREBP1_01	3.72	3.95E-05	2.49
CREB3L3	7.00	CREBP1_01	3.72	3.95E-05	2.49
SI:CH211-199M3.9	2.58	CREBP1_01	3.72	3.95E-05	2.49
PPARGC1B	22.17	PPAR_DR1_Q2	3.82	4.63E-05	2.37
NR1D1	23.75	RORA2_01	12.43	5.41E-05	3.11
NR1D4A	19.83	RORA2_01	12.43	5.41E-05	3.11
NR1D4B	19.75	RORA2_01	12.43	5.41E-05	3.11
RORAB	7.50	RORA2_01	12.43	5.41E-05	3.11
FOXO3B	12.42	FOXO4_02	12.77	8.52E-05	3.01
TCF12	13.75	HEB_Q6	12.84	1.41E-04	3.56
USF1	13.17	USF_C	23.52	1.42E-04	2.58
PPARGC1B	22.17	PPARA_01	5.02	2.10E-04	2.77
NR1D1	23.75	RORA1_01	21.22	2.17E-04	3.47
NR1D4A	19.83	RORA1_01	21.22	2.17E-04	3.47
NR1D4B	19.75	RORA1_01	21.22	2.17E-04	3.47
RORAB	7.50	RORA1_01	21.22	2.17E-04	3.47
MYOG	10.63	E47_01	16.10	2.40E-04	3.37
TCF12	13.75	E47_01	16.10	2.40E-04	3.37
HNF1A	4.42	HNF1_C	5.14	2.87E-04	2.67
MECOM	12.58	EVI1_04	9.96	2.95E-04	8.11
AR	5.17	AR_Q2	4.34	2.96E-04	3.09
CEBPA	22.42	CEBP_Q2_01	5.98	3.64E-04	3.24
MYOG	10.63	E2A_Q6	20.81	5.22E-04	5.70
TCF12	13.75	E2A_Q6	20.81	5.22E-04	5.70
ATF4B1	15.75	CREB_01	0.67	6.24E-04	1.83
ATF6	2.58	CREB_01	0.67	6.24E-04	1.83
CREB3L3	7.00	CREB_01	0.67	6.24E-04	1.83
SI:CH211-199M3.9	2.58	CREB_01	0.67	6.24E-04	1.83
PPARGC1B	22.17	PPARG_01	5.26	6.30E-04	2.17
ATF4B1	15.75	ATF1_Q6	20.86	6.36E-04	2.69
ATF6	2.58	ATF1_Q6	20.86	6.36E-04	2.69
CREB3L3	7.00	ATF1_Q6	20.86	6.36E-04	2.69
SI:CH211-199M3.9	2.58	ATF1_Q6	20.86	6.36E-04	2.69
AR	5.17	AR_02	10.68	7.15E-04	3.96
NFYBB	16.92	NFY_C	4.75	9.31E-04	2.01
CEBPA	22.42	CEBPDELTA_Q6	14.35	9.75E-04	3.91
NFYBB	16.92	NFY_01	7.44	1.11E-03	2.21

Table S7: Circadian phase of transcription factor and their targets.

TF	TF PK	TRANSFAC MOTIF	Target PK	P value	Odd Ratio
ARNTL1A	13.42	ARNT_02	17.88	1.14E-03	1.92
ARNTL1B	12.79	ARNT_02	17.88	1.14E-03	1.92
ARNTL2	16.29	ARNT_02	17.88	1.14E-03	1.92
CLOCK	15.08	ARNT_02	17.88	1.14E-03	1.92
CLOCK3	15.08	ARNT_02	17.88	1.14E-03	1.92
CEBPA	22.42	CEBPGAMMA_Q6	9.05	1.18E-03	6.01
SMAD1	20.33	SMAD3_Q6	15.65	1.21E-03	2.90
SMAD3A	4.42	SMAD3_Q6	15.65	1.21E-03	2.90
MYOG	10.63	MYOD_Q6	17.26	1.31E-03	4.06
TCF12	13.75	MYOD_Q6	17.26	1.31E-03	4.06
ATF4B1	15.75	ATF6_01	22.34	1.64E-03	2.66
ATF6	2.58	ATF6_01	22.34	1.64E-03	2.66
CREB3L3	7.00	ATF6_01	22.34	1.64E-03	2.66
SI:CH211-199M3.9	2.58	ATF6_01	22.34	1.64E-03	2.66
NFIL3	14.42	TEF_Q6	6.14	1.68E-03	2.92
TEF	3.42	TEF_Q6	6.14	1.68E-03	2.92
ATF4B1	15.75	CREB_Q4_01	0.43	1.83E-03	1.79
ATF6	2.58	CREB_Q4_01	0.43	1.83E-03	1.79
CREB3L3	7.00	CREB_Q4_01	0.43	1.83E-03	1.79
SI:CH211-199M3.9	2.58	CREB_Q4_01	0.43	1.83E-03	1.79
USF1	13.17	USF_01	18.17	1.90E-03	1.89
FOXO3B	12.42	FOXO3_01	14.02	1.94E-03	2.18
ATF4B1	15.75	CREB_02	0.43	2.12E-03	2.01
ATF6	2.58	CREB_02	0.43	2.12E-03	2.01
CREB3L3	7.00	CREB_02	0.43	2.12E-03	2.01
SI:CH211-199M3.9	2.58	CREB_02	0.43	2.12E-03	2.01
ATF4B1	15.75	ATF_B	0.43	2.44E-03	1.83
ATF6	2.58	ATF_B	0.43	2.44E-03	1.83
CREB3L3	7.00	ATF_B	0.43	2.44E-03	1.83
SI:CH211-199M3.9	2.58	ATF_B	0.43	2.44E-03	1.83
ATF4B1	15.75	CREB_Q2	2.09	2.66E-03	1.77
ATF6	2.58	CREB_Q2	2.09	2.66E-03	1.77
CREB3L3	7.00	CREB_Q2	2.09	2.66E-03	1.77
SI:CH211-199M3.9	2.58	CREB_Q2	2.09	2.66E-03	1.77
MYOG	10.63	MYOD_01	18.05	2.73E-03	2.55
TCF12	13.75	MYOD_01	18.05	2.73E-03	2.55
FOXO3B	12.42	FREAC2_01	11.09	3.60E-03	3.35
FOXO3B	12.42	FOXP3_Q4	11.42	3.82E-03	3.35
CDX1A	15.83	CDX2_Q5	7.06	3.86E-03	3.39
CDX1B	14.33	CDX2_Q5	7.06	3.86E-03	3.39
MAF	1.50	NFE2_01	13.51	3.98E-03	2.25
NFE2L1	9.50	NFE2_01	13.51	3.98E-03	2.25
NFE2L2	7.08	NFE2_01	13.51	3.98E-03	2.25
MECOM	12.58	EVI1_05	5.98	5.02E-03	2.59
MAX	22.88	MYCMAX_B	18.05	5.15E-03	2.20
MAF	1.50	MAF_Q6_01	14.09	5.20E-03	2.18
NFE2L1	9.50	MAF_Q6_01	14.09	5.20E-03	2.18
NFE2L2	7.08	MAF_Q6_01	14.09	5.20E-03	2.18
MAX	22.88	MYCMAX_03	23.69	5.25E-03	1.70
MYOG	10.63	MYOD_Q6_01	14.69	5.42E-03	2.39
TCF12	13.75	MYOD_Q6_01	14.69	5.42E-03	2.39
FOXA1	14.67	HNF3ALPHA_Q6	11.30	5.65E-03	4.36
MITFA	21.42	TFE_Q6	18.55	5.77E-03	2.46
MYOG	10.63	E12_Q6	14.93	5.96E-03	2.52
TCF12	13.75	E12_Q6	14.93	5.96E-03	2.52

Table S7: Circadian phase of transcription factor and their targets.

TF	TF PK	TRANSFAC MOTIF	Target PK	P value	Odd Ratio
FOXO3B	12.42	FOXD3_01	8.59	6.68E-03	4.88
ATF4B1	15.75	CREB_Q2_01	1.51	7.01E-03	1.70
ATF6	2.58	CREB_Q2_01	1.51	7.01E-03	1.70
CREB3L3	7.00	CREB_Q2_01	1.51	7.01E-03	1.70
SI:CH211-199M3.9	2.58	CREB_Q2_01	1.51	7.01E-03	1.70
ATF4B1	15.75	CREBATF_Q6	22.63	7.18E-03	1.79
ATF6	2.58	CREBATF_Q6	22.63	7.18E-03	1.79
CREB3L3	7.00	CREBATF_Q6	22.63	7.18E-03	1.79
SI:CH211-199M3.9	2.58	CREBATF_Q6	22.63	7.18E-03	1.79
HSF2	3.75	HSF1_Q6	3.96	7.62E-03	2.12
MAF	1.50	MAF_Q6	14.06	7.78E-03	2.60
NFE2L1	9.50	MAF_Q6	14.06	7.78E-03	2.60
NFE2L2	7.08	MAF_Q6	14.06	7.78E-03	2.60
HNF1A	4.42	HNF1_Q6_01	4.44	7.93E-03	2.02
MEF2A	1.50	MEF2_Q6_01	2.06	7.94E-03	2.70
CEBPA	22.42	CEBP_Q2	21.02	8.05E-03	2.78
SMAD1	20.33	SMAD4_Q6	21.72	8.08E-03	3.03
SMAD3A	4.42	SMAD4_Q6	21.72	8.08E-03	3.03
NFYBB	16.92	NFY_Q6	2.54	8.33E-03	1.64
XBP1	2.92	XBP1_01	23.42	8.56E-03	2.14
NR1D1	23.75	RORA_Q4	14.42	8.97E-03	2.08
NR1D4A	19.83	RORA_Q4	14.42	8.97E-03	2.08
NR1D4B	19.75	RORA_Q4	14.42	8.97E-03	2.08
RORAB	7.50	RORA_Q4	14.42	8.97E-03	2.08
FLI1B	14.17	NERF_Q2	11.52	9.62E-03	3.07
MECOM	12.58	EVI1_06	21.10	9.69E-03	2.89
PAX6A	15.50	PAX6_01	4.13	9.95E-03	2.59
NFYBB	16.92	ALPHACP1_01	7.85	1.03E-02	2.24
MAX	22.88	MYCMAX_01	2.88	1.07E-02	1.82
FLI1B	14.17	ETS_Q4	14.30	1.11E-02	2.28
USF1	13.17	USF_Q6_01	20.88	1.13E-02	2.20
ATF4B1	15.75	CREB_Q4	2.09	1.20E-02	1.56
ATF6	2.58	CREB_Q4	2.09	1.20E-02	1.56
CREB3L3	7.00	CREB_Q4	2.09	1.20E-02	1.56
SI:CH211-199M3.9	2.58	CREB_Q4	2.09	1.20E-02	1.56
CDX1A	15.83	CDXA_01	2.88	1.24E-02	2.59
CDX1B	14.33	CDXA_01	2.88	1.24E-02	2.59
MEF2A	1.50	MEF2_01	9.96	1.24E-02	4.05
MEF2A	1.50	RSRFC4_01	10.18	1.25E-02	3.48
HIF1AB	23.42	HIF1_Q3	21.38	1.32E-02	2.30
PAX6A	15.50	PAX_Q6	9.96	1.37E-02	3.94
NFYBB	16.92	NFY_Q6_01	4.75	1.39E-02	1.62
MAX	22.88	MYCMAX_02	23.52	1.40E-02	1.94
MEF2A	1.50	AMEF2_Q6	3.17	1.40E-02	1.98
PPARGC1B	22.17	PPARG_02	20.09	1.41E-02	2.56
MAF	1.50	NRF1_Q6	13.75	1.45E-02	1.59
NFE2L1	9.50	NRF1_Q6	13.75	1.45E-02	1.59
NFE2L2	7.08	NRF1_Q6	13.75	1.45E-02	1.59
NR3C1	13.71	GR_01	21.34	1.47E-02	2.67
FOXO3B	12.42	XFD2_01	3.17	1.53E-02	2.14
GTF2A1	3.92	TFIIA_Q6	4.51	1.54E-02	2.17
FOXO3B	12.42	HFH1_01	13.10	1.58E-02	2.05
MAX	22.88	MAX_01	23.52	1.58E-02	1.69
ATF4B1	15.75	CREBP1CJUN_01	19.68	1.58E-02	1.95
ATF6	2.58	CREBP1CJUN_01	19.68	1.58E-02	1.95

Table S7: Circadian phase of transcription factor and their targets.

TF	TF PK	TRANSFAC MOTIF	Target PK	P value	Odd Ratio
CREB3L3	7.00	CREBP1CJUN_01	19.68	1.58E-02	1.95
SI:CH211-199M3.9	2.58	CREBP1CJUN_01	19.68	1.58E-02	1.95
MECOM	12.58	EVI1_03	5.98	1.60E-02	2.33
MECOM	12.58	EVI1_02	10.75	1.64E-02	2.95
NR3C1	13.71	GR_Q6	17.06	1.67E-02	2.20
ATF4B1	15.75	ATF3_Q6	19.92	1.70E-02	1.98
ATF6	2.58	ATF3_Q6	19.92	1.70E-02	1.98
CREB3L3	7.00	ATF3_Q6	19.92	1.70E-02	1.98
SI:CH211-199M3.9	2.58	ATF3_Q6	19.92	1.70E-02	1.98
MAF	1.50	TCF11MAFG_01	3.48	1.78E-02	2.05
NFE2L1	9.50	TCF11MAFG_01	3.48	1.78E-02	2.05
NFE2L2	7.08	TCF11MAFG_01	3.48	1.78E-02	2.05
FOXO3B	12.42	FOXJ2_01	9.94	1.79E-02	4.51
FLI1B	14.17	ELF1_Q6	21.10	1.80E-02	2.55
NFIL3	14.42	DBP_Q6	15.43	1.81E-02	2.81
TEF	3.42	DBP_Q6	15.43	1.81E-02	2.81
USF1	13.17	USF_Q6	15.77	1.82E-02	1.69
CEBPA	22.42	CEBP_C	16.92	2.07E-02	1.95
FLI1B	14.17	ETS2_B	16.06	2.10E-02	2.05
MEF2A	1.50	MMEF2_Q6	9.05	2.11E-02	3.50
FLI1B	14.17	ETS_Q6	7.27	2.11E-02	3.15
FLI1B	14.17	ELK1_02	1.92	2.13E-02	1.71
SMAD1	20.33	SMAD_Q6	17.26	2.33E-02	4.02
SMAD3A	4.42	SMAD_Q6	17.26	2.33E-02	4.02
HSF2	3.75	HSF2_01	2.98	2.37E-02	1.97
BHLHE40	5.25	DEC_Q1	12.43	2.41E-02	2.26
BHLHE41	0.54	DEC_Q1	12.43	2.41E-02	2.26
MAX	22.88	EBOX_Q6_01	15.55	2.62E-02	1.83
MXD3	12.67	EBOX_Q6_01	15.55	2.62E-02	1.83
MYCB	4.00	EBOX_Q6_01	15.55	2.62E-02	1.83
TCF12	13.75	EBOX_Q6_01	15.55	2.62E-02	1.83
FLI1B	14.17	PEA3_Q6	14.69	2.64E-02	2.73
NR3C1	13.71	GRE_C	20.35	2.66E-02	2.51
CEBPA	22.42	CEBP_Q3	5.54	2.76E-02	2.38
SMAD1	20.33	SMAD_Q6_01	17.18	2.79E-02	2.17
SMAD3A	4.42	SMAD_Q6_01	17.18	2.79E-02	2.17
PPARGC1B	22.17	PPARA_02	9.96	2.82E-02	3.85
HOXA4A	16.92	HOXA3_01	17.98	2.83E-02	2.13
FOXO3B	12.42	FOXM1_01	17.69	2.84E-02	2.47
AHR1A	2.83	AHR_01	19.27	2.92E-02	2.19
MAF	1.50	NRF2_01	23.30	3.04E-02	1.74
NFE2L1	9.50	NRF2_01	23.30	3.04E-02	1.74
NFE2L2	7.08	NRF2_01	23.30	3.04E-02	1.74
HIF1AB	23.42	HIF1_Q5	22.44	3.10E-02	2.04
MEF2A	1.50	HMEF2_Q6	2.54	3.11E-02	1.95
MEF2A	1.50	RSRFC4_Q2	22.68	3.12E-02	2.16
MAF	1.50	NRF2_Q4	18.55	3.19E-02	1.93
NFE2L1	9.50	NRF2_Q4	18.55	3.19E-02	1.93
NFE2L2	7.08	NRF2_Q4	18.55	3.19E-02	1.93
AR	5.17	AR_01	20.26	3.20E-02	2.22
MEF2A	1.50	MEF2_02	17.26	3.66E-02	1.91
FOXO3B	12.42	FREAC3_01	14.93	3.70E-02	1.88
MECOM	12.58	EVI1_01	4.34	3.80E-02	1.99
TFCP2L1	14.67	CP2_01	11.52	3.87E-02	2.68
CEBPA	22.42	CEBPA_01	6.05	3.92E-02	2.21

Table S7: Circadian phase of transcription factor and their targets.

TF	TF PK	TRANSFAC MOTIF	Target PK	P value	Odd Ratio
MYOG	10.63	MYOGENIN_Q6	13.27	4.15E-02	3.06
TCF12	13.75	MYOGENIN_Q6	13.27	4.15E-02	3.06
FOXO3B	12.42	HFH3_01	21.89	4.21E-02	2.47
MAF	1.50	CMAF_01	6.50	4.53E-02	2.07
NFE2L1	9.50	CMAF_01	6.50	4.53E-02	2.07
NFE2L2	7.08	CMAF_01	6.50	4.53E-02	2.07
MYOG	10.63	E47_02	19.46	4.61E-02	2.52
TCF12	13.75	E47_02	19.46	4.61E-02	2.52
AHR1A	2.83	AHRHIF_Q6	3.50	4.64E-02	2.43
HIF1AB	23.42	AHRHIF_Q6	3.50	4.64E-02	2.43
YY1A	12.67	YY1_01	23.23	4.74E-02	2.09
RARAA	12.17	DR4_Q2	19.46	4.88E-02	2.02
FOXO3B	12.42	FOXO3A_Q1	3.82	4.98E-02	2.15
FOXO3B	12.42	FOXO4_01	14.42	5.14E-02	1.99
ATF4B1	15.75	ATF_01	19.46	5.29E-02	1.73
ATF6	2.58	ATF_01	19.46	5.29E-02	1.73
CREB3L3	7.00	ATF_01	19.46	5.29E-02	1.73
SI:CH211-199M3.9	2.58	ATF_01	19.46	5.29E-02	1.73
HNF1A	4.42	HNF1_Q6	6.60	5.33E-02	1.85
AHR1A	2.83	AHR_Q5	5.23	5.57E-02	2.04
HNF1A	4.42	HNF1_01	20.18	5.72E-02	1.81
AR	5.17	AR_03	1.90	5.80E-02	1.79
FOXO3B	12.42	XFD1_01	14.64	5.89E-02	1.73
FOXA1	14.67	HNF3_Q6	10.44	5.95E-02	2.98
TFCP2L1	14.67	CP2_02	18.77	5.95E-02	2.27
FLI1B	14.17	CETS168_Q6	20.26	6.05E-02	2.19
CRX	16.63	CRX_Q4	14.18	6.09E-02	1.84
FOXO3B	12.42	FOXJ2_02	2.18	6.21E-02	1.72
HSF2	3.75	HSF1_01	2.98	6.24E-02	1.80
FOXO3B	12.42	FREAC4_01	15.72	6.37E-02	1.59
MEF2A	1.50	MEF2_03	15.50	6.38E-02	1.84
FLI1B	14.17	GABP_B	9.43	6.74E-02	2.46
CEBPA	22.42	CEBP_01	9.96	6.81E-02	2.81
FOXO3B	12.42	FOX_Q2	10.68	6.86E-02	2.84
MAF	1.50	TCF11_01	2.69	6.91E-02	2.38
NFE2L1	9.50	TCF11_01	2.69	6.91E-02	2.38
NFE2L2	7.08	TCF11_01	2.69	6.91E-02	2.38
FLI1B	14.17	CETS1P54_02	23.42	7.18E-02	1.78
FLI1B	14.17	PU1_Q6	9.96	7.69E-02	3.30
FLI1B	14.17	ELK1_01	1.51	7.70E-02	1.75
CEBPA	22.42	CEBPB_01	5.09	8.03E-02	2.32
CEBPA	22.42	CEBPB_02	9.50	8.28E-02	2.30
FOXO3B	12.42	HFH4_01	7.44	8.35E-02	2.34
MAF	1.50	VMAF_01	21.26	8.83E-02	2.02
NFE2L1	9.50	VMAF_01	21.26	8.83E-02	2.02
NFE2L2	7.08	VMAF_01	21.26	8.83E-02	2.02
FLI1B	14.17	CETS1P54_01	2.47	9.00E-02	1.66
FOXA1	14.67	HNF3_Q6_01	10.56	9.15E-02	2.53
FLI1B	14.17	ETS1_B	9.96	9.26E-02	2.49
HOXA4A	16.92	HOXA4_Q2	18.10	9.63E-02	2.66
FOXO3B	12.42	FOXO1_01	18.67	9.72E-02	2.11
FOXO3B	12.42	HFH8_01	13.10	9.93E-02	1.71
ATF4B1	15.75	ATF4_Q2	1.85	1.01E-01	1.48
ATF6	2.58	ATF4_Q2	1.85	1.01E-01	1.48
CREB3L3	7.00	ATF4_Q2	1.85	1.01E-01	1.48

Table S7: Circadian phase of transcription factor and their targets.

TF	TF PK	TRANSFAC MOTIF	Target PK	P value	Odd Ratio
SI:CH211-199M3.9	2.58	ATF4_Q2	1.85	1.01E-01	1.48
ATF4B1	15.75	CREBP1_Q2	19.75	1.01E-01	1.54
ATF6	2.58	CREBP1_Q2	19.75	1.01E-01	1.54
CREB3L3	7.00	CREBP1_Q2	19.75	1.01E-01	1.54
SI:CH211-199M3.9	2.58	CREBP1_Q2	19.75	1.01E-01	1.54
FOXO3B	12.42	FOXP1_01	18.77	1.02E-01	1.89
CDX1A	15.83	CDX_Q5	2.35	1.03E-01	1.69
CDX1B	14.33	CDX_Q5	2.35	1.03E-01	1.69
HOXA4A	16.92	HOX13_01	0.10	1.04E-01	1.72
TGIF1	13.67	TGIF_01	0.77	1.22E-01	1.59
ATF4B1	15.75	CREB_Q3	17.38	1.28E-01	6.94
ATF6	2.58	CREB_Q3	17.38	1.28E-01	6.94
CREB3L3	7.00	CREB_Q3	17.38	1.28E-01	6.94
SI:CH211-199M3.9	2.58	CREB_Q3	17.38	1.28E-01	6.94
FLI1B	14.17	TEL2_Q6	18.96	1.32E-01	1.54
HSF2	3.75	HSF_Q6	3.48	1.45E-01	1.48
PAX6A	15.50	PAX6_Q2	17.98	1.50E-01	1.60
ARNTL1A	13.42	ARNT_01	23.69	1.50E-01	1.38
ARNTL1B	12.79	ARNT_01	23.69	1.50E-01	1.38
ARNTL2	16.29	ARNT_01	23.69	1.50E-01	1.38
CLOCK	15.08	ARNT_01	23.69	1.50E-01	1.38
CLOCK3	15.08	ARNT_01	23.69	1.50E-01	1.38
FOXO3B	12.42	XFD3_01	23.88	1.67E-01	1.54
MEF2A	1.50	MEF2_04	17.52	2.05E-01	1.50
MYOG	10.63	E2A_Q2	14.18	2.07E-01	1.53
TCF12	13.75	E2A_Q2	14.18	2.07E-01	1.53
FOXO3B	12.42	FREAC7_01	0.02	2.12E-01	1.51
CDX1A	15.83	CDXA_02	NA	NA	NA
CDX1B	14.33	CDXA_02	NA	NA	NA
USF1	13.17	USF2_Q6	NA	NA	NA
