# Supplementary material for: Analysis of a Gene Regulatory Cascade Mediating Circadian Rhythm in Zebrafish
Source: PLoS Comput Biol. 2013 Feb 28;9(2):e1002940. doi: 10.1371/journal.pcbi.1002940 (PMC3585402; doi:10.1371/journal.pcbi.1002940)
Supplement: Table S8 — Enrichment of co-expressed genes in circadian genes, circadian phase, and TF binding sites. (PDF) [file pcbi.1002940.s013.pdf]

Table S8: Enrichment of co-expressed genes in circadian genes, circadian phase, and TF binding sites.

ClusterID	Circadian Gene Enrich p value	Circadian Gene Enrich Odd Ratio	Enriched Pk	PK Enrich p value	Pk Enrich Odd Ratio	Matrix AC	TF Enrich p value	TF Enrich Odd Ratio
531	4.48E-06	9.05	5.23	1.75E-02	3.98	PPAR_DR1_Q2	4.06E-04	14.96
183	7.42E-06	3.85	0.94	7.89E-12	59.35	YY1_Q6_02	5.71E-07	7.75
181	1.14E-05	3.70	0.17	3.78E-10	21.41	YY1_Q6_02	4.27E-07	8.01
174	2.30E-05	0.07	21.02	9.31E-02	Inf	ELK1_02	2.01E-10	11.06
81	3.53E-05	0.07	18.02	1.91E-01	Inf	TATA_01	8.18E-09	13.63
318	8.87E-05	1.94	23.76	2.54E-21	16.83	MYC_Q2	4.70E-19	10.94
275	4.61E-04	0.09	23.52	2.06E-01	Inf	ZNF219_01	6.81E-05	10.12
173	4.79E-04	0.22	18.65	8.14E-04	Inf	CETS1P54_03	5.03E-06	7.07
169	5.24E-04	2.71	3.22	2.46E-02	2.84	GATA1_03	5.36E-07	13.36
353	6.78E-04	0.15	21.55	1.87E-01	9.17	E2F_Q6_01	3.99E-08	11.12
160	7.03E-04	0.09	8.18	5.58E-02	Inf	SP1_Q4_01	5.43E-06	8.57
335	7.10E-04	9.71	22.51	8.80E-04	15.51	NA	NA	NA
48	1.95E-03	5.55	10.80	6.60E-02	5.90	NA	NA	NA
517	2.28E-03	0.17	17.02	6.14E-02	Inf	RFX1_02	4.08E-09	14.71
71	2.49E-03	0.10	9.84	3.72E-02	Inf	IRF_Q6	9.14E-14	59.87
56	2.60E-03	8.32	16.44	4.98E-02	5.40	NA	NA	NA
200	3.21E-03	3.79	20.06	1.50E-08	77.98	LHX3_01	1.91E-04	11.57
340	3.32E-03	0.18	7.68	4.36E-03	Inf	FXR_Q3	1.38E-04	11.79
525	3.60E-03	2.72	7.13	3.47E-02	3.91	HNF1_01	1.15E-05	15.56
528	3.79E-03	0.11	10.27	4.40E-02	Inf	TATA_01	1.17E-10	18.44
115	4.31E-03	0.27	13.51	1.97E-01	3.76	FAC1_01	2.71E-07	14.83
532	4.87E-03	3.47	21.60	1.33E-02	5.99	COUP_01	4.88E-04	14.23
171	5.43E-03	6.24	17.35	1.30E-01	3.48	NA	NA	NA
140	5.99E-03	16.63	13.63	2.02E-01	3.69	NA	NA	NA
386	6.07E-03	0.11	20.88	9.06E-02	Inf	NA	NA	NA
349	7.29E-03	0.19	23.93	5.25E-02	Inf	E2F4DP2_01	1.31E-04	7.32
519	7.29E-03	0.19	9.84	7.30E-02	26.09	NA	NA	NA
457	8.15E-03	0.25	0.17	1.43E-01	6.43	FAC1_01	8.55E-08	24.51
249	8.21E-03	2.36	23.26	4.61E-11	69.14	CLOCKBMAL_Q6	1.58E-07	11.42
172	1.00E-02	4.99	13.63	1.16E-01	3.69	NA	NA	NA
247	1.04E-02	2.04	23.35	3.74E-07	10.51	ELK1_02	5.25E-07	7.37
536	1.08E-02	0.20	7.46	5.22E-03	Inf	STAT5A_02	7.80E-04	7.84

Table S8: Enrichment of co-expressed genes in circadian genes, circadian phase, and TF binding sites.

ClusterID	Circadian Gene Enrich p value	Circadian Gene Enrich Odd Ratio	Enriched Pk	PK Enrich p value	Pk Enrich Odd Ratio	Matrix AC	TF Enrich p value	TF Enrich Odd Ratio
324	1.13E-02	0.20	20.88	8.25E-03	Inf	NA	NA	NA
104	1.52E-02	8.31	8.47	1.46E-02	18.77	NA	NA	NA
352	1.57E-02	0.38	3.82	2.94E-02	6.61	E2F_03	7.31E-17	18.14
82	1.57E-02	0.22	14.09	5.60E-02	Inf	TATA_01	6.03E-08	12.87
106	1.57E-02	2.15	9.84	2.07E-02	6.05	AREB6_01	8.25E-07	11.00
491	1.59E-02	0.21	9.84	7.30E-02	26.09	LEF1TCF1_Q4	3.84E-08	20.62
518	2.40E-02	0.29	18.26	6.06E-03	Inf	RFX1_02	2.46E-09	19.65
309	2.47E-02	0.44	22.34	7.60E-02	4.00	OCT4_01	3.12E-05	11.53
417	2.96E-02	2.68	20.88	1.93E-01	2.89	CREB_Q4_01	1.81E-04	12.56
437	2.97E-02	0.59	11.76	5.99E-03	4.59	NFMUE1_Q6	2.55E-04	3.69
150	3.00E-02	5.54	7.34	3.02E-02	12.49	MYCMAX_03	1.67E-04	82.59
98	3.09E-02	0.15	7.58	6.70E-02	Inf	NA	NA	NA
78	3.31E-02	0.45	15.48	1.01E-03	17.33	PU1_Q6	6.42E-08	14.25
479	3.34E-02	0.34	2.98	2.91E-01	2.74	MYOD_01	5.42E-14	32.16
218	3.77E-02	0.00	5.54	2.49E-02	Inf	NA	NA	NA
20	3.83E-02	3.12	17.47	1.21E-01	3.63	USF_C	3.47E-04	29.02
168	3.91E-02	1.86	22.10	4.68E-02	3.04	CRX_Q4	2.79E-07	14.94
419	4.48E-02	3.47	5.23	4.23E-02	6.97	NA	NA	NA
54	4.48E-02	3.47	5.71	1.68E-01	3.75	E2_01	6.48E-04	22.73
430	4.69E-02	0.16	11.69	8.44E-02	Inf	YY1_Q6	1.29E-04	9.91
509	4.83E-02	0.33	10.58	1.48E-01	9.17	AIRE_02	8.34E-04	7.70
511	4.86E-02	0.32	19.44	4.05E-02	14.56	FOXD3_01	1.04E-04	12.23
74	5.10E-02	4.16	5.02	1.54E-01	4.51	NA	NA	NA
33	5.35E-02	6.23	16.85	1.63E-01	5.81	NA	NA	NA
137	6.49E-02	2.97	14.69	1.11E-01	4.35	NA	NA	NA
359	6.79E-02	0.34	9.26	1.14E-01	12.23	COREBINDINGFACTOR_Q6	4.32E-08	19.70
291	6.82E-02	0.19	19.44	1.21E-01	Inf	NA	NA	NA
87	6.82E-02	0.19	9.84	3.72E-02	Inf	NA	NA	NA
385	6.82E-02	0.18	18.67	1.64E-01	Inf	NA	NA	NA
15	7.08E-02	0.28	3.89	5.17E-02	Inf	TATA_01	6.63E-05	10.67
348	7.08E-02	0.28	10.90	1.11E-01	16.62	UF1H3BETA_Q6	5.39E-04	8.83
462	7.08E-02	0.28	22.10	1.47E-02	Inf	NA	NA	NA

Table S8: Enrichment of co-expressed genes in circadian genes, circadian phase, and TF binding sites.

ClusterID	Circadian Gene Enrich p value	Circadian Gene Enrich Odd Ratio	Enriched Pk	PK Enrich p value	Pk Enrich Odd Ratio	Matrix AC	TF Enrich p value	TF Enrich Odd Ratio
494	7.10E-02	0.17	6.89	9.80E-02	Inf	TBP_Q6	6.12E-10	39.86
201	7.35E-02	2.08	21.60	2.93E-03	7.49	NA	NA	NA
521	7.52E-02	2.43	1.39	2.06E-02	6.52	NA	NA	NA
445	7.80E-02	3.33	4.44	2.66E-02	12.17	NA	NA	NA
167	7.82E-02	1.99	2.98	1.15E-02	4.80	TATA_01	6.63E-05	10.67
182	8.50E-02	1.91	23.18	8.11E-06	20.62	NA	NA	NA
38	8.74E-02	0.41	0.60	2.73E-01	2.90	ATF_01	4.42E-04	7.11
191	9.17E-02	4.16	19.63	3.55E-02	15.75	NA	NA	NA
5	9.17E-02	4.16	4.13	1.23E-01	7.14	NA	NA	NA
420	9.44E-02	0.49	16.34	5.18E-02	5.32	NA	NA	NA
246	9.84E-02	8.31	11.86	9.42E-03	Inf	NA	NA	NA
258	9.84E-02	8.31	11.76	8.48E-03	Inf	NA	NA	NA
300	1.01E-01	0.21	5.35	1.69E-01	Inf	NFY_Q6_01	3.29E-04	8.39
401	1.02E-01	0.30	8.30	1.05E-01	17.63	NA	NA	NA
53	1.11E-01	2.77	18.05	2.38E-02	12.77	NF1_Q6	1.49E-04	44.97
319	1.15E-01	2.08	18.26	7.46E-04	13.52	NA	NA	NA
323	1.25E-01	0.54	20.09	3.96E-02	5.36	YY1_Q6_02	1.54E-06	5.65
475	1.25E-01	0.54	0.02	2.10E-01	0.00	NA	NA	NA
341	1.28E-01	1.69	6.60	4.28E-02	3.74	NFY_01	9.83E-06	6.54
507	1.32E-01	0.40	9.84	1.07E-01	13.08	NA	NA	NA
94	1.35E-01	1.64	14.98	2.54E-02	0.00	GC_01	8.93E-05	8.13
226	1.38E-01	3.12	22.85	6.88E-02	10.51	NA	NA	NA
387	1.42E-01	0.48	6.26	1.42E-02	11.12	AHRARNT_02	3.62E-04	7.16
231	1.47E-01	0.32	16.75	6.77E-02	Inf	NA	NA	NA
529	1.49E-01	2.60	7.01	6.84E-02	6.72	GATA4_Q3	1.21E-04	48.30
64	1.49E-01	2.60	7.27	4.22E-03	17.90	NA	NA	NA
159	1.51E-01	0.23	11.35	7.20E-02	Inf	OLF1_01	1.57E-05	21.00
230	1.51E-01	0.23	20.88	9.06E-02	Inf	NA	NA	NA
274	1.51E-01	0.23	23.38	1.94E-01	Inf	E2F4DP2_01	6.26E-05	16.24
117	1.53E-01	0.22	5.42	1.64E-01	Inf	NA	NA	NA
267	1.53E-01	0.22	13.10	1.73E-01	Inf	E2_Q6	2.07E-04	16.71
426	1.53E-01	0.22	20.88	9.06E-02	Inf	NA	NA	NA

Table S8: Enrichment of co-expressed genes in circadian genes, circadian phase, and TF binding sites.

ClusterID	Circadian Gene Enrich p value	Circadian Gene Enrich Odd Ratio	Enriched Pk	PK Enrich p value	Pk Enrich Odd Ratio	Matrix AC	TF Enrich p value	TF Enrich Odd Ratio
393	1.61E-01	0.56	11.86	3.54E-02	5.63	NA	NA	NA
480	1.61E-01	0.46	20.06	5.61E-02	8.68	NA	NA	NA
332	1.66E-01	2.31	14.64	1.07E-03	Inf	RFX1_02	9.15E-07	28.48
166	1.75E-01	1.70	3.02	1.39E-02	5.54	TATA_01	9.57E-07	13.51
512	1.82E-01	1.49	23.02	2.96E-02	3.29	ELK1_02	1.24E-04	6.15
372	1.82E-01	0.43	5.23	8.34E-02	9.28	MEIS1AHOXA9_01	5.50E-05	14.66
276	1.88E-01	2.08	0.10	1.23E-02	13.11	NA	NA	NA
408	1.88E-01	2.08	15.26	2.60E-02	9.99	MYCMAX_B	1.99E-04	18.15
501	1.88E-01	2.08	12.84	1.69E-01	3.73	CREB_01	5.78E-04	14.89
179	1.89E-01	0.50	20.88	6.84E-02	6.72	YY1_Q6	7.16E-07	8.47
310	1.89E-01	0.50	12.26	1.12E-01	4.92	TBX5_01	6.54E-07	17.59
119	1.90E-01	2.49	13.34	7.31E-03	Inf	NA	NA	NA
28	1.90E-01	2.49	9.84	1.07E-01	13.08	NFKAPPAB50_01	7.58E-04	25.45
222	1.94E-01	0.00	4.10	2.20E-01	Inf	NA	NA	NA
209	1.96E-01	1.66	17.76	4.89E-06	35.66	ZTA_Q2	9.10E-04	10.48
321	2.05E-01	0.38	17.64	4.30E-02	Inf	OCT1_02	5.41E-04	12.87
421	2.05E-01	0.38	9.84	7.30E-02	26.09	YY1_Q6	6.97E-04	8.94
93	2.05E-01	0.38	9.84	7.30E-02	26.09	SP1_Q6_01	4.16E-04	9.72
97	2.05E-01	0.38	20.88	1.73E-01	10.08	NA	NA	NA
163	2.05E-01	0.36	6.89	1.86E-01	9.23	NFY_Q6_01	7.26E-05	9.04
240	2.10E-01	0.35	18.67	3.02E-01	5.09	NA	NA	NA
346	2.13E-01	0.55	7.68	5.46E-02	7.15	E2F1_Q4_01	3.70E-09	12.47
362	2.13E-01	0.55	6.89	1.11E-01	4.62	PAX4_03	2.31E-04	7.89
530	2.16E-01	1.89	20.88	6.56E-03	15.10	NA	NA	NA
180	2.16E-01	0.54	3.10	3.26E-04	Inf	NRF1_Q6	1.29E-04	5.50
474	2.17E-01	0.49	0.26	4.79E-02	9.28	EN1_01	7.51E-07	24.23
442	2.22E-01	0.65	0.77	5.97E-04	8.47	NA	NA	NA
108	2.24E-01	0.26	8.09	5.71E-02	Inf	NA	NA	NA
19	2.24E-01	0.26	5.88	1.43E-01	Inf	NA	NA	NA
293	2.24E-01	0.26	21.84	1.09E-01	Inf	NA	NA	NA
286	2.28E-01	0.24	13.34	1.94E-01	Inf	NFY_01	7.34E-05	9.56
242	2.35E-01	1.52	12.02	4.22E-04	10.21	NA	NA	NA

Table S8: Enrichment of co-expressed genes in circadian genes, circadian phase, and TF binding sites.

ClusterID	Circadian Gene Enrich p value	Circadian Gene Enrich Odd Ratio	Enriched Pk	PK Enrich p value	Pk Enrich Odd Ratio	Matrix AC	TF Enrich p value	TF Enrich Odd Ratio
414	2.41E-01	2.37	13.27	1.64E-01	4.31	NA	NA	NA
448	2.41E-01	2.37	9.84	1.41E-01	8.74	NA	NA	NA
355	2.50E-01	0.53	20.88	6.84E-02	6.72	OCT1_07	1.84E-05	13.64
403	2.50E-01	0.46	20.52	2.54E-02	19.12	NA	NA	NA
464	2.50E-01	0.46	17.47	1.21E-01	7.25	NA	NA	NA
214	2.51E-01	2.77	9.84	7.30E-02	26.09	NA	NA	NA
397	2.51E-01	2.77	20.88	1.73E-01	10.08	NA	NA	NA
503	2.51E-01	2.77	19.85	1.16E-02	Inf	NA	NA	NA
497	2.53E-01	0.44	10.58	7.95E-03	36.54	E2F_01	3.70E-04	9.48
136	2.65E-01	2.08	12.55	9.34E-02	6.31	NA	NA	NA
350	2.74E-01	0.59	23.09	1.10E-02	9.32	CREBATF_Q6	7.26E-04	5.39
298	2.77E-01	0.64	0.10	7.21E-03	6.56	E2F4DP2_01	1.49E-08	8.43
92	2.82E-01	1.50	5.59	7.04E-03	6.73	OCT1_07	4.42E-04	9.19
165	2.90E-01	0.39	13.44	4.02E-02	Inf	NA	NA	NA
229	2.90E-01	0.39	18.05	3.64E-02	Inf	PAX4_02	7.97E-04	11.30
520	2.90E-01	0.39	10.90	1.11E-01	16.62	NA	NA	NA
339	2.94E-01	1.39	12.43	4.03E-03	5.68	NA	NA	NA
506	2.95E-01	1.54	22.75	5.54E-02	3.66	NA	NA	NA
329	2.95E-01	1.85	20.88	4.37E-02	10.08	YY1_Q6	6.71E-04	14.29
32	2.95E-01	1.85	15.55	7.59E-02	7.38	MAF_Q6	7.10E-04	22.58
344	2.97E-01	1.71	21.26	2.25E-03	12.85	NA	NA	NA
354	3.00E-01	0.62	4.97	7.99E-06	Inf	E2F4DP1_01	2.03E-17	25.69
418	3.08E-01	1.62	15.05	2.02E-02	6.56	NA	NA	NA
314	3.15E-01	1.35	16.58	6.33E-06	16.82	E2F4DP1_01	8.07E-04	6.13
88	3.22E-01	0.59	9.84	1.73E-01	6.56	NA	NA	NA
388	3.24E-01	0.64	18.94	9.87E-02	3.53	WHN_B	3.85E-04	8.95
290	3.26E-01	0.56	23.54	6.29E-02	5.79	HIC1_03	1.24E-04	9.15
197	3.30E-01	0.30	22.51	1.39E-01	Inf	CAAT_01	3.88E-08	30.63
232	3.30E-01	0.30	9.26	3.97E-02	Inf	RSRFC4_Q2	1.11E-04	21.72
366	3.30E-01	0.30	6.34	1.17E-01	Inf	MSX1_01	1.80E-05	37.67
29	3.30E-01	0.32	12.34	1.26E-01	Inf	NA	NA	NA
151	3.30E-01	2.08	7.85	1.19E-01	15.36	NA	NA	NA

Table S8: Enrichment of co-expressed genes in circadian genes, circadian phase, and TF binding sites.

ClusterID	Circadian Gene Enrich p value	Circadian Gene Enrich Odd Ratio	Enriched Pk	PK Enrich p value	Pk Enrich Odd Ratio	Matrix AC	TF Enrich p value	TF Enrich Odd Ratio
400	3.30E-01	2.08	4.44	3.93E-02	Inf	NA	NA	NA
80	3.30E-01	2.08	20.88	1.73E-01	10.08	NA	NA	NA
158	3.38E-01	0.28	18.43	1.75E-01	Inf	OCT4_01	3.31E-04	30.53
273	3.38E-01	0.28	4.80	1.95E-01	Inf	NA	NA	NA
10	3.39E-01	0.52	9.84	1.07E-01	13.08	NA	NA	NA
381	3.39E-01	0.52	5.64	6.58E-02	10.81	ZNF219_01	5.26E-04	12.97
156	3.44E-01	0.48	10.90	9.49E-03	33.17	ZF5_B	4.62E-05	12.22
422	3.44E-01	0.48	15.94	1.94E-01	5.09	NA	NA	NA
510	3.44E-01	0.48	21.10	8.37E-04	Inf	NA	NA	NA
515	3.44E-01	0.48	13.90	1.33E-01	6.77	NA	NA	NA
215	3.51E-01	4.15	20.18	9.86E-02	Inf	NA	NA	NA
216	3.51E-01	4.15	6.72	1.02E-01	Inf	NA	NA	NA
46	3.51E-01	4.15	17.69	2.06E-01	Inf	NA	NA	NA
99	3.51E-01	4.15	5.88	1.43E-01	Inf	NA	NA	NA
473	3.51E-01	0.64	11.35	6.42E-02	6.49	PR_02	7.32E-09	19.83
499	3.54E-01	0.61	19.85	1.29E-01	4.18	CEBP_Q3	4.47E-05	10.76
195	3.64E-01	1.45	2.76	3.06E-05	Inf	ATF6_01	2.30E-04	10.55
243	3.64E-01	1.45	3.82	2.04E-02	5.51	HSF2_01	1.13E-10	35.41
342	3.71E-01	1.60	23.86	1.05E-02	13.82	NA	NA	NA
62	3.71E-01	1.60	9.77	5.35E-04	38.41	HNF4_DR1_Q3	3.94E-04	14.79
360	3.78E-01	0.64	11.18	6.95E-02	5.90	NA	NA	NA
477	3.79E-01	0.71	23.02	9.98E-02	2.82	S8_01	2.75E-04	6.21
322	3.79E-01	1.39	23.86	2.41E-03	10.37	MEF2_Q6_01	2.03E-04	10.65
76	3.83E-01	0.55	7.75	2.18E-02	15.04	IRF7_01	2.59E-05	18.55
487	3.89E-01	1.44	13.44	8.67E-02	3.20	NA	NA	NA
513	3.89E-01	1.44	1.06	1.60E-02	5.33	NA	NA	NA
148	3.90E-01	2.08	21.22	2.48E-02	19.40	NA	NA	NA
364	3.90E-01	2.08	14.59	1.59E-01	5.93	NA	NA	NA
59	3.90E-01	2.08	14.59	1.59E-01	5.93	GABP_B	3.01E-05	37.33
18	3.95E-01	1.48	8.59	2.04E-02	13.59	NA	NA	NA
371	4.01E-01	0.46	20.88	1.73E-01	10.08	CP2_01	1.18E-04	20.87
380	4.01E-01	0.46	9.84	7.30E-02	26.09	NA	NA	NA

Table S8: Enrichment of co-expressed genes in circadian genes, circadian phase, and TF binding sites.

ClusterID	Circadian Gene Enrich p value	Circadian Gene Enrich Odd Ratio	Enriched Pk	PK Enrich p value	Pk Enrich Odd Ratio	Matrix AC	TF Enrich p value	TF Enrich Odd Ratio
116	4.05E-01	0.44	5.88	2.06E-02	Inf	NA	NA	NA
410	4.14E-01	1.56	16.85	4.13E-02	5.81	NA	NA	NA
472	4.16E-01	0.61	0.26	9.80E-02	4.64	HNF4_01	1.14E-12	44.50
145	4.20E-01	1.78	18.67	7.24E-02	10.18	NA	NA	NA
416	4.20E-01	1.78	2.14	1.96E-01	5.04	NA	NA	NA
524	4.20E-01	1.78	7.18	1.89E-02	22.69	NA	NA	NA
122	4.23E-01	0.83	15.89	3.14E-04	4.28	NRSF_Q4	4.65E-10	6.73
129	4.28E-01	1.47	14.18	3.32E-02	6.32	NA	NA	NA
250	4.28E-01	1.47	21.77	1.89E-02	8.45	NA	NA	NA
7	4.28E-01	1.47	3.48	3.63E-02	6.11	SP1_Q4_01	3.68E-04	10.21
505	4.43E-01	0.67	17.26	2.81E-02	6.72	NA	NA	NA
476	4.44E-01	0.65	20.88	9.65E-02	5.05	CEBPA_01	9.14E-04	10.75
91	4.44E-01	0.65	3.65	3.32E-02	6.32	NA	NA	NA
308	4.48E-01	1.34	17.18	2.45E-02	3.98	ARNT_01	1.02E-17	28.49
347	4.48E-01	1.34	1.18	2.82E-04	11.96	PAX4_02	3.41E-05	8.89
363	4.54E-01	0.57	17.76	1.06E-01	7.93	NA	NA	NA
389	4.56E-01	0.54	12.77	5.87E-02	11.62	NA	NA	NA
425	4.56E-01	1.56	18.05	9.52E-02	8.51	NA	NA	NA
427	4.56E-01	1.56	18.96	3.05E-03	Inf	NA	NA	NA
95	4.62E-01	1.45	4.85	2.78E-02	5.72	SMAD_Q6_01	4.19E-04	13.40
383	4.62E-01	1.27	11.76	1.42E-02	5.68	ATF_01	3.70E-04	5.99
34	4.77E-01	2.08	23.86	2.25E-01	Inf	NA	NA	NA
37	4.77E-01	2.08	13.51	2.10E-01	Inf	NA	NA	NA
534	4.77E-01	1.28	3.89	8.45E-03	4.77	HNF4_Q6_01	5.48E-12	17.67
2	4.82E-01	0.38	11.42	7.44E-02	Inf	HNF3B_01	3.05E-04	32.87
367	4.82E-01	0.38	7.51	6.95E-02	Inf	PAX4_03	8.85E-04	21.61
39	4.84E-01	1.25	5.88	3.81E-03	5.99	EGR1_01	6.81E-04	8.03
490	4.84E-01	1.25	13.18	2.53E-03	6.18	NA	NA	NA
154	4.84E-01	0.35	11.54	8.00E-02	Inf	NA	NA	NA
301	4.84E-01	0.35	12.89	1.53E-01	Inf	AHR_Q5	2.10E-04	37.42
441	4.84E-01	0.35	19.92	1.07E-01	Inf	FXR_Q3	4.69E-04	27.00
446	4.84E-01	0.35	8.52	4.90E-02	Inf	HFH4_01	2.00E-04	40.61

Table S8: Enrichment of co-expressed genes in circadian genes, circadian phase, and TF binding sites.

ClusterID	Circadian Gene Enrich p value	Circadian Gene Enrich Odd Ratio	Enriched Pk	PK Enrich p value	Pk Enrich Odd Ratio	Matrix AC	TF Enrich p value	TF Enrich Odd Ratio
90	4.84E-01	0.35	4.22	2.15E-01	Inf	RBPJK_01	5.61E-04	25.37
378	4.91E-01	0.74	20.26	4.79E-03	9.24	ZF5_B	6.24E-07	8.44
320	4.95E-01	0.64	5.88	1.01E-01	5.99	NA	NA	NA
392	4.95E-01	1.66	12.77	1.14E-02	17.43	SF1_Q6_01	3.75E-07	33.35
128	4.96E-01	0.61	20.88	4.37E-02	10.08	NA	NA	NA
239	5.00E-01	0.59	10.85	1.75E-02	17.01	NA	NA	NA
52	5.11E-01	1.51	9.05	1.52E-01	8.03	NA	NA	NA
68	5.11E-01	1.51	17.98	2.54E-02	12.41	NA	NA	NA
435	5.32E-01	1.38	0.77	4.75E-03	Inf	NA	NA	NA
202	5.36E-01	1.29	20.18	5.17E-02	4.59	PAX4_04	2.79E-04	9.86
146	5.53E-01	0.55	8.52	9.56E-02	19.54	NA	NA	NA
16	5.53E-01	0.55	19.13	2.59E-01	6.21	NA	NA	NA
394	5.53E-01	0.55	20.88	1.73E-01	10.08	NA	NA	NA
432	5.53E-01	0.55	20.88	8.25E-03	Inf	NA	NA	NA
522	5.53E-01	0.55	1.44	7.98E-02	Inf	HNF1_01	4.48E-05	33.59
164	5.54E-01	0.52	20.88	1.73E-01	10.08	LXR_Q3	1.34E-04	19.47
299	5.54E-01	0.52	18.43	3.19E-01	4.73	YY1_Q6	1.03E-05	18.41
312	5.54E-01	0.71	3.60	1.56E-01	3.15	EFC_Q6	4.14E-04	9.07
254	5.59E-01	0.49	14.42	6.11E-02	Inf	NA	NA	NA
449	5.59E-01	0.49	20.06	1.08E-02	Inf	NA	NA	NA
261	5.78E-01	1.38	21.43	9.86E-02	Inf	NA	NA	NA
281	5.78E-01	1.38	4.22	2.15E-01	Inf	NA	NA	NA
356	5.78E-01	1.38	4.80	1.95E-01	Inf	NA	NA	NA
377	5.78E-01	1.38	21.60	1.00E-01	Inf	NA	NA	NA
73	5.78E-01	1.38	7.34	7.44E-02	Inf	NA	NA	NA
369	5.79E-01	0.73	7.51	8.06E-02	5.39	NA	NA	NA
330	5.83E-01	1.30	15.10	2.38E-02	10.34	NA	NA	NA
486	5.87E-01	1.20	7.01	7.12E-02	3.79	NA	NA	NA
51	5.87E-01	1.20	4.22	3.19E-03	6.39	CREB_01	4.30E-04	5.12
265	5.94E-01	1.17	23.35	9.81E-03	5.05	NFMUE1_Q6	2.64E-05	6.79
25	6.28E-01	1.66	17.14	5.75E-02	Inf	NA	NA	NA
411	6.33E-01	1.19	13.51	2.05E-02	7.52	NA	NA	NA

Table S8: Enrichment of co-expressed genes in circadian genes, circadian phase, and TF binding sites.

ClusterID	Circadian Gene Enrich p value	Circadian Gene Enrich Odd Ratio	Enriched Pk	PK Enrich p value	Pk Enrich Odd Ratio	Matrix AC	TF Enrich p value	TF Enrich Odd Ratio
79	6.33E-01	1.19	17.02	1.66E-01	3.05	AMEF2_Q6	1.54E-04	18.27
1	6.50E-01	1.21	13.18	3.32E-03	11.04	SRF_Q4	3.79E-07	20.09
211	6.57E-01	1.38	18.65	2.83E-02	Inf	FOXP1_01	2.67E-04	47.99
404	6.57E-01	1.38	16.46	7.29E-02	Inf	NA	NA	NA
451	6.57E-01	1.38	20.18	9.78E-03	Inf	NA	NA	NA
45	6.57E-01	1.38	13.75	5.03E-02	Inf	NA	NA	NA
483	6.57E-01	1.38	12.43	1.74E-02	Inf	NA	NA	NA
488	6.57E-01	1.38	20.88	1.73E-01	10.08	NA	NA	NA
6	6.57E-01	1.38	5.54	2.49E-02	Inf	NA	NA	NA
526	6.59E-01	1.16	6.72	2.77E-02	6.60	HNF4_Q6_01	7.58E-05	10.41
86	6.73E-01	1.19	8.59	3.17E-04	20.35	HIF1_Q5	2.02E-04	17.18
189	6.89E-01	1.19	8.30	1.05E-01	17.63	CREBP1_01	8.78E-07	58.32
237	6.89E-01	1.19	17.47	4.70E-02	Inf	NA	NA	NA
402	6.89E-01	1.19	22.92	2.73E-02	Inf	SP1_Q2_01	2.99E-04	46.13
493	6.89E-01	1.19	4.97	3.45E-02	Inf	E2F1_Q3_01	1.37E-05	51.24
4	6.89E-01	1.19	7.97	1.14E-01	16.06	NA	NA	NA
133	6.98E-01	0.46	18.55	1.73E-01	Inf	MSX1_01	2.81E-04	33.82
194	6.98E-01	0.46	8.64	4.59E-02	Inf	SRF_Q5_01	1.23E-04	62.90
266	6.98E-01	0.46	13.18	1.85E-01	Inf	NA	NA	NA
279	6.98E-01	0.46	8.69	4.40E-02	Inf	CETS168_Q6	5.71E-04	25.21
306	6.98E-01	0.46	4.44	1.98E-01	Inf	NA	NA	NA
461	6.98E-01	0.46	20.88	9.06E-02	Inf	NA	NA	NA
516	7.03E-01	0.81	16.85	7.69E-02	3.88	NA	NA	NA
253	7.03E-01	0.41	19.44	1.21E-01	Inf	NA	NA	NA
368	7.03E-01	0.41	13.51	2.10E-01	Inf	POU1F1_Q6	7.97E-06	49.09
374	7.03E-01	0.41	11.35	7.20E-02	Inf	NA	NA	NA
395	7.03E-01	0.41	5.35	1.69E-01	Inf	NA	NA	NA
405	7.03E-01	0.41	12.02	1.05E-01	Inf	NA	NA	NA
413	7.08E-01	0.89	19.13	9.46E-02	2.39	ATF_B	5.42E-04	3.91
107	7.08E-01	1.15	10.27	6.95E-02	5.49	NA	NA	NA
207	7.13E-01	1.38	13.51	1.14E-01	7.52	NA	NA	NA
287	7.13E-01	1.38	15.10	1.90E-01	5.17	NA	NA	NA

Table S8: Enrichment of co-expressed genes in circadian genes, circadian phase, and TF binding sites.

ClusterID	Circadian Gene Enrich p value	Circadian Gene Enrich Odd Ratio	Enriched Pk	PK Enrich p value	Pk Enrich Odd Ratio	Matrix AC	TF Enrich p value	TF Enrich Odd Ratio
325	7.13E-01	1.38	9.50	1.13E-01	12.43	NA	NA	NA
63	7.13E-01	1.38	9.84	1.07E-01	13.08	NA	NA	NA
143	7.26E-01	1.25	20.88	2.48E-01	5.05	USF_02	2.27E-05	20.80
147	7.26E-01	1.25	9.26	1.14E-01	12.23	NA	NA	NA
205	7.26E-01	1.25	19.44	4.05E-02	14.56	NA	NA	NA
244	7.26E-01	1.25	18.34	8.45E-02	9.21	NA	NA	NA
49	7.26E-01	1.25	19.22	4.96E-02	12.88	NA	NA	NA
500	7.26E-01	1.25	10.34	6.04E-03	42.37	NA	NA	NA
502	7.26E-01	1.25	17.98	9.92E-02	8.28	NA	NA	NA
142	7.43E-01	1.13	16.92	1.59E-01	5.93	NA	NA	NA
176	7.43E-01	1.13	5.02	8.73E-02	9.02	NA	NA	NA
523	7.43E-01	1.13	7.27	4.77E-04	Inf	NA	NA	NA
66	7.43E-01	1.13	5.88	2.98E-03	Inf	NA	NA	NA
130	7.50E-01	0.64	14.42	6.11E-02	Inf	NA	NA	NA
27	7.50E-01	0.64	3.94	5.08E-02	Inf	SRF_01	6.32E-08	55.81
294	7.50E-01	0.64	3.05	7.00E-02	Inf	NA	NA	NA
409	7.50E-01	0.64	11.71	1.66E-01	10.56	NA	NA	NA
415	7.50E-01	0.64	20.88	1.73E-01	10.08	NA	NA	NA
485	7.51E-01	1.08	23.59	8.30E-02	0.00	CREB_Q2	6.72E-05	5.91
153	7.52E-01	0.59	12.67	2.10E-02	Inf	NA	NA	NA
313	7.53E-01	0.84	2.42	1.48E-02	4.51	NA	NA	NA
297	7.57E-01	1.28	13.10	1.82E-02	14.35	NA	NA	NA
391	7.57E-01	1.28	17.88	1.78E-01	4.06	NA	NA	NA
390	7.65E-01	1.19	16.01	6.99E-02	7.70	NA	NA	NA
11	7.76E-01	1.11	12.72	1.03E-01	5.93	NA	NA	NA
178	7.76E-01	1.11	3.10	4.70E-03	Inf	SF1_Q6_01	4.42E-04	14.10
412	7.76E-01	1.11	18.96	1.03E-01	5.93	NA	NA	NA
131	7.82E-01	0.73	3.94	1.29E-01	6.89	NA	NA	NA
13	7.82E-01	0.73	7.01	2.32E-02	20.13	CEBPB_01	3.70E-05	27.79
271	7.82E-01	0.73	23.86	1.14E-02	Inf	NFMUE1_Q6	2.08E-04	11.95
96	7.82E-01	0.73	18.96	5.68E-02	11.86	PEBP_Q6	9.32E-06	23.46
436	7.83E-01	0.69	13.51	1.14E-01	7.52	NRF2_01	6.36E-04	12.97

Table S8: Enrichment of co-expressed genes in circadian genes, circadian phase, and TF binding sites.

ClusterID	Circadian Gene Enrich p value	Circadian Gene Enrich Odd Ratio	Enriched Pk	PK Enrich p value	Pk Enrich Odd Ratio	Matrix AC	TF Enrich p value	TF Enrich Odd Ratio
126	7.86E-01	0.65	17.02	1.54E-01	6.09	NA	NA	NA
272	7.92E-01	1.15	20.93	6.93E-02	6.67	NA	NA	NA
345	7.92E-01	1.15	12.60	1.52E-01	4.02	NA	NA	NA
245	8.04E-01	0.79	9.84	1.41E-01	8.74	NA	NA	NA
105	8.05E-01	0.75	14.02	3.03E-03	Inf	AREB6_01	3.07E-05	12.66
396	8.11E-01	1.13	12.34	3.12E-03	13.91	NFKB_C	7.44E-04	22.97
514	8.11E-01	1.13	3.50	2.19E-04	Inf	ATF4_Q2	1.35E-04	12.27
8	8.21E-01	0.83	20.26	7.89E-02	6.16	NA	NA	NA
496	8.21E-01	0.80	12.60	2.34E-02	9.03	NA	NA	NA
527	8.21E-01	0.93	5.35	2.32E-05	6.39	HNF4_01	2.69E-08	7.16
270	8.35E-01	0.80	11.76	9.88E-02	4.97	NA	NA	NA
384	8.37E-01	1.11	5.98	8.33E-02	3.83	NA	NA	NA
161	8.49E-01	1.07	11.69	4.65E-03	8.72	MTF1_Q4	2.67E-05	9.52
9	8.52E-01	1.04	12.34	9.41E-02	3.48	SREBP1_Q6	8.73E-04	7.60
311	8.53E-01	0.87	18.10	9.50E-04	12.82	USF_01	2.26E-15	23.32
508	8.55E-01	1.09	18.34	2.10E-02	4.61	STRA13_01	7.15E-10	13.24
535	8.59E-01	1.04	11.02	1.88E-02	6.82	HNF4_Q6_01	1.79E-07	11.21
498	8.69E-01	1.06	19.34	1.20E-02	4.97	YY1_Q6_02	2.15E-05	5.60
196	8.77E-01	0.91	0.89	9.83E-02	2.72	SRF_01	1.41E-04	7.05
100	1.00E+00	0.69	8.88	4.34E-02	Inf	NA	NA	NA
103	1.00E+00	0.59	6.48	1.13E-01	Inf	NA	NA	NA
118	1.00E+00	0.69	9.84	7.30E-02	26.09	NA	NA	NA
120	1.00E+00	0.83	18.77	1.57E-01	Inf	NA	NA	NA
124	1.00E+00	0.59	4.85	1.89E-01	Inf	TATA_01	9.02E-04	23.92
125	1.00E+00	1.04	17.47	4.70E-02	Inf	IPF1_Q4_01	2.08E-04	40.06
127	1.00E+00	0.94	18.17	2.45E-02	4.40	NRSE_B	1.31E-10	15.42
132	1.00E+00	0.92	20.88	1.73E-01	10.08	NA	NA	NA
135	1.00E+00	0.59	11.18	6.39E-02	Inf	NA	NA	NA
138	1.00E+00	1.04	6.14	1.64E-02	Inf	NA	NA	NA
139	1.00E+00	1.04	13.44	2.00E-01	Inf	NA	NA	NA
141	1.00E+00	0.78	10.44	1.43E-01	9.53	NA	NA	NA
149	1.00E+00	0.75	21.34	1.84E-01	9.37	NA	NA	NA

Table S8: Enrichment of co-expressed genes in circadian genes, circadian phase, and TF binding sites.

ClusterID	Circadian Gene Enrich p value	Circadian Gene Enrich Odd Ratio	Enriched Pk	PK Enrich p value	Pk Enrich Odd Ratio	Matrix AC	TF Enrich p value	TF Enrich Odd Ratio
14	1.00E+00	0.90	19.63	1.22E-02	11.82	PAX9_B	5.79E-04	12.52
155	1.00E+00	0.94	23.35	1.38E-01	0.00	NA	NA	NA
175	1.00E+00	1.00	3.38	6.13E-03	4.76	CREBP1_01	4.54E-04	5.71
185	1.00E+00	0.83	8.47	2.62E-03	Inf	E4F1_Q6	5.61E-05	29.43
187	1.00E+00	0.59	8.64	4.59E-02	Inf	NA	NA	NA
188	1.00E+00	1.04	22.44	1.38E-01	Inf	NA	NA	NA
190	1.00E+00	0.83	6.60	1.07E-01	Inf	NA	NA	NA
192	1.00E+00	0.83	17.64	2.07E-01	Inf	NA	NA	NA
198	1.00E+00	1.00	20.09	1.17E-01	4.47	HSF1_Q6	6.61E-04	8.27
199	1.00E+00	1.04	20.26	1.11E-01	4.62	NA	NA	NA
203	1.00E+00	0.92	22.18	2.34E-01	7.04	NA	NA	NA
208	1.00E+00	0.99	15.22	1.02E-03	10.24	HSF_Q6	7.43E-04	6.17
210	1.00E+00	0.83	13.34	1.94E-01	Inf	NA	NA	NA
212	1.00E+00	0.83	12.17	1.15E-01	Inf	NA	NA	NA
225	1.00E+00	0.75	17.14	5.75E-02	Inf	NA	NA	NA
228	1.00E+00	0.92	0.38	6.20E-02	Inf	CETS1P54_01	5.57E-04	26.63
22	1.00E+00	0.52	20.88	9.06E-02	Inf	NA	NA	NA
233	1.00E+00	0.83	23.09	1.77E-01	Inf	NA	NA	NA
234	1.00E+00	0.69	7.80	1.20E-01	15.20	NA	NA	NA
248	1.00E+00	0.83	20.86	1.76E-01	9.86	CDC5_01	3.48E-04	29.99
24	1.00E+00	0.78	17.35	1.28E-01	6.96	NA	NA	NA
252	1.00E+00	1.04	19.13	1.94E-02	Inf	NA	NA	NA
255	1.00E+00	0.83	11.76	9.18E-02	Inf	NA	NA	NA
256	1.00E+00	0.83	18.72	1.63E-01	Inf	NA	NA	NA
259	1.00E+00	0.59	11.93	1.00E-01	Inf	NA	NA	NA
260	1.00E+00	0.83	13.01	1.66E-01	Inf	NA	NA	NA
262	1.00E+00	0.69	13.44	2.00E-01	Inf	NA	NA	NA
269	1.00E+00	0.83	11.86	9.42E-03	Inf	NA	NA	NA
277	1.00E+00	0.69	20.88	9.06E-02	Inf	NA	NA	NA
278	1.00E+00	0.59	22.75	1.54E-01	Inf	NA	NA	NA
288	1.00E+00	1.04	5.71	6.20E-02	11.23	NA	NA	NA
295	1.00E+00	0.87	13.10	1.41E-01	4.79	YY1_Q6	2.33E-08	21.53

Table S8: Enrichment of co-expressed genes in circadian genes, circadian phase, and TF binding sites.

ClusterID	Circadian Gene Enrich p value	Circadian Gene Enrich Odd Ratio	Enriched Pk	PK Enrich p value	Pk Enrich Odd Ratio	Matrix AC	TF Enrich p value	TF Enrich Odd Ratio
302	1.00E+00	0.69	6.14	1.28E-01	Inf	NA	NA	NA
303	1.00E+00	0.83	19.68	1.11E-01	Inf	NA	NA	NA
304	1.00E+00	0.83	20.88	9.06E-02	Inf	NA	NA	NA
315	1.00E+00	0.69	20.88	9.06E-02	Inf	NA	NA	NA
317	1.00E+00	0.59	18.34	1.79E-01	Inf	NA	NA	NA
31	1.00E+00	0.69	9.84	3.72E-02	Inf	NA	NA	NA
328	1.00E+00	0.59	5.47	1.61E-01	Inf	NA	NA	NA
331	1.00E+00	0.75	19.85	2.03E-01	8.34	NA	NA	NA
333	1.00E+00	1.04	9.84	1.07E-01	13.08	NA	NA	NA
336	1.00E+00	0.83	4.97	1.85E-01	Inf	NA	NA	NA
337	1.00E+00	0.69	13.01	1.66E-01	Inf	NA	NA	NA
343	1.00E+00	0.92	16.34	5.66E-03	Inf	NA	NA	NA
351	1.00E+00	0.94	11.52	5.24E-02	7.90	NA	NA	NA
365	1.00E+00	0.69	11.42	7.44E-02	Inf	NA	NA	NA
36	1.00E+00	0.69	13.18	1.85E-01	Inf	MEIS1BHOXA9_02	1.48E-04	49.66
370	1.00E+00	0.89	7.01	2.32E-02	20.13	NA	NA	NA
382	1.00E+00	0.69	12.89	1.53E-01	Inf	NA	NA	NA
399	1.00E+00	0.59	5.98	1.36E-01	Inf	GR_Q6	4.82E-04	26.76
406	1.00E+00	1.04	12.94	1.59E-01	Inf	NA	NA	NA
40	1.00E+00	1.04	7.18	8.13E-02	Inf	NA	NA	NA
41	1.00E+00	0.69	19.92	1.07E-01	Inf	NA	NA	NA
423	1.00E+00	0.89	19.13	5.27E-02	12.41	ZID_01	5.17E-04	13.51
424	1.00E+00	0.59	12.60	1.43E-01	Inf	NA	NA	NA
429	1.00E+00	1.04	10.80	5.40E-02	Inf	NA	NA	NA
42	1.00E+00	0.59	18.77	1.57E-01	Inf	NA	NA	NA
431	1.00E+00	1.01	12.55	3.51E-03	4.91	NRF2_01	3.66E-04	5.11
433	1.00E+00	0.59	13.18	1.85E-01	Inf	NA	NA	NA
434	1.00E+00	0.83	23.42	1.03E-01	8.09	NA	NA	NA
439	1.00E+00	0.52	12.94	1.59E-01	Inf	ELK1_02	6.39E-04	29.80
43	1.00E+00	1.04	12.34	2.36E-01	6.96	CETS168_Q6	5.71E-04	25.21
447	1.00E+00	0.59	18.10	1.90E-01	Inf	NA	NA	NA
450	1.00E+00	1.04	3.72	5.45E-02	Inf	PAX5_02	6.36E-20	176.08

Table S8: Enrichment of co-expressed genes in circadian genes, circadian phase, and TF binding sites.

ClusterID	Circadian Gene Enrich p value	Circadian Gene Enrich Odd Ratio	Enriched Pk	PK Enrich p value	Pk Enrich Odd Ratio	Matrix AC	TF Enrich p value	TF Enrich Odd Ratio
452	1.00E+00	0.59	4.34	2.03E-01	Inf	NA	NA	NA
458	1.00E+00	0.52	8.69	4.40E-02	Inf	NA	NA	NA
463	1.00E+00	0.83	8.69	1.65E-01	7.32	OCT1_01	4.65E-06	19.87
465	1.00E+00	0.83	19.51	1.15E-01	Inf	NA	NA	NA
469	1.00E+00	1.04	13.44	2.00E-01	Inf	NA	NA	NA
478	1.00E+00	1.02	5.35	1.49E-03	6.88	MYCMAX_01	5.85E-04	6.50
47	1.00E+00	0.83	23.69	2.15E-01	Inf	NA	NA	NA
481	1.00E+00	1.04	17.02	4.50E-03	15.21	E2F_01	8.72E-05	13.62
482	1.00E+00	1.00	17.26	8.90E-03	8.40	NA	NA	NA
489	1.00E+00	0.59	18.65	1.68E-01	Inf	NA	NA	NA
495	1.00E+00	0.52	18.43	1.75E-01	Inf	OCT4_01	8.05E-06	48.96
55	1.00E+00	1.04	7.13	1.64E-01	10.72	NA	NA	NA
57	1.00E+00	1.04	20.86	1.76E-01	9.86	NA	NA	NA
58	1.00E+00	1.04	18.67	1.64E-01	Inf	NA	NA	NA
61	1.00E+00	0.52	5.23	1.77E-01	Inf	NA	NA	NA
65	1.00E+00	0.59	9.84	3.72E-02	Inf	NA	NA	NA
67	1.00E+00	0.83	17.69	1.10E-01	7.72	ICSBP_Q6	4.74E-04	14.69
69	1.00E+00	0.69	18.10	3.61E-02	Inf	NRF2_01	2.25E-04	18.54
72	1.00E+00	1.04	9.84	7.30E-02	26.09	IRF7_01	2.05E-04	44.36

clusterID: ID of the cluster

circadian gene enrich p value: Enrichment p value of circadian gene in the cluster

circadian gene enrich odd ratio: Enrichment odd ratios of circadian genes

enriched pk Enriched peak time

PK enrich p value: Enrichment p value of peak time

PK enrich odd ratio: Enrichment odd ratios of peak time

matrix ac: TRANSFAC TF matrix accession number

TF enrich p value: Enrichment p value of TF binding sites in the cluster

TF enrich odd ratio: Enrichment odd ratios of TF binding sites in the cluster
