# Supplementary material for: Analysis of a Gene Regulatory Cascade Mediating Circadian Rhythm in Zebrafish
Source: PLoS Comput Biol. 2013 Feb 28;9(2):e1002940. doi: 10.1371/journal.pcbi.1002940 (PMC3585402; doi:10.1371/journal.pcbi.1002940)
Supplement: Table S9 — Up/Down regulated genes of mitfa KD. (PDF) [file pcbi.1002940.s014.pdf]

Table S9: Up/Down regulated genes of mitfa KD.

Gene ID	Gene Symbol	Pvalue	diff_KO_WT	UP/Down Regulated
30188	ccne	3.00E-02	-0.52	Up Regulated
30228	pes	1.31E-02	-1.06	Up Regulated
30260	flh	2.26E-02	-0.51	Up Regulated
30289	her3	1.00E-02	-1.02	Up Regulated
30300	her2	2.74E-02	-0.97	Up Regulated
30304	oep	2.57E-02	-0.59	Up Regulated
30354	lpl	2.12E-02	-0.57	Up Regulated
30449	hsp47	7.37E-05	-0.58	Up Regulated
30476	wif1	9.22E-04	-0.52	Up Regulated
30483	gata4	3.74E-04	-0.66	Up Regulated
30590	tp53	1.39E-02	-2.39	Up Regulated
30632	bmp2b	1.52E-02	-0.52	Up Regulated
30637	mdm2	3.64E-03	-2.08	Up Regulated
30649	psme3	2.04E-02	-0.53	Up Regulated
30710	igf2a	2.71E-02	-0.88	Up Regulated
57933	caspa	9.78E-03	-0.53	Up Regulated
58013	noc2l	3.99E-03	-0.74	Up Regulated
58022	casp8	7.20E-03	-2.18	Up Regulated
58039	dazl	1.48E-02	-0.60	Up Regulated
58081	baxa	1.69E-03	-0.58	Up Regulated
58097	myf5	1.50E-02	-0.87	Up Regulated
60658	nos1	2.32E-02	-1.26	Up Regulated
64692	zp3b	7.37E-03	-0.83	Up Regulated
114404	cx44.2	1.92E-02	-0.54	Up Regulated
118437	gpc4	3.89E-03	-0.51	Up Regulated
140423	jag1b	3.74E-03	-0.59	Up Regulated
171473	ccng1	7.18E-03	-1.47	Up Regulated
171480	crabp2a	1.26E-02	-0.58	Up Regulated
192312	mak16	2.96E-03	-0.78	Up Regulated
252845	mpp5a	6.66E-03	-0.68	Up Regulated
252851	mycn	2.06E-02	-0.61	Up Regulated
266985	npm1	1.87E-03	-0.97	Up Regulated
266987	cbsb	1.37E-02	-0.60	Up Regulated
266990	pwp2h	3.89E-03	-0.65	Up Regulated
282678	timm9	1.86E-02	-0.59	Up Regulated
286745	ddx55	5.01E-04	-0.66	Up Regulated
286746	sdad1	1.22E-03	-0.84	Up Regulated
286778	c1orf109	1.61E-03	-0.51	Up Regulated
317638	igfbp1a	1.13E-02	-0.82	Up Regulated
317639	tsr2	4.87E-03	-0.74	Up Regulated
317644	mki67ip	1.42E-03	-0.71	Up Regulated
317739	ppan	6.42E-04	-0.61	Up Regulated
321058	wdr3	2.73E-03	-0.68	Up Regulated
321114	nhp2l1	1.04E-02	-0.54	Up Regulated
321127	ddx18	1.40E-02	-0.63	Up Regulated
321130	si:dkey-91i17.1	2.28E-02	-0.86	Up Regulated
321133	nop14	1.92E-02	-0.53	Up Regulated
321183	si:dkey-236e20.6	1.34E-02	-0.50	Up Regulated
321277	mybbp1a	2.81E-03	-0.78	Up Regulated
321289	noc3l	1.56E-02	-0.66	Up Regulated
321442	nppa	2.50E-02	-0.62	Up Regulated
321504	ssb	3.99E-03	-0.51	Up Regulated
321652	wdr12	3.24E-03	-0.79	Up Regulated
321700	zgc:109901	8.44E-03	-0.67	Up Regulated
321720	nt5c3	6.18E-03	-0.64	Up Regulated

Table S9: Up/Down regulated genes of mitfa KD.

Gene ID	Gene Symbol	Pvalue	diff_KO_WT	UP/Down Regulated
321745	wdr36	1.93E-03	-0.69	Up Regulated
321875	rchy1	1.52E-04	-0.50	Up Regulated
321892	ckmt1	7.03E-04	-1.16	Up Regulated
321897	gnl3	7.20E-04	-0.66	Up Regulated
321974	prmt1	9.90E-03	-0.55	Up Regulated
321985	wu:fb40b03	2.01E-02	-0.61	Up Regulated
322229	adkb	2.95E-02	-0.53	Up Regulated
322251	rpl7l1	2.66E-04	-0.59	Up Regulated
322660	upb1	1.72E-04	-1.07	Up Regulated
322739	wu:fb72c11	7.49E-03	-0.53	Up Regulated
323016	exosc8	1.07E-03	-0.73	Up Regulated
323052	zgc:165515	1.05E-02	-0.53	Up Regulated
323355	drg2	7.80E-04	-0.53	Up Regulated
323424	sult1st1	2.82E-03	-0.71	Up Regulated
323426	mphosph10	4.13E-03	-0.74	Up Regulated
323462	pa2g4b	1.65E-03	-0.54	Up Regulated
323465	vcanb	2.07E-02	-0.62	Up Regulated
323961	wu:fc15e02	1.06E-02	-0.52	Up Regulated
324052	c1galt1c1	1.18E-02	-0.62	Up Regulated
324079	adi1	4.97E-03	-0.57	Up Regulated
324148	wu:fc20c04	4.02E-03	-0.52	Up Regulated
324212	si:ch73-73d17.3	2.31E-02	-0.54	Up Regulated
324303	slc39a6	1.80E-02	-0.55	Up Regulated
324319	wu:fc25e04	3.80E-03	-0.54	Up Regulated
324426	si:ch211-42i9.5	1.34E-02	-0.57	Up Regulated
324573	ogfod1	7.29E-05	-0.52	Up Regulated
324944	magt1	6.53E-04	-0.53	Up Regulated
325603	polr1b	8.03E-03	-0.66	Up Regulated
326102	hhip	6.23E-03	-0.65	Up Regulated
326761	lnpa	1.35E-02	-0.59	Up Regulated
326846	zff9	8.97E-04	-0.59	Up Regulated
327053	rrp15	1.40E-02	-0.53	Up Regulated
327057	uck2b	5.55E-03	-0.52	Up Regulated
327078	polr1a	4.95E-03	-0.61	Up Regulated
327082	napa	2.01E-02	-0.51	Up Regulated
327335	srp72	7.07E-03	-0.62	Up Regulated
327377	tuba4l	2.16E-02	-0.82	Up Regulated
327403	h1m	1.20E-02	-0.64	Up Regulated
327494	zgc:66472	4.44E-03	-0.52	Up Regulated
334055	taf5l	2.26E-02	-0.53	Up Regulated
334271	zgc:171476	2.26E-02	-0.90	Up Regulated
334315	orc3	6.22E-04	-0.73	Up Regulated
334393	iars	5.43E-03	-0.72	Up Regulated
334446	heatr1	7.09E-03	-0.81	Up Regulated
334724	anxa1a	2.85E-02	-0.88	Up Regulated
334823	wu:fa04e07	1.62E-02	-0.50	Up Regulated
334936	trmt2a	3.79E-03	-0.56	Up Regulated
335049	ebna1bp2	1.05E-03	-0.62	Up Regulated
335270	wu:fl03e01	1.26E-03	-0.57	Up Regulated
335288	gpr34a	1.61E-02	-0.60	Up Regulated
335411	si:dkeyp-46h3.2	1.69E-02	-1.04	Up Regulated
335495	parp3	4.90E-04	-1.01	Up Regulated
335750	zgc:77551	2.68E-03	-0.53	Up Regulated
336149	utp11l	4.53E-03	-0.53	Up Regulated
336442	wu:fb10g03	7.43E-03	-0.72	Up Regulated

Table S9: Up/Down regulated genes of mitfa KD.

Gene ID	Gene Symbol	Pvalue	diff_KO_WT	UP/Down Regulated
336470	zgc:56683	1.56E-03	-0.83	Up Regulated
336764	rps15a	1.57E-02	-0.75	Up Regulated
336984	dnajc21	2.96E-03	-0.70	Up Regulated
336998	chek2	1.31E-02	-0.52	Up Regulated
337046	ranbp1	1.90E-03	-0.63	Up Regulated
337155	nap1l4a	3.28E-04	-0.70	Up Regulated
337179	mmp2	7.30E-04	-0.76	Up Regulated
337214	zgc:66430	3.82E-03	-0.84	Up Regulated
337399	rrs1	7.46E-03	-0.51	Up Regulated
337461	zgc:65979	4.57E-03	-0.53	Up Regulated
337487	pus3	7.12E-03	-0.74	Up Regulated
338230	unc119.1	2.38E-03	-0.59	Up Regulated
338277	id:ibd1090	5.95E-03	-0.57	Up Regulated
338290	hmbsb	1.91E-02	-0.71	Up Regulated
352916	sepx1a	1.56E-02	-0.62	Up Regulated
353223	tshb	1.28E-03	-0.82	Up Regulated
353313	foxi1	4.18E-03	-1.27	Up Regulated
368201	ved	1.72E-03	-0.67	Up Regulated
368235	yars	2.59E-03	-0.61	Up Regulated
368253	pinx1	1.56E-02	-0.82	Up Regulated
368270	sult1st3	3.49E-03	-0.65	Up Regulated
368478	wdr75	1.22E-03	-0.72	Up Regulated
368631	pms1	6.26E-03	-0.57	Up Regulated
368779	phlda3	6.32E-04	-2.16	Up Regulated
368824	ccdc65	2.29E-02	-0.90	Up Regulated
368856	cenpn	4.18E-03	-0.59	Up Regulated
368857	si:busm1-241h12.4	6.51E-03	-0.54	Up Regulated
368909	rrm2b	7.89E-03	-2.68	Up Regulated
373084	hig1	8.28E-03	-0.69	Up Regulated
373085	hspa9	6.14E-03	-0.59	Up Regulated
378722	rdh10b	1.16E-02	-0.52	Up Regulated
378844	ddx27	9.91E-04	-0.61	Up Regulated
378855	slmo2	1.17E-03	-0.59	Up Regulated
386639	zcchc9	2.30E-03	-0.55	Up Regulated
386642	ngdn	2.73E-04	-0.71	Up Regulated
386850	eif6	1.47E-03	-0.71	Up Regulated
386932	poc1b	5.57E-03	-0.64	Up Regulated
387255	rbm19	3.47E-03	-0.64	Up Regulated
387261	baiap2l1a	7.54E-03	-0.52	Up Regulated
393095	mocs3	1.19E-02	-0.83	Up Regulated
393106	rad18	7.74E-04	-0.60	Up Regulated
393109	rasl11b	2.50E-03	-0.85	Up Regulated
393117	ccdc94	6.07E-04	-0.52	Up Regulated
393143	umps	9.58E-03	-0.84	Up Regulated
393165	zgc:56116	4.72E-04	-0.56	Up Regulated
393173	zgc:56178	1.62E-03	-0.52	Up Regulated
393176	zgc:56197	2.97E-02	-0.51	Up Regulated
393185	trmt11	6.74E-03	-0.63	Up Regulated
393192	zgc:56288	2.31E-02	-0.61	Up Regulated
393206	nol10	1.16E-02	-0.60	Up Regulated
393222	zgc:55343	3.93E-03	-0.57	Up Regulated
393224	snapc1b	1.21E-04	-0.54	Up Regulated
393236	sybl1	3.92E-03	-0.75	Up Regulated
393237	imp3	1.88E-04	-0.65	Up Regulated
393241	rnaseh2b	1.79E-03	-0.67	Up Regulated

Table S9: Up/Down regulated genes of mitfa KD.

Gene ID	Gene Symbol	Pvalue	diff_KO_WT	UP/Down Regulated
393243	zgc:56688	6.67E-04	-0.84	Up Regulated
393257	zgc:56407	2.57E-04	-0.55	Up Regulated
393260	zgc:56457	2.77E-03	-0.77	Up Regulated
393277	mrpl45	3.31E-04	-0.54	Up Regulated
393308	minal	2.73E-02	-0.66	Up Regulated
393349	zgc:64116	1.44E-03	-0.78	Up Regulated
393370	pdcd2	1.80E-02	-0.52	Up Regulated
393371	dnajc5gb	2.48E-02	-0.69	Up Regulated
393393	zgc:64213	1.47E-03	-0.70	Up Regulated
393397	lsm14aa	2.96E-02	-0.51	Up Regulated
393404	zgc:63466	2.41E-03	-0.54	Up Regulated
393409	zgc:63572	2.28E-02	-0.51	Up Regulated
393429	tmem206	1.63E-03	-0.55	Up Regulated
393482	tm9sf4	5.17E-03	-0.73	Up Regulated
393511	stc1l	1.37E-02	-0.76	Up Regulated
393526	mtap	5.75E-03	-0.60	Up Regulated
393538	polr1c	3.44E-04	-0.70	Up Regulated
393548	gadd45aa	5.10E-04	-1.21	Up Regulated
393596	wdr46	2.43E-02	-0.51	Up Regulated
393604	srfl	2.24E-02	-0.77	Up Regulated
393650	irf7	1.92E-04	-0.81	Up Regulated
393653	rhoga	2.08E-03	-0.62	Up Regulated
393654	slc5a1	2.17E-02	-1.57	Up Regulated
393713	fhit	1.03E-03	-0.92	Up Regulated
393717	smfn	1.23E-04	-0.54	Up Regulated
393744	zgc:73273	1.69E-03	-0.75	Up Regulated
393749	tfdp1b	1.71E-02	-0.51	Up Regulated
393780	ccdc58	2.24E-05	-0.65	Up Regulated
393787	pcbd1	6.14E-04	-0.50	Up Regulated
393813	pdlim7	8.31E-03	-0.61	Up Regulated
393863	heatr3	9.17E-04	-0.60	Up Regulated
393864	naprt1	2.44E-03	-0.51	Up Regulated
393981	ttc4	1.56E-02	-0.70	Up Regulated
393982	alp	3.93E-03	-0.56	Up Regulated
393984	aacs	5.47E-03	-0.66	Up Regulated
394000	pgm1	1.57E-02	-0.56	Up Regulated
394021	shmt1	1.69E-02	-1.25	Up Regulated
394040	surf6	8.66E-03	-0.67	Up Regulated
394064	zgc:55695	1.08E-02	-0.52	Up Regulated
394086	zgc:66419	4.28E-03	-0.58	Up Regulated
394129	hmbsa	1.66E-02	-0.62	Up Regulated
394138	asns	9.56E-03	-0.83	Up Regulated
394145	vrk2	6.05E-04	-1.70	Up Regulated
394150	oxsr1b	3.99E-03	-0.77	Up Regulated
394160	bokb	1.98E-02	-1.16	Up Regulated
394185	pufa	4.85E-04	-0.73	Up Regulated
394188	qars	2.57E-02	-0.55	Up Regulated
394189	eif3ja	1.19E-02	-0.60	Up Regulated
394196	nop56	5.31E-03	-0.54	Up Regulated
402798	qtrtd1	2.41E-03	-0.66	Up Regulated
402800	kri1l	1.24E-02	-0.53	Up Regulated
402802	zgc:162119	1.97E-02	-0.72	Up Regulated
402808	rrp9	4.14E-03	-0.83	Up Regulated
402856	twistnb	2.47E-03	-0.75	Up Regulated
402862	cdkn1bl	2.88E-02	-0.60	Up Regulated

Table S9: Up/Down regulated genes of mitfa KD.

Gene ID	Gene Symbol	Pvalue	diff_KO_WT	UP/Down Regulated
402863	gtpbp1l	5.42E-04	-1.39	Up Regulated
402871	gstcd	1.10E-02	-0.60	Up Regulated
402880	LOC402880	1.05E-02	-1.46	Up Regulated
402937	mgp	4.32E-03	-0.69	Up Regulated
402976	si:dkey-194e6.1	1.13E-03	-0.72	Up Regulated
402995	pus7	1.98E-02	-0.60	Up Regulated
403013	irf9	3.58E-03	-0.54	Up Regulated
404619	n6amt2	3.50E-04	-0.64	Up Regulated
404725	cx30.3	1.74E-02	-0.56	Up Regulated
405787	fibpl	5.73E-03	-0.64	Up Regulated
405812	cyb5b	6.23E-03	-0.90	Up Regulated
405828	nup43	2.53E-03	-0.63	Up Regulated
405867	zgc:85909	8.25E-04	-0.67	Up Regulated
405874	zgc:85939	1.55E-03	-0.72	Up Regulated
406311	zgc:73230	8.94E-03	-0.54	Up Regulated
406342	zgc:55891	2.19E-02	-1.46	Up Regulated
406382	znf259	1.68E-03	-0.63	Up Regulated
406457	rcc1	8.00E-03	-0.58	Up Regulated
406479	orc5	2.79E-04	-0.88	Up Regulated
406486	m6pr	5.37E-03	-0.57	Up Regulated
406571	cirh1a	1.25E-03	-0.94	Up Regulated
406630	zgc:77499	2.16E-02	-0.63	Up Regulated
406633	tesc	1.13E-02	-0.99	Up Regulated
406637	mre11a	3.11E-05	-0.61	Up Regulated
406721	rad51l1	3.45E-03	-0.70	Up Regulated
406741	e2f4	2.35E-02	-0.55	Up Regulated
406746	fpgs	1.39E-02	-0.70	Up Regulated
406805	nvl	1.41E-02	-0.53	Up Regulated
406831	gyg1	8.74E-04	-0.80	Up Regulated
406840	sesn3	5.65E-03	-1.06	Up Regulated
407084	riok2	1.37E-02	-0.54	Up Regulated
407612	tmem106a	8.75E-03	-0.67	Up Regulated
407635	ptpn3	1.12E-02	-0.60	Up Regulated
407655	zfpm1	9.52E-03	-0.50	Up Regulated
407673	rpia	1.40E-02	-0.76	Up Regulated
407696	ddx52	1.12E-03	-0.73	Up Regulated
407736	fabp7b	1.72E-03	-0.89	Up Regulated
415150	eif2b1	7.27E-04	-0.56	Up Regulated
415191	tnni1al	1.25E-03	-0.79	Up Regulated
415200	zgc:86839	4.54E-03	-0.65	Up Regulated
415216	zgc:86776	7.58E-03	-0.55	Up Regulated
415234	med22	1.52E-02	-0.52	Up Regulated
415236	zgc:86715	7.97E-03	-0.69	Up Regulated
415238	rpp40l	5.04E-03	-0.89	Up Regulated
415250	rpf1	3.24E-04	-0.70	Up Regulated
432386	mgc35261l	1.30E-03	-0.66	Up Regulated
436587	ltv1	7.90E-04	-0.60	Up Regulated
436605	stx5a	1.41E-03	-0.71	Up Regulated
436625	rspo1	3.56E-03	-2.67	Up Regulated
436691	zgc:92670	1.93E-03	-0.57	Up Regulated
436758	zgc:92862	4.68E-04	-0.53	Up Regulated
436771	hccsa	8.60E-03	-0.73	Up Regulated
436779	lyrm1	2.81E-04	-1.59	Up Regulated
436787	dusp22a	1.67E-02	-0.78	Up Regulated
436798	rpp38	9.47E-03	-0.54	Up Regulated

Table S9: Up/Down regulated genes of mitfa KD.

Gene ID	Gene Symbol	Pvalue	diff_KO_WT	UP/Down Regulated
436807	nop16	1.48E-03	-0.62	Up Regulated
436808	rwdd1	1.13E-03	-0.58	Up Regulated
436826	polr3k	1.77E-03	-0.58	Up Regulated
436841	gc	2.86E-02	-0.56	Up Regulated
436845	zgc:92746	2.01E-03	-0.72	Up Regulated
436890	tspan33	5.87E-05	-0.68	Up Regulated
436892	nup37	1.55E-02	-0.55	Up Regulated
436936	jmjd5	1.79E-04	-0.55	Up Regulated
437005	zgc:100799	1.24E-02	-1.10	Up Regulated
442932	gnl3l	1.19E-03	-0.77	Up Regulated
445058	tipin	2.74E-03	-0.80	Up Regulated
445082	dnajc15	4.69E-03	-0.51	Up Regulated
445101	zgc:92006	5.16E-03	-0.50	Up Regulated
445104	zgc:91999	2.52E-02	-0.88	Up Regulated
445115	grwd1	8.69E-04	-0.67	Up Regulated
445144	chchd4	3.16E-03	-0.61	Up Regulated
445161	cenpp	5.84E-03	-0.60	Up Regulated
445162	dimt1l	2.57E-02	-0.51	Up Regulated
445166	ctu2	4.88E-03	-0.52	Up Regulated
445173	zgc:101084	9.74E-03	-0.69	Up Regulated
445243	timm8a	1.89E-03	-0.96	Up Regulated
445292	sgcg	1.31E-02	-0.69	Up Regulated
445306	eif3ha	2.49E-03	-0.53	Up Regulated
445385	rfc5	3.96E-03	-0.64	Up Regulated
445388	rcl1	3.33E-03	-0.74	Up Regulated
445389	gtf3aa	1.32E-04	-1.29	Up Regulated
445390	mybl2	2.31E-02	-0.86	Up Regulated
445394	wdr55	2.02E-03	-0.70	Up Regulated
445399	ddx56	3.86E-03	-0.61	Up Regulated
445406	ect2	2.92E-02	-0.61	Up Regulated
445412	polr1d	3.59E-04	-0.83	Up Regulated
445499	tnpo2	1.21E-02	-0.60	Up Regulated
445567	st3gal2l	2.23E-02	-0.79	Up Regulated
447811	rassf1	1.24E-03	-0.54	Up Regulated
447856	zgc:92127	1.81E-02	-0.60	Up Regulated
447863	bin1	1.09E-02	-0.51	Up Regulated
447868	zgc:92599	1.55E-02	-0.64	Up Regulated
447872	dscc1	9.67E-03	-0.56	Up Regulated
447875	smyd5	1.65E-02	-0.80	Up Regulated
447888	zgc:101748	5.69E-04	-0.57	Up Regulated
447896	si:ch211-217g15.2	1.99E-02	-0.58	Up Regulated
447901	polr3gla	5.45E-03	-0.89	Up Regulated
447909	tgm2	1.45E-03	-0.56	Up Regulated
447914	cox17	8.77E-05	-0.72	Up Regulated
447933	uxt	5.41E-03	-0.54	Up Regulated
449553	zgc:103672	5.31E-03	-0.61	Up Regulated
449670	srl	2.41E-02	-0.59	Up Regulated
449825	zgc:101560	8.68E-03	-0.57	Up Regulated
449833	zdhhc15a	1.49E-02	-0.65	Up Regulated
449857	si:dkey-145p14.5	2.03E-03	-1.42	Up Regulated
449954	si:ch73-37h15.2	1.31E-03	-0.63	Up Regulated
450000	amt	1.83E-02	-0.51	Up Regulated
450020	cd151	5.60E-03	-0.53	Up Regulated
450024	cited2	3.22E-04	-0.78	Up Regulated
450027	zgc:101814	1.80E-03	-0.52	Up Regulated

Table S9: Up/Down regulated genes of mitfa KD.

Gene ID	Gene Symbol	Pvalue	diff_KO_WT	UP/Down Regulated
450030	zgc:101803	2.35E-03	-0.98	Up Regulated
492339	creg2	1.14E-02	-0.57	Up Regulated
492353	zgc:92172	1.44E-02	-0.55	Up Regulated
492478	slc25a44a	2.83E-02	-0.50	Up Regulated
492480	rnf24	1.35E-02	-0.60	Up Regulated
492485	lrrc20	1.34E-02	-0.54	Up Regulated
492514	dph5	1.28E-03	-0.75	Up Regulated
492754	im:7154516	1.61E-02	-0.58	Up Regulated
492761	tbl3	6.87E-03	-0.55	Up Regulated
492784	zgc:101716	4.07E-03	-0.74	Up Regulated
492814	zgc:101609	1.48E-02	-0.51	Up Regulated
493608	farsb	1.62E-02	-0.56	Up Regulated
493915	atoh1b	2.52E-02	-0.75	Up Regulated
494040	cd2apl	2.33E-02	-0.51	Up Regulated
494059	zgc:103536	4.88E-04	-0.54	Up Regulated
494105	foxj1b	3.96E-03	-0.81	Up Regulated
494106	nsun4	4.87E-03	-0.62	Up Regulated
497181	tomm5	3.98E-03	-0.50	Up Regulated
497333	im:7136639	2.30E-02	-0.58	Up Regulated
503593	mrpl48	1.13E-04	-0.58	Up Regulated
503604	zgc:113138	1.03E-02	-0.60	Up Regulated
503606	zgc:110753	1.63E-03	-0.59	Up Regulated
503742	zgc:113334	3.95E-03	-0.72	Up Regulated
503748	rfc2	7.81E-03	-0.59	Up Regulated
503751	zgc:110848	3.53E-03	-0.77	Up Regulated
503752	ptrh1	1.20E-02	-0.68	Up Regulated
503758	slc25a32b	1.61E-03	-0.57	Up Regulated
503762	zgc:113036	4.48E-04	-0.71	Up Regulated
503783	metap1	6.04E-03	-0.54	Up Regulated
541317	si:ch211-150a22.1	2.36E-02	-0.59	Up Regulated
541336	zgc:112496	2.81E-03	-0.62	Up Regulated
541344	zgc:110343	1.96E-02	-0.89	Up Regulated
541352	rpp30	1.46E-03	-0.73	Up Regulated
541404	cdc42ep2	2.27E-02	-0.66	Up Regulated
541413	slc39a9	2.65E-02	-0.61	Up Regulated
541428	znrd1	6.96E-04	-0.61	Up Regulated
541448	zgc:113346	1.79E-02	-0.92	Up Regulated
541489	acp1	2.90E-02	-0.59	Up Regulated
550264	fcf1	2.09E-03	-0.57	Up Regulated
550279	lypla1	2.36E-02	-0.76	Up Regulated
550284	wdr92	2.64E-03	-0.52	Up Regulated
550328	park2	7.65E-03	-0.54	Up Regulated
550343	si:dkey-22a1.3	4.46E-03	-0.63	Up Regulated
550348	prmt3	1.68E-03	-0.72	Up Regulated
550349	pdss1	5.98E-04	-0.53	Up Regulated
550350	zgc:110388	2.52E-03	-0.85	Up Regulated
550358	zgc:112052	2.63E-04	-0.51	Up Regulated
550359	morn3	1.06E-02	-0.77	Up Regulated
550391	mrpl12	5.97E-03	-0.53	Up Regulated
550399	grem2	4.55E-03	-0.65	Up Regulated
550440	zgc:112104	4.44E-03	-0.51	Up Regulated
550455	mrps18b	2.48E-04	-0.54	Up Regulated
550466	hmgb3b	1.30E-04	-0.51	Up Regulated
550481	rhoub	1.60E-02	-0.94	Up Regulated
550514	utp23	7.28E-03	-0.63	Up Regulated

Table S9: Up/Down regulated genes of mitfa KD.

Gene ID	Gene Symbol	Pvalue	diff_KO_WT	UP/Down Regulated
550525	zgc:110197	1.14E-02	-0.58	Up Regulated
550549	eif4e1c	1.19E-02	-0.53	Up Regulated
550559	dph1	9.83E-03	-0.54	Up Regulated
550561	ppp1r14aa	2.29E-03	-0.56	Up Regulated
550569	myl10	5.63E-04	-0.85	Up Regulated
550570	itprip	4.46E-03	-0.99	Up Regulated
550574	zgc:113106	3.29E-03	-1.14	Up Regulated
550579	zgc:113076	4.38E-04	-0.60	Up Regulated
550580	sqrdl	9.29E-04	-0.72	Up Regulated
550612	zgc:113229	2.26E-02	-1.06	Up Regulated
552924	slc2a15b	2.69E-02	-0.75	Up Regulated
553083	rbbp9	9.29E-04	-0.50	Up Regulated
553297	cfh	2.64E-02	-1.31	Up Regulated
553298	im:7150454	1.24E-03	-1.03	Up Regulated
553325	ptcd3	1.87E-03	-0.53	Up Regulated
553438	LOC553438	2.26E-02	-0.68	Up Regulated
553483	haus7	1.27E-02	-0.51	Up Regulated
553492	LOC553492	2.13E-02	-0.98	Up Regulated
553512	slc27a1b	2.12E-02	-0.54	Up Regulated
553527	LOC553527	4.71E-03	-0.90	Up Regulated
553568	zgc:109995	4.23E-03	-0.90	Up Regulated
553571	tp53rk	6.30E-04	-0.55	Up Regulated
553592	tatdn3	2.35E-02	-0.54	Up Regulated
553606	nle1	8.66E-03	-0.80	Up Regulated
553616	nip7	1.64E-03	-0.88	Up Regulated
553622	phlda2	4.30E-05	-1.08	Up Regulated
553636	trnau1ap	6.98E-03	-0.58	Up Regulated
553655	pygma	1.62E-03	-0.62	Up Regulated
553657	rab12	3.96E-04	-0.71	Up Regulated
553671	zgc:112030	5.41E-03	-0.53	Up Regulated
553673	zgc:112038	1.30E-02	-0.75	Up Regulated
553695	zgc:112255	1.48E-02	-0.63	Up Regulated
553697	fam100ab	2.49E-02	-0.65	Up Regulated
553698	zgc:112271	4.97E-03	-0.79	Up Regulated
553709	mrpl27	2.55E-02	-0.57	Up Regulated
553720	zgc:112365	1.39E-03	-0.52	Up Regulated
553726	zgc:112397	2.50E-02	-0.97	Up Regulated
553733	polr1e	8.44E-03	-0.74	Up Regulated
553748	zgc:113276	8.14E-03	-0.98	Up Regulated
553791	prep	1.75E-02	-0.68	Up Regulated
553795	zgc:111947	2.12E-02	-0.73	Up Regulated
553808	ankrd45	1.08E-03	-0.65	Up Regulated
554096	noc4l	2.03E-05	-0.82	Up Regulated
554117	morn5	2.24E-02	-0.57	Up Regulated
554231	zgc:109926	2.01E-02	-0.55	Up Regulated
555238	pcgf6	1.88E-02	-0.67	Up Regulated
555267	si:zfos-47c12.1	1.83E-02	-0.88	Up Regulated
555328	zgc:171630	6.14E-03	-0.88	Up Regulated
555517	kat2a	1.30E-02	-0.52	Up Regulated
555570	si:ch211-218c6.5	1.24E-02	-0.69	Up Regulated
555604	nox1	2.56E-02	-0.88	Up Regulated
555753	zgc:113389	2.59E-02	-0.55	Up Regulated
555804	wu:fk76c08	1.61E-02	-0.73	Up Regulated
555824	LOC555824	2.95E-02	-0.68	Up Regulated
555828	LOC555828	2.71E-02	-0.90	Up Regulated

Table S9: Up/Down regulated genes of mitfa KD.

Gene ID	Gene Symbol	Pvalue	diff_KO_WT	UP/Down Regulated
555835	si:ch211-225p5.3	1.05E-04	-1.21	Up Regulated
555926	lace1b	7.75E-03	-0.60	Up Regulated
555997	si:ch211-284b7.3	2.91E-04	-0.55	Up Regulated
556208	akap1b	1.27E-02	-0.66	Up Regulated
556226	cdc42l2	5.41E-03	-0.76	Up Regulated
556362	LOC556362	6.22E-03	-0.53	Up Regulated
556389	fxn	1.88E-03	-0.72	Up Regulated
556493	ncapd3	5.30E-03	-0.95	Up Regulated
556515	LOC556515	6.32E-03	-0.50	Up Regulated
556702	wu:fi41d10	2.22E-05	-1.60	Up Regulated
556728	crfb8	3.00E-02	-0.86	Up Regulated
557052	hfm1	5.73E-03	-0.53	Up Regulated
557168	si:dkey-240e12.6	4.22E-03	-0.83	Up Regulated
557254	wu:fc27e05	9.59E-03	-0.65	Up Regulated
557725	zgc:154110	1.25E-03	-0.57	Up Regulated
557741	si:dkey-14d8.6	2.60E-02	-0.50	Up Regulated
557849	im:7159098	2.00E-03	-0.52	Up Regulated
557881	dok7	2.75E-02	-0.53	Up Regulated
557892	LOC557892	2.75E-02	-0.60	Up Regulated
558036	lmx1a	2.14E-02	-0.95	Up Regulated
558146	mfsd4b	6.89E-03	-0.88	Up Regulated
558373	extl2	1.84E-02	-1.03	Up Regulated
558421	si:ch211-173p18.9	8.09E-03	-0.68	Up Regulated
558500	oxnad1	2.43E-02	-0.72	Up Regulated
558534	si:dkey-42i9.5	1.21E-03	-0.78	Up Regulated
558677	zp3a.2	2.90E-02	-1.07	Up Regulated
558684	b4galnt1a	2.53E-02	-0.60	Up Regulated
558764	zgc:101633	2.99E-02	-0.77	Up Regulated
558911	wu:fa99c08	9.92E-04	-0.71	Up Regulated
558956	isg15	2.04E-02	-2.58	Up Regulated
559192	LOC559192	8.77E-03	-0.70	Up Regulated
559352	znf593	9.68E-04	-0.64	Up Regulated
559409	si:ch211-217k17.7	4.04E-04	-0.94	Up Regulated
559477	aatf	4.29E-03	-0.74	Up Regulated
559754	tmprss13a	2.68E-02	-0.64	Up Regulated
560202	ghrb	2.93E-02	-0.52	Up Regulated
560402	zgc:165500	2.18E-02	-0.53	Up Regulated
560478	slc25a26	2.28E-03	-0.65	Up Regulated
560620	hemk1	6.83E-03	-0.56	Up Regulated
560648	si:ch211-81a5.8	2.08E-03	-1.05	Up Regulated
560651	ces3	1.15E-03	-0.54	Up Regulated
560729	ppp6r2	1.07E-02	-0.66	Up Regulated
561131	nolc1	2.38E-02	-0.61	Up Regulated
561226	lnx1	7.04E-03	-2.30	Up Regulated
561280	zfp36l1a	1.04E-02	-0.62	Up Regulated
561301	zgc:165651	4.80E-04	-0.54	Up Regulated
561410	tat	8.01E-04	-1.29	Up Regulated
561824	LOC561824	4.94E-03	-0.57	Up Regulated
561927	angptl6	1.45E-02	-0.67	Up Regulated
562445	arl8bb	4.31E-03	-0.65	Up Regulated
562538	zgc:162269	1.02E-03	-0.68	Up Regulated
562639	rtn2b	2.80E-03	-0.66	Up Regulated
563100	si:ch211-51h9.6	1.50E-03	-0.56	Up Regulated
563195	rrp12	2.27E-03	-0.73	Up Regulated
563247	LOC563247	1.11E-03	-1.00	Up Regulated

Table S9: Up/Down regulated genes of mitfa KD.

Gene ID	Gene Symbol	Pvalue	diff_KO_WT	UP/Down Regulated
563392	zgc:172056	6.09E-03	-0.56	Up Regulated
563398	zgc:172079	8.99E-03	-0.61	Up Regulated
563448	zgc:193690	8.87E-03	-1.03	Up Regulated
563523	LOC563523	7.29E-03	-0.55	Up Regulated
563576	si:dkey-245p14.4	1.35E-02	-0.50	Up Regulated
563675	slc35e3	2.05E-03	-0.63	Up Regulated
563708	skp2	1.19E-02	-0.81	Up Regulated
563867	si:ch211-237l4.5	9.73E-03	-0.62	Up Regulated
564078	trmt5	2.19E-03	-0.51	Up Regulated
564166	txlnbb	2.71E-02	-0.74	Up Regulated
564382	si:ch211-103i6.5	3.66E-04	-0.64	Up Regulated
564693	tmprss13b	2.35E-03	-0.85	Up Regulated
564785	LOC564785	1.26E-02	-0.60	Up Regulated
564815	slc5a9	1.78E-02	-0.68	Up Regulated
564961	dnttip2	2.98E-03	-0.93	Up Regulated
565000	exosc3	3.08E-03	-0.62	Up Regulated
565002	LOC565002	9.44E-03	-0.78	Up Regulated
565038	ccnd2b	2.35E-02	-0.74	Up Regulated
565232	hspa14	2.41E-03	-0.56	Up Regulated
565269	her8.2	4.46E-03	-0.61	Up Regulated
565410	LOC565410	2.95E-02	-0.77	Up Regulated
565437	si:dkey-221j11.2	1.97E-03	-0.73	Up Regulated
565728	si:dkey-193b15.6	2.52E-02	-1.26	Up Regulated
565828	stk33	7.96E-03	-0.62	Up Regulated
566075	slc4a2a	1.92E-02	-0.92	Up Regulated
566173	LOC566173	7.69E-03	-0.77	Up Regulated
566203	mthfsd	5.32E-03	-0.60	Up Regulated
566223	si:dkey-127j5.5	3.07E-03	-0.63	Up Regulated
566423	si:dkey-27p23.3	1.83E-02	-0.92	Up Regulated
566470	gdf6a	4.10E-03	-0.57	Up Regulated
566612	ca9	5.71E-03	-0.70	Up Regulated
566735	insb	1.50E-02	-0.76	Up Regulated
566817	LOC566817	1.30E-02	-0.66	Up Regulated
566876	wfs1a	1.44E-02	-0.74	Up Regulated
566971	LOC566971	6.73E-03	-0.58	Up Regulated
567019	ptpmt1	5.53E-03	-0.85	Up Regulated
567105	taf1a	8.38E-04	-0.83	Up Regulated
567341	si:ch211-261f7.2	8.17E-04	-0.76	Up Regulated
567483	si:dkey-22a1.2	2.35E-02	-0.91	Up Regulated
567594	LOC567594	2.29E-03	-0.86	Up Regulated
567726	si:ch211-163l21.7	7.76E-03	-0.65	Up Regulated
567941	e2f7	2.46E-02	-0.64	Up Regulated
567972	zgc:123339	1.14E-02	-0.51	Up Regulated
568202	por	1.91E-02	-0.74	Up Regulated
568363	LOC568363	1.18E-02	-0.59	Up Regulated
568418	si:dkeyp-55f12.3	6.06E-03	-0.57	Up Regulated
568463	ryr2b	1.30E-02	-0.59	Up Regulated
568593	sdc4	7.84E-03	-0.59	Up Regulated
568741	eftud1	4.76E-03	-0.76	Up Regulated
568757	si:dkey-121j17.1	2.56E-02	-0.57	Up Regulated
568788	LOC568788	2.26E-02	-0.80	Up Regulated
568835	slc27a6	1.31E-02	-0.54	Up Regulated
569076	si:dkey-181i3.3	1.32E-02	-0.56	Up Regulated
569118	ddx10	3.43E-03	-0.57	Up Regulated
569134	si:ch211-11i5.1	6.14E-03	-0.99	Up Regulated

Table S9: Up/Down regulated genes of mitfa KD.

Gene ID	Gene Symbol	Pvalue	diff_KO_WT	UP/Down Regulated
569261	pkhd1l1	1.20E-02	-0.78	Up Regulated
569275	si:ch211-199o1.5	2.14E-02	-0.63	Up Regulated
569381	LOC569381	2.13E-02	-1.53	Up Regulated
569427	LOC569427	1.75E-02	-1.51	Up Regulated
569454	crfb1	4.95E-03	-1.05	Up Regulated
569583	LOC569583	4.47E-03	-0.69	Up Regulated
569841	mrps18c	2.63E-02	-0.67	Up Regulated
569930	zgc:153896	7.04E-03	-1.62	Up Regulated
570158	alkbh5	1.38E-02	-0.56	Up Regulated
570159	zgc:123271	2.20E-02	-0.80	Up Regulated
570267	LOC570267	1.82E-02	-0.62	Up Regulated
570432	LOC570432	6.97E-03	-1.02	Up Regulated
570726	LOC570726	8.97E-03	-0.56	Up Regulated
571152	si:dkey-258f14.3	3.01E-03	-0.94	Up Regulated
571299	nenf	9.63E-03	-0.53	Up Regulated
572027	ppat	1.52E-02	-0.51	Up Regulated
572170	mrpl9	8.89E-04	-0.66	Up Regulated
573266	dync1li2	4.66E-03	-0.58	Up Regulated
573998	dusp12	2.79E-02	-0.66	Up Regulated
574000	tnnt2d	1.23E-02	-0.71	Up Regulated
606657	trmt2b	1.14E-02	-0.82	Up Regulated
613021	si:ch211-103f16.2	6.53E-03	-0.56	Up Regulated
613239	hcrt	1.64E-02	-0.66	Up Regulated
641330	atg10	5.27E-04	-0.75	Up Regulated
641427	zgc:123272	8.34E-03	-2.28	Up Regulated
641570	impad1	1.82E-02	-0.54	Up Regulated
678516	tnfaip6	1.35E-03	-0.71	Up Regulated
678541	zgc:136861	8.72E-03	-0.69	Up Regulated
678588	zgc:136360	5.38E-03	-0.67	Up Regulated
678604	irf1	2.83E-02	-0.53	Up Regulated
678616	zgc:136403	4.55E-03	-1.26	Up Regulated
678634	zgc:136871	8.38E-04	-1.25	Up Regulated
678636	wbscr27	2.04E-02	-0.61	Up Regulated
692250	fancf	1.40E-02	-0.53	Up Regulated
692329	farsa	1.04E-02	-0.51	Up Regulated
724049	krr1	9.13E-03	-0.53	Up Regulated
751632	exosc5	1.30E-04	-0.66	Up Regulated
751646	zgc:153151	1.26E-02	-1.15	Up Regulated
751684	fbxo36	2.87E-02	-0.88	Up Regulated
751714	zgc:153631	2.70E-02	-0.80	Up Regulated
751717	zgc:153763	2.04E-03	-0.73	Up Regulated
751763	bbc3	2.35E-04	-2.23	Up Regulated
767661	zgc:153046	1.03E-02	-0.71	Up Regulated
767664	zgc:153177	5.41E-03	-0.77	Up Regulated
767671	ccdc146	1.47E-02	-0.81	Up Regulated
767699	pmt	1.34E-02	-0.60	Up Regulated
767713	zgc:154067	2.50E-03	-0.67	Up Regulated
767778	zgc:154071	1.34E-02	-0.62	Up Regulated
767793	ccdc37	2.66E-02	-0.52	Up Regulated
768123	zgc:154116	2.79E-03	-1.12	Up Regulated
768134	si:dkey-184p18.2	6.70E-03	-0.57	Up Regulated
768135	zgc:153341	8.89E-04	-0.63	Up Regulated
768152	zgc:153687	2.97E-02	-0.62	Up Regulated
768161	zgc:153041	1.58E-02	-0.54	Up Regulated
768192	zgc:153035	2.69E-02	-0.55	Up Regulated

Table S9: Up/Down regulated genes of mitfa KD.

Gene ID	Gene Symbol	Pvalue	diff_KO_WT	UP/Down Regulated
777623	zgc:154093	1.41E-02	-0.61	Up Regulated
790933	fam132a	1.35E-02	-0.53	Up Regulated
791153	ldlrap1b	1.72E-02	-0.70	Up Regulated
791187	srpk1b	1.58E-02	-0.75	Up Regulated
791212	zgc:158296	2.28E-02	-0.52	Up Regulated
791222	zgc:158636	2.71E-02	-0.53	Up Regulated
791500	zgc:153921	1.56E-02	-1.47	Up Regulated
791595	wnt11	2.01E-03	-1.33	Up Regulated
791612	zgc:110847	2.01E-02	-0.91	Up Regulated
792182	eef2a.1	9.58E-03	-0.73	Up Regulated
792919	LOC792919	4.43E-03	-1.19	Up Regulated
792923	rnmtl1b	1.64E-03	-0.51	Up Regulated
792966	LOC792966	1.68E-02	-0.53	Up Regulated
793666	popdc2	1.27E-02	-0.83	Up Regulated
794079	pdcd11	5.43E-03	-0.69	Up Regulated
794522	LOC794522	1.89E-04	-0.79	Up Regulated
794891	si:ch211-89f7.4	1.75E-02	-0.85	Up Regulated
795748	LOC795748	3.69E-03	-1.51	Up Regulated
796378	si:dkey-286j15.1	2.51E-03	-0.75	Up Regulated
796814	LOC796814	6.71E-03	-0.81	Up Regulated
797085	LOC797085	1.16E-02	-0.69	Up Regulated
797099	si:dkeyp-84a8.8	8.21E-04	-0.72	Up Regulated
797196	dnajc12	7.61E-04	-0.56	Up Regulated
797198	wu:fk33d07	4.74E-05	-0.87	Up Regulated
797343	zgc:162301	1.60E-03	-0.66	Up Regulated
797346	spint1b	2.27E-02	-0.69	Up Regulated
797675	LOC797675	5.81E-03	-0.87	Up Regulated
798290	LOC798290	2.34E-02	-1.96	Up Regulated
798354	ropn1l	1.85E-02	-0.55	Up Regulated
799423	si:ch211-191i18.3	1.42E-02	-0.76	Up Regulated
799739	spata17	7.11E-03	-0.75	Up Regulated
799782	LOC799782	1.84E-03	-0.58	Up Regulated
100000098	LOC100000098	5.53E-03	-0.55	Up Regulated
100000596	LOC100000596	2.44E-02	-0.67	Up Regulated
100000748	LOC100000748	1.04E-02	-0.73	Up Regulated
100002136	si:ch211-224b1.4	4.12E-03	-0.77	Up Regulated
100002293	zgc:110020	2.55E-02	-0.51	Up Regulated
100002541	zgc:172053	4.57E-03	-0.84	Up Regulated
100003066	LOC100003066	9.07E-03	-0.60	Up Regulated
100003104	LOC100003104	1.62E-02	-0.53	Up Regulated
100003313	zgc:123180	5.41E-03	-0.61	Up Regulated
100004673	zgc:194508	1.12E-02	-0.80	Up Regulated
100005086	si:ch211-93g23.2	1.15E-02	-0.79	Up Regulated
100005148	ar	1.79E-04	-0.54	Up Regulated
100005158	cox11	6.40E-03	-0.57	Up Regulated
100005455	mettl20	5.02E-03	-0.69	Up Regulated
100005763	uck2a	1.61E-02	-0.67	Up Regulated
100006146	LOC100006146	1.42E-02	-0.94	Up Regulated
100007289	si:dkey-21o19.6	2.64E-02	-3.10	Up Regulated
100007926	p2ry11	2.23E-02	-0.62	Up Regulated
100008088	zgc:173556	2.37E-02	-0.62	Up Regulated
100009635	dhx37	3.85E-03	-0.74	Up Regulated
100009650	zgc:158482	8.10E-03	-0.64	Up Regulated
100034415	si:dkey-6a5.3	7.24E-03	-0.50	Up Regulated
100034506	si:ch211-232m10.4	5.23E-03	-0.52	Up Regulated

Table S9: Up/Down regulated genes of mitfa KD.

Gene ID	Gene Symbol	Pvalue	diff_KO_WT	UP/Down Regulated
100037317	cdnf	3.68E-03	-0.79	Up Regulated
100037332	zgc:162608	5.75E-03	-1.39	Up Regulated
100038797	rhcga	6.73E-03	-1.59	Up Regulated
100093702	si:dkey-121a9.3	3.14E-03	-0.91	Up Regulated
100124531	loxl5b	2.10E-02	-0.88	Up Regulated
100126104	zgc:171779	1.03E-02	-1.04	Up Regulated
100126114	zgc:171679	3.31E-03	-0.56	Up Regulated
100136849	zgc:172270	3.79E-03	-0.96	Up Regulated
100137128	hbl4	6.81E-03	-0.74	Up Regulated
100147884	or132-2	2.48E-02	-0.89	Up Regulated
100148342	atp8b1	1.64E-02	-0.68	Up Regulated
100148706	or125-4	2.87E-02	-1.21	Up Regulated
100149100	LOC100149100	1.48E-02	-0.59	Up Regulated
100150665	LOC100150665	2.34E-03	-0.76	Up Regulated
100170836	odf3l2	5.28E-03	-0.80	Up Regulated
100307105	si:rp71-80o10.4	2.39E-02	-0.92	Up Regulated
100317105	si:ch211-145b13.6	6.83E-04	-0.66	Up Regulated
100318879	si:dkey-148f10.4	2.21E-05	-0.71	Up Regulated
100329344	LOC100329344	2.13E-02	-1.13	Up Regulated
100329570	LOC100329570	1.73E-02	-0.68	Up Regulated
100330805	LOC100330805	7.69E-03	-0.64	Up Regulated
100331324	LOC100331324	2.36E-02	-1.13	Up Regulated
100331698	LOC100331698	2.49E-02	-0.90	Up Regulated
100332249	LOC100332249	1.39E-02	-0.76	Up Regulated
100332610	LOC100332610	2.70E-03	-0.94	Up Regulated
100332843	LOC100332843	4.50E-03	-0.61	Up Regulated
100333064	LOC100333064	2.01E-02	-0.53	Up Regulated
100333759	si:ch211-264f7.1	7.08E-03	-0.64	Up Regulated
100334215	LOC100334215	2.95E-02	-0.53	Up Regulated
100334610	LOC100334610	1.10E-02	-1.38	Up Regulated
100334939	LOC100334939	1.11E-02	-0.50	Up Regulated
30066	meis2.1	1.26E-02	0.87	Down Regulated
30101	skia	1.33E-02	0.54	Down Regulated
30147	isl1	7.37E-03	0.91	Down Regulated
30157	isl2a	2.24E-02	0.50	Down Regulated
30207	tyr	1.08E-02	0.77	Down Regulated
30248	calr	2.15E-02	0.53	Down Regulated
30317	hoxb5a	2.45E-02	0.60	Down Regulated
30349	hoxd3a	1.88E-02	0.64	Down Regulated
30418	nr2f1a	9.87E-04	0.78	Down Regulated
30424	nr2f2	1.99E-02	0.54	Down Regulated
30425	pax2a	1.78E-02	0.58	Down Regulated
30463	lhx1a	2.96E-02	0.95	Down Regulated
30501	otx2	1.75E-02	0.88	Down Regulated
30510	tcf7l2	3.45E-05	0.75	Down Regulated
30598	vsx1	1.59E-02	2.44	Down Regulated
30634	nadl1.2	9.35E-03	0.63	Down Regulated
30635	six3a	1.79E-02	0.57	Down Regulated
30656	nadl1.1	1.74E-02	0.53	Down Regulated
30676	glra1	1.71E-03	0.63	Down Regulated
30679	prox1	1.93E-03	0.90	Down Regulated
30701	lef1	8.31E-03	0.58	Down Regulated
30712	snap25a	9.71E-03	0.60	Down Regulated
30726	cntn2	2.29E-02	0.58	Down Regulated
30732	elavl3	2.32E-02	0.52	Down Regulated

Table S9: Up/Down regulated genes of mitfa KD.

Gene ID	Gene Symbol	Pvalue	diff_KO_WT	UP/Down Regulated
30737	elavl4	7.34E-03	0.93	Down Regulated
30745	pdgfra	1.96E-03	0.64	Down Regulated
30749	nr5a2	6.04E-03	0.73	Down Regulated
30766	tal1	9.99E-03	0.61	Down Regulated
57922	celf3	1.39E-02	0.92	Down Regulated
58038	stx1b	8.15E-03	0.70	Down Regulated
58057	pou4f1	5.84E-03	1.00	Down Regulated
58061	hoxa11a	2.63E-02	0.56	Down Regulated
58074	dct	9.50E-06	0.91	Down Regulated
58134	adarb1	4.44E-03	0.89	Down Regulated
60309	robo2	2.31E-02	0.61	Down Regulated
60310	hmx3	1.89E-02	0.54	Down Regulated
64279	psmb11	1.39E-03	0.74	Down Regulated
64603	eomesa	4.32E-04	0.58	Down Regulated
64610	atp1a3a	1.37E-02	1.09	Down Regulated
65231	mdkb	2.05E-02	0.52	Down Regulated
81881	crx	2.15E-02	2.84	Down Regulated
114415	neurod6b	2.32E-02	1.54	Down Regulated
114467	maf	1.43E-02	0.89	Down Regulated
117234	mab21l2	1.60E-02	0.77	Down Regulated
140615	fads2	2.81E-03	1.55	Down Regulated
140618	tfap2a	5.45E-04	0.57	Down Regulated
140621	casp3a	4.35E-03	0.51	Down Regulated
140744	ckbb	1.01E-02	1.04	Down Regulated
142987	dmbx1a	2.78E-02	0.64	Down Regulated
170782	mkrn1	6.32E-03	0.65	Down Regulated
245948	gbx2	8.05E-03	0.66	Down Regulated
246091	mab21l1	6.52E-03	0.81	Down Regulated
259186	pcdh10a	4.44E-03	0.74	Down Regulated
266794	ccng2	9.38E-04	1.00	Down Regulated
266965	tlx3b	9.76E-03	0.54	Down Regulated
280646	lmo1	9.39E-03	1.03	Down Regulated
282554	drd3	1.85E-02	1.49	Down Regulated
286784	arl13b	4.84E-04	0.51	Down Regulated
321113	zgc:65851	1.05E-02	0.57	Down Regulated
321239	pmela	2.85E-03	0.76	Down Regulated
321707	si:ch211-57k11.4	2.92E-03	0.87	Down Regulated
321742	si:ch211-288g17.3	7.96E-03	0.53	Down Regulated
322056	sulf2l	2.76E-03	0.67	Down Regulated
322485	agt	2.01E-02	1.67	Down Regulated
322970	wu:fb78a10	6.11E-03	0.51	Down Regulated
323326	zgc:73225	2.00E-02	0.58	Down Regulated
323342	wu:fb95f11	1.82E-02	0.55	Down Regulated
323473	prdm1a	2.30E-03	0.84	Down Regulated
323699	wu:fc07c11	4.93E-03	0.51	Down Regulated
323706	rtn1a	1.62E-02	0.94	Down Regulated
324153	taf13	5.39E-03	0.65	Down Regulated
324160	wu:fc20g02	1.34E-02	0.73	Down Regulated
324261	sp5l	3.74E-03	1.53	Down Regulated
324285	wu:fc23f11	7.57E-03	0.74	Down Regulated
324416	dpysl5a	8.29E-03	0.71	Down Regulated
324723	zgc:73290	4.93E-03	0.73	Down Regulated
324920	wu:fc48a11	4.90E-03	0.67	Down Regulated
324971	zgc:73142	1.27E-02	0.97	Down Regulated
325288	olig2	2.03E-02	0.90	Down Regulated

Table S9: Up/Down regulated genes of mitfa KD.

Gene ID	Gene Symbol	Pvalue	diff_KO_WT	UP/Down Regulated
325356	rcan3	1.93E-02	0.68	Down Regulated
325372	scrt2	1.99E-03	0.71	Down Regulated
325395	mll5	1.46E-03	0.55	Down Regulated
325402	atp6v0cb	9.96E-03	0.80	Down Regulated
325607	wu:fc92b01	6.09E-03	0.53	Down Regulated
325690	serinc1	2.92E-02	0.56	Down Regulated
327065	rtn1b	7.12E-03	0.88	Down Regulated
327160	apba2	2.74E-02	0.75	Down Regulated
327165	pgam1b	1.17E-02	0.62	Down Regulated
327415	kcnd3	1.41E-02	0.67	Down Regulated
327429	zgc:55318	2.29E-02	0.63	Down Regulated
334035	olfm2	2.02E-02	0.94	Down Regulated
334490	hnrnpu	2.73E-03	0.64	Down Regulated
334798	wu:fa04a07	8.58E-03	0.52	Down Regulated
334932	mdm4	6.96E-04	0.81	Down Regulated
335257	lmbrd2b	8.77E-03	0.57	Down Regulated
335357	syt4	2.31E-02	0.69	Down Regulated
335551	zgc:73062	1.43E-02	0.77	Down Regulated
335667	wu:fk54d01	2.01E-02	0.69	Down Regulated
335699	wu:fk57g06	1.43E-02	0.57	Down Regulated
335707	ptmaa	2.89E-02	0.58	Down Regulated
335737	si:dkey-52h23.1	1.26E-02	0.55	Down Regulated
335836	slc1a2b	1.56E-02	0.89	Down Regulated
335881	kidins220b	9.83E-03	0.73	Down Regulated
336042	wu:fj44g11	1.63E-02	0.64	Down Regulated
336082	nell2b	2.92E-02	0.90	Down Regulated
336089	wu:fj48a08	3.58E-03	0.57	Down Regulated
336099	trim9	7.92E-03	0.74	Down Regulated
336116	bnip3	2.06E-02	0.75	Down Regulated
336227	cdr2l	2.77E-02	0.60	Down Regulated
336248	wu:fj59c10	1.64E-03	0.69	Down Regulated
336281	vamp2	7.46E-03	0.82	Down Regulated
336319	wu:fj63c11	9.76E-05	0.66	Down Regulated
336346	sox4a	5.51E-03	0.90	Down Regulated
337168	nbr1	2.32E-03	0.58	Down Regulated
353179	otx5	1.26E-02	1.40	Down Regulated
360137	dixdc1a	7.96E-03	0.59	Down Regulated
360138	pcdh10b	8.25E-03	0.54	Down Regulated
368211	ptn	2.04E-02	0.61	Down Regulated
368223	gpm6aa	2.17E-02	0.74	Down Regulated
368305	nptna	5.96E-03	0.51	Down Regulated
368329	cdkn1b	1.33E-02	0.51	Down Regulated
368483	nsfa	1.04E-02	0.96	Down Regulated
368752	vat1	5.26E-03	0.65	Down Regulated
373103	pak1	2.96E-02	0.72	Down Regulated
373117	celf2	1.11E-02	0.62	Down Regulated
373872	fyna	1.48E-03	0.69	Down Regulated
378441	gad1b	1.66E-02	0.87	Down Regulated
378456	cmt4b2	4.71E-03	0.57	Down Regulated
378854	zgc:110586	1.66E-02	0.59	Down Regulated
378993	si:ch211-195k18.2	8.49E-03	0.69	Down Regulated
386590	hsp90b1	4.44E-03	0.59	Down Regulated
386701	tubb5	2.24E-02	0.85	Down Regulated
386762	apc	1.41E-02	0.62	Down Regulated
386768	dnajc5aa	3.31E-03	0.59	Down Regulated

Table S9: Up/Down regulated genes of mitfa KD.

Gene ID	Gene Symbol	Pvalue	diff_KO_WT	UP/Down Regulated
387001	wu:fc74b12	2.90E-02	0.57	Down Regulated
387591	ivns1abpa	1.38E-03	0.51	Down Regulated
393118	rap2ip	2.05E-02	0.70	Down Regulated
393119	ankrd12	3.87E-04	0.66	Down Regulated
393229	rbpms2	9.65E-03	0.62	Down Regulated
393546	zgc:65895	1.33E-02	0.59	Down Regulated
393579	slc25a1	2.71E-03	0.84	Down Regulated
393614	cacng2a	1.24E-02	0.89	Down Regulated
393665	ndrg1l	1.76E-02	1.91	Down Regulated
393672	rims2a	1.19E-02	0.78	Down Regulated
393695	zgc:73124	2.42E-02	0.67	Down Regulated
393724	rpe65a	9.41E-03	0.75	Down Regulated
393794	tfap2e	1.08E-02	0.98	Down Regulated
393804	amph	7.76E-03	0.86	Down Regulated
393805	mid1ip1b	1.33E-02	0.91	Down Regulated
393844	znrf1	1.56E-02	0.75	Down Regulated
393875	sox4b	1.81E-02	0.67	Down Regulated
393930	bhlhe22	8.52E-03	0.96	Down Regulated
393944	sncb	3.88E-03	0.83	Down Regulated
393969	gpr19	2.13E-02	0.60	Down Regulated
394032	irx2a	1.04E-02	0.82	Down Regulated
394091	sh3gl2	1.38E-02	0.62	Down Regulated
394110	atp6v1al	1.46E-02	0.69	Down Regulated
394171	shox2	1.49E-02	0.92	Down Regulated
394174	pik3cd	1.98E-02	0.55	Down Regulated
394177	zgc:56685	1.28E-02	0.90	Down Regulated
394239	tal2	7.47E-03	0.98	Down Regulated
402812	pth2	5.56E-03	1.29	Down Regulated
402816	pou4f2	1.10E-02	1.50	Down Regulated
402833	camsap1a	3.50E-03	0.64	Down Regulated
402874	eno2	1.33E-02	0.70	Down Regulated
402929	evlb	1.04E-02	0.80	Down Regulated
402973	nrp1b	6.75E-03	0.71	Down Regulated
402991	gadd45g	1.40E-02	0.51	Down Regulated
402999	irx4a	2.00E-02	1.09	Down Regulated
403005	zgc:77409	7.62E-03	0.59	Down Regulated
403006	nptnb	1.30E-02	0.94	Down Regulated
403053	hirip5	6.13E-03	0.71	Down Regulated
403057	zswim6	2.38E-02	0.75	Down Regulated
403143	sarm1	9.38E-03	0.89	Down Regulated
403308	rtn4rl1	1.36E-02	0.76	Down Regulated
404233	aplp	1.56E-03	0.63	Down Regulated
405796	dlg4	2.58E-02	0.62	Down Regulated
405836	zgc:77058	2.60E-02	1.06	Down Regulated
405844	zgc:77466	1.80E-02	0.68	Down Regulated
405877	mafg2	1.19E-02	0.66	Down Regulated
405890	esrrga	1.44E-02	0.94	Down Regulated
405898	pak7	5.15E-03	0.77	Down Regulated
406239	ccni	4.96E-03	0.80	Down Regulated
406303	tuba2	1.20E-02	1.00	Down Regulated
406364	elmo1	2.83E-02	0.59	Down Regulated
406391	zgc:65870	8.85E-03	0.53	Down Regulated
406443	ywhah	4.72E-03	0.68	Down Regulated
406705	fez1	3.69E-03	0.67	Down Regulated
406834	arglu1a	1.60E-02	0.59	Down Regulated

Table S9: Up/Down regulated genes of mitfa KD.

Gene ID	Gene Symbol	Pvalue	diff_KO_WT	UP/Down Regulated
407077	nkx1.2lb	1.30E-02	0.86	Down Regulated
407734	gpm6ab	1.20E-02	0.75	Down Regulated
407735	gria4a	2.28E-02	0.57	Down Regulated
407979	cpe	2.05E-02	0.79	Down Regulated
407982	zgc:85722	1.67E-02	0.88	Down Regulated
431714	zgc:91968	1.51E-02	3.06	Down Regulated
431740	zgc:91860	4.38E-03	0.50	Down Regulated
431770	mpp2b	2.83E-02	0.53	Down Regulated
432384	rbp2b	8.90E-03	0.74	Down Regulated
436636	cd36	1.41E-02	0.76	Down Regulated
436669	zgc:92710	9.13E-03	1.10	Down Regulated
436679	nr4a2b	1.09E-02	0.93	Down Regulated
436704	rgs20	2.12E-02	0.81	Down Regulated
436712	tspan18b	1.29E-02	0.75	Down Regulated
436736	syt1a	3.00E-02	0.67	Down Regulated
436742	napg	2.82E-02	1.09	Down Regulated
436745	atp2b3a	4.55E-03	0.99	Down Regulated
436781	zgc:92827	4.07E-03	0.51	Down Regulated
436788	cdk5r1b	8.23E-03	1.16	Down Regulated
436793	cbln1	1.53E-02	0.75	Down Regulated
436803	rab3c	1.92E-02	0.84	Down Regulated
436814	rgs7	2.84E-02	0.60	Down Regulated
436815	camk2d2	9.66E-03	1.03	Down Regulated
436822	itm2ca	2.22E-02	0.68	Down Regulated
436920	zgc:92020	1.73E-02	0.86	Down Regulated
436927	pdlim5	1.74E-02	0.52	Down Regulated
436939	slc1a1	1.04E-02	0.83	Down Regulated
437006	nxph1	7.12E-03	1.08	Down Regulated
437022	tyrp1b	6.27E-03	1.03	Down Regulated
437027	rac3a	9.14E-03	0.61	Down Regulated
442926	t2gtl56	4.62E-03	0.77	Down Regulated
445060	b3gat2	1.28E-02	0.62	Down Regulated
445061	dnajb4	1.76E-02	0.51	Down Regulated
445088	c1qb	2.62E-02	0.87	Down Regulated
445123	zgc:100906	3.34E-03	0.77	Down Regulated
445124	chst1	1.67E-02	0.86	Down Regulated
445212	fam49a	3.57E-03	0.61	Down Regulated
445213	zgc:100994	1.92E-02	0.81	Down Regulated
445286	picalml	1.04E-02	0.53	Down Regulated
445374	negr1	1.34E-02	0.50	Down Regulated
445478	epb41l3a	7.28E-03	0.70	Down Regulated
447837	lingo1b	6.54E-03	1.04	Down Regulated
447906	zgc:101095	1.89E-02	0.50	Down Regulated
447912	vac14	2.36E-03	0.62	Down Regulated
449546	ric8a	2.34E-02	0.68	Down Regulated
449554	rbfox1	1.75E-02	0.95	Down Regulated
449664	arfip2a	3.84E-03	0.54	Down Regulated
449794	zgc:101846	1.30E-02	0.57	Down Regulated
449864	grin2da	2.76E-02	0.69	Down Regulated
449991	kctd15a	2.93E-02	0.53	Down Regulated
450043	ank2	1.69E-02	0.70	Down Regulated
474317	irx6a	1.27E-02	1.42	Down Regulated
474318	irx4b	2.90E-02	0.59	Down Regulated
492490	zgc:103663	1.86E-02	1.12	Down Regulated
492566	im:7141573	1.05E-02	0.86	Down Regulated

Table S9: Up/Down regulated genes of mitfa KD.

Gene ID	Gene Symbol	Pvalue	diff_KO_WT	UP/Down Regulated
492792	gpr137ba	1.71E-02	0.56	Down Regulated
492818	zgc:101525	1.94E-02	0.74	Down Regulated
493587	pcdh2ac	2.77E-02	0.63	Down Regulated
494033	tfap2c	6.61E-04	0.58	Down Regulated
494050	zgc:101847	2.15E-02	0.51	Down Regulated
494450	slc6a5	7.35E-03	0.58	Down Regulated
494492	slc17a6a	2.47E-02	0.61	Down Regulated
494533	prkar1aa	7.33E-04	0.53	Down Regulated
497072	zgc:198371	2.09E-03	0.57	Down Regulated
497127	pcdh1a4	1.08E-02	0.79	Down Regulated
497281	ext1c	2.77E-02	0.76	Down Regulated
497284	zgc:92612	2.27E-02	0.81	Down Regulated
497419	zgc:165461	8.77E-03	0.80	Down Regulated
503940	spen	1.87E-03	0.51	Down Regulated
541370	gnb2	2.10E-02	0.59	Down Regulated
541373	nbea	2.15E-02	0.80	Down Regulated
541396	islr2	1.81E-02	0.83	Down Regulated
541438	tmem22	9.55E-03	0.71	Down Regulated
541533	scrt1b	2.17E-04	0.75	Down Regulated
541540	kcnab1	9.15E-03	0.56	Down Regulated
548345	nalcn	1.18E-02	0.83	Down Regulated
548606	erc1a	2.75E-02	0.62	Down Regulated
548607	si:dkey-22o20.1	1.70E-02	0.76	Down Regulated
550228	golga7ba	2.20E-02	0.56	Down Regulated
550267	kif3a	8.42E-03	0.50	Down Regulated
550377	sema6dl	2.47E-02	0.59	Down Regulated
550397	c1qtnf4	1.14E-02	0.70	Down Regulated
550403	gad2	1.09E-02	0.72	Down Regulated
550405	lhx9	1.31E-03	0.83	Down Regulated
550511	zgc:110314	1.16E-02	0.75	Down Regulated
550545	tfap2b	8.03E-03	1.04	Down Regulated
553082	klf7l	7.90E-04	0.54	Down Regulated
553166	dpysl3	4.28E-03	0.85	Down Regulated
553186	barhl1.1	3.15E-03	0.76	Down Regulated
553266	arrb1	1.06E-03	0.53	Down Regulated
553281	abcc8	1.04E-02	0.59	Down Regulated
553300	dchs1	5.12E-03	0.52	Down Regulated
553331	lnx2a	2.59E-02	0.82	Down Regulated
553336	lppr3a	1.54E-02	0.56	Down Regulated
553377	prg4b	2.03E-02	0.57	Down Regulated
553402	si:ch211-145n14.1	2.56E-02	0.67	Down Regulated
553410	dpysl5b	1.55E-02	0.85	Down Regulated
553411	dpysl4	2.53E-03	0.75	Down Regulated
553422	LOC553422	1.87E-02	1.02	Down Regulated
553451	LOC553451	2.12E-02	0.54	Down Regulated
553542	zgc:109889	1.15E-02	0.72	Down Regulated
553565	zgc:109985	5.67E-03	0.53	Down Regulated
553590	zgc:110158	5.46E-04	0.66	Down Regulated
553591	ttc39c	1.76E-03	0.66	Down Regulated
553607	camk2n1a	5.71E-03	0.53	Down Regulated
553611	six6b	5.22E-03	0.67	Down Regulated
553619	lactb	1.45E-03	0.65	Down Regulated
553691	atp6v0a1b	2.78E-02	0.63	Down Regulated
553750	mtmr1a	3.55E-03	0.89	Down Regulated
553774	lmo3	7.82E-03	0.83	Down Regulated

Table S9: Up/Down regulated genes of mitfa KD.

Gene ID	Gene Symbol	Pvalue	diff_KO_WT	UP/Down Regulated
554123	3-Sep	9.91E-04	0.67	Down Regulated
554998	pdia4	7.69E-03	0.51	Down Regulated
555391	trim3b	1.22E-03	0.55	Down Regulated
555414	wu:fa07b03	2.29E-02	0.81	Down Regulated
555437	LOC555437	2.31E-02	0.70	Down Regulated
555640	fam184a	1.40E-02	0.97	Down Regulated
556080	si:ch211-214j24.7	1.14E-02	0.56	Down Regulated
556251	LOC556251	2.40E-02	0.63	Down Regulated
556338	si:dkeyp-22b2.2	1.83E-02	0.56	Down Regulated
556348	wu:fj35c01	4.97E-03	0.57	Down Regulated
556409	g3bp2	2.24E-03	0.92	Down Regulated
556453	LOC556453	1.05E-02	0.66	Down Regulated
556537	nrcam	5.30E-03	0.81	Down Regulated
556619	chrna4	1.40E-02	0.66	Down Regulated
556665	nfia	2.77E-03	0.73	Down Regulated
556789	stxbp5a	2.27E-02	0.64	Down Regulated
556928	phactr3a	1.68E-02	0.96	Down Regulated
557002	myt1	9.23E-03	0.78	Down Regulated
557148	mdm1	1.81E-02	0.61	Down Regulated
557162	prdm8b	1.76E-02	0.95	Down Regulated
557319	im:7142942	5.51E-03	0.70	Down Regulated
557451	daam1a	7.81E-03	0.52	Down Regulated
557463	si:ch211-227c6.4	1.65E-02	0.66	Down Regulated
557556	dlgap3	1.15E-02	0.54	Down Regulated
557661	sox14	6.40E-03	1.16	Down Regulated
557675	c1galt1a	1.43E-02	0.78	Down Regulated
557717	stxbp1b	2.35E-02	1.12	Down Regulated
557772	si:ch211-250g4.3	2.86E-03	0.99	Down Regulated
558164	si:ch211-246m4.3	1.09E-02	1.26	Down Regulated
558206	cspg5a	1.83E-02	0.88	Down Regulated
558311	slc45a2	4.04E-03	0.51	Down Regulated
558326	nrxn2a	1.35E-02	0.90	Down Regulated
558453	si:dkey-12o15.1	6.28E-03	0.50	Down Regulated
558560	lcor	1.77E-03	0.64	Down Regulated
558790	sult3st1	1.61E-02	0.56	Down Regulated
558810	ppp2r2bb	6.35E-03	0.63	Down Regulated
558834	si:dkey-92k1.12	9.04E-04	0.53	Down Regulated
558981	si:ch211-81a5.7	2.19E-03	0.60	Down Regulated
558997	dclk1	1.99E-02	1.00	Down Regulated
559029	si:ch211-214j24.9	1.96E-02	0.78	Down Regulated
559358	cspg5b	1.95E-02	1.48	Down Regulated
559375	si:ch211-108c6.2	2.81E-02	1.04	Down Regulated
559447	scn1lab	9.24E-03	0.71	Down Regulated
559505	myt1la	1.91E-02	1.18	Down Regulated
559643	LOC559643	2.45E-02	0.57	Down Regulated
559668	slc29a4	1.42E-02	0.74	Down Regulated
559732	ttc9b	1.57E-02	0.74	Down Regulated
559786	dtnb	9.69E-03	0.50	Down Regulated
559792	LOC559792	5.97E-03	0.72	Down Regulated
559868	LOC559868	1.43E-02	0.74	Down Regulated
559941	si:ch211-1o14.5	2.52E-02	0.73	Down Regulated
560062	si:ch73-60i13.2	1.51E-02	0.65	Down Regulated
560168	coro1b	3.34E-03	0.76	Down Regulated
560413	si:ch211-15i6.4	5.43E-03	0.53	Down Regulated
560560	ywhag2	1.72E-02	0.67	Down Regulated

Table S9: Up/Down regulated genes of mitfa KD.

Gene ID	Gene Symbol	Pvalue	diff_KO_WT	UP/Down Regulated
560594	garnl3	5.47E-03	0.51	Down Regulated
560805	LOC560805	2.79E-02	0.83	Down Regulated
561162	vsnl1b	1.22E-02	0.70	Down Regulated
561285	LOC561285	6.65E-03	0.58	Down Regulated
561336	pcdh17	2.52E-02	0.55	Down Regulated
561408	LOC561408	2.55E-03	0.81	Down Regulated
561525	dync1i1	3.05E-03	0.77	Down Regulated
561653	vps45	1.60E-02	0.51	Down Regulated
561679	col11a2	2.65E-02	0.70	Down Regulated
561769	wu:fq41a10	3.48E-03	0.61	Down Regulated
561879	asap1	9.61E-03	0.97	Down Regulated
561905	mlphb	1.08E-02	1.41	Down Regulated
561943	neurla	1.62E-02	0.75	Down Regulated
562019	wu:fc07b10	9.56E-03	0.62	Down Regulated
562136	si:dkey-13n15.2	1.20E-02	0.92	Down Regulated
562282	LOC562282	1.81E-02	0.72	Down Regulated
562381	si:dkey-76p14.4	2.37E-03	1.05	Down Regulated
562450	LOC562450	1.19E-02	0.64	Down Regulated
562633	LOC562633	1.78E-02	0.64	Down Regulated
563117	si:dkey-174m14.3	2.69E-02	0.78	Down Regulated
563166	rimbp2	9.60E-04	0.61	Down Regulated
563197	odz2	1.43E-02	0.72	Down Regulated
563244	zgc:158337	1.72E-02	0.68	Down Regulated
563252	LOC563252	1.70E-02	0.73	Down Regulated
563352	LOC563352	4.64E-03	0.65	Down Regulated
563353	si:ch211-106n13.3	3.04E-03	0.81	Down Regulated
563377	rph3ab	6.54E-03	0.55	Down Regulated
563561	LOC563561	8.49E-03	1.29	Down Regulated
563578	tbc1d9	2.86E-02	0.56	Down Regulated
563730	si:ch211-106n13.1	1.65E-02	0.75	Down Regulated
563802	kcnh2l	7.73E-03	0.82	Down Regulated
563874	LOC563874	2.25E-02	1.76	Down Regulated
563942	kat2b	1.57E-02	0.88	Down Regulated
564019	tle1	8.31E-03	0.77	Down Regulated
564147	glsa	4.69E-04	0.68	Down Regulated
564165	zgc:172323	1.19E-03	1.97	Down Regulated
564367	si:ch211-233a24.2	2.25E-02	0.53	Down Regulated
564403	si:ch211-153j24.3	3.38E-04	0.80	Down Regulated
564462	sc:d0316	2.29E-02	0.80	Down Regulated
564915	gnaz	2.63E-02	0.53	Down Regulated
564943	lingo1a	2.38E-02	0.67	Down Regulated
565021	lrrc4.1	6.20E-03	0.57	Down Regulated
565109	zgc:153779	1.28E-02	1.04	Down Regulated
565492	aplnrb	2.64E-02	0.67	Down Regulated
565507	apba1	4.84E-03	0.97	Down Regulated
565531	nrxn1a	6.45E-03	0.67	Down Regulated
565700	sat1a	4.53E-04	0.63	Down Regulated
565771	apc2	1.01E-02	0.91	Down Regulated
565842	si:dkey-266j7.1	2.19E-03	0.58	Down Regulated
565863	LOC565863	8.34E-03	0.68	Down Regulated
566117	zgc:163001	9.07E-03	0.80	Down Regulated
566219	dpf1	2.66E-02	0.79	Down Regulated
566408	si:ch73-6k16.1	2.65E-02	0.52	Down Regulated
566456	LOC566456	2.72E-02	0.81	Down Regulated
566591	furinb	2.67E-03	0.50	Down Regulated

Table S9: Up/Down regulated genes of mitfa KD.

Gene ID	Gene Symbol	Pvalue	diff_KO_WT	UP/Down Regulated
566596	si:dkey-153k10.9	1.12E-02	0.72	Down Regulated
566608	upf3a	1.64E-02	0.64	Down Regulated
566685	arrdc3a	2.56E-03	0.55	Down Regulated
567095	ptprt	2.73E-02	0.96	Down Regulated
567151	eif3hb	8.08E-03	0.51	Down Regulated
567190	git2b	3.22E-03	0.54	Down Regulated
567355	gpr27	1.04E-02	0.94	Down Regulated
567419	oca2	6.48E-04	0.86	Down Regulated
567479	ssh1b	1.76E-02	0.63	Down Regulated
567731	fam69b	1.30E-02	0.66	Down Regulated
567751	ebf3	6.72E-03	0.84	Down Regulated
568055	zgc:109984	2.60E-02	1.17	Down Regulated
568165	si:ch211-278j3.3	2.85E-02	0.50	Down Regulated
568315	nfatc1	2.05E-03	0.50	Down Regulated
568367	si:ch211-160j6.2	5.57E-03	0.59	Down Regulated
568464	zfpm2b	1.07E-02	1.37	Down Regulated
568618	si:dkey-110c1.7	6.59E-03	0.66	Down Regulated
568668	ntrk3b	1.15E-02	0.51	Down Regulated
568981	ankrd13b	4.49E-03	0.64	Down Regulated
568996	zfpm2a	2.22E-02	1.75	Down Regulated
569012	zgc:194665	2.46E-02	0.63	Down Regulated
569698	mapk10	6.60E-03	0.76	Down Regulated
569855	cacng2b	2.30E-02	0.92	Down Regulated
569946	zgc:162329	2.69E-02	0.58	Down Regulated
570011	wu:fb77g05	1.17E-02	1.08	Down Regulated
570082	mxd1	4.73E-03	0.62	Down Regulated
570094	zgc:165621	1.26E-03	0.61	Down Regulated
570147	add2	1.00E-02	0.69	Down Regulated
570312	slc24a5	2.93E-02	0.58	Down Regulated
570321	si:dkeyp-117h8.2	1.16E-02	0.93	Down Regulated
570606	zgc:194983	1.97E-02	0.60	Down Regulated
570693	LOC570693	7.70E-03	1.15	Down Regulated
570841	trpc1	6.55E-03	0.60	Down Regulated
570960	zgc:158824	1.01E-02	0.82	Down Regulated
571143	mmp24	1.39E-02	0.89	Down Regulated
571355	ampd2	2.22E-02	0.60	Down Regulated
571425	zgc:165603	2.03E-02	0.71	Down Regulated
571586	LOC571586	1.87E-02	0.63	Down Regulated
571664	fstl4	1.64E-02	0.86	Down Regulated
571943	si:dkeyp-35f11.3	2.26E-02	1.20	Down Regulated
572084	zgc:158291	7.18E-03	1.21	Down Regulated
572369	kirrel3	2.23E-02	1.00	Down Regulated
573122	tuba1l	1.79E-02	1.00	Down Regulated
664741	inpp4a	1.24E-02	0.57	Down Regulated
664748	shox	2.15E-02	1.03	Down Regulated
664755	zgc:110045	2.06E-02	0.95	Down Regulated
678521	zgc:136878	2.68E-02	1.69	Down Regulated
678523	zgc:136874	1.37E-02	0.55	Down Regulated
678560	cacnb4b	1.58E-02	0.50	Down Regulated
678600	rab40c	2.40E-02	0.76	Down Regulated
678641	dab1b	8.45E-03	1.07	Down Regulated
678650	bcl11aa	9.96E-04	0.68	Down Regulated
678652	zgc:136817	5.86E-03	0.55	Down Regulated
692255	dachc	2.96E-02	0.64	Down Regulated
692305	ndrg4	1.54E-02	0.99	Down Regulated

Table S9: Up/Down regulated genes of mitfa KD.

Gene ID	Gene Symbol	Pvalue	diff_KO_WT	UP/Down Regulated
692351	dab1a	2.77E-02	0.90	Down Regulated
751668	gck	2.76E-03	0.97	Down Regulated
751673	zgc:153240	3.10E-03	1.05	Down Regulated
767685	prkar1b	1.76E-02	0.59	Down Regulated
767702	tmem178	2.48E-02	0.51	Down Regulated
767738	abi1b	9.93E-04	0.57	Down Regulated
767809	runx1t1	5.40E-03	0.75	Down Regulated
768130	hmga1b	6.11E-03	0.78	Down Regulated
768183	gabra1	1.24E-02	1.09	Down Regulated
768198	klhl24	2.83E-02	0.55	Down Regulated
768297	zgc:153423	2.89E-02	0.86	Down Regulated
777704	zgc:123035	2.85E-02	0.77	Down Regulated
777708	zgc:153892	2.43E-02	0.50	Down Regulated
777735	fgf12	1.34E-02	0.52	Down Regulated
790943	phyhipla	1.41E-02	0.59	Down Regulated
791150	gripap1	2.61E-02	0.52	Down Regulated
791170	zgc:158791	1.63E-03	0.78	Down Regulated
791179	si:dkey-19f23.3	8.62E-03	0.52	Down Regulated
792435	astn1	1.70E-02	1.00	Down Regulated
793087	zgc:73189	9.58E-03	0.87	Down Regulated
793364	LOC793364	3.26E-04	1.08	Down Regulated
793374	LOC793374	2.63E-02	0.56	Down Regulated
793907	igfbp1b	1.01E-02	1.27	Down Regulated
794175	wu:fj40f01	1.06E-02	0.62	Down Regulated
794258	LOC794258	1.34E-02	0.89	Down Regulated
794574	ppp3ca	9.74E-03	0.85	Down Regulated
794783	zgc:162150	1.74E-02	1.73	Down Regulated
796144	LOC796144	1.44E-02	0.59	Down Regulated
796345	dnajc6	1.29E-02	1.00	Down Regulated
796410	LOC796410	2.44E-02	0.68	Down Regulated
796981	LOC796981	7.20E-03	0.62	Down Regulated
797939	LOC797939	2.61E-02	0.55	Down Regulated
798555	LOC798555	8.00E-03	0.90	Down Regulated
798575	slc32a1	1.44E-02	0.93	Down Regulated
100000252	pik3r3	2.67E-02	0.52	Down Regulated
100000342	mapta	1.21E-02	0.68	Down Regulated
100000711	ngb	4.59E-03	0.66	Down Regulated
100000890	LOC100000890	1.38E-02	0.60	Down Regulated
100001552	pcyt1ba	2.07E-02	0.81	Down Regulated
100001699	barhl2	6.61E-03	0.83	Down Regulated
100002110	stxbp6l	6.04E-05	0.79	Down Regulated
100002795	LOC100002795	1.28E-02	0.71	Down Regulated
100003026	dctn1b	5.48E-03	0.63	Down Regulated
100003493	si:dkey-229p15.1	6.78E-03	1.34	Down Regulated
100003558	ablim1a	5.16E-03	0.81	Down Regulated
100003563	stmn2b	5.21E-03	1.01	Down Regulated
100003819	zgc:172133	1.78E-02	0.70	Down Regulated
100003938	LOC100003938	1.93E-02	0.63	Down Regulated
100004140	LOC100004140	1.32E-02	0.64	Down Regulated
100004523	LOC100004523	2.11E-02	0.54	Down Regulated
100004913	phyhiplb	5.01E-03	0.53	Down Regulated
100005195	LOC100005195	2.28E-03	0.54	Down Regulated
100005267	LOC100005267	2.37E-02	0.54	Down Regulated
100005846	LOC100005846	1.20E-02	0.86	Down Regulated
100006371	zgc:171639	1.07E-02	0.80	Down Regulated

Table S9: Up/Down regulated genes of mitfa KD.

Gene ID	Gene Symbol	Pvalue	diff_KO_WT	UP/Down Regulated
100006588	LOC100006588	1.84E-02	0.54	Down Regulated
100006754	LOC100006754	1.98E-02	0.60	Down Regulated
100006857	LOC100006857	1.01E-02	0.71	Down Regulated
100006858	csnk1e	8.68E-03	0.81	Down Regulated
100006895	LOC100006895	5.04E-04	0.88	Down Regulated
100007164	si:rp71-1g18.10	2.91E-02	0.60	Down Regulated
100007304	LOC100007304	1.18E-03	0.79	Down Regulated
100008101	nr4a2a	2.49E-02	0.82	Down Regulated
100034631	si:dkeyp-39e9.3	1.44E-02	0.89	Down Regulated
100101642	ralgps1	2.87E-02	1.03	Down Regulated
100101649	zgc:165647	1.13E-02	0.70	Down Regulated
100126805	zgc:173905	1.81E-03	0.60	Down Regulated
100126812	pnpla8	9.33E-03	0.57	Down Regulated
100149357	rims1a	2.19E-02	0.59	Down Regulated
100149670	cdk19	2.91E-02	0.92	Down Regulated
100149996	LOC100149996	1.23E-02	0.69	Down Regulated
100150414	LOC100150414	8.55E-03	0.95	Down Regulated
100151220	map1aa	8.47E-03	0.53	Down Regulated
100151347	fb06f03	2.26E-03	0.51	Down Regulated
100170805	si:dkey-6n6.7	2.60E-02	0.61	Down Regulated
100307098	si:dkey-246l19.2	8.75E-04	0.52	Down Regulated
100319240	serp2	2.15E-02	0.89	Down Regulated
100329926	LOC100329926	1.36E-02	0.63	Down Regulated
100330023	slitrk5	2.25E-02	0.99	Down Regulated
100330524	LOC100330524	2.55E-03	1.25	Down Regulated
100331262	LOC100331262	2.75E-02	0.78	Down Regulated
100331387	LOC100331387	9.86E-03	0.96	Down Regulated
100331916	LOC100331916	6.25E-03	1.04	Down Regulated
100331925	LOC100331925	6.88E-03	0.81	Down Regulated
100332125	LOC100332125	1.06E-02	0.62	Down Regulated
100333211	zfhx3	3.65E-03	0.56	Down Regulated
100333238	LOC100333238	6.98E-03	0.60	Down Regulated
100333537	ephb1	2.04E-02	0.58	Down Regulated
100333973	LOC100333973	2.39E-02	0.93	Down Regulated
100334620	LOC100334620	2.25E-02	0.64	Down Regulated
100498671	ccdc88ab	8.64E-03	1.03	Down Regulated
