# Supplementary material for: Analysis of a Gene Regulatory Cascade Mediating Circadian Rhythm in Zebrafish
Source: PLoS Comput Biol. 2013 Feb 28;9(2):e1002940. doi: 10.1371/journal.pcbi.1002940 (PMC3585402; doi:10.1371/journal.pcbi.1002940)
Supplement: Table S10 — Comparison of our light-induced genes with Gavriouchkina et al (2010) and Weger et al. (2011) studies. (PDF) [file pcbi.1002940.s015.pdf]

Table S10: Comparison of our light-induced genes with Gavriouchkina et al (2010) and Weger et al. (2011) studies.

Light-indese	GeneID	Symbol	description	Experiment Source*
Fast Light-induced	30674	tef	thyrotroph embryonic factor	G & W
Fast Light-induced	83776	cry5	cryptochrome 5	G & W
Fast Light-induced	140633	per2	period homolog 2 (Drosophila)	G & W
Fast Light-induced	394039	zgc:66475	zgc:66475	G & W
Fast Light-induced	406605	zgc:85644	zgc:85644	G & W
Fast Light-induced	450011	tspo	translocator protein	G & W
Fast Light-induced	565155	hsp90a.2	heat shock protein 90-alpha 2	G & W
Fast Light-induced	570063	wdr76	WD repeat domain 76	G & W
Fast Light-induced	100004133	ddb2	damage-specific DNA binding protein 2	G & W
Slow Light-induced	573209	cry2a	cryptochrome 2a	G & W
Fast Light-induced	373084	hig1	hypoxia induced gene 1	G
Fast Light-induced	393253	neil1	nei endonuclease VIII-like 1 (E. coli)	G
Fast Light-induced	415184	msrb3	methionine sulfoxide reductase B3	G
Slow Light-induced	83780	cry2b	cryptochrome 2b	G
Fast Light-induced	58215	fech	ferrochelatase	W
Fast Light-induced	336492	ptgds	prostaglandin D2 synthase	W
Fast Light-induced	373099	guca1c	guanylate cyclase activator 1C	W
Fast Light-induced	393167	zgc:56136	zgc:56136	W
Fast Light-induced	393884	sdha	succinate dehydrogenase complex	W
Fast Light-induced	402986	cry-dash	cryptochrome DASH	W
Fast Light-induced	436678	arr3a	arrestin 3a	W
Fast Light-induced	436959	blvrb	biliverdin reductase B (flavin reductase (NADPH))	W
Fast Light-induced	447914	cox17	COX17 cytochrome c oxidase assembly homolog (S. cerevisiae)	W
Fast Light-induced	541386	xpc	xeroderma pigmentosum	W
Fast Light-induced	553695	zgc:112255	zgc:112255	W
Fast Light-induced	553766	zgc:109977	zgc:109977	W
Fast Light-induced	556393	zgc:153679	zgc:153679	W
Fast Light-induced	556875	tmem177	transmembrane protein 177	W
Fast Light-induced	558648	selo	selenoprotein O	W
Fast Light-induced	562149	sdhb	succinate dehydrogenase complex	W
Fast Light-induced	563956	si:busm1-265n4.4	si:busm1-265n4.4	W
Fast Light-induced	568061	napepld	N-acyl phosphatidylethanolamine phospholipase D	W

Table S10: Comparison of our light-induced genes with Gavriouchkina et al (2010) and Weger et al. (2011) studies.

Light-indese	GeneID	Symbol	description	Experiment Source*
Fast Light-induced	568835	slc27a6	solute carrier family 27 (fatty acid transporter)	W
Fast Light-induced	678653	unc119b	unc-119 homolog b (C. elegans)	W
Fast Light-induced	751701	zgc:153154	zgc:153154	W
Fast Light-induced	768159	si:ch211-195b13.1	si:ch211-195b13.1	W
Fast Light-induced	100003670	sepx1b	selenoprotein X	W
Slow Light-induced	30077	rbp4	retinol binding protein 4	W
Slow Light-induced	58099	arntl2	aryl hydrocarbon receptor nuclear translocator-like 2	W
Slow Light-induced	352915	sepw1	selenoprotein W	W
Slow Light-induced	352927	clock3	clock homolog 3 (mouse)	W
Slow Light-induced	492783	dhrs13	dehydrogenase/reductase (SDR family) member 13	W
Slow Light-induced	559626	crtac1a	cartilage acidic protein 1a	W
Slow Light-induced	724003	zgc:136474	zgc:136474	W

* G were obtained from Gavriouchkina et al(2010)[36].
W were obtained from Weger et al(2011)[37].
