# Supplementary material for: Analysis of a Gene Regulatory Cascade Mediating Circadian Rhythm in Zebrafish
Source: PLoS Comput Biol. 2013 Feb 28;9(2):e1002940. doi: 10.1371/journal.pcbi.1002940 (PMC3585402; doi:10.1371/journal.pcbi.1002940)
Supplement: Text S1 — Supplementary Materials and Methods. (DOC) [file pcbi.1002940.s016.doc]

**Supporting Information for**

**An­alysis of a Gene Regulatory Cascade mediating Circadian Rhythm in Zebrafish**

Ying Li^1,§^, Guang Li^1,§^, Haifang Wang^1^, Jiulin Du^2^, Jun Yan^1,*^

^1^CAS-MPG Partner Institute for Computational Biology, Shanghai Institutes of Biological Sciences, Chinese Academy of Sciences, 320 Yue Yang Road, Shanghai, 200031, China ^2^Institute of Neuroscience, Shanghai Institutes of Biological Sciences, Chinese Academy of Sciences, 320 Yue Yang Road, Shanghai, 200031, China

^§^ These authors contributed equally to this work

^*^ To whom correspondence should be addressed. Email: junyan@picb.ac.cn

**Running head:** Circadian Gene Regulatory Cascade in Zebrafish

**Keywords**: circadian rhythm; light entrainment; tissue specificity; transcription factor; zebrafish; melanogenesis

**Supplementary** **Materials and Methods**

**Larval zebrafish locomotor activity assay**

On 5dpf of development, single larva was placed in a 96-well plate (Cat. 167008, NUNC) individually, which allowed tracking of each larva simultaneously and prevented them from interfering the activity of each other. Locomotor activity of larval zebrafish was monitored by an automated video-tracking system (ViewPoint S.A.). This system (Figure S1A) included an enclosed box with an infrared light (850nm, ViewPoint S.A.) and white light matrix in the bottom. The 96-well plate placed on the matrix was illuminated by uniformly distributed white light from 9:00 to 23:00 and continuously illuminated by infrared light. An infrared camera (640 pixels ×480 pixels, 25Fps, 1/3” CS M, 1.4 aperture, 2.8-12mm adapted lens, Point Grey Research Inc.) was placed above the 96-well plate and connected with a computer. Images (25 frames/s) and videos were recorded using the infrared camera and signals were transmitted to computer and then processed by software ZebraLab3.10 (ViewPoint S.A.). The locomotion data of each larva under light and dark conditions were analyzed using videotrack quantization mode so that each image was converted to a digital value according to its luminosity. The data were further analyzed using a customized program in R programming language.

**Functional analyses of circadian genes**

The functional annotations for zebrafish genes were obtained from the Gene Ontology (GO) project (www.geneontology.org) and KEGG pathway from KEGG project (www.genome.jp/kegg/) using their Entrez GENE IDs. In GO analysis, we used Perl CPAN go-perl module to find all paths (terms and intervening relationships) to the root. We used a background including 6,597 zebrafish genes matched to GO terms excluding IEA evidence codes. We calculated p-values using Fisher's test against all GO categories, KEGG pathways and tissue or cell types. The tissue or cell type expression information for zebrafish genes were parsed from the ZFIN databases ([http://zfin.org](http://zfin.org/)) using a customized Perl script together with the relationship of Zebrafish Anatomical Terms.

**Real-time PCR**

Real-time PCR was performed as described before . The first strand cDNA synthesis of total RNA was performed with SuperScript II (Invitrogen). 500ng total RNA of each larvae sample with random hexamers (250ng) and dNTP mix (final concentration 0.5mM) was denatured at 65°C for 5min. Then, 1×First-Strand buffer, DTT (final concentration 0.01M), RNaseOUT (40 units) were added and the mixture was incubated at 25°C for 2min for annealing. RT was performed by adding SuperScript II enzyme (200 units) and then incubating at 25 °C for 10 min and 42°C for 50 min. The reaction was terminated by heating the sample to 70°C for 15min. Real-time PCR was performed in a volume of 20μl including 10μl 2×SYBR Green I Master Mix (Roche), 3μl PCR-grade water, 5μl RT product and 2 μl primers (1μM) on LightCycler 480 machine (Roche). The amplification cycle consisted of 1 cycle of 5min at 95°C, 40 cycles of 10s at 95°C, 20s at 58°C, 20s at 72°C, followed by a melting curve. The PCR primers were designed using Primer 3 and *β-actin* was used as reference. The primer sequences of genes tested are listed in Table S5. The specificity of PCR reaction was checked by melting curve analysis. The critical threshold (C_T_) value is the PCR cycle number where the PCR growth curve crosses a defined threshold in the linear range of the reaction. Relative log2 expression values are calculated from , where is the maximum value of C_T_ in tested samples.

***Mitfa* mopholino (MO) microarray analysis**

Two batches of *mitfa* MO samples together with wild type (WT) and MO control samples were used for microarray assay. One batch was collected at 48 hpf for KD: *mitfa* MO (9ng injection); CON: standard control MO (9ng injection). The other batch was collected at 50hpf for KD1: *mitfa* MO (9ng injection); KD2: *mitfa* MO (13ng injection); CON: standard control MO (9ng injection). Each sample had 50 individual larvae treated together. Our analysis showed that there were minor differences between 48hpf and 50hpf *mitfa* MO samples and between 13ng vs. 9ng injected *mitfa* MO samples. Thus, we considered KD, KD1, KD2 samples as one group and all WT and CON samples as another group. Differential expressed genes were identified using student’s t-test between the two groups. P value < 0.02 and fold change more than 2-fold were chosen as the criteria so that the previously real-time PCR validated genes including *tyr*, *tyrp1b*, *dct,* and *slc24a5* were included as down-regulated genes in *mitfa* MO in microarray analysis.

**Drug treatment**

Antalarmin (Sigma) was dissolved in dimethylsulfoxide (DMSO, Sigma) at a stock concentration of 10mM. Wild type larvae raised in 14h: 10h LD cycle at 28°C were distributed to 35mm dish and each dish had 20 larvae in 5ml E3 buffer. Larvae were exposed to antalarmin for 10h at final concentration of 10μΜ and were sampled at CT0 and CT12 on 5dpf respectively. No larva in the samples was dead during this process.

**Supplementary Reference**

1. Shao C, Liu Y, Ruan H, Li Y, Wang H, et al. (2010) Shotgun proteomics analysis of hibernating arctic ground squirrels. Mol Cell Proteomics 9: 313-326.
